# Multiomics-Based Profiling of the Fecal Microbiome Reveals Potential Disease-Specific Signatures in Pediatric IBD (PIBD) †

**DOI:** 10.3390/biom15050746

**Published:** 2025-05-21

**Authors:** Anita H. DeSantis, Kristina Buss, Keaton M. Coker, Brad A. Pasternak, Jinhua Chi, Jeffrey S. Patterson, Haiwei Gu, Peter W. Jurutka, Todd R. Sandrin

**Affiliations:** 1 School of Mathematical and Natural Sciences, Arizona State University, 4701 W. Thunderbird Rd, Glendale, AZ 85306, USA; anita.desantis@asu.edu (A.H.D.); keatoncoker1@gmail.com (K.M.C.); peter.jurutka@asu.edu (P.W.J.); 2 Biosciences Core, Arizona State University, 1001 S. McAllister Ave, Tempe, AZ 85281, USA; kristina.buss@asu.edu; 3 Phoenix Children’s Hospital, 1919 E. Thomas Rd, Phoenix, AZ 85016, USA; bpasternak@phoenixchildrens.com; 4 College of Health Solutions, Health North Building, Arizona State University, 550 N. 3rd St, Suite 501, Phoenix, AZ 85004, USA; jinhua.chi@asu.edu (J.C.); jspatte5@asu.edu (J.S.P.); haiweigu@asu.edu (H.G.); 5 College of Medicine, University of Arizona, 475 N. 5th St, Phoenix, AZ 85004, USA; 6 Center for Health through Microbiomes, Arizona State University, 1001 S. McAllister Ave, Tempe, AZ 85281, USA

**Keywords:** microbiome, biomarkers, inflammatory bowel disease, pediatric inflammatory bowel disease, Crohn’s Disease, Ulcerative Colitis, multiomics, metatranscriptomics, metagenomics, metabolomics

## Abstract

Inflammatory bowel disease (IBD), which includes Crohn’s Disease (CD) and Ulcerative Colitis (UC), is a chronic gastrointestinal (GI) disorder affecting 1 in 100 people in the United States. Pediatric IBD (PIBD) is estimated to impact 15 per 100,000 children in North America. Factors such as the gut microbiome (GM), genetic predisposition to the disease, and certain environmental factors are thought to be involved in pathogenesis. However, the pathophysiology of IBD is incompletely understood, and diagnostic biomarkers and effective treatments, particularly for PIBD, are limited. Recent work suggests that these factors may interact to influence disease development, and multiomic approaches have emerged as promising tools to elucidate the pathophysiology. We employed metagenomics, metabolomics- and metatranscriptomics-based approaches to examine the microbiome, its genetic potential, and its activity to identify factors associated with PIBD. Metagenomics-based analyses revealed pathways such as octane oxidation and glycolysis that were differentially expressed in UC patients. Additionally, metatranscriptomics-based analyses suggested enrichment of glycan degradation and two component systems in UC samples as well as protein processing in the endoplasmic reticulum, ribosome, and protein export in CD and UC samples. In addition, metabolomics-based approaches revealed patterns of differentially abundant metabolites between healthy and PIBD individuals. Interestingly, overall microbiome community composition (as measured by alpha and beta diversity indices) did not appear to be associated with PIBD. However, we observed a small number of differentially abundant taxa in UC versus healthy controls, including members of the Classes *Gammaproteobacteria* and *Clostridia* as well as members of the Family *Rikenellaceae*. Accordingly, when identifying potential biomarkers for PIBD, our results suggest that multiomics-based approaches afford enhanced potential to detect putative biomarkers for PIBD compared to microbiome community composition sequence data alone.

## 1. Introduction

Pediatric inflammatory bowel disease (PIBD) is a chronic, multifactorial gastrointestinal (GI) condition that affects approximately 15 per 100,000 children in North America [1,2,3,4]. PIBD encompasses two main subtypes: Crohn’s Disease (CD) and Ulcerative Colitis (UC), both marked by alternating periods of inflammation and remission [2,5,6]. During inflammation phases, symptoms such as weight loss, abdominal pain, and bloody diarrhea are most prominent [2,5,6]. The pathophysiology of PIBD remains incompletely understood, and both diagnostic biomarkers and effective treatments are limited. Environmental factors, as well as genetic influences on the immune system and gut microbiota, are key contributors to PIBD pathogenesis, rather than ancestry or ethnicity [6,7,8,9]. Compared to adult IBD, PIBD often follows a more severe disease course, with extensive GI involvement, frequent surgical interventions, and unique challenges such as delayed growth and puberty, along with psychological impacts on body image [2,10,11]. The global prevalence of PIBD has steadily increased, particularly in newly industrialized and developing countries [1,8,12]. Particularly, while the incidence of adult IBD has stabilized in early industrialized regions during the 21st century, it has continued to rise in PIBD with an ongoing increase in the overall prevalence of both adult IBD and PIBD [13]. The growing case burden, coupled with the lifelong need for medical care among PIBD patients, imposes significant direct and indirect costs on healthcare systems [8,14]. Consequently, there is a pressing need for more effective treatments, early diagnostic tools, and a greater research focus on pediatric-specific aspects of this disease.

One key factor in PIBD pathophysiology is the intestinal microbiome, consisting of the microbial community and its collective genetic material residing in the gut [9,15]. Under normal conditions, this symbiotic relationship helps maintain immune system balance and supports the overall health of the host [6,14]. However, dysbiosis, characterized by the gain or loss of specific bacterial groups, the imbalance of microbiome and host interactions, and unfavorable changes in bacterial composition can disrupt this equilibrium and contribute to the onset, progression, and severity of PIBD [7,14,16]. Nonetheless, the role of the gut microbiome in IBD is still poorly understood, and questions remain, including whether IBD causes changes in the microbiome, or if dysbiosis is the result of inflammation after IBD onset [14,15]. To address these unanswered questions more effectively, small subunit ribosomal RNA (16S rRNA) amplicon sequencing has been used [6]; however, this method has been criticized for providing only limited taxonomic and functional information [6]. As a result, there is a growing need for more comprehensive analytical tools, and multiomics-based methods have been proposed to complement existing sequencing data and enhance our understanding of the interactions between PIBD and the microbiome.

Multiomic tools including metatranscriptomics, metagenomics, metaproteomics, and metabolomics have already been successfully applied in cancer research; however, their application to the study of inflammatory diseases, including PIBD, has been slower due to limited collaboration and resources [17]. Advances in sequencing platforms and bioinformatic analyses hold promises for bridging this gap, enabling deeper insights into the diverse interactions among microbes, their interplay with the host, and their contributions to PIBD pathogenesis [14,18]. Given the complexity of multifactorial diseases like PIBD, single-component or taxonomical and compositional studies alone are likely insufficient to fully elucidate underlying mechanisms [14,17]. Instead, a focus on functional profiling and the identification of relevant microbiome-mediated mechanisms using multiomic and bioinformatic approaches is essential [14,17,19]. While previous studies have effectively leveraged these techniques to explore disease mechanisms in broader IBD research, further investigations specifically targeting PIBD are urgently needed [19,20,21,22,23,24]. Additionally, advancements in multiomics tools and their applications could pave the way for personalized, precision medicine approaches in the future [24].

To address these gaps in PIBD research, we employed a multiomic approach to identify factors associated with this disorder and to examine the gut microbiome, its genetic potential, and activity. By integrating metabolomics and metatranscriptomics, we sought to gain deeper insight into the role of the microbiome activity in PIBD pathophysiology. Beyond identifying potential biomarkers, our goal was to evaluate whether multiomics-based methodologies can complement amplicon-based findings and offer superior potential for detecting putative biomarkers for PIBD compared to microbiome community composition sequencing data alone.

## 2. Materials and Methods

### 2.1. Patient Recruitment

The study was conducted in accordance with the Declaration of Helsinki and approved by the Institutional Review Board of the Phoenix Children’s Hospital. Participants were recruited and samples collected from pediatric IBD patients and healthy controls at Phoenix Children’s Hospital (PCH). The study cohort (n = 26) included pediatric patients aged 2–21 years: 16 females and 10 males. Patients with histologically verified IBD were included in the study. Patients who had been exposed to antibiotics or had a bowel cleanout over the past 30 days and those who had undergone bowel surgery were excluded. Dietary changes were not an exclusion criterion in this study. Additionally, healthy controls with no histological evidence of IBD were included. If biopsy of healthy subjects showed IBD, they were enrolled into the appropriate disease group. Patients undergoing evaluation for possible IBD but with a normal endoscopy were included in the healthy control group. The Research Coordinator initially identified those patients that met all the study requirements. Once selected, the patient’s provider was contacted, and during the next appointment, information was shared with the family about the study. If agreement to participate in the study was reached, a consent form was signed, and a quick blood draw for vitamin D was completed. Patients were provided with a stool sample home kit that was returned to PCH after collection.

Demographic data were collected from the electronic medical record (EMR) of both healthy controls and disease patients including age, race, ethnicity, gender, disease duration, disorder type, disorder severity (utilizing the Pediatric Crohn’s Disease Activity Index (PCDAI), Pediatric Ulcerative Colitis Activity Index (PUCAI), and Marsh Criteria), other relevant co-morbidities, dietary habits (vegetarian, vegan, etc.), supplement intake, yogurt intake, probiotics, quality of life, socioeconomics, zip code, body mass index, and sun exposure time (to account for vitamin D levels in their serum). Blood serum was collected in red-top (no-gel) vials either during the patient’s initial evaluation, at the time of a scheduled endoscopy, or during a standard-of-care blood draw. Samples were allowed to clot for 30 min and centrifuged within 2 h of collection. Serum was transferred into a clean, plastic vial and frozen at −80 °C until batch shipment. Stool specimens were collected for microbiome analysis prior to colonoscopy and associated prep or at a much later stage post-colonoscopy. Deidentified stool and serum samples were sent to Arizona State University for evaluation, including assessment of serum 25-hydroxyvitamin D levels by a third-party analytical laboratory.

### 2.2. Nucleic Acid Extraction

Microbial DNA was extracted from samples using the DNeasy PowerSoil Kit (Qiagen; Venlo, The Netherlands) following manufacturer’s directions. Microbial RNA was extracted using the RNeasy PowerMicrobiome Kit (Qiagen; Venlo, The Netherlands) following the manufacturer’s directions.

### 2.3. 16S Microbiome Library Preparation

The V4 region of the 16S rRNA gene (approximately 291 bp in length) was isolated from microbial DNA with the barcoded primer set 515f/806r (IDT; Newark, NJ, USA) specified by Caporaso [25], following the protocol given by the Earth Microbiome Project [26] (EMP) ((https://earthmicrobiome.org/protocols-and-standards/16s/) (accessed 14 May 2025) for library preparation. PCR amplifications for each sample were conducted in duplicate, then pooled and quantified using Qubit (Fisher Scientific, Invitrogen; Pittsburgh, PA, USA). A blank sample was included during the library preparation as a control for extraneous nucleic acid contamination. DNA (100 ng per sample) was pooled, and the normalized pool was then cleaned using KAPA Pure Beads (Roche, KAPA; Basel, Switzerland). The pool was quantified with the Qubit (Fisher Scientific, Invitrogen; Pittsburgh, PA, USA) and diluted and denatured in preparation for sequencing. It was then loaded on the Illumina MiSeq (Illumina; San Diego, CA, USA) according to manufacturer specifications and sequenced on a v2 2 × 50 paired end-run module.

### 2.4. Sequencing Quality Control Analysis

As a general confirmation of sequencing quality, a FastQC [27] analysis was run on each sample; this generated an HTML report on each sample providing a quick visual overview of sequence quality in areas such as sequence duplication, base-call confidence, and adapter contamination. MultiQC [28] was run on the FastQC reports to generate a visual summary of the sequencing for the entire project.

### 2.5. Metagenomics Library Preparation

Illumina compatible Genomic DNA libraries were generated on an Eppendorf epMotion 5075 liquid handler (Eppendorf; Hamburg, Germany) using Kapa Biosystem’s Hyper plus library preparation kit (Roche, KAPA; Basel, Switzerland). DNA was enzymatically sheared to approximately 300 bp fragments, end repaired, and A-tailed as described in the Kapa protocol. Illumina-compatible adapters with unique indexes (IDT #00989130v2; Newark, NJ, USA) were ligated on each sample individually. The adapter-ligated molecules were cleaned using Kapa pure beads (Roche, KAPA; Basel, Switzerland) and amplified with Kapa’s HIFI enzyme (Roche, KAPA; Basel, Switzerland). Each library was then analyzed for fragment size on a Tapestation 4200 (Agilent; Santa Clara, CA, USA) and quantified by Qubit (Fisher Scientific, Invitrogen; Pittsburgh, PA, USA) before multiplex pooling and sequencing using an S4 300 flow cell on the NovaSeq platform (Illumina; San Diego, CA, USA) at the Collaborative Sequencing Center (TGen; Phoenix, AZ, USA).

### 2.6. Transcriptomics Library Preparation

Extracted RNA was ribodepleted using the MICROBExpress Bacterial mRNA Enrichment Kit (Fisher Scientific, Invitrogen; Pittsburgh, PA, USA) following the manufacturer’s protocol. Ribodepletion was verified with a Tapestation 4200 (Agilent; Santa Clara, CA, USA) and libraries were built from the ribodepleted RNA using a KAPA RNA HyperPrep library preparation kit (Roche, KAPA; Basel, Switzerland) following the manufacturer’s directions. RNA was enzymatically fragmented to approximately 300 bp fragments. The first cDNA strand was synthesized using random priming followed by the second strand of cDNA synthesis and A-tailing. Illumina-compatible adapters with unique indexes (IDT #00989130v2; Newark, NJ, USA) were ligated on each sample individually and libraries were amplified. Each library was then analyzed for fragment size on a Tapestation 4200 (Agilent; Santa Clara, CA, USA) and quantified by Qubit (Fisher Scientific, Invitrogen; Pittsburgh, PA, USA) before multiplex pooling and sequencing using an S4 300 flow cell on the NovaSeq platform (Illumina; San Diego, CA, USA) at the Collaborative Sequencing Center (TGen; Phoenix, AZ, USA).

### 2.7. Fecal Sample Preparation for LC-MS

Each fecal sample (~20 mg) was homogenized in an Eppendorf tube with 200 µL LC-MS grade methanol (MeOH, Fisher Scientific; Pittsburgh, PA, USA): PBS (Healthcare Life Sciences; Logan, UT, USA), (4:1, v:v, containing 1810.5 μM ^13^C_3_-lactate and 142 μM ^13^C_5_-glutamic Acid) using a Bullet Blender homogenizer (Next Advance; Averill Park, NY, USA). Then, 800 µL of MeOH:PBS (4:1, v:v, containing 1810.5 μM ^13^C_3_-lactate and 142 μM ^13^C_5_-glutamic Acid) was added to each sample. Samples were vortexed for 10 s, and afterwards, kept at −20 °C for 30 min. They were then sonicated in an ice bath for 30 min and centrifuged at 21,694× *g* for 10 min (4 °C). Then, 800 µL of supernatant was transferred to a new Eppendorf tube. Samples were dried under a vacuum using a CentriVap Concentrator (Labconco; Fort Scott, KS, USA). Prior to MS analysis, the obtained residue was reconstituted in 150 μL 40% PBS/60% LC-MS grade acetonitrile (ACN, Fisher Scientific; Pittsburgh, PA, USA). A pooled quality control (QC) sample was prepared by combining aliquots from all the study samples. The standard compounds for the selected metabolites were purchased from Sigma-Aldrich (Saint Louis, MO, USA) and Fisher Scientific (Pittsburgh, PA, USA). Deionized water was supplied by an in-house water purification system from EMD Millipore (Billerica, MA, USA).

### 2.8. Untargeted LC-MS Metabolomics

The untargeted LC-MS metabolomics method, described here, was modeled after several previously developed and implemented studies [29,30,31,32,33,34,35]. The LC-MS experiments were performed on a Thermo Vanquish UPLC-Exploris 240 Orbitrap MS instrument (Waltham, MA, USA). Each sample was injected twice: 10 µL for analysis using the negative ionization mode and 4 µL using the positive ionization mode. Both chromatographic separations were performed in the hydrophilic interaction chromatography (HILIC) mode on a Waters XBridge BEH Amide column (150 mm × 2.1 mm, 2.5 µm particle size, Waters Corporation; Milford, MA, USA). These methods were chosen as HILIC utilizes a polar stationary phase and is a robust method for detecting relatively polar metabolites. Reverse phase liquid chromatography, a nonpolar stationary phase (C18, T3, etc.), is used to detect hydrophobic metabolites and has been utilized in several former metabolomic studies. However, HILIC is becoming more commonly used in newer metabolomic analyses due to its ability to detect highly polar or ionic compounds that are relevant to many studies and would be undetected with RPLC-MS [30,36,37,38,39]. The flow rate was maintained at 0.3 mL/min, with the autosampler temperature set to 4 °C and the column compartment set at 40 °C. The mobile phase was composed of Solvents A (10 mM ammonium acetate (Fisher Scientific; Pittsburgh, PA, USA), 10 mM ammonium hydroxide (Sigma-Aldrich; Saint Louis, MO, USA) in 95% H_2_O/5% ACN) and B (10 mM ammonium acetate, 10 mM ammonium hydroxide in 95% ACN/5% H_2_O). Following an initial 1 min isocratic elution of 90% solvent B, the percentage of solvent B was reduced to 40% at t = 11 min. This composition was maintained at 40% solvent B for 4 min (t = 15 min), after which the percentage of Solvent B was gradually increased back to 90% in preparation for the next injection. Using a mass spectrometer equipped with an electrospray ionization (ESI) source, we collected untargeted data over a mass range of 70 to 1050 *m*/*z*.

To identify peaks in the MS spectra, we utilized extensive in-house standards of approximately 600 metabolites and supplemented this with searches against external resources, including the HMDB library, LipidMaps database, METLIN database, and commercial databases such as mzCloud, Metabolika, and ChemSpider. The absolute intensity threshold for the MS data extraction was 1000, with a mass accuracy limit of 5 ppm. Identifications and annotations utilized available data for retention time (RT), exact mass (MS), MS/MS fragmentation pattern, and isotopic pattern. We processed the aqueous metabolomics data using Thermo Compound Discoverer 3.3 software, which handled peak picking, alignment, and normalization of the untargeted data. To improve rigor, only signals or peaks with CV < 20% across quality control (QC) pools and those present in more than 80% of all samples were included for further analysis.

### 2.9. 16S Amplicon Analysis

All analysis was performed in Qiime2 (versions 2022.2 and 2024.10). DADA2 was used to remove errors, noise, and chimeras from the data and detect amplicon sequence variants (see Supplemental Table S1). Each ASV was matched to 16S reference databases (Greengenes2 and Silva138.99, both using a naïve Bayes classifier for the 515F/806R region) for taxonomic classifications. For phylogenetic diversity metrics (including Faith’s alpha diversity and weighted/unweighted UniFrac beta diversity), mafft and FastTree were used within Qiime2 to align all ASVs and construct a rooted tree for the sample set. Other alpha diversity metrics calculated include Shannon’s diversity index, observed features, and Pielou’s evenness, while other beta diversity metrics include Jaccard and Bray-Curtis distances. Relative differential abundance between sample groups was calculated with ANCOM-BC using the Qiime2 plugin composition.

### 2.10. Metagenomic and Metatranscriptomic Sequence QC

Trimmomatic was used to filter each sample to retain only reads with an average Phred quality score of 15 for each 4 bp window and to trim adapter content from each read. The trimmed and filtered reads were aligned to the GRCh38_102 human reference genome and all read pairs where both forward and reverse reads mapped to the human genome were removed. Metagenomic samples were aligned with Bowtie2 and metatranscriptomic samples were aligned with the RNA-optimized aligner STAR (See Supplemental Tables S2 and S3).

### 2.11. Assembly-Free Taxonomic Classification

Metagenomics samples were classified by Kraken2’s k-mer algorithm using the PlusPF database, which contains all RefSeq sequences from archaea, bacteria, virus, plasmid, protozoa, fungal, and human genomes. Alpha and beta diversity metrics were calculated based on the taxonomic profile of each sample’s population.

Metagenomic and metatranscriptomic samples were classified with MetaPhlAn4, which maps reads against a marker database (ChocoPhlAn3, derived from UniProt and NCBI) and computes each clade’s coverage based on the alignment coverage across all markers within that clade. Using taxonomic classifications, samples were clustered using principal coordinates analysis.

### 2.12. Assembly-Free Functional Pathway Analysis

HUMAnN3 was used to characterize the functional profile of each metagenomic and metatranscriptomic sample using similarity to clusters of UniRef proteins at 90% or 50% identity. Differential functional profiles between groups were modeled using MaAsLin3 with incorporated generalized linear models, with an adjusted *p*-value cutoff of 0.05 to consider a pathway differentially represented. Differential expression for metatranscriptomic samples was modeled with MTXmodel to identify pathways-by-taxa with over or underrepresented expression in any sample group, by taking into consideration the underlying population structure and gene copy number differences that influence transcript abundance differences. An adjusted *p*-value of ≤0.05 was again used to establish statistical significance for a differential pathway.

### 2.13. Assembly-Based Analysis

Reads from all metagenomic samples were combined to create a contig-level reference assembly using the program MegaHit. Metagenomics reads were aligned back to the reference with Bowtie2 and metatranscriptomics reads were aligned with Hisat2 for gene abundance profiling. CAT was used for protein sequence prediction and contig taxonomic classification, and functional annotations for predicted proteins were made with MicrobeAnnotator.

Gene counts were obtained with HTSeq-count for metagenomics and Hisat2 for metatranscriptomics. Functional genomic potential for each population was determined and compared using predicted KEGG pathway terms for annotated genes. Differentially expressed genes were determined using standard RNA packages including DESeq2, edgeR, and NOISeq, retaining genes with an adjusted *p*-value ≤ 0.05 for all three identification methods. MicrobiomeProfiler was used to evaluate all KEGG pathways associated with the differentially expressed genes to determine functional differences between sample groups.

### 2.14. MetaboAnalyst Analysis

Metabolomics data were assessed with MetaboAnalyst 6.0 [https://www.metaboanalyst.ca/], (accessed on 20 March 2025 and 14 May 2025) a comprehensive web-based tool for metabolomic data analysis. The dataset was initially normalized by median value and log transformed. Principle component analysis (PCA) was utilized to examine variance and similarities between the sample groups, whereas one-way ANOVA was performed to elicit significant metabolites between the PIBD patients and healthy controls. To visualize metabolomic differences, a heatmap was generated using Ward hierarchical clustering and Pearson’s correlation coefficient in which the top 50 differentially abundant metabolites were included. Subsequently, only the biologically relevant metabolites (n = 21) within the group of 50 were used to generate the heatmap. Additionally, a metabolic pathway analysis was performed with *Escherichia coli* K-12 (MG1655) pathway library between UC and CD samples.

## 3. Results

For this study, stool samples (n = 26) were collected from PIBD patients and healthy controls at Phoenix Children’s Hospital. The study group consisted of 10 Crohn’s Disease (CD) and 12 Ulcerative Colitis (UC) patients, as well as four healthy controls (Table 1). Among the participants, 16 were female and 10 male. The average age was 13 years, and participants were predominantly white (84.6%), with a smaller proportion identifying as Hispanic (11.5%) and Asian (3.8%).

### 3.1. Microbiome Population Analysis with 16S rRNA Sequencing

**Figure 1:**
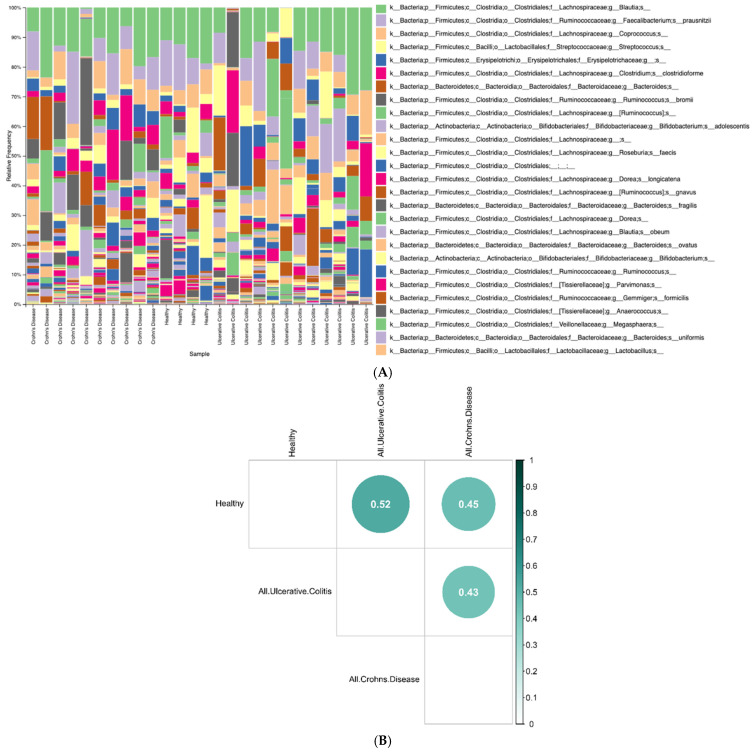

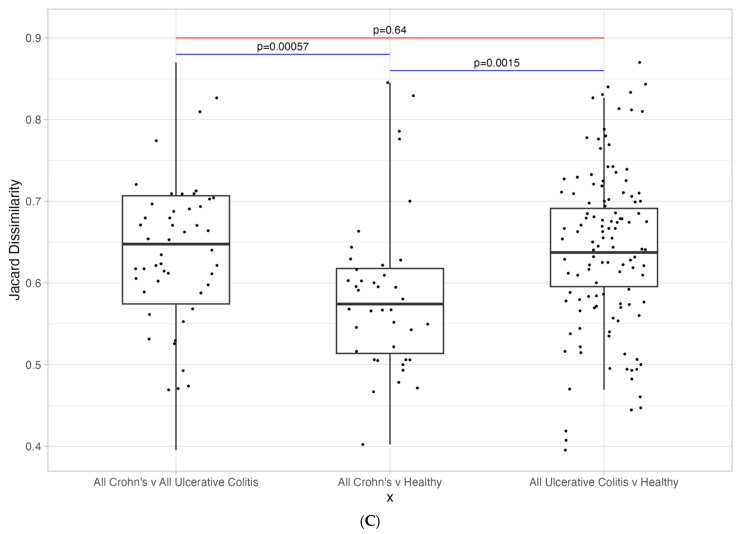
(**A**) Overall microbiome composition did not vary as a function of disease state based on relative frequency of taxonomic classification. The species-level taxonomic assignments from 16S sequencing were matched with the Greengenes database. (**B**) CD, UC, and healthy samples appear similar; however, CD and healthy samples seem slightly more similar to one another than to UC samples based on the correlation plot showing Jaccard dissimilarity indices between groups by diagnosis. The darker and larger circles correspond to higher levels of dissimilarity. (**C**) Beta diversity dissimilarities are comparable between CD, UC, and healthy samples, but CD and healthy samples are more similar to one another than to UC samples. Box plots show all Jaccard dissimilarity index values between groups based on the presence and absence of species-level taxa from 16S sequencing. The dissimilarities between each pair of groups are represented by the distributions on the box-plot, and the distributions of each comparison are then compared to each other using a Wilcox test.

To investigate the microbiome population composition and possible dysbiosis in our PIBD sample set, we first performed 16S rRNA sequencing and analysis. The role of the gut microbiota in PIBD has been extensively investigated and previous studies have reported inconsistent findings in microbiome diversity and abundance between PIBD patients and healthy subjects [6,40]. Maukonen et al., for example, found that the abundances of *Lachnospiraceae* and *Coriobacteriaceae* were lower in IBD patients compared to controls [41], whereas Schwiertz et al. found decreased numbers of *F praunsitzii* and increased abundance of *E.coli* in the microbiota of pediatric CD patients [42]. Our microbial community composition analysis using 16S rRNA sequence data, in contrast, revealed no differences in overall population composition. (Figure 1A). This suggests a complex and dynamic gut microbiome across all groups, with high levels of individual variability. We observed, however, with adjusted *p*-value of 0.05 and ANCOM-BC analysis on the 16S data, no significantly different taxa between CD and healthy samples. Conversely, the Class *Gammaproteobacteria* was enriched in UC samples compared to healthy controls (log2 fold change 2.903 ± 0.889 and p-adjusted 0.017). Additionally, at the Family level, *Rikenellaceae* was depleted in UC samples (log2fold change −2.339 ± 0.674 and p-adjusted 0.023). Similarly, Michail et al. found *Gammaproteobacteria* enriched in their UC sample set [43] whereas Papa et al. discovered prevalent *Gammaproteobacteria* when investigating the relevance of the different microbes to active disease state [44]. Like our findings, Jacobs et al. reported decreased *Rikenellaceae* in IBD [45]. These findings reinforce the complexity and variability of the gut microbiome in PIBD, suggesting that broad community composition may not be a reliable disease marker. However, the observed enrichment of *Gammaproteobacteria* in UC and depletion of *Rikenellaceae* align with prior studies, indicating that specific microbial alterations could still play a role in disease pathophysiology. Additionally, using an adjusted *p*-value of 0.05, ANCOM-BC analysis on the 16S data at species level found no significantly different taxa between CD and healthy samples (see Supplemental Tables S4 and S5). However, *Gemmiger formicilis* and *Clostridium clostridioforme* were both significantly depleted in UC samples as compared to healthy samples, with a log2 fold change of −4.273 ± 0.499 and an adjusted *p*-value of 1.437 × 10^−15^ for *G. formicillis* and a log2 fold change of −2.975 ± 0.648 and an adjusted *p*-value of 5.662 × 10^−4^ for *C. clostridioforme*. Previous studies have also discovered similar findings [46,47].

In addition to taxonomic assignments, we utilized a presence/absence matrix of the 16S species classifications to calculate a Jaccard dissimilarity index to obtain beta diversity information between the group populations (Figure 1B,C). We observed similarities across CD, UC, and healthy samples; however, CD and healthy samples appeared slightly more similar to one another than to UC samples (UC vs. CD samples have an average Jaccard dissimilarity of 0.6413, UC vs. healthy 0.6358, and CD vs. healthy 0.5859). Based on an additional Wilcox test, these results are statistically significant and show that CD and healthy samples are less dissimilar than either are to UC samples (CD vs. healthy dissimilarity versus UC vs. healthy dissimilarity is significantly different with *p*-value 0.0015, CD vs. healthy dissimilarity versus CD vs. UC dissimilarity is significantly different with *p*-value 0.00057, and CD vs. UC dissimilarity versus UC vs. healthy dissimilarity is not significantly different with a *p*-value of 0.64. Cluster analysis (Figure 1C) did not show a strong correlation between diagnosis and microbial community composition, suggesting that potential PIBD pathophysiology likely involves other factors beyond overall microbial community composition alone.

### 3.2. Metagenomics and Functional Pathway Analysis

**Figure 2:**
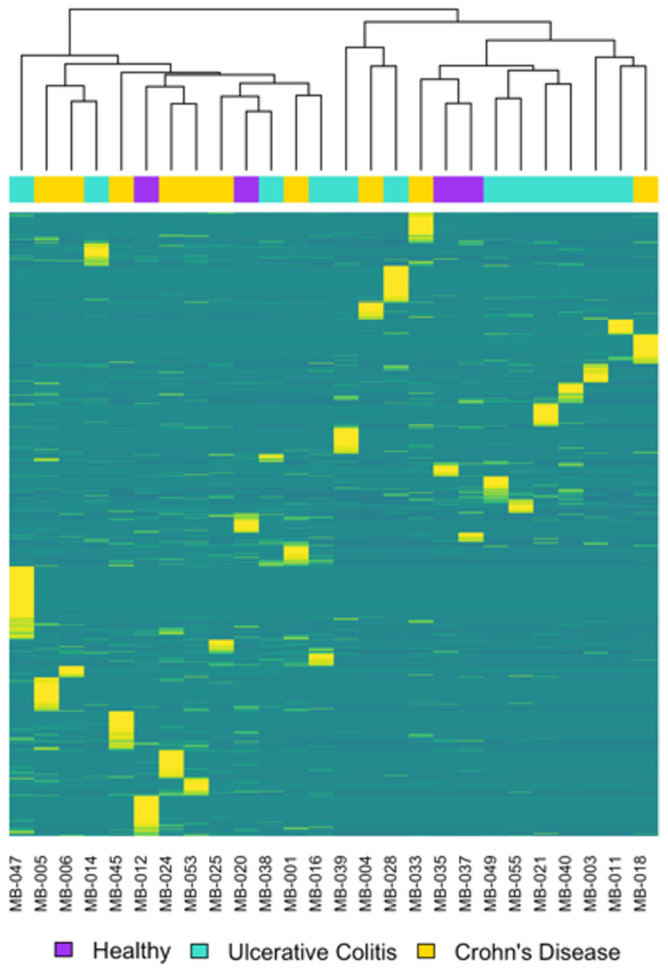
No significant clustering of samples was found in the microbiome composition between the study groups using whole genome sequencing data. The heatmap shows the relative abundance of taxonomic species identified using Metaphlan. The top dendrogram shows the clustering of samples, with each sample color-coded according to diagnosis. In the figure, yellow indicates species with higher abundance, transitioning to blue for species with lower abundance.

Because 16S rRNA analysis revealed limited overall microbial community differences, we utilized metagenomic analyses to assess and to compare the genetic potential of the fecal microbiome. These methods target the entire genomic content of the microbes and their functional potential [48,49]. Our microbiome composition analysis from this whole genome sequencing data revealed no significant differences in either CD or UC samples and the healthy controls, as visualized in the Metaphlan classification heatmap (Figure 2). This was confirmed with MaAsLin3 using TSS normalization and log transformation (see Supplemental Tables S6 and S7). These tools have been used in previous IBD studies and Knoll et al., for example, discovered new insights into the IBD microbiome, validated previous 16S rRNA analyses, and evaluated antibiotic resistance genes with the metagenomic tools in their comparative sibling study [50]. Thoman et al., conversely, explored the connection of IBD and depression [51]. While finding 16S rRNA and metagenomic tools useful for predicting UC in children, Zuo et al. additionally discovered limited differences between these two sequencing approaches [52].

**Figure 3:**
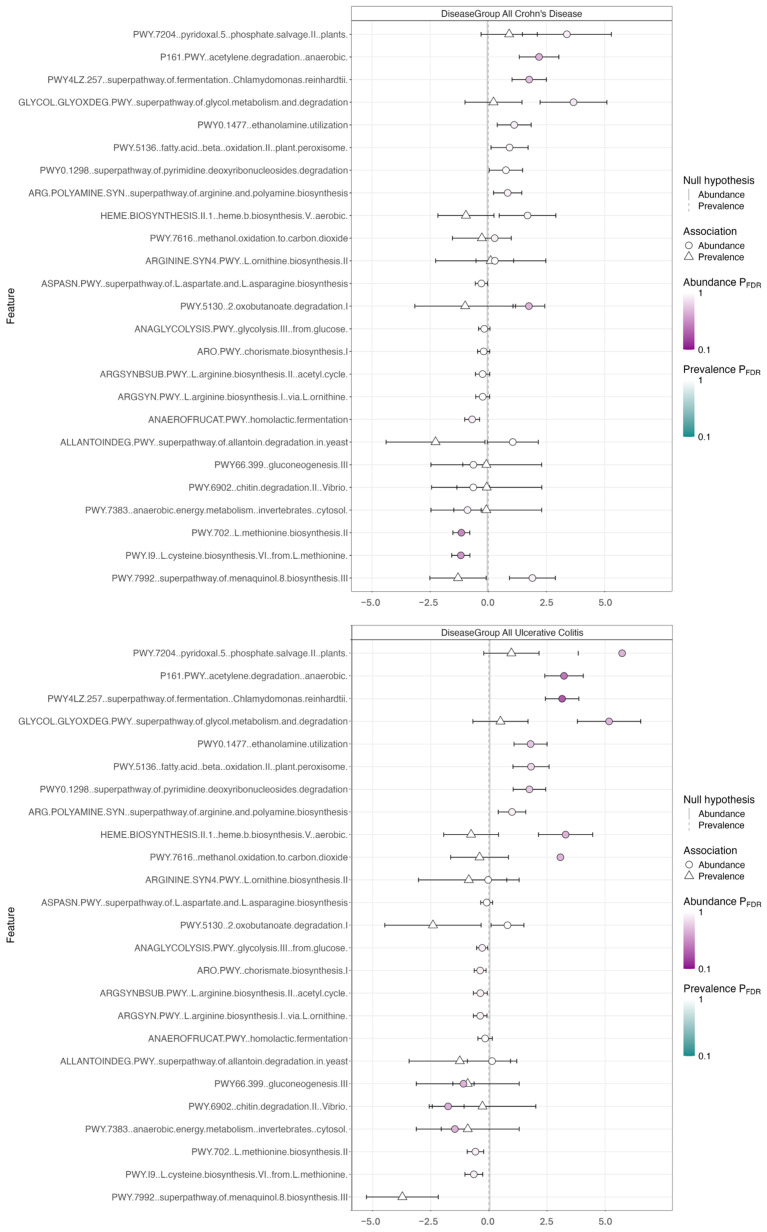
Fermentation superpathway and acetylene degradation pathway appear enriched in CD and UC versus healthy controls based on the comparison of the genomic potential of functional pathways using assembly-free analysis for all CD and all UC vs. healthy.

In addition to the microbiome composition analysis, we assessed the genetic potential of the fecal microbiome of each sample using assembly-free analysis of functional pathways (Figure 3). The coefficient plot for all CD and all UC versus healthy showed enrichment in the fermentation superpathway (*p*-value 2.55 × 10^−4^ for UC vs. Healthy and 2.05 × 10^−2^ for CD vs. Healthy) and acetylene degradation pathway (enriched *p*-value 8.38 × 10^−4^ for UC vs. Healthy, *p*-value 1.39 × 10^−2^ for CD vs. Healthy). Pathways were enriched in PIBD patients compared to healthy controls (see Supplemental Tables S8 and S9); however, q-values suggested a lack of statistical significance, and underscore a need for further exploration with larger sample sizes.

### 3.3. Metatranscriptomics and KEGG Pathway Analyses

**Figure 4:**
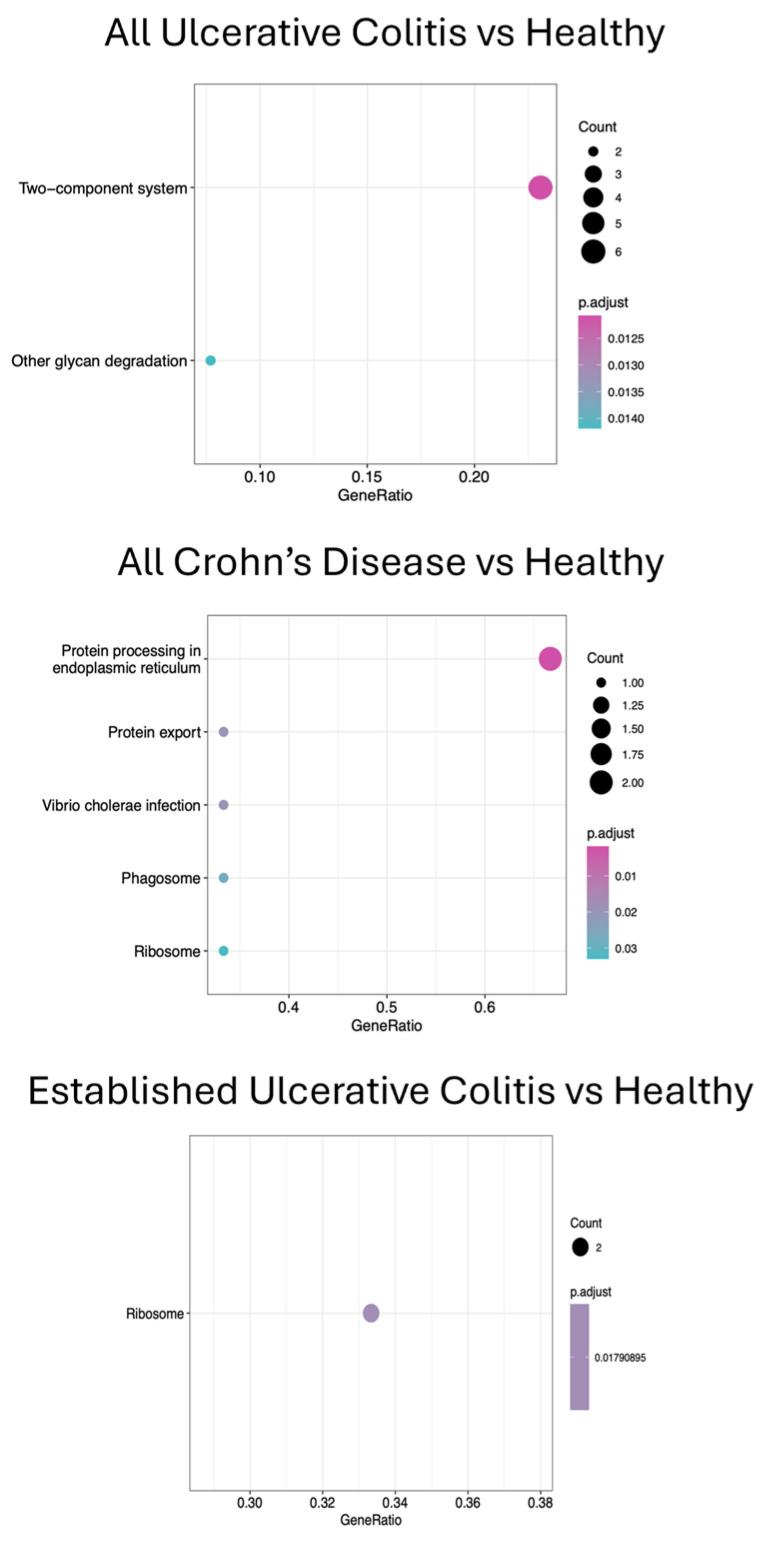
Protein synthesis pathways—RNA polymerase, protein processing, and ribosomes—and sugar metabolism pathway—glycan degradation— appear enriched in diseased groups compared to healthy controls according to the Dotplots for pairwise group comparisons with Kegg functional pathways. The X-axis shows the ratio of differentially expressed genes that are annotated with each KEGG term (different GeneRatio values do not add up to 1.0 because genes are often annotated with multiple KEGG terms). The color of the dot represents the adjusted *p*-value with the color scale in the legends for each plot. The size of the dot represents the number of differentially expressed genes associated with that term.

Metatranscriptomics analysis provides a link between the genetic potential of the microbiome and its molecular activity [53]. It offers insights into the collective gene expression of the microbial community by analyzing all bacterial RNA transcripts (mRNA, rRNA, tRNA) and revealing actively transcribed genes after sequencing and analysis [49]. These tools are still relatively new and have only had limited use in human health in general [53] and, to the best of our knowledge, in PIBD research in particular. We used metatranscriptomic analyses to assess gene expression across all KEGG pathways (Figure 4) and identified several that were upregulated in UC and/or CD compared to healthy controls. These included the protein synthesis pathways (enriched in CD and UC)—RNA polymerase, protein processing, and ribosomes—as well as sugar metabolism pathway (enriched in UC) -glycan degradation. As the use of these tools expands, integrating metatranscriptomic and metagenomic approaches will likely be essential for gaining a more comprehensive understanding of the microbiome’s role in IBD [49,53,54].

### 3.4. Metabolomics Analysis

**Figure 5:**
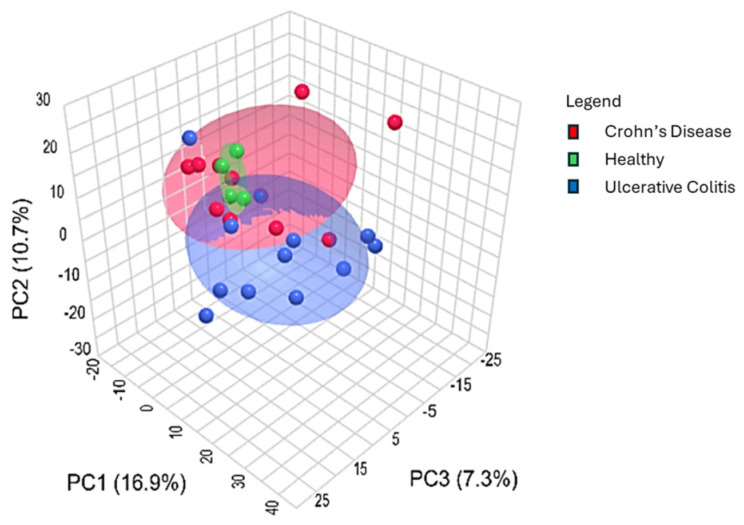
The metabolomes of healthy and PIBD samples tended to group together when visualized via Principal Component Analysis (PCA). CD patients appear to cluster closer to healthy controls than UC patients as demonstrated by the overlapping circles. Groups were analyzed using PERMANOVA, and distributions were computed by using the Euclidean distance based on the PCs in MetaboAnalyst 6.0 software. Respective group colorings denote 95% confidence intervals.

Metabolomic analyses have emerged as a powerful tool to identify potential metabolic biomarkers for physiological and pathological conditions [55]. While the application of these approaches to PIBD has been limited and there appears to be a lack of consensus due to different methods and samples [55,56], a few studies have evaluated metabolites from breath, serum, fecal, and urine samples of PIBD patients [57,58,59,60,61,62]. Similarly to Kolho et al., we utilized an untargeted metabolomic approach to determine potential differences in the metabolites of PIBD patients and healthy controls. PCA analysis (Figure 5) revealed a separation between healthy and PIBD samples, with distinct clustering patterns. Notably, CD patients tended to group more closely with healthy controls than with UC patients, as indicated by the overlapping circles in the PCA plot, suggesting greater similarity between these two groups. This same trend was also observed in our microbiome population composition (Figure 1B,C).

**Figure 6:**
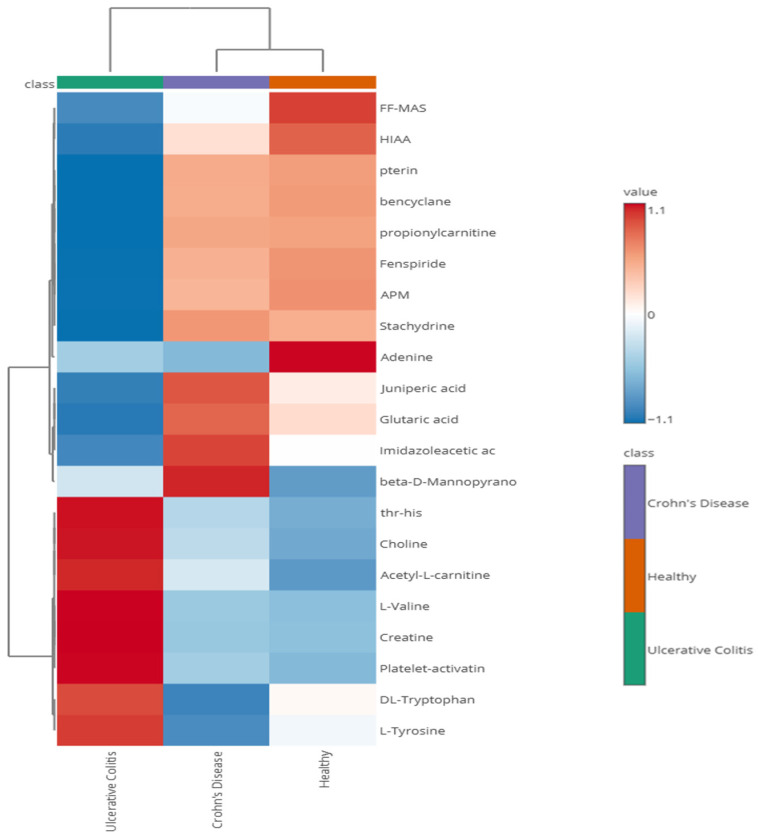
The fecal metabolomes of UC patients exhibit distinct clustering patterns from CD and healthy controls in a Pearson Ward heatmap of metabolite abundance across consolidated diagnoses. Rows represent individual metabolites while columns correspond to consolidated diagnoses. The color scale indicates relative abundance with red representing higher concentrations of a given metabolite while blue indicates lower concentrations.

**Figure 7:**
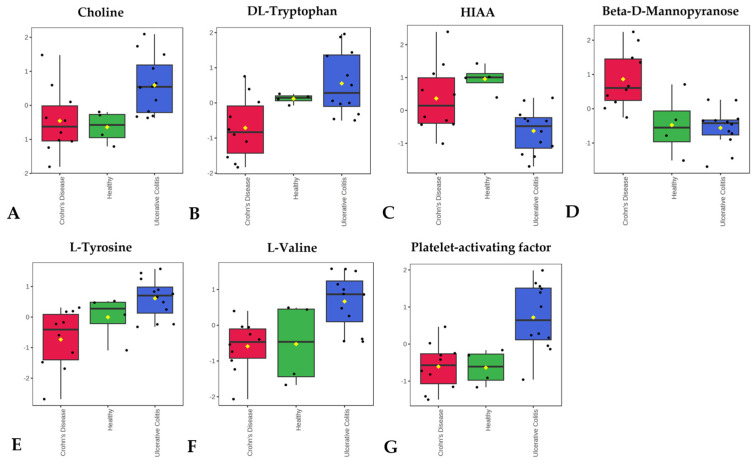
Abundance of several metabolites varies according to disease state: (**A**) choline, (**B**) DL-tryptophan, (**C**) HIAA, (**D**) Beta-D-Mannopyranose (**E**) L-tyrosine, (**F**) L-valine (**G**) Platelet-activating factor. Boxplots of 6 metabolites show significant differences between the consolidated diagnoses and healthy controls.

**Figure 8:**
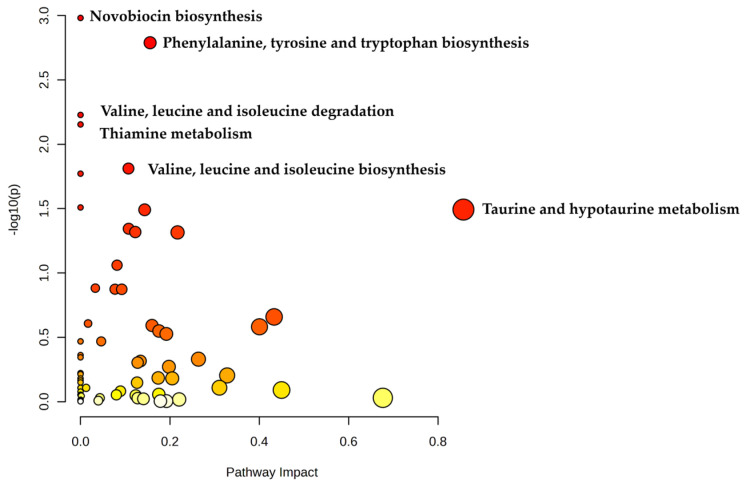
Pathway enrichment analysis of Ulcerative Colitis and Crohn’s Disease samples show significant differences in vital microbial metabolic pathways. Data are plotted as −log10(p) versus pathway impact and darker color represent greater significant differences. Larger circle sizes represent a greater pathway impact and darker shades of red represent greater significance.

Heatmap analysis (Figure 6) also revealed this same tendency of UC samples to separate from the CD and healthy control samples. The heatmap and the associated box plots additionally illustrate several metabolites that appear differentially abundant between PIBD patients and healthy controls (with significant *p*-values, Supplementary Table S10). While FDR q-values did not indicate significant differences, these findings may offer promising leads for future biomarker exploration with larger cohorts. Interestingly, Kolho et al. also discovered different levels of metabolites in UC compared to CD and elevated levels of tryptophan and choline in UC samples corresponding to our findings [62]. For example, choline metabolism (Figure 7) has been linked to IBD with studies suggesting that alterations in choline availability affect gut microbiota composition and function. In IBD mouse models, disruptions in choline metabolism have been associated with increased inflammation and even mood disorders, highlighting its role in both gut and neurological health [63,64,65]. Also, valine and tyrosine (Figure 7) have been reported to be elevated in UC [62] or in both UC and CD [66]. Additionally, tryptophan metabolism, including 5-HIAA (Figure 7) is closely tied to IBD, as the gut microbiome can regulate tryptophan conversion through mechanisms such as the kynurenine pathway, which influences immune responses and intestinal inflammation. Since tryptophan is also a precursor for serotonin, an essential neurotransmitter in gut motility and secretion, disruptions in this pathway may impact serotonin synthesis and contribute to gut dysfunction commonly observed in IBD patients [67,68,69]. Interestingly, despite elevated tryptophan levels in UC samples, we observed reduced levels of 5-hydroxyindoleacetic acid (5HIAA), the principal metabolite of serotonin degradation (Figure 7). This apparent disconnect may suggest a regulatory block in serotonin biosynthesis. A key rate-limiting enzyme in the serotonin pathway is tryptophan hydroxylase-1 (TPH1), which catalyzes the conversion of tryptophan to 5-hydroxytryptophan. Notably, TPH1 expression is positively regulated by vitamin D. Dussik et al. [70] demonstrated that 1,25-dihydroxyvitamin D increases TPH1 mRNA expression in colonic tissue, and subsequent work by Grozić et al. [71] confirmed that vitamin D enhances TPH1 activity and serotonin pathway activation in human colonic epithelial models. Given the reported prevalence of vitamin D deficiency in UC patients, reduced TPH1 expression may limit serotonin synthesis and 5HIAA formation [72], despite an abundance of upstream tryptophan, as suggested in Figure 7. Additionally, platelet activating factor has been linked to the pathophysiology of IBD [73,74] and was discovered elevated in UC patients by Oshimoto et al. [75]. Mannose sugars, on the other hand, have been found to inhibit inflammation and protect the intestinal barrier in UC and colitis mouse models [76,77]. Additionally, a pathway enrichment analysis of UC and CD samples was conducted (Figure 8) and found significant differences in phenylalanine, tyrosine, and tryptophan biosynthesis (*p* = 0.002, Impact = 0.156) and valine, leucine, and isoleucine biosynthesis (*p* = 0.015, Impact = 0.107).

## 4. Discussion

As our results indicate, 16S rRNA sequencing suggested that disease state did not determine overall microbiome community even though a few taxa appear to be enriched or depleted in response to disease or because of it. The evaluation of the genomic potential of the microbiome, however, revealed pathways that were differentially enriched in UC and CD. Additionally, select KEGG functional pathways appeared to be enriched in transcriptome in PIBD patients, particularly in UC. Furthermore, the metabolomic analyses uncovered metabolites that were differentially abundant between the two study populations. A pathway analysis of UC and CD samples also revealed significant alterations to branched chain and aromatic amino acid, thiamine, and taurine in vital microbial meta-bolic pathways. Importantly, some of these metabolites are critical to host-microbe interactions, immune regulation, and/or epithelial barrier function, and their differential abundance may contribute to the pathophysiology of IBD. Thus, our findings highlight the need for expanded use of multiomic approaches, which may offer greater potential for identifying diagnostic biomarkers and therapeutic targets for PIBD compared to traditional microbiome community composition analyses.

Previous studies have already successfully utilized the combination of these techniques to explore aspects of this multifactorial disease. Zhang et al., for example, used metatranscriptomic, -genomic and -proteomic approaches to evaluate the activity of several microbial proteins, as well as their roles in cell to cell and microbiome to host interactions in IBD [78]. Additionally, a combination of metagenomics, metaviromics, and metatranscriptomics approaches on community and contig level validated and contextualized the human gut virome better than metagenome and -virome studies alone in a small cohort of pediatric ulcerative colitis patients [79]. The effectiveness of multiomic approaches was further demonstrated in studies of mucosal inflammation and healing in a small pediatric Crohn’s Disease cohort. While metagenomic analysis alone revealed no significant differences, integrating multiomic tools allowed for a more detailed characterization of microbial and metabolomic features, providing deeper insights into disease mechanisms [80]. As the cost of multiomic techniques decreases and advancements improve accessibility, throughput, profiling depth, data integration, storage, and analysis, these tools may provide deeper insights into the complexity of PIBD and facilitate the discovery of new therapeutic and diagnostic options [81,82,83].

While multiomic-based approaches show greater potential in our study for identifying putative PIBD biomarkers compared to microbiome community composition data alone, our study presents certain limitations and, therefore, future analyses and perspectives are needed. In particular, to enhance statistical power in our study and better differentiate between healthy and PIBD states, an increased number of study participants is needed. Additionally, subdividing patient cohorts may help minimize batch effects and improve analysis accuracy. Leveraging machine learning and AI may also enhance predictive modeling for PIBD diagnosis and biomarker discovery [84]. Additionally, standardized sample acquisition, preparation, and analyses may be needed because discrepancies in reported results may be due, at least in part, to heterogeneous methodologies, type of samples, sample handling, and microbiome assessment, as Zhuang et al. note [40]. Multiomic tools might also broaden the understanding of PIBD pathophysiology, since 16S rRNA amplicon sequencing has been criticized with its limited ability to accurately identify certain microbial species and provide more narrow taxonomic classification of the microorganisms present [6,50,85]. Additionally, fecal samples are generally more diluted than intestinal tissue samples, which may limit their accuracy in reflecting the microbiome’s composition and functional activity [85]. Diarrhea, associated with PIBD, can also decrease the microbe population in the sample [43]. Inflammation in very early onset IBD patients, microbial maturity in all groups, and age should also be taken into consideration (microbiome development) when investigating PIBD [86]. Integrating metaproteomic tools could further enhance our understanding of PIBD by providing insights into microbial protein expression and function [87]. In particular, these approaches hold promise for improving non-invasive diagnostic biomarkers and tailoring microbiome-targeted interventions, such as “precision probiotics” or dietary modifications, to support personalized treatment strategies in clinical practice.

## 5. Conclusions

This study underscores the importance of integrating multiomic and bioinformatic tools to enhance our understanding of the gut microbiome, its interactions within the intestinal microenvironment, and its potential role in PIBD pathogenesis. Our findings suggest that relying solely on 16S rRNA sequencing may be insufficient to capture the complexity of PIBD-associated microbial changes. Instead, multiomic approaches, including metagenomics, metatranscriptomics, and metabolomics, may provide a more comprehensive view of microbial function and activity, and offer a greater potential for identifying disease biomarkers. Expanding the use of these advanced techniques could lead to improved diagnostic and therapeutic strategies for PIBD.

This article is a revised and expanded version of a poster entitled “A Multiomics-based Analysis of the Fecal Microbiome of Pediatric inflammatory Bowel Disease (IBD) Patients Reveals Potential Disease-specific Features More Effectively than Microbiome Community Composition Sequence Data”, which was presented at the Crohn’s & Colitis Congress, San Francisco, on 6–8 February 2025 [88].

## Figures and Tables

**Table 1:** Demographics of recruited patients.

	Individuals	Sex: F, M (% F)	Average Age (Range)	Race: White, Hispanic, Asian (% White)
Diagnosis				
Crohn’s Disease—Naive	2	1, 1 (50%)	17	2, 0, 0 (100%)
Crohn’s Disease—Established	8	5, 3 (62.5%)	13.25 (7–17)	8, 0, 0 (100%)
Ulcerative Colitis—Naive	7	4, 3 (57.1%)	13.43 (8–16)	6, 1, 0 (85.7%)
Ulcerative Colitis—Established	5	3, 2 (60%)	12.4 (6–18)	3, 1, 1 (60%)
Healthy	4	3, 1 (75%)	11.25 (6–14)	3, 1, 0 (75%)
Disease Group				
All Crohn’s Disease	10	6, 4 (60%)	14 (7–17)	10, 0, 0 (100%)
All Ulcerative Colitis	12	7, 5 (58.3%)	13 (6–18)	9, 2, 1 (75%)
All Healthy	4	3, 1 (75%)	9.75 (5–17)	3, 1, 0 (75%)
All Samples	26	16, 10 (61.5%)	13 (5–18)	22, 3, 1 (84.6%)

## Data Availability

The scripts used in this study are openly available in GitHub at https://github.com/ASU-Bioinformatics/project-scripts/tree/main/Project_Scripts/Sandrin_6209010; Scripts were added to this site on 19 March 2025; The data presented in this study are openly available in the National Library of Medicine at https://dataview.ncbi.nlm.nih.gov/object/PRJNA1241499?reviewer=c1co73pf9rolu6aepfan9701n6 (accessed on 19 March 2025) reference number PRJNA1241499.

## Supplementary Materials

The following supporting information can be downloaded at: https://www.mdpi.com/article/10.3390/biom15050746/s1, Table S1: 16S rRNA Sequencing Counts; Table S2: Metagenomics Sequencing Counts; Table S3: Metatranscriptomics Sequencing Counts; Table S4: ANCOM-BC Results for Crohn’s Disease; Table S5: ANCOM-BC Results for Ulcerative Colitis; Table S6: Differential Abundance of Taxa; Table S7: Differential Prevalence of Taxa; Table S8: Differential Abundance of Functional Pathways; Table S9: Differential Prevalence of Functional Pathways; Table S10: Metabolite analysis with ANOVA and Tukey’s HSD comparison

## Abbreviations

The following abbreviations are used in this manuscript:

PIBD	Pediatric Inflammatory Bowel Disease
IBD	Inflammatory Bowel Disease
GI	Gastrointestinal
CD	Crohn’s Disease
UC	Ulcerative Colitis
