# Supplementary material for: Multiomics-Based Profiling of the Fecal Microbiome Reveals Potential Disease-Specific Signatures in Pediatric IBD (PIBD)
Source: Biomolecules. 2025 May 21;15(5):746. doi: 10.3390/biom15050746 (PMC12109367; doi:10.3390/biom15050746)
Supplement: Supplementary file 1 [file biomolecules-15-00746-s001.zip › supplemental1-dada2-stats.pdf]

Supplemental Table S1

Read depth, quality filtering, and denoising statistics from DADA2 for 16S samples.

SampleID	Input Reads	Filtered Reads	% Input Passed Filter	Denoised	Non-Chimeric	% Input Non-Chimeric
MB_001	31615	30232	95.63	29958	29310	92.71
MB_003	36682	34630	94.41	33816	32807	89.44
MB_004	14424	13605	94.32	13454	12954	89.81
MB_005	45054	43391	96.31	42960	41621	92.38
MB_006	30815	29248	94.91	29013	28268	91.73
MB_011	28596	27159	94.97	26910	26400	92.32
MB_012	13874	13340	96.15	13212	12879	92.83
MB_014	22949	21950	95.65	21737	21283	92.74
MB_016	15069	14386	95.47	14195	13874	92.07
MB_017	36669	34888	95.14	34499	33609	91.66
MB_018	16445	15601	94.87	15349	15239	92.67
MB_020	12795	12218	95.49	12095	11885	92.89
MB_021	22875	21352	93.34	21078	20774	90.82
MB_023	23338	22434	96.13	22328	22106	94.72
MB_024	28893	27166	94.02	26938	26786	92.71
MB_025	23067	22071	95.68	21846	21668	93.94
MB_028	21692	20826	96.01	20651	20430	94.18
MB_033	17864	16986	95.09	16839	16703	93.5
MB_035	15213	14389	94.58	14249	14206	93.38
MB_037	13371	12482	93.35	12323	12145	90.83
MB_038	11539	10941	94.82	10784	10759	93.24
MB_039	15555	14870	95.6	14759	14447	92.88
MB_040	14221	13571	95.43	13403	13015	91.52
MB_044	26751	25165	94.07	24928	24614	92.01
MB_045	22945	21759	94.83	21535	21384	93.2
MB_047	19294	18222	94.44	18016	17874	92.64
MB_049	15672	14971	95.53	14857	14723	93.94
MB_050	12083	11453	94.79	11281	10949	90.61
MB_053	24824	23682	95.4	23469	23425	94.36
MB_055	19339	18139	93.79	18008	17794	92.01
