# Supplementary material for: Multiomics-Based Profiling of the Fecal Microbiome Reveals Potential Disease-Specific Signatures in Pediatric IBD (PIBD)
Source: Biomolecules. 2025 May 21;15(5):746. doi: 10.3390/biom15050746 (PMC12109367; doi:10.3390/biom15050746)
Supplement: Supplementary file 1 [file biomolecules-15-00746-s001.zip › supplemental10-metabolites-ANOVA posthoc.pdf]

Supplemental Table S10

Metabolite analysis with ANOVA and Tukey’s HSD comparison

Metabolite	p.value	Tukey's HSD
beta-D-Mannopyranose	0.00050155	Healthy-Crohn's Disease; Ulcerative Colitis-Crohn's Disease
Platelet-activating factor	0.00081318	Ulcerative Colitis-Crohn's Disease; Ulcerative Colitis-Healthy
L-Valine	0.0029709	Ulcerative Colitis-Crohn's Disease; Ulcerative Colitis-Healthy
L-Tyrosine	0.0031045	Ulcerative Colitis-Crohn's Disease
HIAA	0.0035998	Ulcerative Colitis-Crohn's Disease; Ulcerative Colitis-Healthy
DL-Tryptophan	0.0068022	Ulcerative Colitis-Crohn's Disease
Choline	0.012573	Ulcerative Colitis-Crohn's Disease

In Tukey's HSD pair-wise comparison, group comparison listed first has the greater abundance.
