# Supplementary material for: Multiomics-Based Profiling of the Fecal Microbiome Reveals Potential Disease-Specific Signatures in Pediatric IBD (PIBD)
Source: Biomolecules. 2025 May 21;15(5):746. doi: 10.3390/biom15050746 (PMC12109367; doi:10.3390/biom15050746)
Supplement: Supplementary file 1 [file biomolecules-15-00746-s001.zip › supplemental2-metagenomics-reads.pdf]

Supplemental Table S2

Read depth retained through quality filtering and removal of host contamination, for whole genome metagenomics samples.

Sample	Raw Reads	Filtered Reads	Decontaminated Reads	Percent Retained
MB-001	21,324,461	19,002,163	15,872,689	74.43%
MB-003	23,841,606	20,363,496	2,382,061	9.99%
MB-004	24,881,627	22,103,101	22,091,696	88.79%
MB-005	22,139,938	19,606,924	19,603,171	88.54%
MB-006	24,395,152	20,965,036	20,952,993	85.89%
MB-011	22,103,047	19,020,010	2,471,846	11.18%
MB-012	25,717,530	21,743,679	21,739,098	84.53%
MB-014	22,295,269	18,741,482	18,710,073	83.92%
MB-016	25,251,042	21,121,271	5,903,460	23.38%
MB-017	23,865,823	20,537,848	20,504,206	85.91%
MB-018	28,604,464	15,366,085	12,430,116	43.46%
MB-020	16,469,701	14,591,913	14,584,316	88.55%
MB-021	20,590,941	17,537,653	17,528,075	85.13%
MB-023	17,943,517	15,564,877	11,231,554	62.59%
MB-024	24,193,283	21,135,351	21,121,850	87.30%
MB-025	27,662,790	23,832,734	23,821,831	86.12%
MB-028	27,090,886	22,390,292	8,999,393	33.22%
MB-033	22,885,383	19,862,234	19,702,416	86.09%
MB-035	22,930,949	19,437,972	19,402,527	84.61%
MB-037	22,046,744	18,472,024	18,466,329	83.76%
MB-038	31,000,238	25,156,197	16,038,664	51.74%
MB-039	42,616,872	36,095,570	27,330,470	64.13%
MB-040	21,607,704	18,785,883	18,782,218	86.92%
MB-044	22,964,367	19,241,895	19,228,853	83.73%
MB-045	25,247,772	22,534,172	22,531,303	89.24%
MB-047	27,612,265	24,190,426	24,175,642	87.55%
MB-049	28,944,904	24,272,083	24,114,649	83.31%
MB-050	18,700,839	16,527,727	16,520,559	88.34%
MB-053	18,904,343	16,371,025	16,363,336	86.56%
MB-055	39,026,123	32,034,408	30,250,935	77.51%
