# Supplementary material for: Multiomics-Based Profiling of the Fecal Microbiome Reveals Potential Disease-Specific Signatures in Pediatric IBD (PIBD)
Source: Biomolecules. 2025 May 21;15(5):746. doi: 10.3390/biom15050746 (PMC12109367; doi:10.3390/biom15050746)
Supplement: Supplementary file 1 [file biomolecules-15-00746-s001.zip › supplemental3-metatranscriptomics-reads.pdf]

Supplemental Table S3
Read depth retained through quality filtering and removal of host contamination, for whole transcriptome metatranscriptomics samples.

Sample	Raw Reads	Filtered Reads	Decontaminated Reads	Percent Retained
MB-001	38,166,790	17,628,268	17,490,235	45.83%
MB-003	36,913,554	11,172,292	6,420,905	17.39%
MB-004	40,213,719	12,265,533	12,248,981	30.46%
MB-005	31,024,403	12,185,225	12,178,538	39.25%
MB-006	29,405,829	14,864,990	14,849,049	50.50%
MB-011	24,982,386	12,475,258	11,030,877	44.15%
MB-012	34,272,692	15,948,861	15,945,838	46.53%
MB-014	34,404,409	22,444,918	22,426,801	65.19%
MB-016	40,059,658	20,888,266	18,028,822	45.00%
MB-017	30,816,093	12,657,138	12,630,098	40.99%
MB-018	33,997,130	14,069,434	12,893,653	37.93%
MB-020	28,744,529	13,434,551	13,422,337	46.70%
MB-021	26,219,166	16,482,913	16,458,903	62.77%
MB-023	20,710,290	13,058,249	12,994,897	62.75%
MB-024	27,538,634	14,033,384	14,025,991	50.93%
MB-025	29,915,690	20,250,757	20,247,757	67.68%
MB-028	37,749,691	23,787,723	22,832,845	60.48%
MB-033	35,678,398	14,588,451	14,582,101	40.87%
MB-035	44,078,858	16,472,096	16,470,115	37.37%
MB-037	31,419,381	14,728,176	14,724,239	46.86%
MB-038	40,967,443	20,819,030	18,774,320	45.83%
MB-039	20,439,657	13,651,699	13,570,856	66.39%
MB-040	47,322,223	20,745,316	20,738,274	43.82%
MB-044	47,453,859	22,137,780	22,131,129	46.64%
MB-045	38,639,193	24,748,160	24,743,185	64.04%
MB-047	36,359,827	20,708,606	20,694,390	56.92%
MB-049	29,596,031	14,885,141	14,870,616	50.25%
MB-050	22,102,256	13,044,908	13,031,514	58.96%
MB-053	21,569,855	14,340,033	14,335,172	66.46%
MB-055	26,192,022	18,539,302	18,457,941	70.47%
