# Supplementary material for: Multiomics-Based Profiling of the Fecal Microbiome Reveals Potential Disease-Specific Signatures in Pediatric IBD (PIBD)
Source: Biomolecules. 2025 May 21;15(5):746. doi: 10.3390/biom15050746 (PMC12109367; doi:10.3390/biom15050746)
Supplement: Supplementary file 1 [file biomolecules-15-00746-s001.zip › supplemental4-ancombc-7-crohns.pdf]

Supplemental Table S4

ANCOM-BC results for all detected taxa in 16S samples, comparing Crohn's with healthy samples.

Taxa ID	Log Fold Change	P-Value	Q-Value	Standard Error	W Score
k__Bacteria;p__Firmicutes;c__Erysipelotrichi;o__Erysipelotrichales; f__Erysipelotrichaceae;g__Holdemania;s__	0.703	0.00417	0.538	0.245	2.87
k__Bacteria;__;__;__;__;__;__	0.437	0.276	1	0.401	1.09
k__Bacteria;p__Actinobacteria;c__Actinobacteria;o__Actinomycetales; f__Actinomycetaceae;g__Actinomyces;s__	-0.54	0.202	1	0.423	-1.28
k__Bacteria;p__Actinobacteria;c__Actinobacteria;o__Actinomycetales; f__Corynebacteriaceae;g__Corynebacterium;s__durum	-0.213	0.25	1	0.185	-1.15
k__Bacteria;p__Actinobacteria;c__Actinobacteria;o__Actinomycetales; f__Micrococcaceae;g__Rothia;s__dentocariosa	-0.0516	0.831	1	0.242	-0.214
k__Bacteria;p__Actinobacteria;c__Actinobacteria;o__Actinomycetales; f__Micrococcaceae;g__Rothia;s__mucilaginosa	0.223	0.493	1	0.326	0.685
k__Bacteria;p__Actinobacteria;c__Actinobacteria;o__Bifidobacteriales; f__Bifidobacteriaceae;g__Bifidobacterium;__	-0.812	0.114	1	0.514	-1.58
k__Bacteria;p__Actinobacteria;c__Actinobacteria;o__Bifidobacteriales; f__Bifidobacteriaceae;g__Bifidobacterium;s__	-2.68	0.107	1	1.66	-1.61
k__Bacteria;p__Actinobacteria;c__Actinobacteria;o__Bifidobacteriales; f__Bifidobacteriaceae;g__Bifidobacterium;s__adolescentis	-1.05	0.22	1	0.852	-1.23
k__Bacteria;p__Actinobacteria;c__Actinobacteria;o__Bifidobacteriales; f__Bifidobacteriaceae;g__Bifidobacterium;s__bifidum	-1.38	0.357	1	1.5	-0.921
k__Bacteria;p__Actinobacteria;c__Coriobacteriia;o__Coriobacteriales; f__Coriobacteriaceae;g__;s__	0.275	0.742	1	0.836	0.329
k__Bacteria;p__Actinobacteria;c__Coriobacteriia;o__Coriobacteriales; f__Coriobacteriaceae;g__Adlercreutzia;s__	0.705	0.178	1	0.524	1.35
k__Bacteria;p__Actinobacteria;c__Coriobacteriia;o__Coriobacteriales; f__Coriobacteriaceae;g__Atopobium;s__	-0.074	0.762	1	0.244	-0.303
k__Bacteria;p__Actinobacteria;c__Coriobacteriia;o__Coriobacteriales; f__Coriobacteriaceae;g__Collinsella;s__aerofaciens	0.146	0.909	1	1.29	0.114
k__Bacteria;p__Actinobacteria;c__Coriobacteriia;o__Coriobacteriales; f__Coriobacteriaceae;g__Collinsella;s__stercoris	0.00713	0.979	1	0.27	0.0264
k__Bacteria;p__Actinobacteria;c__Coriobacteriia;o__Coriobacteriales; f__Coriobacteriaceae;g__Eggerthella;s__lenta	-0.29	0.758	1	0.941	-0.308
k__Bacteria;p__Actinobacteria;c__Coriobacteriia;o__Coriobacteriales; f__Coriobacteriaceae;g__Slackia;s__	-1.06	0.0546	1	0.553	-1.92

k__Bacteria;p__Bacteroidetes;c__Bacteroidia;o__Bacteroidales; f__[Barnesiellaceae];g__;s__	0.681	0.451	1	0.904	0.753
k__Bacteria;p__Bacteroidetes;c__Bacteroidia;o__Bacteroidales; f__[Odoribacteraceae];g__Butyricimonas;s__	0.246	0.415	1	0.302	0.815
k__Bacteria;p__Bacteroidetes;c__Bacteroidia;o__Bacteroidales; f__[Odoribacteraceae];g__Odoribacter;s__	1.22	0.0136	1	0.496	2.47
k__Bacteria;p__Bacteroidetes;c__Bacteroidia;o__Bacteroidales; f__[Paraprevotellaceae];g__Paraprevotella;s__	-0.481	0.457	1	0.646	-0.744
k__Bacteria;p__Bacteroidetes;c__Bacteroidia;o__Bacteroidales; f__Bacteroidaceae;g__Bacteroides;__	0.803	0.547	1	1.33	0.602
k__Bacteria;p__Bacteroidetes;c__Bacteroidia;o__Bacteroidales; f__Bacteroidaceae;g__Bacteroides;s__	-0.295	0.676	1	0.706	-0.418
k__Bacteria;p__Bacteroidetes;c__Bacteroidia;o__Bacteroidales; f__Bacteroidaceae;g__Bacteroides;s__caccae	-0.229	0.869	1	1.39	-0.165
k__Bacteria;p__Bacteroidetes;c__Bacteroidia;o__Bacteroidales; f__Bacteroidaceae;g__Bacteroides;s__fragilis	-0.614	0.69	1	1.54	-0.399
k__Bacteria;p__Bacteroidetes;c__Bacteroidia;o__Bacteroidales; f__Bacteroidaceae;g__Bacteroides;s__ovatus	-0.122	0.926	1	1.31	-0.0928
k__Bacteria;p__Bacteroidetes;c__Bacteroidia;o__Bacteroidales; f__Bacteroidaceae;g__Bacteroides;s__uniformis	-1.14	0.136	1	0.767	-1.49
k__Bacteria;p__Bacteroidetes;c__Bacteroidia;o__Bacteroidales; f__Porphyromonadaceae;g__Parabacteroides;s__	0.178	0.846	1	0.913	0.195
k__Bacteria;p__Bacteroidetes;c__Bacteroidia;o__Bacteroidales; f__Porphyromonadaceae;g__Parabacteroides;s__distasonis	-0.844	0.525	1	1.33	-0.636
k__Bacteria;p__Bacteroidetes;c__Bacteroidia;o__Bacteroidales; f__Prevotellaceae;g__Prevotella;s__	-1.04	0.138	1	0.7	-1.48
k__Bacteria;p__Bacteroidetes;c__Bacteroidia;o__Bacteroidales; f__Prevotellaceae;g__Prevotella;s__copri	-0.437	0.757	1	1.41	-0.31
k__Bacteria;p__Bacteroidetes;c__Bacteroidia;o__Bacteroidales; f__Rikenellaceae;__;__	-0.291	0.698	1	0.749	-0.389
k__Bacteria;p__Bacteroidetes;c__Bacteroidia;o__Bacteroidales; f__Rikenellaceae;g__;s__	0.476	0.526	1	0.751	0.634
k__Bacteria;p__Bacteroidetes;c__Bacteroidia;o__Bacteroidales; f__Rikenellaceae;g__Alistipes;s__finegoldii	-0.95	0.24	1	0.808	-1.18
k__Bacteria;p__Bacteroidetes;c__Bacteroidia;o__Bacteroidales; f__Rikenellaceae;g__Alistipes;s__indistinctus	-0.896	0.0911	1	0.53	-1.69
k__Bacteria;p__Bacteroidetes;c__Bacteroidia;o__Bacteroidales; f__Rikenellaceae;g__Alistipes;s__onderdonkii	-0.123	0.901	1	0.992	-0.124

k__Bacteria;p__Bacteroidetes;c__Bacteroidia;o__Bacteroidales; f__Rikenellaceae;g__Alistipes;s__putredinis	1.28	0.27	1	1.16	1.1
k__Bacteria;p__Cyanobacteria;c__Chloroplast;o__Stramenopiles; f__;g__;s__	0.134	0.663	1	0.307	0.436
k__Bacteria;p__Firmicutes;c__Bacilli;o__Gemellales; f__Gemellaceae;__;__	1.05	0.189	1	0.801	1.31
k__Bacteria;p__Firmicutes;c__Bacilli;o__Lactobacillales; f__Carnobacteriaceae;__;__	-0.74	0.322	1	0.748	-0.99
k__Bacteria;p__Firmicutes;c__Bacilli;o__Lactobacillales; f__Enterococcaceae;g__Enterococcus;__	0.41	0.572	1	0.725	0.565
k__Bacteria;p__Firmicutes;c__Bacilli;o__Lactobacillales; f__Lactobacillaceae;g__Lactobacillus;s__	0.535	0.293	1	0.509	1.05
k__Bacteria;p__Firmicutes;c__Bacilli;o__Lactobacillales; f__Lactobacillaceae;g__Lactobacillus;s__zeae	0.256	0.54	1	0.418	0.612
k__Bacteria;p__Firmicutes;c__Bacilli;o__Lactobacillales; f__Streptococcaceae;g__Lactococcus;s__	0.226	0.757	1	0.729	0.309
k__Bacteria;p__Firmicutes;c__Bacilli;o__Lactobacillales; f__Streptococcaceae;g__Streptococcus;s__	-1.14	0.248	1	0.987	-1.16
k__Bacteria;p__Firmicutes;c__Bacilli;o__Lactobacillales; f__Streptococcaceae;g__Streptococcus;s__anginosus	-0.213	0.25	1	0.185	-1.15
k__Bacteria;p__Firmicutes;c__Bacilli;o__Lactobacillales; f__Streptococcaceae;g__Streptococcus;s__infantis	0.69	0.454	1	0.921	0.749
k__Bacteria;p__Firmicutes;c__Bacilli;o__Lactobacillales; f__Streptococcaceae;g__Streptococcus;s__luteciae	0.238	0.585	1	0.437	0.546
k__Bacteria;p__Firmicutes;c__Bacilli;o__Turicibacterales; f__Turicibacteraceae;g__Turicibacter;s__	0.184	0.861	1	1.05	0.175
k__Bacteria;p__Firmicutes;c__Clostridia;o__Clostridiales;__;__;__	-0.27	0.76	1	0.881	-0.306
k__Bacteria;p__Firmicutes;c__Clostridia;o__Clostridiales; f__;g__;s__	0.987	0.323	1	0.998	0.989
k__Bacteria;p__Firmicutes;c__Clostridia;o__Clostridiales; f__[Mogibacteriaceae];g__;s__	0.109	0.86	1	0.618	0.177
k__Bacteria;p__Firmicutes;c__Clostridia;o__Clostridiales; f__[Mogibacteriaceae];g__Mogibacterium;s__	-0.0136	0.979	1	0.527	-0.0259
k__Bacteria;p__Firmicutes;c__Clostridia;o__Clostridiales; f__[Tissierellaceae];g__Anaerococcus;s__	0.986	0.0876	1	0.577	1.71
k__Bacteria;p__Firmicutes;c__Clostridia;o__Clostridiales; f__[Tissierellaceae];g__Finegoldia;s__	-0.615	0.141	1	0.418	-1.47
k__Bacteria;p__Firmicutes;c__Clostridia;o__Clostridiales; f__[Tissierellaceae];g__Parvimonas;s__	0.293	0.616	1	0.585	0.501
k__Bacteria;p__Firmicutes;c__Clostridia;o__Clostridiales; f__[Tissierellaceae];g__Peptoniphilus;s__	-0.155	0.785	1	0.568	-0.273

k__Bacteria;p__Firmicutes;c__Clostridia;o__Clostridiales; f__Christensenellaceae;g__;s__	-0.125	0.863	1	0.729	-0.172
k__Bacteria;p__Firmicutes;c__Clostridia;o__Clostridiales; f__Christensenellaceae;g__Christensenella;s__	0.235	0.332	1	0.243	0.969
k__Bacteria;p__Firmicutes;c__Clostridia;o__Clostridiales; f__Clostridiaceae;g__;s__	1.02	0.361	1	1.12	0.914
k__Bacteria;p__Firmicutes;c__Clostridia;o__Clostridiales; f__Clostridiaceae;g__Clostridium;__	1.02	0.091	1	0.603	1.69
k__Bacteria;p__Firmicutes;c__Clostridia;o__Clostridiales; f__Clostridiaceae;g__Clostridium;s__	0.615	0.544	1	1.01	0.606
k__Bacteria;p__Firmicutes;c__Clostridia;o__Clostridiales; f__Clostridiaceae;g__Clostridium;s__celatum	-0.229	0.827	1	1.05	-0.218
k__Bacteria;p__Firmicutes;c__Clostridia;o__Clostridiales; f__Clostridiaceae;g__Clostridium;s__paraputrificum	0.338	0.608	1	0.659	0.514
k__Bacteria;p__Firmicutes;c__Clostridia;o__Clostridiales; f__Clostridiaceae;g__SMB53;s__	1.22	0.116	1	0.773	1.57
k__Bacteria;p__Firmicutes;c__Clostridia;o__Clostridiales; f__Eubacteriaceae;g__Anaerofustis;s__	0.0633	0.895	1	0.48	0.132
k__Bacteria;p__Firmicutes;c__Clostridia;o__Clostridiales; f__Eubacteriaceae;g__Pseudoramibacter_Eubacterium;s__	-0.396	0.491	1	0.575	-0.688
k__Bacteria;p__Firmicutes;c__Clostridia;o__Clostridiales; f__Lachnospiraceae;__;__	0.643	0.517	1	0.991	0.649
k__Bacteria;p__Firmicutes;c__Clostridia;o__Clostridiales; f__Lachnospiraceae;g__;s__	-0.438	0.558	1	0.748	-0.585
k__Bacteria;p__Firmicutes;c__Clostridia;o__Clostridiales; f__Lachnospiraceae;g__[Ruminococcus];s__	-0.642	0.416	1	0.789	-0.813
k__Bacteria;p__Firmicutes;c__Clostridia;o__Clostridiales; f__Lachnospiraceae;g__[Ruminococcus];s__gnavus	0.188	0.88	1	1.25	0.151
k__Bacteria;p__Firmicutes;c__Clostridia;o__Clostridiales; f__Lachnospiraceae;g__[Ruminococcus];s__torques	0.182	0.88	1	1.21	0.15
k__Bacteria;p__Firmicutes;c__Clostridia;o__Clostridiales; f__Lachnospiraceae;g__Anaerostipes;s__	0.902	0.228	1	0.748	1.21
k__Bacteria;p__Firmicutes;c__Clostridia;o__Clostridiales; f__Lachnospiraceae;g__Blautia;__	-0.0981	0.912	1	0.889	-0.11
k__Bacteria;p__Firmicutes;c__Clostridia;o__Clostridiales; f__Lachnospiraceae;g__Blautia;s__	-0.0683	0.865	1	0.403	-0.17
k__Bacteria;p__Firmicutes;c__Clostridia;o__Clostridiales; f__Lachnospiraceae;g__Blautia;s__obeum	-0.0539	0.969	1	1.38	-0.0391

k__Bacteria;p__Firmicutes;c__Clostridia;o__Clostridiales; f__Lachnospiraceae;g__Blautia;s__producta	1.6	0.103	1	0.983	1.63
k__Bacteria;p__Firmicutes;c__Clostridia;o__Clostridiales; f__Lachnospiraceae;g__Clostridium;__	0.33	0.716	1	0.907	0.364
k__Bacteria;p__Firmicutes;c__Clostridia;o__Clostridiales; f__Lachnospiraceae;g__Clostridium;s__aldenense	0.633	0.393	1	0.741	0.854
k__Bacteria;p__Firmicutes;c__Clostridia;o__Clostridiales; f__Lachnospiraceae;g__Clostridium;s__clostridioforme	-0.248	0.738	1	0.74	-0.335
k__Bacteria;p__Firmicutes;c__Clostridia;o__Clostridiales; f__Lachnospiraceae;g__Clostridium;s__hathewayi	0.749	0.387	1	0.867	0.864
k__Bacteria;p__Firmicutes;c__Clostridia;o__Clostridiales; f__Lachnospiraceae;g__Clostridium;s__symbiosum	0.357	0.668	1	0.833	0.429
k__Bacteria;p__Firmicutes;c__Clostridia;o__Clostridiales; f__Lachnospiraceae;g__Coprococcus;s__	0.25	0.538	1	0.407	0.615
k__Bacteria;p__Firmicutes;c__Clostridia;o__Clostridiales; f__Lachnospiraceae;g__Coprococcus;s__catus	0.839	0.464	1	1.14	0.733
k__Bacteria;p__Firmicutes;c__Clostridia;o__Clostridiales; f__Lachnospiraceae;g__Coprococcus;s__eutactus	-0.19	0.673	1	0.45	-0.422
k__Bacteria;p__Firmicutes;c__Clostridia;o__Clostridiales; f__Lachnospiraceae;g__Dorea;s__	2.43	0.0914	1	1.44	1.69
k__Bacteria;p__Firmicutes;c__Clostridia;o__Clostridiales; f__Lachnospiraceae;g__Dorea;s__formicigenerans	1.6	0.181	1	1.2	1.34
k__Bacteria;p__Firmicutes;c__Clostridia;o__Clostridiales; f__Lachnospiraceae;g__Dorea;s__longicatena	-0.167	0.829	1	0.773	-0.216
k__Bacteria;p__Firmicutes;c__Clostridia;o__Clostridiales; f__Lachnospiraceae;g__Lachnospira;s__	1.08	0.298	1	1.04	1.04
k__Bacteria;p__Firmicutes;c__Clostridia;o__Clostridiales; f__Lachnospiraceae;g__Roseburia;s__	0.504	0.699	1	1.3	0.386
k__Bacteria;p__Firmicutes;c__Clostridia;o__Clostridiales; f__Lachnospiraceae;g__Roseburia;s__faecis	0.0633	0.967	1	1.54	0.041
k__Bacteria;p__Firmicutes;c__Clostridia;o__Clostridiales; f__Lachnospiraceae;g__Roseburia;s__inulinivorans	-0.499	0.671	1	1.17	-0.425
k__Bacteria;p__Firmicutes;c__Clostridia;o__Clostridiales; f__Lachnospiraceae;g__Ruminococcus;s__lactaris	-0.128	0.933	1	1.53	-0.0836
k__Bacteria;p__Firmicutes;c__Clostridia;o__Clostridiales; f__Peptostreptococcaceae;g__[Clostridium];__	0.276	0.468	1	0.38	0.726
k__Bacteria;p__Firmicutes;c__Clostridia;o__Clostridiales; f__Peptostreptococcaceae;g__Peptostreptococcus;s__	0.409	0.536	1	0.66	0.619

k__Bacteria;p__Firmicutes;c__Clostridia;o__Clostridiales; f__Ruminococcaceae;__;__	-0.21	0.724	1	0.595	-0.353
k__Bacteria;p__Firmicutes;c__Clostridia;o__Clostridiales; f__Ruminococcaceae;g__;s__	0.665	0.57	1	1.17	0.569
k__Bacteria;p__Firmicutes;c__Clostridia;o__Clostridiales; f__Ruminococcaceae;g__Anaerotruncus;s__	-0.255	0.54	1	0.416	-0.613
k__Bacteria;p__Firmicutes;c__Clostridia;o__Clostridiales; f__Ruminococcaceae;g__Butyricicoccus;s__pullicaecorum	1.14	0.167	1	0.826	1.38
k__Bacteria;p__Firmicutes;c__Clostridia;o__Clostridiales; f__Ruminococcaceae;g__Clostridium;s__methylpentosum	0.437	0.124	1	0.284	1.54
k__Bacteria;p__Firmicutes;c__Clostridia;o__Clostridiales; f__Ruminococcaceae;g__Faecalibacterium;s__prausnitzii	-0.222	0.701	1	0.579	-0.384
k__Bacteria;p__Firmicutes;c__Clostridia;o__Clostridiales; f__Ruminococcaceae;g__Gemmiger;s__formicilis	-0.343	0.666	1	0.794	-0.432
k__Bacteria;p__Firmicutes;c__Clostridia;o__Clostridiales; f__Ruminococcaceae;g__Oscillospira;s__	0.316	0.603	1	0.607	0.521
k__Bacteria;p__Firmicutes;c__Clostridia;o__Clostridiales; f__Ruminococcaceae;g__Ruminococcus;s__	-1.82	0.0182	1	0.773	-2.36
k__Bacteria;p__Firmicutes;c__Clostridia;o__Clostridiales; f__Ruminococcaceae;g__Ruminococcus;s__bromii	2.64	0.0883	1	1.55	1.7
k__Bacteria;p__Firmicutes;c__Clostridia;o__Clostridiales; f__Ruminococcaceae;g__Ruminococcus;s__callidus	0.431	0.743	1	1.32	0.328
k__Bacteria;p__Firmicutes;c__Clostridia;o__Clostridiales; f__Ruminococcaceae;g__Ruminococcus;s__flavefaciens	0.388	0.752	1	1.23	0.317
k__Bacteria;p__Firmicutes;c__Clostridia;o__Clostridiales; f__Veillonellaceae;g__Dialister;s__	1.08	0.418	1	1.33	0.811
k__Bacteria;p__Firmicutes;c__Clostridia;o__Clostridiales; f__Veillonellaceae;g__Phascolarctobacterium;s__	0.316	0.653	1	0.703	0.45
k__Bacteria;p__Firmicutes;c__Clostridia;o__Clostridiales; f__Veillonellaceae;g__Veillonella;s__dispar	0.446	0.636	1	0.942	0.474
k__Bacteria;p__Firmicutes;c__Clostridia;o__Clostridiales; f__Veillonellaceae;g__Veillonella;s__parvula	0.538	0.37	1	0.601	0.896
k__Bacteria;p__Firmicutes;c__Erysipelotrichi;o__Erysipelotrichales; f__Erysipelotrichaceae;g__;s__	2.38	0.0167	1	0.993	2.39
k__Bacteria;p__Firmicutes;c__Erysipelotrichi;o__Erysipelotrichales; f__Erysipelotrichaceae;g__[Eubacterium];s__biforme	-1.79	0.264	1	1.6	-1.12
k__Bacteria;p__Firmicutes;c__Erysipelotrichi;o__Erysipelotrichales; f__Erysipelotrichaceae;g__[Eubacterium];s__dolichum	-1.41	0.0105	1	0.551	-2.56

k__Bacteria;p__Firmicutes;c__Erysipelotrichi;o__Erysipelotrichales; f__Erysipelotrichaceae;g__Bulleidia;s__moorei	0.012	0.981	1	0.49	0.0244
k__Bacteria;p__Firmicutes;c__Erysipelotrichi;o__Erysipelotrichales; f__Erysipelotrichaceae;g__Clostridium;s__ramosum	0.377	0.79	1	1.42	0.266
k__Bacteria;p__Firmicutes;c__Erysipelotrichi;o__Erysipelotrichales; f__Erysipelotrichaceae;g__Clostridium;s__saccharogumia	-0.419	0.523	1	0.656	-0.639
k__Bacteria;p__Firmicutes;c__Erysipelotrichi;o__Erysipelotrichales; f__Erysipelotrichaceae;g__Clostridium;s__spiroforme	0.117	0.913	1	1.07	0.109
k__Bacteria;p__Firmicutes;c__Erysipelotrichi;o__Erysipelotrichales; f__Erysipelotrichaceae;g__Coprobacillus;s__	0.468	0.22	1	0.381	1.23
k__Bacteria;p__Fusobacteria;c__Fusobacteriia;o__Fusobacteriales; f__Fusobacteriaceae;g__Fusobacterium;s__	0.166	0.708	1	0.443	0.375
k__Bacteria;p__Proteobacteria;c__Betaproteobacteria;o__Burkholderiales; f__Alcaligenaceae;g__Achromobacter;s__	0.0919	0.809	1	0.379	0.242
k__Bacteria;p__Proteobacteria;c__Betaproteobacteria;o__Burkholderiales; f__Alcaligenaceae;g__Sutterella;s__	-0.0635	0.948	1	0.979	-0.0648
k__Bacteria;p__Proteobacteria;c__Deltaproteobacteria;o__Desulfovibrionales; f__Desulfovibrionaceae;g__Bilophila;s__	0.487	0.425	1	0.61	0.798
k__Bacteria;p__Proteobacteria;c__Gammaproteobacteria;o__Enterobacteriales; f__Enterobacteriaceae;__;__	1.94	0.0249	1	0.866	2.24
k__Bacteria;p__Proteobacteria;c__Gammaproteobacteria;o__Enterobacteriales; f__Enterobacteriaceae;g__Proteus;s__	-0.213	0.25	1	0.185	-1.15
k__Bacteria;p__Proteobacteria;c__Gammaproteobacteria;o__Pasteurellales; f__Pasteurellaceae;g__Aggregatibacter;s__	0.117	0.77	1	0.401	0.292
k__Bacteria;p__Proteobacteria;c__Gammaproteobacteria;o__Pasteurellales; f__Pasteurellaceae;g__Haemophilus;s__parainfluenzae	0.578	0.323	1	0.585	0.988
k__Bacteria;p__Tenericutes;c__Mollicutes;o__RF39; f__;g__;s__	0.408	0.362	1	0.447	0.912
k__Bacteria;p__Verrucomicrobia;c__Verrucomicrobiae;o__Verrucomicrobiales; f__Verrucomicrobiaceae;g__Akkermansia;s__muciniphila	1.02	0.359	1	1.11	0.917
