# Supplementary material for: Multiomics-Based Profiling of the Fecal Microbiome Reveals Potential Disease-Specific Signatures in Pediatric IBD (PIBD)
Source: Biomolecules. 2025 May 21;15(5):746. doi: 10.3390/biom15050746 (PMC12109367; doi:10.3390/biom15050746)
Supplement: Supplementary file 1 [file biomolecules-15-00746-s001.zip › supplemental5-ancombc-7-uc.pdf]

Supplemental Table S5

ANCOM-BC results at species level for all detected taxa in 16S samples, comparing UC samples to healthy controls.

Taxa ID	Log Fold Change	P-Value	Q-Value	Standard Error	W Score
k__Bacteria;p__Firmicutes;c__Clostridia;o__Clostridiales; f__Ruminococcaceae;g__Gemmiger;s__formicilis	-4.27	1.11×10^{-17}	1.44×10^{-15}	0.499	-8.56
k__Bacteria;p__Firmicutes;c__Clostridia;o__Clostridiales; f__Lachnospiraceae;g__Clostridium;s__clostridioforme	-2.98	4.42×10^{-06}	0.000566	0.648	-4.59
k__Bacteria;p__Firmicutes;c__Clostridia;o__Clostridiales; f__Ruminococcaceae;g__Ruminococcus;s__	-2.93	0.000421	0.0534	0.832	-3.53
k__Bacteria;p__Firmicutes;c__Clostridia;o__Clostridiales; f__Ruminococcaceae;g__Faecalibacterium;s__prausnitzii	-1.9	0.00386	0.486	0.656	-2.89
k__Bacteria;p__Firmicutes;c__Clostridia;o__Clostridiales; f__Lachnospiraceae;g__Blautia;s__producta	2.59	0.00571	0.714	0.936	2.76
k__Bacteria;__;__;__;__;__;__	1.36	0.0253	1	0.609	2.24
k__Bacteria;p__Actinobacteria;c__Actinobacteria;o__Actinomycetales; f__Actinomycetaceae;g__Actinomyces;s__	-0.731	0.144	1	0.501	-1.46
k__Bacteria;p__Actinobacteria;c__Actinobacteria;o__Actinomycetales; f__Corynebacteriaceae;g__Corynebacterium;s__durum	0.165	0.369	1	0.183	0.899
k__Bacteria;p__Actinobacteria;c__Actinobacteria;o__Actinomycetales; f__Micrococcaceae;g__Rothia;s__dentocariosa	0.083	0.783	1	0.301	0.276
k__Bacteria;p__Actinobacteria;c__Actinobacteria;o__Actinomycetales; f__Micrococcaceae;g__Rothia;s__mucilaginosa	0.905	0.0335	1	0.425	2.13
k__Bacteria;p__Actinobacteria;c__Actinobacteria;o__Bifidobacteriales; f__Bifidobacteriaceae;g__Bifidobacterium;__	-0.159	0.821	1	0.704	-0.226
k__Bacteria;p__Actinobacteria;c__Actinobacteria;o__Bifidobacteriales; f__Bifidobacteriaceae;g__Bifidobacterium;s__	-2.37	0.154	1	1.67	-1.43
k__Bacteria;p__Actinobacteria;c__Actinobacteria;o__Bifidobacteriales; f__Bifidobacteriaceae;g__Bifidobacterium;s__adolescentis	-1.13	0.23	1	0.938	-1.2
k__Bacteria;p__Actinobacteria;c__Actinobacteria;o__Bifidobacteriales; f__Bifidobacteriaceae;g__Bifidobacterium;s__bifidum	-0.217	0.887	1	1.53	-0.142
k__Bacteria;p__Actinobacteria;c__Coriobacteriia;o__Coriobacteriales; f__Coriobacteriaceae;g__;s__	0.0219	0.979	1	0.831	0.0264
k__Bacteria;p__Actinobacteria;c__Coriobacteriia;o__Coriobacteriales; f__Coriobacteriaceae;g__Adlercreutzia;s__	-0.26	0.535	1	0.419	-0.62
k__Bacteria;p__Actinobacteria;c__Coriobacteriia;o__Coriobacteriales; f__Coriobacteriaceae;g__Atopobium;s__	0.219	0.56	1	0.375	0.583

k__Bacteria;p__Actinobacteria;c__Coriobacteriia;o__Coriobacteriales; f__Coriobacteriaceae;g__Collinsella;s__aerofaciens	-0.635	0.585	1	1.16	-0.546
k__Bacteria;p__Actinobacteria;c__Coriobacteriia;o__Coriobacteriales; f__Coriobacteriaceae;g__Collinsella;s__stercoris	0.394	0.421	1	0.489	0.805
k__Bacteria;p__Actinobacteria;c__Coriobacteriia;o__Coriobacteriales; f__Coriobacteriaceae;g__Eggerthella;s__lenta	-0.582	0.527	1	0.918	-0.633
k__Bacteria;p__Actinobacteria;c__Coriobacteriia;o__Coriobacteriales; f__Coriobacteriaceae;g__Slackia;s__	-1.03	0.0478	1	0.522	-1.98
k__Bacteria;p__Bacteroidetes;c__Bacteroidia;o__Bacteroidales; f__[Barnesiellaceae];g__;s__	0.0893	0.904	1	0.74	0.121
k__Bacteria;p__Bacteroidetes;c__Bacteroidia;o__Bacteroidales; f__[Odoribacteraceae];g__Butyricimonas;s__	0.618	0.154	1	0.434	1.42
k__Bacteria;p__Bacteroidetes;c__Bacteroidia;o__Bacteroidales; f__[Odoribacteraceae];g__Odoribacter;s__	0.142	0.654	1	0.317	0.448
k__Bacteria;p__Bacteroidetes;c__Bacteroidia;o__Bacteroidales; f__[Paraprevotellaceae];g__Paraprevotella;s__	-0.35	0.575	1	0.624	-0.561
k__Bacteria;p__Bacteroidetes;c__Bacteroidia;o__Bacteroidales; f__Bacteroidaceae;g__Bacteroides;__	-1.03	0.398	1	1.22	-0.844
k__Bacteria;p__Bacteroidetes;c__Bacteroidia;o__Bacteroidales; f__Bacteroidaceae;g__Bacteroides;s__	-0.317	0.614	1	0.629	-0.505
k__Bacteria;p__Bacteroidetes;c__Bacteroidia;o__Bacteroidales; f__Bacteroidaceae;g__Bacteroides;s__caccae	-0.126	0.918	1	1.22	-0.103
k__Bacteria;p__Bacteroidetes;c__Bacteroidia;o__Bacteroidales; f__Bacteroidaceae;g__Bacteroides;s__fragilis	-1.09	0.464	1	1.49	-0.733
k__Bacteria;p__Bacteroidetes;c__Bacteroidia;o__Bacteroidales; f__Bacteroidaceae;g__Bacteroides;s__ovatus	-0.606	0.64	1	1.3	-0.467
k__Bacteria;p__Bacteroidetes;c__Bacteroidia;o__Bacteroidales; f__Bacteroidaceae;g__Bacteroides;s__uniformis	-2.07	0.0097	1	0.799	-2.59
k__Bacteria;p__Bacteroidetes;c__Bacteroidia;o__Bacteroidales; f__Porphyromonadaceae;g__Parabacteroides;s__	-0.595	0.446	1	0.78	-0.762
k__Bacteria;p__Bacteroidetes;c__Bacteroidia;o__Bacteroidales; f__Porphyromonadaceae;g__Parabacteroides;s__distasonis	-0.317	0.809	1	1.31	-0.242
k__Bacteria;p__Bacteroidetes;c__Bacteroidia;o__Bacteroidales; f__Prevotellaceae;g__Prevotella;s__	-0.336	0.687	1	0.834	-0.403
k__Bacteria;p__Bacteroidetes;c__Bacteroidia;o__Bacteroidales; f__Prevotellaceae;g__Prevotella;s__copri	-0.861	0.519	1	1.34	-0.645
k__Bacteria;p__Bacteroidetes;c__Bacteroidia;o__Bacteroidales; f__Rikenellaceae;__;__	-1.04	0.128	1	0.683	-1.52

k__Bacteria;p__Bacteroidetes;c__Bacteroidia;o__Bacteroidales; f__Rikenellaceae;g__;s__	-0.0417	0.942	1	0.576	-0.0723
k__Bacteria;p__Bacteroidetes;c__Bacteroidia;o__Bacteroidales; f__Rikenellaceae;g__Alistipes;s__finegoldii	-1.65	0.0207	1	0.712	-2.31
k__Bacteria;p__Bacteroidetes;c__Bacteroidia;o__Bacteroidales; f__Rikenellaceae;g__Alistipes;s__indistinctus	-1.16	0.0214	1	0.502	-2.3
k__Bacteria;p__Bacteroidetes;c__Bacteroidia;o__Bacteroidales; f__Rikenellaceae;g__Alistipes;s__onderdonkii	-2.02	0.0131	1	0.815	-2.48
k__Bacteria;p__Bacteroidetes;c__Bacteroidia;o__Bacteroidales; f__Rikenellaceae;g__Alistipes;s__putredinis	-0.346	0.752	1	1.1	-0.316
k__Bacteria;p__Cyanobacteria;c__Chloroplast;o__Stramenopiles; f__;g__;s__	-0.0575	0.747	1	0.178	-0.322
k__Bacteria;p__Firmicutes;c__Bacilli;o__Gemellales; f__Gemellaceae;__;__	1.62	0.00919	1	0.623	2.6
k__Bacteria;p__Firmicutes;c__Bacilli;o__Lactobacillales; f__Carnobacteriaceae;__;__	-0.267	0.737	1	0.796	-0.335
k__Bacteria;p__Firmicutes;c__Bacilli;o__Lactobacillales; f__Enterococcaceae;g__Enterococcus;__	1.05	0.098	1	0.636	1.65
k__Bacteria;p__Firmicutes;c__Bacilli;o__Lactobacillales; f__Lactobacillaceae;g__Lactobacillus;s__	1.38	0.123	1	0.899	1.54
k__Bacteria;p__Firmicutes;c__Bacilli;o__Lactobacillales; f__Lactobacillaceae;g__Lactobacillus;s__zeae	1.51	0.0652	1	0.819	1.84
k__Bacteria;p__Firmicutes;c__Bacilli;o__Lactobacillales; f__Streptococcaceae;g__Lactococcus;s__	-0.633	0.264	1	0.567	-1.12
k__Bacteria;p__Firmicutes;c__Bacilli;o__Lactobacillales; f__Streptococcaceae;g__Streptococcus;s__	-0.434	0.651	1	0.959	-0.452
k__Bacteria;p__Firmicutes;c__Bacilli;o__Lactobacillales; f__Streptococcaceae;g__Streptococcus;s__anginosus	0.785	0.142	1	0.535	1.47
k__Bacteria;p__Firmicutes;c__Bacilli;o__Lactobacillales; f__Streptococcaceae;g__Streptococcus;s__infantis	0.469	0.624	1	0.958	0.49
k__Bacteria;p__Firmicutes;c__Bacilli;o__Lactobacillales; f__Streptococcaceae;g__Streptococcus;s__luteciae	0.63	0.37	1	0.702	0.897
k__Bacteria;p__Firmicutes;c__Bacilli;o__Turicibacterales; f__Turicibacteraceae;g__Turicibacter;s__	-0.997	0.349	1	1.06	-0.937
k__Bacteria;p__Firmicutes;c__Clostridia;o__Clostridiales;__;__;__	-0.251	0.628	1	0.518	-0.484
k__Bacteria;p__Firmicutes;c__Clostridia;o__Clostridiales; f__;g__;s__	-1.59	0.112	1	0.999	-1.59
k__Bacteria;p__Firmicutes;c__Clostridia;o__Clostridiales; f__[Mogibacteriaceae];g__;s__	-1.31	0.0579	1	0.688	-1.9
k__Bacteria;p__Firmicutes;c__Clostridia;o__Clostridiales; f__[Mogibacteriaceae];g__Mogibacterium;s__	-0.447	0.248	1	0.387	-1.15

k__Bacteria;p__Firmicutes;c__Clostridia;o__Clostridiales; f__[Tissierellaceae];g__Anaerococcus;s__	1.25	0.0817	1	0.721	1.74
k__Bacteria;p__Firmicutes;c__Clostridia;o__Clostridiales; f__[Tissierellaceae];g__Finegoldia;s__	0.0138	0.979	1	0.532	0.0259
k__Bacteria;p__Firmicutes;c__Clostridia;o__Clostridiales; f__[Tissierellaceae];g__Parvimonas;s__	0.296	0.697	1	0.759	0.39
k__Bacteria;p__Firmicutes;c__Clostridia;o__Clostridiales; f__[Tissierellaceae];g__Peptoniphilus;s__	0.895	0.233	1	0.75	1.19
k__Bacteria;p__Firmicutes;c__Clostridia;o__Clostridiales; f__Christensenellaceae;g__;s__	-0.213	0.786	1	0.783	-0.272
k__Bacteria;p__Firmicutes;c__Clostridia;o__Clostridiales; f__Christensenellaceae;g__Christensenella;s__	-0.00548	0.977	1	0.19	-0.0289
k__Bacteria;p__Firmicutes;c__Clostridia;o__Clostridiales; f__Clostridiaceae;g__;s__	-0.425	0.666	1	0.985	-0.432
k__Bacteria;p__Firmicutes;c__Clostridia;o__Clostridiales; f__Clostridiaceae;g__Clostridium;__	1.08	0.0764	1	0.609	1.77
k__Bacteria;p__Firmicutes;c__Clostridia;o__Clostridiales; f__Clostridiaceae;g__Clostridium;s__	-2.41	0.0131	1	0.97	-2.48
k__Bacteria;p__Firmicutes;c__Clostridia;o__Clostridiales; f__Clostridiaceae;g__Clostridium;s__celatum	-0.871	0.389	1	1.01	-0.862
k__Bacteria;p__Firmicutes;c__Clostridia;o__Clostridiales; f__Clostridiaceae;g__Clostridium;s__paraputrificum	0.46	0.326	1	0.469	0.983
k__Bacteria;p__Firmicutes;c__Clostridia;o__Clostridiales; f__Clostridiaceae;g__SMB53;s__	0.484	0.388	1	0.56	0.864
k__Bacteria;p__Firmicutes;c__Clostridia;o__Clostridiales; f__Eubacteriaceae;g__Anaerofustis;s__	-0.287	0.453	1	0.382	-0.751
k__Bacteria;p__Firmicutes;c__Clostridia;o__Clostridiales; f__Eubacteriaceae;g__Pseudoramibacter_Eubacterium;s__	-0.453	0.433	1	0.577	-0.784
k__Bacteria;p__Firmicutes;c__Clostridia;o__Clostridiales; f__Lachnospiraceae;__;__	-0.379	0.721	1	1.06	-0.358
k__Bacteria;p__Firmicutes;c__Clostridia;o__Clostridiales; f__Lachnospiraceae;g__;s__	-1.36	0.126	1	0.892	-1.53
k__Bacteria;p__Firmicutes;c__Clostridia;o__Clostridiales; f__Lachnospiraceae;g__[Ruminococcus];s__	-1.61	0.0228	1	0.708	-2.28
k__Bacteria;p__Firmicutes;c__Clostridia;o__Clostridiales; f__Lachnospiraceae;g__[Ruminococcus];s__gnavus	0.358	0.782	1	1.3	0.276
k__Bacteria;p__Firmicutes;c__Clostridia;o__Clostridiales; f__Lachnospiraceae;g__[Ruminococcus];s__torques	-0.211	0.864	1	1.24	-0.171

k__Bacteria;p__Firmicutes;c__Clostridia;o__Clostridiales; f__Lachnospiraceae;g__Anaerostipes;s__	0.489	0.523	1	0.766	0.638
k__Bacteria;p__Firmicutes;c__Clostridia;o__Clostridiales; f__Lachnospiraceae;g__Blautia;__	-0.616	0.464	1	0.842	-0.732
k__Bacteria;p__Firmicutes;c__Clostridia;o__Clostridiales; f__Lachnospiraceae;g__Blautia;s__	-0.765	0.143	1	0.523	-1.46
k__Bacteria;p__Firmicutes;c__Clostridia;o__Clostridiales; f__Lachnospiraceae;g__Blautia;s__obeum	-1.26	0.357	1	1.36	-0.922
k__Bacteria;p__Firmicutes;c__Clostridia;o__Clostridiales; f__Lachnospiraceae;g__Clostridium;__	0.737	0.433	1	0.939	0.784
k__Bacteria;p__Firmicutes;c__Clostridia;o__Clostridiales; f__Lachnospiraceae;g__Clostridium;s__aldenense	0.33	0.566	1	0.575	0.574
k__Bacteria;p__Firmicutes;c__Clostridia;o__Clostridiales; f__Lachnospiraceae;g__Clostridium;s__hathewayi	0.334	0.678	1	0.802	0.416
k__Bacteria;p__Firmicutes;c__Clostridia;o__Clostridiales; f__Lachnospiraceae;g__Clostridium;s__symbiosum	0.45	0.592	1	0.841	0.535
k__Bacteria;p__Firmicutes;c__Clostridia;o__Clostridiales; f__Lachnospiraceae;g__Coprococcus;s__	-0.897	0.119	1	0.575	-1.56
k__Bacteria;p__Firmicutes;c__Clostridia;o__Clostridiales; f__Lachnospiraceae;g__Coprococcus;s__catus	-0.0805	0.94	1	1.08	-0.0747
k__Bacteria;p__Firmicutes;c__Clostridia;o__Clostridiales; f__Lachnospiraceae;g__Coprococcus;s__eutactus	0.351	0.633	1	0.736	0.477
k__Bacteria;p__Firmicutes;c__Clostridia;o__Clostridiales; f__Lachnospiraceae;g__Dorea;s__	2.21	0.115	1	1.4	1.58
k__Bacteria;p__Firmicutes;c__Clostridia;o__Clostridiales; f__Lachnospiraceae;g__Dorea;s__formicigenerans	-0.31	0.8	1	1.22	-0.254
k__Bacteria;p__Firmicutes;c__Clostridia;o__Clostridiales; f__Lachnospiraceae;g__Dorea;s__longicatena	-2.24	0.0159	1	0.927	-2.41
k__Bacteria;p__Firmicutes;c__Clostridia;o__Clostridiales; f__Lachnospiraceae;g__Lachnospira;s__	-0.605	0.548	1	1.01	-0.601
k__Bacteria;p__Firmicutes;c__Clostridia;o__Clostridiales; f__Lachnospiraceae;g__Roseburia;s__	-0.829	0.538	1	1.35	-0.616
k__Bacteria;p__Firmicutes;c__Clostridia;o__Clostridiales; f__Lachnospiraceae;g__Roseburia;s__faecis	-0.989	0.511	1	1.5	-0.658
k__Bacteria;p__Firmicutes;c__Clostridia;o__Clostridiales; f__Lachnospiraceae;g__Roseburia;s__inulinivorans	-0.856	0.476	1	1.2	-0.713
k__Bacteria;p__Firmicutes;c__Clostridia;o__Clostridiales; f__Lachnospiraceae;g__Ruminococcus;s__lactaris	-1.74	0.179	1	1.29	-1.34

k__Bacteria;p__Firmicutes;c__Clostridia;o__Clostridiales; f__Peptostreptococcaceae;g__[Clostridium];__	0.191	0.684	1	0.469	0.407
k__Bacteria;p__Firmicutes;c__Clostridia;o__Clostridiales; f__Peptostreptococcaceae;g__Peptostreptococcus;s__	1.18	0.0955	1	0.71	1.67
k__Bacteria;p__Firmicutes;c__Clostridia;o__Clostridiales; f__Ruminococcaceae;__;__	-1.54	0.00892	1	0.588	-2.62
k__Bacteria;p__Firmicutes;c__Clostridia;o__Clostridiales; f__Ruminococcaceae;g__;s__	-1.17	0.329	1	1.2	-0.975
k__Bacteria;p__Firmicutes;c__Clostridia;o__Clostridiales; f__Ruminococcaceae;g__Anaerotruncus;s__	-0.514	0.0943	1	0.307	-1.67
k__Bacteria;p__Firmicutes;c__Clostridia;o__Clostridiales; f__Ruminococcaceae;g__Butyricicoccus;s__pullicaecorum	0.0738	0.93	1	0.836	0.0883
k__Bacteria;p__Firmicutes;c__Clostridia;o__Clostridiales; f__Ruminococcaceae;g__Clostridium;s__methylpentosum	0.0341	0.895	1	0.257	0.133
k__Bacteria;p__Firmicutes;c__Clostridia;o__Clostridiales; f__Ruminococcaceae;g__Oscillospira;s__	-0.712	0.273	1	0.65	-1.1
k__Bacteria;p__Firmicutes;c__Clostridia;o__Clostridiales; f__Ruminococcaceae;g__Ruminococcus;s__bromii	0.0255	0.985	1	1.31	0.0194
k__Bacteria;p__Firmicutes;c__Clostridia;o__Clostridiales; f__Ruminococcaceae;g__Ruminococcus;s__callidus	-1.27	0.257	1	1.12	-1.13
k__Bacteria;p__Firmicutes;c__Clostridia;o__Clostridiales; f__Ruminococcaceae;g__Ruminococcus;s__flavefaciens	-1.05	0.348	1	1.12	-0.939
k__Bacteria;p__Firmicutes;c__Clostridia;o__Clostridiales; f__Veillonellaceae;g__Dialister;s__	1.22	0.325	1	1.24	0.985
k__Bacteria;p__Firmicutes;c__Clostridia;o__Clostridiales; f__Veillonellaceae;g__Phascolarctobacterium;s__	0.249	0.682	1	0.609	0.41
k__Bacteria;p__Firmicutes;c__Clostridia;o__Clostridiales; f__Veillonellaceae;g__Veillonella;s__dispar	0.497	0.546	1	0.825	0.603
k__Bacteria;p__Firmicutes;c__Clostridia;o__Clostridiales; f__Veillonellaceae;g__Veillonella;s__parvula	0.713	0.138	1	0.481	1.48
k__Bacteria;p__Firmicutes;c__Erysipelotrichi;o__Erysipelotrichales; f__Erysipelotrichaceae;g__;s__	0.842	0.469	1	1.16	0.725
k__Bacteria;p__Firmicutes;c__Erysipelotrichi;o__Erysipelotrichales; f__Erysipelotrichaceae;g__[Eubacterium];s__biforme	-2.05	0.194	1	1.58	-1.3
k__Bacteria;p__Firmicutes;c__Erysipelotrichi;o__Erysipelotrichales; f__Erysipelotrichaceae;g__[Eubacterium];s__dolichum	-0.447	0.426	1	0.562	-0.795
k__Bacteria;p__Firmicutes;c__Erysipelotrichi;o__Erysipelotrichales; f__Erysipelotrichaceae;g__Bulleidia;s__moorei	-0.329	0.375	1	0.371	-0.888

k__Bacteria;p__Firmicutes;c__Erysipelotrichi;o__Erysipelotrichales; f__Erysipelotrichaceae;g__Clostridium;s__ramosum	0.2	0.864	1	1.16	0.172
k__Bacteria;p__Firmicutes;c__Erysipelotrichi;o__Erysipelotrichales; f__Erysipelotrichaceae;g__Clostridium;s__saccharogumia	-0.976	0.0858	1	0.568	-1.72
k__Bacteria;p__Firmicutes;c__Erysipelotrichi;o__Erysipelotrichales; f__Erysipelotrichaceae;g__Clostridium;s__spiroforme	0.804	0.445	1	1.05	0.764
k__Bacteria;p__Firmicutes;c__Erysipelotrichi;o__Erysipelotrichales; f__Erysipelotrichaceae;g__Coprobacillus;s__	0.929	0.0954	1	0.557	1.67
k__Bacteria;p__Firmicutes;c__Erysipelotrichi;o__Erysipelotrichales; f__Erysipelotrichaceae;g__Holdemania;s__	0.769	0.0348	1	0.364	2.11
k__Bacteria;p__Fusobacteria;c__Fusobacteriia;o__Fusobacteriales; f__Fusobacteriaceae;g__Fusobacterium;s__	1.11	0.095	1	0.663	1.67
k__Bacteria;p__Proteobacteria;c__Betaproteobacteria;o__Burkholderiales; f__Alcaligenaceae;g__Achromobacter;s__	0.000267	0.999	1	0.204	0.00131
k__Bacteria;p__Proteobacteria;c__Betaproteobacteria;o__Burkholderiales; f__Alcaligenaceae;g__Sutterella;s__	-0.405	0.684	1	0.996	-0.407
k__Bacteria;p__Proteobacteria;c__Deltaproteobacteria;o__Desulfovibrionales; f__Desulfovibrionaceae;g__Bilophila;s__	0.49	0.481	1	0.695	0.705
k__Bacteria;p__Proteobacteria;c__Gammaproteobacteria;o__Enterobacteriales; f__Enterobacteriaceae;__;__	2.38	0.025	1	1.06	2.24
k__Bacteria;p__Proteobacteria;c__Gammaproteobacteria;o__Enterobacteriales; f__Enterobacteriaceae;g__Proteus;s__	0.34	0.403	1	0.406	0.836
k__Bacteria;p__Proteobacteria;c__Gammaproteobacteria;o__Pasteurellales; f__Pasteurellaceae;g__Aggregatibacter;s__	0.124	0.681	1	0.301	0.411
k__Bacteria;p__Proteobacteria;c__Gammaproteobacteria;o__Pasteurellales; f__Pasteurellaceae;g__Haemophilus;s__parainfluenzae	0.958	0.214	1	0.772	1.24
k__Bacteria;p__Tenericutes;c__Mollicutes;o__RF39; f__;g__;s__	0.146	0.713	1	0.397	0.367
k__Bacteria;p__Verrucomicrobia;c__Verrucomicrobiae;o__Verrucomicrobiales; f__Verrucomicrobiaceae;g__Akkermansia;s__muciniphila	-0.776	0.391	1	0.905	-0.857
