# Supplementary material for: Multiomics-Based Profiling of the Fecal Microbiome Reveals Potential Disease-Specific Signatures in Pediatric IBD (PIBD)
Source: Biomolecules. 2025 May 21;15(5):746. doi: 10.3390/biom15050746 (PMC12109367; doi:10.3390/biom15050746)
Supplement: Supplementary file 1 [file biomolecules-15-00746-s001.zip › supplemental6-maaslin-diseasegroup-taxaabundance.pdf]

Supplemental Table S6

Differential taxonomic abundance in UC and Crohn’s as compared to healthy microbiome, calculated using MaAsLin3.

Pathway	Condition	Effect Size	Standard Error	P-Value	Q-Value
Gemmiger_formicilis	All Ulcerative Colitis	-5.78	1.49	1.23×10^{-03}	0.573
Streptococcus_sanguinis	All Ulcerative Colitis	4.69	0.99	2.16×10^{-03}	0.67
Abiotrophia_defectiva	All Ulcerative Colitis	-0.7	1.4	0.612	1.
Actinobaculum_sp_oral_taxon_183	All Crohn's Disease	-3.69	2.15	0.206	1.
Actinobaculum_sp_oral_taxon_183	All Ulcerative Colitis	-1.81	2.53	0.578	1.
Actinomyces_gerencseriae	All Crohn's Disease	-0.139	4.26	0.51	1.
Actinomyces_gerencseriae	All Ulcerative Colitis	4.37	9.85	0.237	1.
Actinomyces_graevenitzii	All Crohn's Disease	-2.85	1.68	0.165	1.
Actinomyces_graevenitzii	All Ulcerative Colitis	-1.71	1.68	0.317	1.
Actinomyces_johnsonii	All Ulcerative Colitis	0.406	2.28	0.991	1.
Actinomyces_massiliensis	All Crohn's Disease	-3.1	2.84	0.575	1.
Actinomyces_massiliensis	All Ulcerative Colitis	0.593	2.59	0.999	1.
Actinomyces_naeslundii	All Crohn's Disease	1.51	1.33	0.576	1.
Actinomyces_naeslundii	All Ulcerative Colitis	1.08	1.34	0.563	1.
Actinomyces_oris	All Crohn's Disease	-1.7	1.43	0.309	1.
Actinomyces_oris	All Ulcerative Colitis	1.42×10^{-03}	1.46	0.85	1.
Actinomyces_SGB17154	All Crohn's Disease	-1.85	3.94	0.259	1.
Actinomyces_SGB17154	All Ulcerative Colitis	-0.0829	5.24	0.421	1.
Actinomyces_SGB17168	All Crohn's Disease	-0.179	1.68	0.944	1.
Actinomyces_SGB17168	All Ulcerative Colitis	1.85	1.86	0.818	1.
Actinomyces_sp_ICM47	All Crohn's Disease	-1.74	1.38	0.283	1.
Actinomyces_sp_ICM47	All Ulcerative Colitis	0.587	1.41	0.369	1.
Actinomyces_sp_ICM58	All Crohn's Disease	-0.365	2.42	0.443	1.
Actinomyces_sp_ICM58	All Ulcerative Colitis	1.18	2.72	0.125	1.
Actinomyces_sp_S6_Spd3	All Crohn's Disease	-0.108	1.98	0.898	1.
Actinomyces_sp_S6_Spd3	All Ulcerative Colitis	3.06	2.51	0.64	1.
Adlercreutzia_equolifaciens	All Crohn's Disease	0.212	1.22	1.	1.
Adlercreutzia_equolifaciens	All Ulcerative Colitis	0.0612	1.27	0.776	1.
Agathobaculum_butyriciproducens	All Crohn's Disease	1.35	0.849	0.451	1.
Agathobaculum_butyriciproducens	All Ulcerative Colitis	3.15	1.05	0.0779	1.
Alistipes_communis	All Crohn's Disease	2.12	1.31	0.524	1.
Alistipes_communis	All Ulcerative Colitis	-2.68	1.34	0.234	1.
Alistipes_finegoldii	All Crohn's Disease	-0.295	0.931	0.762	1.
Alistipes_ihumii	All Crohn's Disease	1.95	1.63	0.755	1.
Alistipes_ihumii	All Ulcerative Colitis	-1.05	1.43	0.672	1.
Alistipes_onderdonkii	All Crohn's Disease	1.61	1.07	0.465	1.
Alistipes_onderdonkii	All Ulcerative Colitis	-1.66	1.3	0.134	1.
Alistipes_putredinis	All Crohn's Disease	3.53	2.42	0.382	1.
Alistipes_putredinis	All Ulcerative Colitis	-1.7	2.57	0.592	1.
Alistipes_shahii	All Crohn's Disease	1.91	1.07	0.334	1.
Alistipes_shahii	All Ulcerative Colitis	1.97	1.17	0.229	1.
Anaerobutyricum_hallii	All Crohn's Disease	-0.267	0.705	0.636	1.
Anaerobutyricum_hallii	All Ulcerative Colitis	0.217	0.707	0.708	1.
Anaerofustis_stercorihominis	All Crohn's Disease	-1.6	1.78	0.532	1.
Anaerofustis_stercorihominis	All Ulcerative Colitis	-0.496	1.81	0.765	1.
Anaeromassilibacillus_sp_An250	All Crohn's Disease	-0.855	2.86	0.732	1.
Anaeromassilibacillus_sp_An250	All Ulcerative Colitis	1.67	2.87	0.788	1.
Anaerostipes_caccae	All Crohn's Disease	-0.646	3.78	0.318	1.
Anaerostipes_caccae	All Ulcerative Colitis	1.38	3.48	0.551	1.
Anaerostipes_hadrus	All Crohn's Disease	2.28	2.12	0.606	1.
Anaerostipes_hadrus	All Ulcerative Colitis	1.75	2.09	0.674	1.
Anaerotignum_faecicola	All Crohn's Disease	0.509	2.72	0.832	1.
Anaerotignum_faecicola	All Ulcerative Colitis	1.68	4.24	0.814	1.
Anaerotruncus_colihominis	All Crohn's Disease	2.85	1.18	0.192	1.
Anaerotruncus_colihominis	All Ulcerative Colitis	0.687	1.2	0.427	1.
Bacteroides_caccae	All Crohn's Disease	2.05	1.8	0.596	1.
Bacteroides_caccae	All Ulcerative Colitis	0.0447	1.8	0.911	1.
Bacteroides_eggerthii	All Ulcerative Colitis	-6.63	6.42	0.703	1.

Bacteroides_fragilis	All Crohn's Disease	0.164	2.1	0.872	1.
Bacteroides_fragilis	All Ulcerative Colitis	-2.74	2.21	0.269	1.
Bacteroides_ovatus	All Crohn's Disease	-0.585	1.57	0.808	1.
Bacteroides_ovatus	All Ulcerative Colitis	-1.25	1.59	0.403	1.
Bacteroides_stercoris	All Crohn's Disease	2.05	2.49	0.685	1.
Bacteroides_stercoris	All Ulcerative Colitis	1.66	2.61	0.931	1.
Bacteroides_thetaiotaomicron	All Crohn's Disease	-1.69	2.3	0.634	1.
Bacteroides_thetaiotaomicron	All Ulcerative Colitis	-1.48	2.39	0.605	1.
Bacteroides_uniformis	All Crohn's Disease	-1.34	1.53	0.486	1.
Bacteroides_uniformis	All Ulcerative Colitis	-1.01	1.57	0.474	1.
Bacteroides_xylanisolvens	All Crohn's Disease	-1.85	4.72	0.848	1.
Bacteroides_xylanisolvens	All Ulcerative Colitis	-0.232	5.98	0.666	1.
Barnesiella_intestinihominis	All Crohn's Disease	0.625	4.54	0.856	1.
Barnesiella_intestinihominis	All Ulcerative Colitis	1.03	4.95	0.979	1.
Bifidobacterium_animalis	All Ulcerative Colitis	1.92	3.54	0.959	1.
Bifidobacterium_bifidum	All Crohn's Disease	-8.9	4.45	0.138	1.
Bifidobacterium_bifidum	All Ulcerative Colitis	-3.	4.4	0.662	1.
Bifidobacterium_dentium	All Crohn's Disease	3.21	9.69	0.739	1.
Bifidobacterium_dentium	All Ulcerative Colitis	7.61	9.1	0.831	1.
Bifidobacterium_longum	All Crohn's Disease	-3.07	2.14	0.234	1.
Bifidobacterium_longum	All Ulcerative Colitis	-1.35	2.09	0.546	1.
Bifidobacterium_pseudocatenulatum	All Crohn's Disease	-3.93	2.88	0.309	1.
Bifidobacterium_pseudocatenulatum	All Ulcerative Colitis	-6.42	2.93	0.0678	1.
Bilophila_wadsworthia	All Crohn's Disease	1.51	2.39	0.713	1.
Bilophila_wadsworthia	All Ulcerative Colitis	2.24	2.74	0.222	1.
Bittarella_massiliensis	All Crohn's Disease	1.9	2.05	0.731	1.
Bittarella_massiliensis	All Ulcerative Colitis	0.257	2.13	0.969	1.
Blautia_caecimuris	All Crohn's Disease	1.73	3.73	0.469	1.
Blautia_caecimuris	All Ulcerative Colitis	3.11	3.36	0.628	1.
Blautia_faecis	All Crohn's Disease	-1.47	1.71	0.508	1.
Blautia_faecis	All Ulcerative Colitis	-1.7	1.7	0.303	1.
Blautia_massiliensis	All Crohn's Disease	-0.861	1.84	0.768	1.
Blautia_massiliensis	All Ulcerative Colitis	-0.816	1.88	0.451	1.
Blautia_obeum	All Crohn's Disease	-1.27	1.32	0.42	1.
Blautia_obeum	All Ulcerative Colitis	-0.138	1.35	0.768	1.
Blautia_producta	All Crohn's Disease	-0.347	2.64	0.959	1.
Blautia_producta	All Ulcerative Colitis	3.62	2.7	0.529	1.
Blautia_schinkii	All Crohn's Disease	3.1	0.368	0.162	1.
Blautia_schinkii	All Ulcerative Colitis	-1.65	0.389	0.194	1.
Blautia_SGB4805	All Crohn's Disease	-0.579	1.56	0.811	1.
Blautia_SGB4805	All Ulcerative Colitis	-0.216	1.82	0.222	1.
Blautia_SGB4815	All Crohn's Disease	-1.77	1.79	0.464	1.
Blautia_SGB4815	All Ulcerative Colitis	-1.14	1.87	0.564	1.
Blautia_SGB4831	All Crohn's Disease	-1.37	4.52	0.947	1.
Blautia_SGB4831	All Ulcerative Colitis	-0.584	5.69	0.712	1.
Blautia_sp_OF03_15BH	All Crohn's Disease	4.88	2.9	0.594	1.
Blautia_sp_OF03_15BH	All Ulcerative Colitis	3.69	3.97	0.836	1.
Blautia_wexlerae	All Crohn's Disease	-1.33	1.2	0.319	1.
Blautia_wexlerae	All Ulcerative Colitis	-0.361	1.15	0.57	1.
Candidatus_Avimicrobium_caecorum	All Crohn's Disease	-0.0352	2.18	0.969	1.
Candidatus_Avimicrobium_caecorum	All Ulcerative Colitis	-1.42	2.23	0.677	1.
Candidatus_Cibiobacter_qucibialis	All Crohn's Disease	-0.26	2.33	0.776	1.
Candidatus_Cibiobacter_qucibialis	All Ulcerative Colitis	-1.92	2.51	0.528	1.
Candidatus_Saccharibacteria_unclassified_SGB19850	All Crohn's Disease	-2.19	2.4	0.133	1.
Candidatus_Saccharibacteria_unclassified_SGB19850	All Ulcerative Colitis	-1.41	2.16	0.555	1.
Christensenella_minuta	All Crohn's Disease	-1.18	1.72	0.717	1.
Christensenella_minuta	All Ulcerative Colitis	1.96	1.73	0.806	1.
Christensenellaceae_bacterium	All Crohn's Disease	-0.553	2.5	0.763	1.
Christensenellaceae_bacterium	All Ulcerative Colitis	1.33	2.73	0.399	1.
Clostridia_bacterium	All Crohn's Disease	-1.94	1.38	0.218	1.
Clostridia_bacterium	All Ulcerative Colitis	-2.9	1.47	0.0462	1.
Clostridia_bacterium_UC5_1_1D1	All Crohn's Disease	-0.718	1.54	0.658	1.

Clostridia_bacterium_UC5_1_1D1	All Ulcerative Colitis	1.19	1.55	0.336	1.
Clostridia_unclassified_SGB4121	All Crohn's Disease	0.578	0.966	0.847	1.
Clostridia_unclassified_SGB4121	All Ulcerative Colitis	-0.0174	0.995	0.58	1.
Clostridiaceae_bacterium	All Crohn's Disease	1.17	0.815	0.55	1.
Clostridiaceae_bacterium	All Ulcerative Colitis	-0.44	0.887	0.281	1.
Clostridiaceae_bacterium_OM08_6BH	All Crohn's Disease	2.73	2.07	0.338	1.
Clostridiaceae_bacterium_OM08_6BH	All Ulcerative Colitis	2.19	4.92	0.0472	1.
Clostridiales_bacterium	All Crohn's Disease	3.35	1.42	0.192	1.
Clostridiales_bacterium_KLE1615	All Crohn's Disease	1.41	1.74	0.801	1.
Clostridiales_bacterium_KLE1615	All Ulcerative Colitis	0.98	1.72	0.643	1.
Clostridium_disporicum	All Crohn's Disease	-0.731	1.72	0.459	1.
Clostridium_disporicum	All Ulcerative Colitis	2.72	2.14	0.124	1.
Clostridium_fessum	All Crohn's Disease	-1.06	2.3	0.337	1.
Clostridium_fessum	All Ulcerative Colitis	-0.0709	2.58	0.172	1.
Clostridium_innocuum	All Crohn's Disease	-2.24	1.58	0.226	1.
Clostridium_innocuum	All Ulcerative Colitis	-1.57	1.42	0.226	1.
Clostridium_leptum	All Crohn's Disease	-1.62	1.83	0.51	1.
Clostridium_leptum	All Ulcerative Colitis	1.65	2.08	0.185	1.
Clostridium_paraputrificum	All Crohn's Disease	6.6	4.65	0.525	1.
Clostridium_paraputrificum	All Ulcerative Colitis	-0.788	3.63	0.917	1.
Clostridium_phoceensis	All Crohn's Disease	-1.33	2.19	0.716	1.
Clostridium_phoceensis	All Ulcerative Colitis	-0.26	2.43	0.643	1.
Clostridium_saccharogumia	All Crohn's Disease	-2.36	1.49	0.537	1.
Clostridium_scindens	All Crohn's Disease	-1.67	1.84	0.5	1.
Clostridium_scindens	All Ulcerative Colitis	0.0176	2.3	0.338	1.
Clostridium_SGB6179	All Crohn's Disease	5.17	2.32	0.175	1.
Clostridium_SGB6179	All Ulcerative Colitis	7.38	2.73	0.116	1.
Clostridium_sp_AF20_17LB	All Crohn's Disease	1.8	2.89	0.864	1.
Clostridium_sp_AF20_17LB	All Ulcerative Colitis	0.753	3.73	0.522	1.
Clostridium_sp_AF34_10BH	All Crohn's Disease	-0.934	1.72	0.296	1.
Clostridium_sp_AF34_10BH	All Ulcerative Colitis	-1.69	1.91	0.402	1.
Clostridium_sp_AF36_4	All Crohn's Disease	2.28	2.68	0.753	1.
Clostridium_sp_AF36_4	All Ulcerative Colitis	1.26	2.99	0.389	1.
Clostridium_sp_AM22_11AC	All Crohn's Disease	0.143	1.27	0.554	1.
Clostridium_sp_AM22_11AC	All Ulcerative Colitis	-0.0815	1.38	0.23	1.
Clostridium_sp_AM33_3	All Crohn's Disease	0.348	0.969	0.958	1.
Clostridium_sp_AM33_3	All Ulcerative Colitis	-1.37	1.07	0.179	1.
Clostridium_sp_AT4	All Crohn's Disease	-3.71	4.15	0.676	1.
Clostridium_sp_AT4	All Ulcerative Colitis	1.84	4.53	0.935	1.
Clostridium_sp_NSJ_42	All Crohn's Disease	1.25	2.14	0.706	1.
Clostridium_sp_NSJ_42	All Ulcerative Colitis	5.83	3.69	0.456	1.
Clostridium_spiroforme	All Crohn's Disease	1.44	1.58	0.758	1.
Clostridium_spiroforme	All Ulcerative Colitis	1.92	1.51	0.708	1.
Clostridium_symbiosum	All Crohn's Disease	2.89	2.56	0.564	1.
Clostridium_symbiosum	All Ulcerative Colitis	4.47	2.62	0.329	1.
Collinsella_aerofaciens	All Crohn's Disease	-3.42	3.74	0.573	1.
Collinsella_aerofaciens	All Ulcerative Colitis	0.49	4.35	0.589	1.
Coprococcus_catus	All Crohn's Disease	0.877	0.432	0.474	1.
Coprococcus_catus	All Ulcerative Colitis	0.706	0.493	0.997	1.
Coprococcus_comes	All Crohn's Disease	0.0668	1.03	0.744	1.
Coprococcus_comes	All Ulcerative Colitis	0.798	1.29	0.702	1.
Corynebacterium_durum	All Crohn's Disease	-1.15	3.73	0.925	1.
Corynebacterium_durum	All Ulcerative Colitis	-0.184	3.79	0.97	1.
Dialister_invisus	All Crohn's Disease	1.67	1.44	0.612	1.
Dialister_invisus	All Ulcerative Colitis	2.56	1.39	0.388	1.
Dielma_fastidiosa	All Crohn's Disease	5.22	3.95	0.471	1.
Dielma_fastidiosa	All Ulcerative Colitis	3.75	4.66	0.417	1.
Dorea_formicigenerans	All Crohn's Disease	-0.218	1.07	0.522	1.
Dorea_formicigenerans	All Ulcerative Colitis	0.245	1.15	0.889	1.
Dorea_longicatena	All Crohn's Disease	0.506	0.673	0.698	1.
Dorea_longicatena	All Ulcerative Colitis	0.983	0.698	0.366	1.
Dorea_sp_AF24_7LB	All Crohn's Disease	-1.96	1.25	0.232	1.

Dorea_sp_AF24_7LB	All Ulcerative Colitis	6.18	2.96	0.0471	1.
Dorea_sp_AF36_15AT	All Crohn's Disease	0.501	0.616	0.542	1.
Dorea_sp_AF36_15AT	All Ulcerative Colitis	-0.384	0.629	0.309	1.
Dysosmobacter_sp_NSJ_60	All Crohn's Disease	3.76	3.9	0.514	1.
Dysosmobacter_sp_NSJ_60	All Ulcerative Colitis	3.19	3.6	0.621	1.
Dysosmobacter_welbionis	All Crohn's Disease	-1.64	1.29	0.275	1.
Dysosmobacter_welbionis	All Ulcerative Colitis	-1.65	1.6	0.276	1.
Eggerthella_lenta	All Crohn's Disease	-0.506	1.71	0.858	1.
Eggerthella_lenta	All Ulcerative Colitis	1.18	1.83	0.39	1.
Eggerthellaceae_unclassified_SGB14341	All Crohn's Disease	-3.26	0.845	0.0257	1.
Eggerthellaceae_unclassified_SGB14341	All Ulcerative Colitis	-5.79	0.956	4.77×10^{-03}	1.
Eisenbergiella_massiliensis	All Crohn's Disease	4.52	2.98	0.342	1.
Eisenbergiella_massiliensis	All Ulcerative Colitis	5.51	2.98	0.26	1.
Enterocloster_aldensis	All Crohn's Disease	1.97	2.62	0.798	1.
Enterocloster_aldensis	All Ulcerative Colitis	1.53	2.6	0.948	1.
Enterocloster_asparagiformis	All Crohn's Disease	0.384	1.18	0.984	1.
Enterocloster_asparagiformis	All Ulcerative Colitis	1.72	1.28	0.719	1.
Enterocloster_bolteae	All Crohn's Disease	0.0918	1.77	0.798	1.
Enterocloster_bolteae	All Ulcerative Colitis	0.888	1.79	0.891	1.
Enterocloster_clostridioformis	All Crohn's Disease	-1.68	1.86	0.521	1.
Enterocloster_clostridioformis	All Ulcerative Colitis	1.61	1.85	0.632	1.
Enterocloster_lavalensis	All Crohn's Disease	2.14	0.951	0.361	1.
Enterocloster_lavalensis	All Ulcerative Colitis	11.6	2.53	0.0976	1.
Enterococcus_faecalis	All Crohn's Disease	6.74	7.21	0.718	1.
Enterococcus_faecalis	All Ulcerative Colitis	8.17	6.71	0.621	1.
Erysipelatoclostridium_ramosum	All Crohn's Disease	-0.14	3.22	0.944	1.
Erysipelatoclostridium_ramosum	All Ulcerative Colitis	-0.236	3.17	0.942	1.
Erysipelotrichaceae_bacterium_3_1_53	All Crohn's Disease	-3.17	0.988	0.136	1.
Erysipelotrichaceae_bacterium_3_1_53	All Ulcerative Colitis	-2.24	1.03	0.19	1.
Escherichia_coli	All Crohn's Disease	-1.15	4.07	0.3	1.
Escherichia_coli	All Ulcerative Colitis	2.28	4.12	0.621	1.
Eubacteriaceae_bacterium	All Crohn's Disease	1.22	1.16	0.724	1.
Eubacteriaceae_bacterium	All Ulcerative Colitis	-0.324	1.24	0.648	1.
Eubacterium_brachy	All Crohn's Disease	-3.33	3.42	0.569	1.
Eubacterium_brachy	All Ulcerative Colitis	-1.52	3.03	0.745	1.
Eubacterium_ramulus	All Crohn's Disease	3.58	2.62	0.426	1.
Eubacterium_ramulus	All Ulcerative Colitis	2.1	2.77	0.868	1.
Eubacterium_rectale	All Crohn's Disease	2.13	2.55	0.742	1.
Eubacterium_rectale	All Ulcerative Colitis	1.03	2.51	0.75	1.
Eubacterium_siraeum	All Crohn's Disease	-1.55	3.06	0.839	1.
Eubacterium_siraeum	All Ulcerative Colitis	-8.95	3.21	0.18	1.
Eubacterium_sp_AF15_50	All Crohn's Disease	4.08	0.735	0.072	1.
Eubacterium_sp_AF15_50	All Ulcerative Colitis	1.36	1.28	0.713	1.
Eubacterium_sulci	All Crohn's Disease	-0.582	1.57	0.816	1.
Eubacterium_sulci	All Ulcerative Colitis	-0.372	1.64	0.763	1.
Eubacterium_ventriosum	All Crohn's Disease	0.42	2.88	0.872	1.
Eubacterium_ventriosum	All Ulcerative Colitis	2.38	2.78	0.699	1.
Evtepia_gabavorous	All Crohn's Disease	1.6	1.67	0.668	1.
Evtepia_gabavorous	All Ulcerative Colitis	-0.292	1.68	0.802	1.
Faecalibacillus_faecis	All Crohn's Disease	-3.02	3.23	0.586	1.
Faecalibacillus_faecis	All Ulcerative Colitis	-9.3	4.34	0.156	1.
Faecalibacillus_intestinalis	All Crohn's Disease	-1.34	2.16	0.535	1.
Faecalibacillus_intestinalis	All Ulcerative Colitis	1.43	2.23	0.945	1.
Faecalibacterium_prausnitzii	All Crohn's Disease	0.059	1.99	0.868	1.
Faecalibacterium_prausnitzii	All Ulcerative Colitis	-2.6	1.94	0.186	1.
Faecalibacterium_SGB15346	All Crohn's Disease	-2.25	3.23	0.516	1.
Faecalibacterium_SGB15346	All Ulcerative Colitis	-3.85	3.33	0.376	1.
Faecalicatena_contorta	All Crohn's Disease	-0.0967	1.13	0.538	1.
Faecalicatena_contorta	All Ulcerative Colitis	2.28	1.2	0.254	1.
Faecalicatena_fissicatena	All Crohn's Disease	2.96	2.8	0.607	1.
Faecalicatena_fissicatena	All Ulcerative Colitis	0.985	2.72	0.943	1.
Faecalimonas_umbilicata	All Crohn's Disease	2.69	4.04	0.821	1.

Faecalimonas_umbilicata	All Ulcerative Colitis	3.22	3.79	0.781	1.
Firmicutes_bacterium_AF16_15	All Crohn's Disease	-1.61	1.62	0.431	1.
Firmicutes_bacterium_AF16_15	All Ulcerative Colitis	-3.77	1.87	0.0587	1.
Flavonifractor_plautii	All Crohn's Disease	-0.948	1.23	0.52	1.
Flavonifractor_plautii	All Ulcerative Colitis	1.51	1.19	0.628	1.
Frisingicoccus_SGB4674	All Crohn's Disease	0.902	0.251	0.291	1.
Frisingicoccus_SGB4674	All Ulcerative Colitis	7.47	0.414	7.54×10^{-03}	1.
Fusicatenibacter_saccharivorans	All Crohn's Disease	-0.0146	2.02	0.858	1.
Fusicatenibacter_saccharivorans	All Ulcerative Colitis	-3.74	2.05	0.0811	1.
Gemella_haemolysans	All Crohn's Disease	-2.52	0.753	0.121	1.
Gemella_haemolysans	All Ulcerative Colitis	1.2	0.718	0.835	1.
Gemella_morbillorum	All Crohn's Disease	3.98	3.62	0.501	1.
Gemella_morbillorum	All Ulcerative Colitis	3.18	2.95	0.689	1.
Gemella_sanguinis	All Crohn's Disease	0.187	1.46	0.565	1.
Gemella_sanguinis	All Ulcerative Colitis	2.32	1.51	0.543	1.
Gemmiger_formicilis	All Crohn's Disease	0.348	1.36	0.686	1.
GGB2980_SGB3962	All Crohn's Disease	1.5	3.03	0.488	1.
GGB2980_SGB3962	All Ulcerative Colitis	-0.0908	3.12	0.427	1.
GGB2982_SGB3964	All Crohn's Disease	0.726	0.248	0.602	1.
GGB3571_SGB4778	All Crohn's Disease	0.563	1.13	0.863	1.
GGB3571_SGB4778	All Ulcerative Colitis	-2.22	1.3	0.162	1.
GGB4456_SGB6141	All Crohn's Disease	2.13	3.79	0.878	1.
GGB4456_SGB6141	All Ulcerative Colitis	1.26	3.76	0.737	1.
GGB45432_SGB63101	All Crohn's Disease	3.06	0.799	0.0381	1.
GGB45432_SGB63101	All Ulcerative Colitis	3.2	0.952	0.097	1.
GGB51441_SGB71759	All Crohn's Disease	6.06	1.07	0.0656	1.
GGB51441_SGB71759	All Ulcerative Colitis	6.48	2.41	0.261	1.
GGB58158_SGB79798	All Crohn's Disease	-4.22	8.21	0.866	1.
GGB9581_SGB14999	All Crohn's Disease	-0.39	2.23	0.824	1.
GGB9581_SGB14999	All Ulcerative Colitis	1.22	2.39	0.773	1.
GGB9614_SGB15049	All Crohn's Disease	2.19	3.28	0.314	1.
GGB9614_SGB15049	All Ulcerative Colitis	3.19	2.89	0.512	1.
GGB9615_SGB15053	All Crohn's Disease	4.97	1.49	0.0418	1.
GGB9615_SGB15053	All Ulcerative Colitis	6.61	2.07	0.0593	1.
GGB9632_SGB15089	All Crohn's Disease	1.79	2.31	0.761	1.
GGB9632_SGB15089	All Ulcerative Colitis	1.94	3.1	0.573	1.
GGB9667_SGB15164	All Crohn's Disease	-2.07	2.13	0.709	1.
GGB9699_SGB15216	All Crohn's Disease	3.72	3.04	0.534	1.
GGB9707_SGB15229	All Crohn's Disease	1.78	2.06	0.736	1.
GGB9730_SGB15291	All Crohn's Disease	1.07	2.62	0.788	1.
GGB9730_SGB15291	All Ulcerative Colitis	-1.5	2.75	0.506	1.
Gordonibacter_pamelaeae	All Crohn's Disease	0.602	1.09	0.491	1.
Gordonibacter_pamelaeae	All Ulcerative Colitis	1.44	1.28	0.106	1.
Haemophilus_parainfluenzae	All Crohn's Disease	1.73	2.03	0.762	1.
Haemophilus_parainfluenzae	All Ulcerative Colitis	2.85	1.88	0.499	1.
Holdemania_filiformis	All Crohn's Disease	1.93	1.38	0.507	1.
Holdemania_filiformis	All Ulcerative Colitis	3.53	1.53	0.226	1.
Hungatella_hathewayi	All Crohn's Disease	1.55	2.72	0.524	1.
Hungatella_hathewayi	All Ulcerative Colitis	-0.171	2.85	0.938	1.
Hydrogeniiclostidium_mannosilyticum	All Crohn's Disease	6.2	3.71	0.381	1.
Intestinibacter_bartlettii	All Crohn's Disease	4.15	1.51	0.0413	1.
Intestinibacter_bartlettii	All Ulcerative Colitis	1.78	1.41	0.726	1.
Isoptericola_variabilis	All Crohn's Disease	-0.33	1.33	0.853	1.
Isoptericola_variabilis	All Ulcerative Colitis	0.395	1.3	0.955	1.
Lachnospira_eligens	All Crohn's Disease	-1.62	2.99	0.783	1.
Lachnospira_eligens	All Ulcerative Colitis	-2.34	2.86	0.539	1.
Lachnospira_sp_NSJ_43	All Crohn's Disease	2.34	1.69	0.526	1.
Lachnospira_sp_NSJ_43	All Ulcerative Colitis	1.39	1.78	0.401	1.
Lachnospiraceae_bacterium	All Crohn's Disease	2.12	1.8	0.559	1.
Lachnospiraceae_bacterium	All Ulcerative Colitis	0.0679	1.71	0.905	1.
Lachnospiraceae_bacterium_NSJ_29	All Crohn's Disease	-1.05	1.35	0.552	1.
Lachnospiraceae_bacterium_NSJ_29	All Ulcerative Colitis	-1.12	1.75	0.124	1.

Lachnospiraceae_bacterium_WCA3_601_WT_6H	All Crohn's Disease	2.19	2.63	0.772	1.
Lachnospiraceae_bacterium_WCA3_601_WT_6H	All Ulcerative Colitis	4.23	4.35	0.239	1.
Lachnospiraceae_unclassified_SGB4882	All Crohn's Disease	4.09	7.11	0.905	1.
Lachnospiraceae_unclassified_SGB4882	All Ulcerative Colitis	-0.656	4.06	0.718	1.
Lacrimispora_amygdalina	All Crohn's Disease	0.999	2.9	0.946	1.
Lacrimispora_amygdalina	All Ulcerative Colitis	-3.29	3.14	0.42	1.
Lacrimispora_celerecrescens	All Crohn's Disease	-1.23	2.97	0.843	1.
Lacrimispora_celerecrescens	All Ulcerative Colitis	-3.37	2.8	0.3	1.
Lacticaseibacillus_paracasei	All Crohn's Disease	6.32	2.76	0.183	1.
Lacticaseibacillus_paracasei	All Ulcerative Colitis	5.3	2.41	0.247	1.
Lactococcus_lactis	All Crohn's Disease	2.25	2.56	0.72	1.
Lactococcus_lactis	All Ulcerative Colitis	1.2	2.49	0.846	1.
Lancefieldella_parvula	All Crohn's Disease	0.109	2.37	0.715	1.
Lancefieldella_parvula	All Ulcerative Colitis	2.78	2.24	0.648	1.
Longibaculum_muris	All Crohn's Disease	1.37	1.69	0.734	1.
Longibaculum_muris	All Ulcerative Colitis	6.82	1.71	0.0469	1.
Longicatena_caecimuris	All Crohn's Disease	-2.09	1.89	0.395	1.
Longicatena_caecimuris	All Ulcerative Colitis	0.924	2.06	0.822	1.
Massilimaliae_massiliensis	All Crohn's Disease	6.06	2.8	0.324	1.
Massilimaliae_massiliensis	All Ulcerative Colitis	-0.587	3.1	0.732	1.
Massilimicrobiota_timonensis	All Crohn's Disease	0.89	3.26	0.969	1.
Massilimicrobiota_timonensis	All Ulcerative Colitis	-0.683	2.73	0.867	1.
Mediterraneibacter_butyricigenes	All Crohn's Disease	-1.12	0.695	0.169	1.
Mediterraneibacter_butyricigenes	All Ulcerative Colitis	-0.0871	0.817	0.367	1.
Mediterraneibacter_glycyrrhizinilyticus	All Crohn's Disease	-2.79	2.2	0.364	1.
Mediterraneibacter_glycyrrhizinilyticus	All Ulcerative Colitis	-0.184	2.43	0.572	1.
Merdimonas_faecis	All Crohn's Disease	3.17	3.08	0.777	1.
Merdimonas_faecis	All Ulcerative Colitis	2.42	2.66	0.874	1.
Mogibacterium_diversum	All Crohn's Disease	-0.053	0.841	0.407	1.
Mogibacterium_diversum	All Ulcerative Colitis	0.989	0.966	0.265	1.
Monoglobus_pectinilyticus	All Crohn's Disease	1.56	2.7	0.728	1.
Monoglobus_pectinilyticus	All Ulcerative Colitis	3.2	2.86	0.427	1.
Neobittarella_massiliensis	All Crohn's Disease	1.09	1.66	0.114	1.
Neobittarella_massiliensis	All Ulcerative Colitis	1.28	1.46	0.0968	1.
Odoribacter_splanchnicus	All Crohn's Disease	2.77	1.1	0.151	1.
Odoribacter_splanchnicus	All Ulcerative Colitis	2.21	1.2	0.477	1.
Oscillibacter_sp_ER4	All Crohn's Disease	-0.125	0.863	0.764	1.
Parabacteroides_distasonis	All Crohn's Disease	-0.792	1.71	0.708	1.
Parabacteroides_distasonis	All Ulcerative Colitis	-1.07	1.59	0.469	1.
Parabacteroides_merdae	All Crohn's Disease	0.997	1.92	0.929	1.
Parabacteroides_merdae	All Ulcerative Colitis	2.06	2.23	0.547	1.
Paraprevotella_clara	All Crohn's Disease	1.22	1.94	0.767	1.
Paraprevotella_clara	All Ulcerative Colitis	-0.165	1.59	0.889	1.
Parasutterella_excrementihominis	All Crohn's Disease	4.5	1.8	0.0917	1.
Parasutterella_excrementihominis	All Ulcerative Colitis	0.907	2.38	0.105	1.
Phocaeicola_vulgatus	All Crohn's Disease	1.66	2.03	0.548	1.
Phocaeicola_vulgatus	All Ulcerative Colitis	2.73	1.96	0.546	1.
Phocea_massiliensis	All Crohn's Disease	-0.719	3.	0.862	1.
Phocea_massiliensis	All Ulcerative Colitis	-0.528	2.77	0.885	1.
Romboutsia_timonensis	All Crohn's Disease	0.254	1.56	0.509	1.
Romboutsia_timonensis	All Ulcerative Colitis	-1.03	1.52	0.446	1.
Roseburia_faecis	All Crohn's Disease	-0.46	2.61	0.82	1.
Roseburia_faecis	All Ulcerative Colitis	-1.8	2.6	0.569	1.
Roseburia_hominis	All Crohn's Disease	1.06	3.18	0.313	1.
Roseburia_hominis	All Ulcerative Colitis	-0.888	3.21	0.856	1.
Roseburia_intestinalis	All Crohn's Disease	2.12	2.36	0.275	1.
Roseburia_intestinalis	All Ulcerative Colitis	0.0722	2.1	0.641	1.
Roseburia_inulinivorans	All Crohn's Disease	-0.59	2.44	0.914	1.
Roseburia_inulinivorans	All Ulcerative Colitis	1.35	2.5	0.688	1.
Roseburia_sp_AF02_12	All Crohn's Disease	-0.049	2.65	0.973	1.
Roseburia_sp_AF02_12	All Ulcerative Colitis	-4.83	1.44	0.117	1.
Ruminococcaceae_bacterium	All Crohn's Disease	0.424	1.6	0.806	1.

Ruminococcaceae_bacterium	All Ulcerative Colitis	2.46	1.77	0.587	1.
Ruminococcaceae_unclassified_SGB15236	All Crohn's Disease	6.62	1.7	0.308	1.
Ruminococcaceae_unclassified_SGB15265	All Crohn's Disease	6.62	4.59	0.427	1.
Ruminococcaceae_unclassified_SGB15265	All Ulcerative Colitis	4.86	4.41	0.644	1.
Ruminococcus_bicirculans	All Crohn's Disease	4.3	2.66	0.314	1.
Ruminococcus_bicirculans	All Ulcerative Colitis	-1.14	2.55	0.725	1.
Ruminococcus_bromii	All Crohn's Disease	4.55	3.32	0.407	1.
Ruminococcus_bromii	All Ulcerative Colitis	-2.17	3.49	0.503	1.
Ruminococcus_callidus	All Crohn's Disease	-4.72	4.85	0.607	1.
Ruminococcus_callidus	All Ulcerative Colitis	-10.4	5.07	0.219	1.
Ruminococcus_gnavus	All Crohn's Disease	-1.71	2.55	0.673	1.
Ruminococcus_gnavus	All Ulcerative Colitis	0.443	2.48	0.787	1.
Ruminococcus_lactaris	All Crohn's Disease	2.23	3.36	0.865	1.
Ruminococcus_lactaris	All Ulcerative Colitis	-1.06	4.96	0.817	1.
Ruminococcus_torques	All Crohn's Disease	-1.85	2.13	0.526	1.
Ruminococcus_torques	All Ulcerative Colitis	-3.01	2.11	0.169	1.
Ruthenibacterium_lactatiformans	All Crohn's Disease	-0.198	1.25	0.854	1.
Ruthenibacterium_lactatiformans	All Ulcerative Colitis	-0.903	1.43	0.263	1.
Sellimonas_intestinalis	All Crohn's Disease	-2.81	1.94	0.251	1.
Sellimonas_intestinalis	All Ulcerative Colitis	2.82	1.98	0.54	1.
Solobacterium_SGB6833	All Crohn's Disease	-1.57	0.913	0.134	1.
Solobacterium_SGB6833	All Ulcerative Colitis	0.0406	0.93	0.714	1.
Streptococcus_anginosus	All Crohn's Disease	-0.861	1.84	0.553	1.
Streptococcus_anginosus	All Ulcerative Colitis	0.68	1.79	0.687	1.
Streptococcus_gordonii	All Crohn's Disease	-2.68	1.62	0.18	1.
Streptococcus_gordonii	All Ulcerative Colitis	1.13	1.34	0.856	1.
Streptococcus_infantis	All Crohn's Disease	-0.488	1.37	0.801	1.
Streptococcus_infantis	All Ulcerative Colitis	-0.181	1.33	0.745	1.
Streptococcus_mitis	All Crohn's Disease	1.67	1.24	0.513	1.
Streptococcus_mitis	All Ulcerative Colitis	2.22	1.16	0.394	1.
Streptococcus_oralis	All Crohn's Disease	-0.347	2.31	0.913	1.
Streptococcus_oralis	All Ulcerative Colitis	-0.125	2.07	0.461	1.
Streptococcus_parasanguinis	All Crohn's Disease	-0.52	1.17	0.716	1.
Streptococcus_parasanguinis	All Ulcerative Colitis	0.533	1.14	0.917	1.
Streptococcus_salivarius	All Crohn's Disease	-0.0585	1.53	0.856	1.
Streptococcus_salivarius	All Ulcerative Colitis	0.832	1.47	0.967	1.
Streptococcus_sanguinis	All Crohn's Disease	3.57	1.03	0.013	1.
Streptococcus_sp_263_SSPC	All Crohn's Disease	-0.0977	1.33	0.934	1.
Streptococcus_sp_263_SSPC	All Ulcerative Colitis	0.317	1.24	0.562	1.
Streptococcus_sp_A12	All Crohn's Disease	-2.6	2.6	0.478	1.
Streptococcus_sp_A12	All Ulcerative Colitis	-2.22	2.68	0.308	1.
Streptococcus_thermophilus	All Crohn's Disease	-4.42	1.86	0.0348	1.
Streptococcus_thermophilus	All Ulcerative Colitis	-3.08	1.81	0.0901	1.
Sutterella_wadsworthensis	All Crohn's Disease	2.55	5.94	0.936	1.
Sutterella_wadsworthensis	All Ulcerative Colitis	-1.48	6.39	0.943	1.
Trueperella_pyogenes	All Crohn's Disease	-1.81	1.29	0.258	1.
Trueperella_pyogenes	All Ulcerative Colitis	-0.445	1.34	0.344	1.
Turicibacter_sanguinis	All Crohn's Disease	-0.945	1.93	0.765	1.
Turicibacter_sanguinis	All Ulcerative Colitis	-2.01	1.95	0.33	1.
Tyzzerella_nexilis	All Crohn's Disease	4.82	3.08	0.393	1.
Tyzzerella_nexilis	All Ulcerative Colitis	5.3	2.67	0.301	1.
Veillonella_atypica	All Crohn's Disease	4.03	6.81	0.86	1.
Veillonella_atypica	All Ulcerative Colitis	4.87	6.28	0.805	1.
Veillonella_dispar	All Crohn's Disease	-3.3	2.46	0.381	1.
Veillonella_dispar	All Ulcerative Colitis	-0.799	2.4	0.8	1.
Veillonella_parvula	All Crohn's Disease	-2.08	4.72	0.302	1.
Veillonella_parvula	All Ulcerative Colitis	-2.69	4.79	0.614	1.
Abiotrophia_defectiva	All Crohn's Disease	NA	NA	0.0755	1.
Abiotrophia_sp_HMSC24B09	All Crohn's Disease	NA	NA	0.659	1.
Abiotrophia_sp_HMSC24B09	All Ulcerative Colitis	NA	NA	0.294	1.
Achromobacter_xylosoxidans	All Crohn's Disease	NA	NA	0.656	1.
Achromobacter_xylosoxidans	All Ulcerative Colitis	NA	NA	0.674	1.

Acidaminococcus_intestini	All Crohn's Disease	NA	NA	0.697	1.
Acidaminococcus_intestini	All Ulcerative Colitis	NA	NA	0.757	1.
Actinomyces_bouchesdurhonensis	All Crohn's Disease	NA	NA	0.461	1.
Actinomyces_bouchesdurhonensis	All Ulcerative Colitis	NA	NA	0.675	1.
Actinomyces_dentalis	All Crohn's Disease	-1.94	NA	0.482	1.
Actinomyces_dentalis	All Ulcerative Colitis	-1.74	NA	0.446	1.
Actinomyces_johnsonii	All Crohn's Disease	NA	NA	0.243	1.
Actinomyces_sp_oral_taxon_448	All Crohn's Disease	3.64	NA	0.503	1.
Actinomyces_sp_oral_taxon_448	All Ulcerative Colitis	5.4	NA	0.472	1.
Akkermansia_muciniphila	All Crohn's Disease	15.6	NA	0.902	1.
Akkermansia_muciniphila	All Ulcerative Colitis	NA	NA	0.19	1.
Akkermansia_sp_KLE1605	All Crohn's Disease	NA	NA	0.469	1.
Akkermansia_sp_KLE1605	All Ulcerative Colitis	NA	NA	0.497	1.
Alistipes_finegoldii	All Ulcerative Colitis	NA	NA	0.0197	1.
Alistipes_indistinctus	All Crohn's Disease	NA	NA	0.0712	1.
Alistipes_indistinctus	All Ulcerative Colitis	-3.8	NA	0.128	1.
Alistipes_senegalensis	All Crohn's Disease	0.667	NA	0.488	1.
Alistipes_senegalensis	All Ulcerative Colitis	-0.893	NA	0.455	1.
Alistipes_sp_AF17_16	All Crohn's Disease	NA	NA	0.651	1.
Alistipes_sp_AF17_16	All Ulcerative Colitis	NA	NA	0.675	1.
Alistipes_timonensis	All Crohn's Disease	NA	NA	0.437	1.
Alistipes_timonensis	All Ulcerative Colitis	NA	NA	0.91	1.
Alloscardovia_omnicolens	All Crohn's Disease	NA	NA	0.624	1.
Alloscardovia_omnicolens	All Ulcerative Colitis	NA	NA	0.595	1.
Amedibacillus_dolichus	All Crohn's Disease	NA	NA	0.945	1.
Amedibacillus_dolichus	All Ulcerative Colitis	NA	NA	0.289	1.
Anaerococcus_obesiensis	All Crohn's Disease	NA	NA	0.442	1.
Anaerococcus_obesiensis	All Ulcerative Colitis	NA	NA	0.277	1.
Anaerosacchariphilus_sp_NSJ_68	All Crohn's Disease	-3.13	NA	0.577	1.
Anaerosacchariphilus_sp_NSJ_68	All Ulcerative Colitis	NA	NA	0.203	1.
Anaerotignum_lactatifermentans	All Crohn's Disease	3.28	NA	0.559	1.
Anaerotignum_lactatifermentans	All Ulcerative Colitis	NA	NA	0.227	1.
Anaerotruncus_rubiinfantis	All Crohn's Disease	NA	NA	0.962	1.
Anaerotruncus_rubiinfantis	All Ulcerative Colitis	NA	NA	0.508	1.
Atopobium_deltae	All Crohn's Disease	NA	NA	0.618	1.
Atopobium_deltae	All Ulcerative Colitis	NA	NA	0.842	1.
Bacilli_bacterium	All Crohn's Disease	NA	NA	0.664	1.
Bacilli_bacterium	All Ulcerative Colitis	NA	NA	0.678	1.
Bacteroidales_bacterium	All Crohn's Disease	-0.786	NA	0.575	1.
Bacteroidales_bacterium	All Ulcerative Colitis	NA	NA	0.206	1.
Bacteroides_cellulosilyticus	All Crohn's Disease	NA	NA	0.435	1.
Bacteroides_cellulosilyticus	All Ulcerative Colitis	NA	NA	0.803	1.
Bacteroides_eggerthii	All Crohn's Disease	NA	NA	0.264	1.
Bacteroides_faecis	All Crohn's Disease	NA	NA	0.457	1.
Bacteroides_faecis	All Ulcerative Colitis	NA	NA	0.986	1.
Bacteroides_finegoldii	All Crohn's Disease	NA	NA	0.614	1.
Bacteroides_finegoldii	All Ulcerative Colitis	NA	NA	0.883	1.
Bacteroides_intestinalis	All Crohn's Disease	NA	NA	0.647	1.
Bacteroides_intestinalis	All Ulcerative Colitis	NA	NA	0.506	1.
Bacteroides_nordii	All Crohn's Disease	NA	NA	0.972	1.
Bacteroides_nordii	All Ulcerative Colitis	NA	NA	0.681	1.
Bacteroides_salyersiae	All Crohn's Disease	NA	NA	0.448	1.
Bacteroides_salyersiae	All Ulcerative Colitis	NA	NA	0.983	1.
Bifidobacterium_adolescentis	All Crohn's Disease	NA	NA	0.244	1.
Bifidobacterium_adolescentis	All Ulcerative Colitis	NA	NA	0.263	1.
Bifidobacterium_animalis	All Crohn's Disease	NA	NA	0.239	1.
Bifidobacterium_breve	All Crohn's Disease	1.46	NA	0.561	1.
Bifidobacterium_breve	All Ulcerative Colitis	6.64	NA	0.414	1.
Bifidobacterium_catenulatum	All Crohn's Disease	NA	NA	0.65	1.
Bifidobacterium_catenulatum	All Ulcerative Colitis	NA	NA	0.676	1.
Blautia_argi	All Crohn's Disease	NA	NA	0.249	1.
Blautia_argi	All Ulcerative Colitis	0.51	NA	0.749	1.

Blautia_glucerasea	All Crohn's Disease	14.1	NA	0.869	1.
Blautia_glucerasea	All Ulcerative Colitis	3.84	NA	0.427	1.
Blautia_hansenii	All Crohn's Disease	NA	NA	0.703	1.
Blautia_hansenii	All Ulcerative Colitis	NA	NA	0.209	1.
Blautia_hominis	All Crohn's Disease	NA	NA	0.974	1.
Blautia_hominis	All Ulcerative Colitis	NA	NA	0.291	1.
Blautia_hydrogenotrophica	All Crohn's Disease	NA	NA	0.19	1.
Blautia_hydrogenotrophica	All Ulcerative Colitis	NA	NA	0.497	1.
Blautia_sp_AF19_10LB	All Crohn's Disease	NA	NA	0.234	1.
Blautia_sp_AF19_10LB	All Ulcerative Colitis	NA	NA	0.804	1.
Blautia_sp_MSK_20_85	All Crohn's Disease	-3.06	NA	0.851	1.
Blautia_sp_MSK_20_85	All Ulcerative Colitis	NA	NA	0.24	1.
Blautia_sp_MSK_21_1	All Crohn's Disease	-3.49	NA	0.565	1.
Blautia_sp_MSK_21_1	All Ulcerative Colitis	-8.33	NA	0.619	1.
Blautia_stercoris	All Crohn's Disease	NA	NA	0.337	1.
Blautia_stercoris	All Ulcerative Colitis	NA	NA	0.504	1.
Butyricicoccus_SGB14990	All Crohn's Disease	NA	NA	0.0795	1.
Butyricicoccus_SGB14990	All Ulcerative Colitis	NA	NA	0.0613	1.
Butyricicoccus_sp_AM29_23AC	All Crohn's Disease	NA	NA	0.311	1.
Butyricicoccus_sp_AM29_23AC	All Ulcerative Colitis	NA	NA	0.837	1.
Butyricimonas_SGB1783	All Crohn's Disease	NA	NA	0.614	1.
Butyricimonas_SGB1783	All Ulcerative Colitis	NA	NA	0.891	1.
Campylobacter_gracilis	All Crohn's Disease	NA	NA	0.993	1.
Campylobacter_gracilis	All Ulcerative Colitis	NA	NA	0.506	1.
Candidatus_Avimonas_narfia	All Crohn's Disease	NA	NA	0.985	1.
Candidatus_Avimonas_narfia	All Ulcerative Colitis	NA	NA	0.674	1.
Candidatus_Borkfalkia_ceftriaxoniphila	All Crohn's Disease	NA	NA	0.646	1.
Candidatus_Borkfalkia_ceftriaxoniphila	All Ulcerative Colitis	NA	NA	0.68	1.
Candidatus_Gastranaerophilales_bacterium	All Crohn's Disease	NA	NA	0.467	1.
Candidatus_Gastranaerophilales_bacterium	All Ulcerative Colitis	NA	NA	0.984	1.
Candidatus_Parachristensenella_avicola	All Crohn's Disease	NA	NA	0.984	1.
Candidatus_Parachristensenella_avicola	All Ulcerative Colitis	NA	NA	0.673	1.
Candidatus_Paralachnospira_caecorum	All Crohn's Disease	NA	NA	0.66	1.
Candidatus_Paralachnospira_caecorum	All Ulcerative Colitis	NA	NA	0.675	1.
Candidatus_Pararuminococcus_gallinarum	All Crohn's Disease	NA	NA	0.339	1.
Candidatus_Pararuminococcus_gallinarum	All Ulcerative Colitis	NA	NA	0.675	1.
Candidatus_Pseudoruminococcus_merdavium	All Crohn's Disease	NA	NA	0.677	1.
Candidatus_Pseudoruminococcus_merdavium	All Ulcerative Colitis	NA	NA	0.51	1.
Candidatus_Schneewindia_gallinarum	All Crohn's Disease	NA	NA	0.669	1.
Candidatus_Schneewindia_gallinarum	All Ulcerative Colitis	NA	NA	0.686	1.
Catabacter_hongkongensis	All Crohn's Disease	NA	NA	0.478	1.
Catabacter_hongkongensis	All Ulcerative Colitis	NA	NA	0.505	1.
Catenibacillus_scindens	All Crohn's Disease	-0.784	NA	0.538	1.
Catenibacillus_scindens	All Ulcerative Colitis	1.21	NA	0.472	1.
Christensenella_massiliensis	All Crohn's Disease	NA	NA	0.993	1.
Christensenella_massiliensis	All Ulcerative Colitis	NA	NA	0.974	1.
Citrobacter_freundii	All Crohn's Disease	NA	NA	0.688	1.
Citrobacter_freundii	All Ulcerative Colitis	NA	NA	0.729	1.
Clostridia_unclassified_SGB14844	All Crohn's Disease	NA	NA	0.486	1.
Clostridia_unclassified_SGB14844	All Ulcerative Colitis	NA	NA	0.52	1.
Clostridia_unclassified_SGB15402	All Crohn's Disease	NA	NA	0.669	1.
Clostridia_unclassified_SGB15402	All Ulcerative Colitis	NA	NA	0.685	1.
Clostridia_unclassified_SGB4367	All Crohn's Disease	-1.9	NA	0.524	1.
Clostridia_unclassified_SGB4367	All Ulcerative Colitis	-7.7	NA	0.746	1.
Clostridia_unclassified_SGB6276	All Crohn's Disease	NA	NA	0.472	1.
Clostridia_unclassified_SGB6276	All Ulcerative Colitis	NA	NA	0.992	1.
Clostridiaceae_bacterium_DONG20_135	All Crohn's Disease	NA	NA	0.638	1.
Clostridiaceae_bacterium_DONG20_135	All Ulcerative Colitis	NA	NA	0.702	1.
Clostridiaceae_bacterium_NSJ_33	All Crohn's Disease	NA	NA	0.953	1.
Clostridiaceae_bacterium_NSJ_33	All Ulcerative Colitis	NA	NA	0.52	1.
Clostridiales_bacterium	All Ulcerative Colitis	NA	NA	0.238	1.
Clostridiales_bacterium_1_7_47FAA	All Crohn's Disease	NA	NA	0.337	1.

Clostridiales_bacterium_1_7_47FAA	All Ulcerative Colitis	NA	NA	0.678	1.
Clostridiales_bacterium_Marseille_P5551	All Crohn's Disease	NA	NA	0.67	1.
Clostridiales_bacterium_Marseille_P5551	All Ulcerative Colitis	NA	NA	0.687	1.
Clostridiales_bacterium_NSJ_40	All Crohn's Disease	NA	NA	0.972	1.
Clostridiales_bacterium_NSJ_40	All Ulcerative Colitis	NA	NA	0.681	1.
Clostridiales_bacterium_UBA1390	All Crohn's Disease	NA	NA	0.669	1.
Clostridiales_bacterium_UBA1390	All Ulcerative Colitis	NA	NA	0.685	1.
Clostridiales_Family_XIII_Incertae_Sedis_unclassified_SGB3978	All Crohn's Disease	NA	NA	0.282	1.
Clostridiales_Family_XIII_Incertae_Sedis_unclassified_SGB3978	All Ulcerative Colitis	8.53	NA	0.316	1.
Clostridiales_unclassified_SGB15145	All Crohn's Disease	NA	NA	0.633	1.
Clostridiales_unclassified_SGB15145	All Ulcerative Colitis	NA	NA	0.724	1.
Clostridioides_difficile	All Crohn's Disease	NA	NA	0.649	1.
Clostridioides_difficile	All Ulcerative Colitis	NA	NA	0.677	1.
Clostridium_methylpentosum	All Crohn's Disease	NA	NA	0.344	1.
Clostridium_methylpentosum	All Ulcerative Colitis	NA	NA	0.984	1.
Clostridium_perfringens	All Crohn's Disease	NA	NA	0.468	1.
Clostridium_perfringens	All Ulcerative Colitis	NA	NA	0.497	1.
Clostridium_saccharogumia	All Ulcerative Colitis	NA	NA	0.226	1.
Clostridium_sp_AF15_49	All Crohn's Disease	NA	NA	0.272	1.
Clostridium_sp_AF15_49	All Ulcerative Colitis	-6.74	NA	0.427	1.
Clostridium_sp_AF27_2AA	All Crohn's Disease	NA	NA	0.454	1.
Clostridium_sp_AF27_2AA	All Ulcerative Colitis	NA	NA	0.689	1.
Clostridium_sp_AM49_4BH	All Crohn's Disease	NA	NA	0.256	1.
Clostridium_sp_AM49_4BH	All Ulcerative Colitis	NA	NA	0.983	1.
Clostridium_sp_Marseille_P3244	All Crohn's Disease	NA	NA	0.466	1.
Clostridium_sp_Marseille_P3244	All Ulcerative Colitis	NA	NA	0.674	1.
Clostridium_sp_SN20	All Crohn's Disease	1.03	NA	0.859	1.
Clostridium_sp_SN20	All Ulcerative Colitis	-2.08	NA	0.449	1.
Collinsella_intestinalis	All Crohn's Disease	NA	NA	0.965	1.
Collinsella_intestinalis	All Ulcerative Colitis	NA	NA	0.288	1.
Collinsella_tanakaei	All Crohn's Disease	NA	NA	0.985	1.
Collinsella_tanakaei	All Ulcerative Colitis	NA	NA	0.674	1.
Coprobacillus_cateniformis	All Crohn's Disease	7.38	NA	0.786	1.
Coprobacillus_cateniformis	All Ulcerative Colitis	16.3	NA	0.458	1.
Coprobacter_fastidiosus	All Crohn's Disease	NA	NA	0.62	1.
Coprobacter_fastidiosus	All Ulcerative Colitis	NA	NA	0.819	1.
Coprobacter_secundus	All Crohn's Disease	NA	NA	0.664	1.
Coprobacter_secundus	All Ulcerative Colitis	NA	NA	0.678	1.
Coprococcus_eutactus	All Crohn's Disease	NA	NA	0.261	1.
Coprococcus_eutactus	All Ulcerative Colitis	NA	NA	0.687	1.
Corynebacterium_argentoratense	All Crohn's Disease	NA	NA	0.649	1.
Corynebacterium_argentoratense	All Ulcerative Colitis	NA	NA	0.983	1.
Desulfovibrio_fairfieldensis	All Crohn's Disease	NA	NA	0.99	1.
Desulfovibrio_fairfieldensis	All Ulcerative Colitis	NA	NA	0.983	1.
Desulfovibrio_piger	All Crohn's Disease	1.31	NA	0.508	1.
Desulfovibrio_piger	All Ulcerative Colitis	NA	NA	0.243	1.
Dialister_pneumosintes	All Crohn's Disease	NA	NA	0.602	1.
Dialister_pneumosintes	All Ulcerative Colitis	NA	NA	0.9	1.
Dorea_phocaeensis	All Crohn's Disease	NA	NA	0.654	1.
Dorea_phocaeensis	All Ulcerative Colitis	NA	NA	0.982	1.
Eggerthella_timonensis	All Crohn's Disease	NA	NA	0.658	1.
Eggerthella_timonensis	All Ulcerative Colitis	NA	NA	0.984	1.
Eggerthellaceae_unclassified_SGB14322	All Crohn's Disease	NA	NA	0.65	1.
Eggerthellaceae_unclassified_SGB14322	All Ulcerative Colitis	NA	NA	0.676	1.
Eikenella_corrodens	All Crohn's Disease	NA	NA	0.619	1.
Eikenella_corrodens	All Ulcerative Colitis	NA	NA	0.825	1.
Eisenbergiella_sp_OF01_20	All Crohn's Disease	NA	NA	0.642	1.
Eisenbergiella_sp_OF01_20	All Ulcerative Colitis	NA	NA	0.994	1.
Eisenbergiella_tayi	All Crohn's Disease	NA	NA	0.14	1.
Eisenbergiella_tayi	All Ulcerative Colitis	NA	NA	0.679	1.
Emergencia_timonensis	All Crohn's Disease	NA	NA	0.669	1.
Emergencia_timonensis	All Ulcerative Colitis	NA	NA	0.686	1.

Enterocloster_citroniae	All Crohn's Disease	NA	NA	0.129	1.
Enterocloster_citroniae	All Ulcerative Colitis	NA	NA	0.345	1.
Enterococcus_avium	All Crohn's Disease	11.6	NA	0.415	1.
Enterococcus_avium	All Ulcerative Colitis	3.33	NA	0.559	1.
Enterococcus_faecium	All Crohn's Disease	NA	NA	0.985	1.
Enterococcus_faecium	All Ulcerative Colitis	NA	NA	0.299	1.
Enterococcus_gallinarum	All Crohn's Disease	NA	NA	0.656	1.
Enterococcus_gallinarum	All Ulcerative Colitis	NA	NA	0.498	1.
Enteroscipio_rubneri	All Crohn's Disease	NA	NA	0.669	1.
Enteroscipio_rubneri	All Ulcerative Colitis	NA	NA	0.686	1.
Erysipelatoclostridium_sp_An15	All Crohn's Disease	NA	NA	0.637	1.
Erysipelatoclostridium_sp_An15	All Ulcerative Colitis	NA	NA	0.992	1.
Erysipelotrichaceae_bacterium	All Crohn's Disease	NA	NA	0.977	1.
Erysipelotrichaceae_bacterium	All Ulcerative Colitis	NA	NA	0.676	1.
Eubacteriaceae_bacterium_CHKCI004	All Crohn's Disease	4.18	NA	0.541	1.
Eubacteriaceae_bacterium_CHKCI004	All Ulcerative Colitis	NA	NA	0.242	1.
Eubacterium_sp_AF34_35BH	All Crohn's Disease	NA	NA	0.341	1.
Eubacterium_sp_AF34_35BH	All Ulcerative Colitis	NA	NA	0.982	1.
Eubacterium_sp_AM28_29	All Crohn's Disease	-11.1	NA	0.518	1.
Eubacterium_sp_AM28_29	All Ulcerative Colitis	NA	NA	0.245	1.
Eubacterium_sp_An11	All Crohn's Disease	NA	NA	0.633	1.
Eubacterium_sp_An11	All Ulcerative Colitis	NA	NA	0.974	1.
Finegoldia_magna	All Crohn's Disease	NA	NA	0.975	1.
Finegoldia_magna	All Ulcerative Colitis	NA	NA	0.378	1.
Firmicutes_bacterium	All Crohn's Disease	NA	NA	0.474	1.
Firmicutes_bacterium	All Ulcerative Colitis	NA	NA	0.683	1.
Frisingicoccus_caecimuris	All Crohn's Disease	4.06	NA	0.509	1.
Frisingicoccus_caecimuris	All Ulcerative Colitis	3.61	NA	0.747	1.
Fusobacterium_nucleatum	All Crohn's Disease	NA	NA	0.63	1.
Fusobacterium_nucleatum	All Ulcerative Colitis	NA	NA	0.274	1.
Gemmiger_SGB15299	All Crohn's Disease	3.24	NA	0.518	1.
Gemmiger_SGB15299	All Ulcerative Colitis	NA	NA	0.245	1.
GGB12785_SGB19823	All Crohn's Disease	NA	NA	0.254	1.
GGB12785_SGB19823	All Ulcerative Colitis	-0.815	NA	0.746	1.
GGB2982_SGB3964	All Ulcerative Colitis	NA	NA	0.191	1.
GGB3005_SGB3996	All Crohn's Disease	NA	NA	0.461	1.
GGB3005_SGB3996	All Ulcerative Colitis	NA	NA	0.675	1.
GGB3034_SGB4030	All Crohn's Disease	NA	NA	0.661	1.
GGB3034_SGB4030	All Ulcerative Colitis	NA	NA	0.676	1.
GGB32463_SGB47515	All Crohn's Disease	-0.907	NA	0.559	1.
GGB32463_SGB47515	All Ulcerative Colitis	NA	NA	0.227	1.
GGB3256_SGB4303	All Crohn's Disease	-2.42	NA	0.496	1.
GGB3256_SGB4303	All Ulcerative Colitis	-0.962	NA	0.465	1.
GGB3293_SGB4348	All Crohn's Disease	NA	NA	0.655	1.
GGB3293_SGB4348	All Ulcerative Colitis	NA	NA	0.982	1.
GGB3433_SGB4573	All Crohn's Disease	NA	NA	0.674	1.
GGB3433_SGB4573	All Ulcerative Colitis	NA	NA	0.696	1.
GGB3463_SGB4621	All Crohn's Disease	NA	NA	0.445	1.
GGB3463_SGB4621	All Ulcerative Colitis	NA	NA	0.546	1.
GGB3478_SGB4643	All Crohn's Disease	NA	NA	0.442	1.
GGB3478_SGB4643	All Ulcerative Colitis	NA	NA	0.743	1.
GGB35068_SGB47850	All Crohn's Disease	NA	NA	0.261	1.
GGB35068_SGB47850	All Ulcerative Colitis	-1.56	NA	0.475	1.
GGB3523_SGB4703	All Crohn's Disease	NA	NA	0.466	1.
GGB3523_SGB4703	All Ulcerative Colitis	NA	NA	0.983	1.
GGB3537_SGB4727	All Crohn's Disease	NA	NA	0.661	1.
GGB3537_SGB4727	All Ulcerative Colitis	NA	NA	0.676	1.
GGB3570_SGB4777	All Crohn's Disease	NA	NA	0.336	1.
GGB3570_SGB4777	All Ulcerative Colitis	NA	NA	0.988	1.
GGB3583_SGB4799	All Crohn's Disease	NA	NA	0.457	1.
GGB3583_SGB4799	All Ulcerative Colitis	NA	NA	0.987	1.
GGB3606_SGB4870	All Crohn's Disease	NA	NA	0.674	1.

GGB3606_SGB4870	All Ulcerative Colitis	NA	NA	0.696	1.
GGB3614_SGB4886	All Crohn's Disease	NA	NA	0.664	1.
GGB3614_SGB4886	All Ulcerative Colitis	NA	NA	0.678	1.
GGB36472_SGB47660	All Crohn's Disease	NA	NA	0.67	1.
GGB36472_SGB47660	All Ulcerative Colitis	NA	NA	0.687	1.
GGB3677_SGB4990	All Crohn's Disease	NA	NA	0.669	1.
GGB3677_SGB4990	All Ulcerative Colitis	NA	NA	0.685	1.
GGB3746_SGB5089	All Crohn's Disease	NA	NA	0.136	1.
GGB3746_SGB5089	All Ulcerative Colitis	NA	NA	0.307	1.
GGB42689_SGB59892	All Crohn's Disease	NA	NA	0.993	1.
GGB42689_SGB59892	All Ulcerative Colitis	NA	NA	0.507	1.
GGB4482_SGB6176	All Crohn's Disease	NA	NA	0.472	1.
GGB4482_SGB6176	All Ulcerative Colitis	NA	NA	0.992	1.
GGB4491_SGB6188	All Crohn's Disease	NA	NA	0.457	1.
GGB4491_SGB6188	All Ulcerative Colitis	NA	NA	0.986	1.
GGB45491_SGB63163	All Crohn's Disease	NA	NA	0.659	1.
GGB45491_SGB63163	All Ulcerative Colitis	NA	NA	0.675	1.
GGB51510_SGB71883	All Crohn's Disease	NA	NA	0.633	1.
GGB51510_SGB71883	All Ulcerative Colitis	NA	NA	0.976	1.
GGB51959_SGB72479	All Crohn's Disease	-0.86	NA	0.533	1.
GGB51959_SGB72479	All Ulcerative Colitis	NA	NA	0.244	1.
GGB58158_SGB79798	All Ulcerative Colitis	NA	NA	0.232	1.
GGB58233_SGB79883	All Crohn's Disease	NA	NA	0.45	1.
GGB58233_SGB79883	All Ulcerative Colitis	NA	NA	0.7	1.
GGB6561_SGB9269	All Crohn's Disease	NA	NA	0.632	1.
GGB6561_SGB9269	All Ulcerative Colitis	NA	NA	0.548	1.
GGB9176_SGB14114	All Crohn's Disease	NA	NA	0.664	1.
GGB9176_SGB14114	All Ulcerative Colitis	NA	NA	0.678	1.
GGB9186_SGB14125	All Crohn's Disease	NA	NA	0.653	1.
GGB9186_SGB14125	All Ulcerative Colitis	NA	NA	0.499	1.
GGB9480_SGB14875	All Crohn's Disease	NA	NA	0.636	1.
GGB9480_SGB14875	All Ulcerative Colitis	NA	NA	0.532	1.
GGB9494_SGB14891	All Crohn's Disease	NA	NA	0.654	1.
GGB9494_SGB14891	All Ulcerative Colitis	NA	NA	0.674	1.
GGB9522_SGB14921	All Crohn's Disease	NA	NA	0.482	1.
GGB9522_SGB14921	All Ulcerative Colitis	NA	NA	0.701	1.
GGB9530_SGB14930	All Crohn's Disease	NA	NA	0.462	1.
GGB9530_SGB14930	All Ulcerative Colitis	NA	NA	0.982	1.
GGB9557_SGB14966	All Crohn's Disease	-0.331	NA	0.565	1.
GGB9557_SGB14966	All Ulcerative Colitis	-1.96	NA	0.4	1.
GGB9574_SGB14987	All Crohn's Disease	NA	NA	0.975	1.
GGB9574_SGB14987	All Ulcerative Colitis	NA	NA	0.678	1.
GGB9581_SGB79823	All Crohn's Disease	NA	NA	0.639	1.
GGB9581_SGB79823	All Ulcerative Colitis	NA	NA	0.698	1.
GGB9602_SGB15031	All Crohn's Disease	NA	NA	0.242	1.
GGB9602_SGB15031	All Ulcerative Colitis	3.9	NA	0.471	1.
GGB9608_SGB15041	All Crohn's Disease	2.64	NA	0.527	1.
GGB9608_SGB15041	All Ulcerative Colitis	-3.14	NA	0.481	1.
GGB9615_SGB15052	All Crohn's Disease	-0.139	NA	0.549	1.
GGB9615_SGB15052	All Ulcerative Colitis	NA	NA	0.237	1.
GGB9620_SGB15068	All Crohn's Disease	NA	NA	0.646	1.
GGB9620_SGB15068	All Ulcerative Colitis	NA	NA	0.681	1.
GGB9623_SGB15076	All Crohn's Disease	NA	NA	0.663	1.
GGB9623_SGB15076	All Ulcerative Colitis	NA	NA	0.678	1.
GGB9627_SGB15081	All Crohn's Disease	-6.36	NA	0.496	1.
GGB9627_SGB15081	All Ulcerative Colitis	-1.76	NA	0.465	1.
GGB9631_SGB15087	All Crohn's Disease	3.22	NA	0.518	1.
GGB9631_SGB15087	All Ulcerative Colitis	NA	NA	0.245	1.
GGB9633_SGB15090	All Crohn's Disease	-0.803	NA	0.542	1.
GGB9633_SGB15090	All Ulcerative Colitis	NA	NA	0.241	1.
GGB9633_SGB15091	All Crohn's Disease	NA	NA	0.483	1.
GGB9633_SGB15091	All Ulcerative Colitis	NA	NA	0.704	1.

GGB9635_SGB15106	All Crohn's Disease	6.34	NA	0.496	1.
GGB9635_SGB15106	All Ulcerative Colitis	7.65	NA	0.465	1.
GGB9640_SGB15115	All Crohn's Disease	-3.33	NA	0.518	1.
GGB9640_SGB15115	All Ulcerative Colitis	NA	NA	0.245	1.
GGB9642_SGB15119	All Crohn's Disease	-1.18	NA	0.518	1.
GGB9642_SGB15119	All Ulcerative Colitis	NA	NA	0.245	1.
GGB9646_SGB15123	All Crohn's Disease	NA	NA	0.438	1.
GGB9646_SGB15123	All Ulcerative Colitis	NA	NA	0.917	1.
GGB9667_SGB15164	All Ulcerative Colitis	NA	NA	0.216	1.
GGB9699_SGB15216	All Ulcerative Colitis	NA	NA	0.0614	1.
GGB9705_SGB15225	All Crohn's Disease	2.64	NA	0.797	1.
GGB9705_SGB15225	All Ulcerative Colitis	NA	NA	0.24	1.
GGB9707_SGB15229	All Ulcerative Colitis	NA	NA	0.0617	1.
GGB9712_SGB15244	All Crohn's Disease	1.37	NA	0.496	1.
GGB9712_SGB15244	All Ulcerative Colitis	1.83	NA	0.465	1.
GGB9758_SGB15368	All Crohn's Disease	5.31	NA	0.508	1.
GGB9758_SGB15368	All Ulcerative Colitis	NA	NA	0.243	1.
Gordonibacter_urolithinfaciens	All Crohn's Disease	NA	NA	0.346	1.
Gordonibacter_urolithinfaciens	All Ulcerative Colitis	NA	NA	0.676	1.
Granulicatella_adiacens	All Crohn's Disease	NA	NA	0.657	1.
Granulicatella_adiacens	All Ulcerative Colitis	NA	NA	0.295	1.
Granulicatella_elegans	All Crohn's Disease	NA	NA	0.94	1.
Granulicatella_elegans	All Ulcerative Colitis	NA	NA	0.543	1.
Holdemania_massiliensis	All Crohn's Disease	5.33	NA	0.841	1.
Holdemania_massiliensis	All Ulcerative Colitis	NA	NA	0.243	1.
Holdemania_sp_Marseille_P2844	All Crohn's Disease	NA	NA	0.671	1.
Holdemania_sp_Marseille_P2844	All Ulcerative Colitis	NA	NA	0.379	1.
Hydrogeniiclostidium_mannosilyticum	All Ulcerative Colitis	NA	NA	0.244	1.
Intestinibacter_SGB6139	All Crohn's Disease	NA	NA	0.26	1.
Intestinibacter_SGB6139	All Ulcerative Colitis	NA	NA	0.681	1.
Intestinimonas_butyriciproducens	All Crohn's Disease	NA	NA	0.452	1.
Intestinimonas_butyriciproducens	All Ulcerative Colitis	NA	NA	0.999	1.
Intestinimonas_gabonensis	All Crohn's Disease	NA	NA	0.0795	1.
Intestinimonas_gabonensis	All Ulcerative Colitis	NA	NA	0.0613	1.
Intestinimonas_massiliensis	All Crohn's Disease	NA	NA	0.45	1.
Intestinimonas_massiliensis	All Ulcerative Colitis	NA	NA	0.701	1.
Klebsiella_pneumoniae	All Crohn's Disease	NA	NA	0.983	1.
Klebsiella_pneumoniae	All Ulcerative Colitis	NA	NA	0.805	1.
Kytococcus_sedentarius	All Crohn's Disease	-2.41	NA	0.535	1.
Kytococcus_sedentarius	All Ulcerative Colitis	-3.51	NA	0.732	1.
Lachnoclostridium_edouardi	All Crohn's Disease	NA	NA	0.317	1.
Lachnoclostridium_edouardi	All Ulcerative Colitis	NA	NA	0.889	1.
Lachnoclostridium_phocaeense	All Crohn's Disease	NA	NA	0.659	1.
Lachnoclostridium_phocaeense	All Ulcerative Colitis	NA	NA	0.497	1.
Lachnoclostridium_sp_An118	All Crohn's Disease	NA	NA	0.659	1.
Lachnoclostridium_sp_An118	All Ulcerative Colitis	NA	NA	0.379	1.
Lachnoclostridium_sp_An138	All Crohn's Disease	-2.67	NA	0.54	1.
Lachnoclostridium_sp_An138	All Ulcerative Colitis	-0.112	NA	0.469	1.
Lachnospira_pectinoschiza	All Crohn's Disease	NA	NA	0.972	1.
Lachnospira_pectinoschiza	All Ulcerative Colitis	NA	NA	0.224	1.
Lachnospira_SGB5077	All Crohn's Disease	NA	NA	0.34	1.
Lachnospira_SGB5077	All Ulcerative Colitis	NA	NA	0.674	1.
Lachnospiraceae_bacterium_OF09_6	All Crohn's Disease	-4.21	NA	0.519	1.
Lachnospiraceae_bacterium_OF09_6	All Ulcerative Colitis	NA	NA	0.245	1.
Lachnospiraceae_bacterium_OM04_12BH	All Crohn's Disease	2.57	NA	0.555	1.
Lachnospiraceae_bacterium_OM04_12BH	All Ulcerative Colitis	NA	NA	0.231	1.
Lachnospiraceae_unclassified_SGB66069	All Crohn's Disease	NA	NA	0.992	1.
Lachnospiraceae_unclassified_SGB66069	All Ulcerative Colitis	NA	NA	0.679	1.
Lacrimispora_saccharolytica	All Crohn's Disease	NA	NA	0.453	1.
Lacrimispora_saccharolytica	All Ulcerative Colitis	NA	NA	0.996	1.
Lacticaseibacillus_rhamnosus	All Crohn's Disease	NA	NA	0.456	1.
Lacticaseibacillus_rhamnosus	All Ulcerative Colitis	NA	NA	0.311	1.

Lactobacillus_acidophilus	All Crohn's Disease	NA	NA	0.974	1.
Lactobacillus_acidophilus	All Ulcerative Colitis	NA	NA	0.498	1.
Lactobacillus_gasseri	All Crohn's Disease	NA	NA	0.639	1.
Lactobacillus_gasseri	All Ulcerative Colitis	NA	NA	0.405	1.
Lactococcus_piscium	All Crohn's Disease	NA	NA	0.951	1.
Lactococcus_piscium	All Ulcerative Colitis	NA	NA	0.523	1.
Lactonifactor_sp_BIOML_A6	All Crohn's Disease	-1.13	NA	0.541	1.
Lactonifactor_sp_BIOML_A6	All Ulcerative Colitis	-2.57	NA	0.468	1.
Lancefieldella_rimae	All Crohn's Disease	NA	NA	0.998	1.
Lancefieldella_rimae	All Ulcerative Colitis	NA	NA	0.517	1.
Lawsonibacter_sp_NSJ_51	All Crohn's Disease	NA	NA	0.985	1.
Lawsonibacter_sp_NSJ_51	All Ulcerative Colitis	NA	NA	0.674	1.
Lawsonibacter_sp_NSJ_52	All Crohn's Disease	NA	NA	0.442	1.
Lawsonibacter_sp_NSJ_52	All Ulcerative Colitis	NA	NA	0.951	1.
Leuconostoc_mesenteroides	All Crohn's Disease	NA	NA	0.953	1.
Leuconostoc_mesenteroides	All Ulcerative Colitis	NA	NA	0.52	1.
Limosilactobacillus_fermentum	All Crohn's Disease	NA	NA	0.339	1.
Limosilactobacillus_fermentum	All Ulcerative Colitis	NA	NA	0.982	1.
Marvinbryantia_SGB4691	All Crohn's Disease	NA	NA	0.975	1.
Marvinbryantia_SGB4691	All Ulcerative Colitis	NA	NA	0.678	1.
Massilicoli_timonensis	All Crohn's Disease	NA	NA	0.647	1.
Massilicoli_timonensis	All Ulcerative Colitis	NA	NA	0.985	1.
Massilimaliae_timonensis	All Crohn's Disease	NA	NA	0.64	1.
Massilimaliae_timonensis	All Ulcerative Colitis	NA	NA	0.695	1.
Massilimicrobiota_sp_An134	All Crohn's Disease	NA	NA	0.657	1.
Massilimicrobiota_sp_An134	All Ulcerative Colitis	NA	NA	0.674	1.
Massilioclostridium_coli	All Crohn's Disease	NA	NA	0.257	1.
Massilioclostridium_coli	All Ulcerative Colitis	5.69	NA	0.741	1.
Massilistercora_timonensis	All Crohn's Disease	NA	NA	0.66	1.
Massilistercora_timonensis	All Ulcerative Colitis	NA	NA	0.675	1.
Mediterraneibacter_massiliensis	All Crohn's Disease	NA	NA	0.65	1.
Mediterraneibacter_massiliensis	All Ulcerative Colitis	NA	NA	0.676	1.
Mediterraneibacter_sp_gm002	All Crohn's Disease	-2.7	NA	0.496	1.
Mediterraneibacter_sp_gm002	All Ulcerative Colitis	-3.8	NA	0.465	1.
Megasphaera_micronuciformis	All Crohn's Disease	NA	NA	0.953	1.
Megasphaera_micronuciformis	All Ulcerative Colitis	NA	NA	0.52	1.
Morganella_morganii	All Crohn's Disease	8.82	NA	0.453	1.
Morganella_morganii	All Ulcerative Colitis	3.79	NA	0.397	1.
Murimonas_intestini	All Crohn's Disease	NA	NA	0.451	1.
Murimonas_intestini	All Ulcerative Colitis	NA	NA	0.999	1.
Negativibacillus_massiliensis	All Crohn's Disease	NA	NA	0.264	1.
Negativibacillus_massiliensis	All Ulcerative Colitis	NA	NA	0.979	1.
Neglecta_timonensis	All Crohn's Disease	NA	NA	0.657	1.
Neglecta_timonensis	All Ulcerative Colitis	NA	NA	0.674	1.
Oscillibacter_sp_ER4	All Ulcerative Colitis	NA	NA	0.0601	1.
Parabacteroides_goldsteinii	All Crohn's Disease	NA	NA	0.281	1.
Parabacteroides_goldsteinii	All Ulcerative Colitis	-7.86	NA	0.523	1.
Parasutterella_SGB9260	All Crohn's Disease	NA	NA	0.284	1.
Parasutterella_SGB9260	All Ulcerative Colitis	4.65	NA	0.289	1.
Parvimonas_micra	All Crohn's Disease	NA	NA	0.636	1.
Parvimonas_micra	All Ulcerative Colitis	NA	NA	0.415	1.
Parvimonas_sp_KA00067	All Crohn's Disease	NA	NA	0.425	1.
Parvimonas_sp_KA00067	All Ulcerative Colitis	NA	NA	0.906	1.
Pauljensenia_hongkongensis	All Crohn's Disease	NA	NA	0.681	1.
Pauljensenia_hongkongensis	All Ulcerative Colitis	NA	NA	0.711	1.
Pediococcus_acidilactici	All Crohn's Disease	NA	NA	1.	1.
Pediococcus_acidilactici	All Ulcerative Colitis	NA	NA	0.32	1.
Peptoniphilus_coxii	All Crohn's Disease	NA	NA	0.272	1.
Peptoniphilus_coxii	All Ulcerative Colitis	0.322	NA	0.427	1.
Peptoniphilus_harei	All Crohn's Disease	NA	NA	0.972	1.
Peptoniphilus_harei	All Ulcerative Colitis	NA	NA	0.29	1.
Peptostreptococcus_anaerobius	All Crohn's Disease	NA	NA	0.618	1.

Peptostreptococcus_anaerobius	All Ulcerative Colitis	NA	NA	0.842	1.
Peptostreptococcus_SGB749	All Crohn's Disease	NA	NA	0.94	1.
Peptostreptococcus_SGB749	All Ulcerative Colitis	NA	NA	0.543	1.
Peptostreptococcus_stomatis	All Crohn's Disease	NA	NA	0.637	1.
Peptostreptococcus_stomatis	All Ulcerative Colitis	NA	NA	0.326	1.
Phascolarctobacterium_faecium	All Crohn's Disease	NA	NA	0.623	1.
Phascolarctobacterium_faecium	All Ulcerative Colitis	NA	NA	0.468	1.
Phascolarctobacterium_succinatutens	All Crohn's Disease	1.86	NA	0.847	1.
Phascolarctobacterium_succinatutens	All Ulcerative Colitis	NA	NA	0.242	1.
Phocaeicola_dorei	All Crohn's Disease	NA	NA	0.343	1.
Phocaeicola_dorei	All Ulcerative Colitis	NA	NA	0.295	1.
Phocaeicola_massiliensis	All Crohn's Disease	NA	NA	0.346	1.
Phocaeicola_massiliensis	All Ulcerative Colitis	NA	NA	0.292	1.
Prevotella_buccae	All Crohn's Disease	NA	NA	0.674	1.
Prevotella_buccae	All Ulcerative Colitis	NA	NA	0.696	1.
Proteus_mirabilis	All Crohn's Disease	NA	NA	0.906	1.
Proteus_mirabilis	All Ulcerative Colitis	NA	NA	0.439	1.
Pseudoflavonifractor_capillosus	All Crohn's Disease	NA	NA	0.615	1.
Pseudoflavonifractor_capillosus	All Ulcerative Colitis	NA	NA	0.878	1.
Pseudoflavonifractor_SGB15156	All Crohn's Disease	NA	NA	0.465	1.
Pseudoflavonifractor_SGB15156	All Ulcerative Colitis	NA	NA	0.498	1.
Pseudoruminococcus_massiliensis	All Crohn's Disease	NA	NA	0.977	1.
Pseudoruminococcus_massiliensis	All Ulcerative Colitis	NA	NA	0.676	1.
Rothia_dentocariosa	All Crohn's Disease	NA	NA	0.99	1.
Rothia_dentocariosa	All Ulcerative Colitis	NA	NA	0.387	1.
Rothia_mucilaginosa	All Crohn's Disease	NA	NA	0.252	1.
Rothia_mucilaginosa	All Ulcerative Colitis	NA	NA	0.149	1.
Ruminococcaceae_unclassified_SGB15234	All Crohn's Disease	NA	NA	0.675	1.
Ruminococcaceae_unclassified_SGB15234	All Ulcerative Colitis	NA	NA	0.696	1.
Ruminococcaceae_unclassified_SGB15236	All Ulcerative Colitis	NA	NA	0.245	1.
Ruminococcaceae_unclassified_SGB4191	All Crohn's Disease	NA	NA	0.629	1.
Ruminococcaceae_unclassified_SGB4191	All Ulcerative Colitis	NA	NA	0.748	1.
Ruminococcus_SGB4421	All Crohn's Disease	NA	NA	0.647	1.
Ruminococcus_SGB4421	All Ulcerative Colitis	NA	NA	0.985	1.
Ruminococcus_sp_AF41_9	All Crohn's Disease	NA	NA	0.269	1.
Ruminococcus_sp_AF41_9	All Ulcerative Colitis	4.11	NA	0.447	1.
Ruminococcus_sp_NSJ_71	All Crohn's Disease	NA	NA	0.643	1.
Ruminococcus_sp_NSJ_71	All Ulcerative Colitis	NA	NA	0.397	1.
Scardovia_wiggsiae	All Crohn's Disease	8.1	NA	0.538	1.
Scardovia_wiggsiae	All Ulcerative Colitis	6.38	NA	0.727	1.
Schaalia_turicensis	All Crohn's Disease	NA	NA	0.411	1.
Schaalia_turicensis	All Ulcerative Colitis	NA	NA	0.871	1.
Slackia_exigua	All Crohn's Disease	NA	NA	0.649	1.
Slackia_exigua	All Ulcerative Colitis	NA	NA	0.677	1.
Slackia_isoflavoniconvertens	All Crohn's Disease	NA	NA	0.265	1.
Slackia_isoflavoniconvertens	All Ulcerative Colitis	-1.44	NA	0.714	1.
Slackia_piriformis	All Crohn's Disease	-1.35	NA	0.146	1.
Slackia_piriformis	All Ulcerative Colitis	-1.37	NA	0.128	1.
Staphylococcus_aureus	All Crohn's Disease	NA	NA	0.649	1.
Staphylococcus_aureus	All Ulcerative Colitis	NA	NA	0.503	1.
Streptococcus_constellatus	All Crohn's Disease	NA	NA	0.974	1.
Streptococcus_constellatus	All Ulcerative Colitis	NA	NA	0.733	1.
Streptococcus_cristatus	All Crohn's Disease	0.831	NA	0.548	1.
Streptococcus_cristatus	All Ulcerative Colitis	1.93	NA	0.698	1.
Streptococcus_intermedius	All Crohn's Disease	5.68	NA	0.814	1.
Streptococcus_intermedius	All Ulcerative Colitis	22.1	NA	0.48	1.
Streptococcus_lutetiensis	All Crohn's Disease	NA	NA	0.646	1.
Streptococcus_lutetiensis	All Ulcerative Colitis	NA	NA	0.391	1.
Streptococcus_mutans	All Crohn's Disease	NA	NA	0.457	1.
Streptococcus_mutans	All Ulcerative Colitis	NA	NA	0.508	1.
Streptococcus_pneumoniae	All Crohn's Disease	NA	NA	0.642	1.
Streptococcus_pneumoniae	All Ulcerative Colitis	NA	NA	0.689	1.

Streptococcus_rubneri	All Crohn's Disease	NA	NA	0.646	1.
Streptococcus_rubneri	All Ulcerative Colitis	NA	NA	0.68	1.
TM7_phylum_sp_oral_taxon_348	All Crohn's Disease	5.33	NA	0.78	1.
TM7_phylum_sp_oral_taxon_348	All Ulcerative Colitis	214.	NA	0.451	1.
Veillonella_rogosae	All Crohn's Disease	NA	NA	0.625	1.
Veillonella_rogosae	All Ulcerative Colitis	NA	NA	0.769	1.
