# Supplementary material for: Multiomics-Based Profiling of the Fecal Microbiome Reveals Potential Disease-Specific Signatures in Pediatric IBD (PIBD)
Source: Biomolecules. 2025 May 21;15(5):746. doi: 10.3390/biom15050746 (PMC12109367; doi:10.3390/biom15050746)
Supplement: Supplementary file 1 [file biomolecules-15-00746-s001.zip › supplemental7-maaslin-diseasegroup-taxaprevalence.pdf]

Supplemental Table S7

Differential taxonomic prevalence in UC and Crohn's as compared to healthy microbiome, calculated using MaAsLin3.

Pathway	Condition	Effect Size	Standard Error	P-Value	Q-Value
Gemmiger_formicilis	All Ulcerative Colitis	-3.09	2.07	1.23×10^{-03}	0.573
Streptococcus_sanguinis	All Ulcerative Colitis	0.378	1.31	2.16×10^{-03}	0.67
Abiotrophia_defectiva	All Crohn's Disease	-2.8	1.58	0.0755	1.
Abiotrophia_defectiva	All Ulcerative Colitis	-0.646	1.1	0.612	1.
Abiotrophia_sp_HMSC24B09	All Crohn's Disease	0.956	2.17	0.659	1.
Abiotrophia_sp_HMSC24B09	All Ulcerative Colitis	2.18	2.08	0.294	1.
Achromobacter_xylosoxidans	All Crohn's Disease	0.965	2.17	0.656	1.
Achromobacter_xylosoxidans	All Ulcerative Colitis	0.905	2.15	0.674	1.
Acidaminococcus_intestini	All Crohn's Disease	0.85	2.18	0.697	1.
Acidaminococcus_intestini	All Ulcerative Colitis	0.687	2.22	0.757	1.
Actinobaculum_sp_oral_taxon_183	All Crohn's Disease	-0.568	1.16	0.206	1.
Actinobaculum_sp_oral_taxon_183	All Ulcerative Colitis	-0.647	1.18	0.578	1.
Actinomyces_bouchesdurhonensis	All Crohn's Disease	1.56	2.12	0.461	1.
Actinomyces_bouchesdurhonensis	All Ulcerative Colitis	0.9	2.15	0.675	1.
Actinomyces_dentalis	All Crohn's Disease	-0.957	1.36	0.482	1.
Actinomyces_dentalis	All Ulcerative Colitis	-1.05	1.38	0.446	1.
Actinomyces_gerencseriae	All Crohn's Disease	-1.22	1.18	0.51	1.
Actinomyces_gerencseriae	All Ulcerative Colitis	-1.87	1.23	0.237	1.
Actinomyces_graevenitzii	All Crohn's Disease	0.246	1.14	0.165	1.
Actinomyces_graevenitzii	All Ulcerative Colitis	0.158	1.14	0.317	1.
Actinomyces_johnsonii	All Crohn's Disease	-1.93	1.65	0.243	1.
Actinomyces_johnsonii	All Ulcerative Colitis	0.0104	1.22	0.991	1.
Actinomyces_massiliensis	All Crohn's Disease	-1.	1.37	0.575	1.
Actinomyces_massiliensis	All Ulcerative Colitis	-0.06	1.27	0.999	1.
Actinomyces_naeslundii	All Crohn's Disease	-1.16	1.24	0.576	1.
Actinomyces_naeslundii	All Ulcerative Colitis	-1.2	1.25	0.563	1.
Actinomyces_oris	All Crohn's Disease	0.735	1.38	0.309	1.
Actinomyces_oris	All Ulcerative Colitis	0.151	1.32	0.85	1.
Actinomyces_SGB17154	All Crohn's Disease	-1.89	1.28	0.259	1.
Actinomyces_SGB17154	All Ulcerative Colitis	-1.39	1.18	0.421	1.
Actinomyces_SGB17168	All Crohn's Disease	-0.0679	1.12	0.944	1.
Actinomyces_SGB17168	All Ulcerative Colitis	-0.59	1.11	0.818	1.
Actinomyces_sp_ICM47	All Crohn's Disease	-3.01	2.1	0.283	1.
Actinomyces_sp_ICM47	All Ulcerative Colitis	-2.66	2.1	0.369	1.
Actinomyces_sp_ICM58	All Crohn's Disease	-1.39	1.22	0.443	1.
Actinomyces_sp_ICM58	All Ulcerative Colitis	-2.32	1.26	0.125	1.
Actinomyces_sp_oral_taxon_448	All Crohn's Disease	-0.906	1.35	0.503	1.
Actinomyces_sp_oral_taxon_448	All Ulcerative Colitis	-0.96	1.34	0.472	1.
Actinomyces_sp_S6_Spd3	All Crohn's Disease	-0.466	1.13	0.898	1.
Actinomyces_sp_S6_Spd3	All Ulcerative Colitis	-0.963	1.14	0.64	1.
Adlercreutzia_equolifaciens	All Crohn's Disease	-3.95×10^{-03}	1.11	1.	1.
Adlercreutzia_equolifaciens	All Ulcerative Colitis	-0.696	1.1	0.776	1.
Agathobaculum_butyriciproducens	All Crohn's Disease	-2.34	2.09	0.451	1.
Agathobaculum_butyriciproducens	All Ulcerative Colitis	-3.58	2.11	0.0779	1.
Akkermansia_muciniphila	All Crohn's Disease	-0.157	1.27	0.902	1.
Akkermansia_muciniphila	All Ulcerative Colitis	-2.38	1.81	0.19	1.
Akkermansia_sp_KLE1605	All Crohn's Disease	1.53	2.12	0.469	1.
Akkermansia_sp_KLE1605	All Ulcerative Colitis	1.43	2.11	0.497	1.
Alistipes_communis	All Crohn's Disease	-0.243	1.26	0.524	1.
Alistipes_communis	All Ulcerative Colitis	-0.0567	1.21	0.234	1.
Alistipes_finegoldii	All Crohn's Disease	-0.501	1.22	0.762	1.
Alistipes_finegoldii	All Ulcerative Colitis	-4.35	1.86	0.0197	1.
Alistipes_ihumii	All Crohn's Disease	-0.807	1.34	0.755	1.
Alistipes_ihumii	All Ulcerative Colitis	-0.483	1.25	0.672	1.
Alistipes_indistinctus	All Crohn's Disease	-2.86	1.58	0.0712	1.
Alistipes_indistinctus	All Ulcerative Colitis	-1.89	1.24	0.128	1.
Alistipes_onderdonkii	All Crohn's Disease	-0.882	1.21	0.465	1.
Alistipes_onderdonkii	All Ulcerative Colitis	-2.32	1.28	0.134	1.

Alistipes_putredinis	All Crohn's Disease	0.401	1.12	0.382	1.
Alistipes_putredinis	All Ulcerative Colitis	-0.547	1.1	0.592	1.
Alistipes_senegalensis	All Crohn's Disease	-0.943	1.36	0.488	1.
Alistipes_senegalensis	All Ulcerative Colitis	-1.02	1.37	0.455	1.
Alistipes_shahii	All Crohn's Disease	-1.25	1.21	0.334	1.
Alistipes_shahii	All Ulcerative Colitis	-1.9	1.23	0.229	1.
Alistipes_sp_AF17_16	All Crohn's Disease	0.981	2.17	0.651	1.
Alistipes_sp_AF17_16	All Ulcerative Colitis	0.9	2.15	0.675	1.
Alistipes_timonensis	All Crohn's Disease	1.65	2.12	0.437	1.
Alistipes_timonensis	All Ulcerative Colitis	-0.269	2.39	0.91	1.
Alloscardovia_omnicolens	All Crohn's Disease	1.06	2.17	0.624	1.
Alloscardovia_omnicolens	All Ulcerative Colitis	1.14	2.14	0.595	1.
Amedibacillus_dolichus	All Crohn's Disease	-0.165	2.37	0.945	1.
Amedibacillus_dolichus	All Ulcerative Colitis	2.23	2.1	0.289	1.
Anaerobutyricum_hallii	All Crohn's Disease	0.221	1.26	0.636	1.
Anaerobutyricum_hallii	All Ulcerative Colitis	-0.505	1.18	0.708	1.
Anaerococcus_obesiensis	All Crohn's Disease	1.63	2.12	0.442	1.
Anaerococcus_obesiensis	All Ulcerative Colitis	2.26	2.08	0.277	1.
Anaerofustis_stercorihominis	All Crohn's Disease	-0.414	1.12	0.532	1.
Anaerofustis_stercorihominis	All Ulcerative Colitis	-0.602	1.1	0.765	1.
Anaeromassilibacillus_sp_An250	All Crohn's Disease	0.856	1.22	0.732	1.
Anaeromassilibacillus_sp_An250	All Ulcerative Colitis	0.746	1.21	0.788	1.
Anaerosacchariphilus_sp_NSJ_68	All Crohn's Disease	-0.753	1.35	0.577	1.
Anaerosacchariphilus_sp_NSJ_68	All Ulcerative Colitis	-2.27	1.78	0.203	1.
Anaerostipes_caccae	All Crohn's Disease	-1.66	1.23	0.318	1.
Anaerostipes_caccae	All Ulcerative Colitis	-1.15	1.18	0.551	1.
Anaerostipes_hadrus	All Crohn's Disease	-1.04	2.17	0.606	1.
Anaerostipes_hadrus	All Ulcerative Colitis	-1.66	2.09	0.674	1.
Anaerotignum_faecicola	All Crohn's Disease	0.654	1.22	0.832	1.
Anaerotignum_faecicola	All Ulcerative Colitis	-0.745	1.31	0.814	1.
Anaerotignum_lactatifermentans	All Crohn's Disease	-0.785	1.34	0.559	1.
Anaerotignum_lactatifermentans	All Ulcerative Colitis	-1.94	1.6	0.227	1.
Anaerotruncus_colihominis	All Crohn's Disease	-1.26	1.18	0.192	1.
Anaerotruncus_colihominis	All Ulcerative Colitis	-1.36	1.16	0.427	1.
Anaerotruncus_rubiinfantis	All Crohn's Disease	-0.113	2.37	0.962	1.
Anaerotruncus_rubiinfantis	All Ulcerative Colitis	1.4	2.12	0.508	1.
Atopobium_deltae	All Crohn's Disease	1.08	2.17	0.618	1.
Atopobium_deltae	All Ulcerative Colitis	0.445	2.24	0.842	1.
Bacilli_bacterium	All Crohn's Disease	0.943	2.17	0.664	1.
Bacilli_bacterium	All Ulcerative Colitis	0.893	2.15	0.678	1.
Bacteroidales_bacterium	All Crohn's Disease	-0.757	1.35	0.575	1.
Bacteroidales_bacterium	All Ulcerative Colitis	-2.21	1.75	0.206	1.
Bacteroides_caccae	All Crohn's Disease	-0.296	1.12	0.596	1.
Bacteroides_caccae	All Ulcerative Colitis	-0.333	1.1	0.911	1.
Bacteroides_cellulosilyticus	All Crohn's Disease	1.65	2.12	0.435	1.
Bacteroides_cellulosilyticus	All Ulcerative Colitis	0.552	2.21	0.803	1.
Bacteroides_eggerthii	All Crohn's Disease	-1.83	1.64	0.264	1.
Bacteroides_eggerthii	All Ulcerative Colitis	-0.0697	1.21	0.703	1.
Bacteroides_faecis	All Crohn's Disease	1.58	2.12	0.457	1.
Bacteroides_faecis	All Ulcerative Colitis	0.0394	2.29	0.986	1.
Bacteroides_finegoldii	All Crohn's Disease	1.09	2.17	0.614	1.
Bacteroides_finegoldii	All Ulcerative Colitis	0.333	2.27	0.883	1.
Bacteroides_fragilis	All Crohn's Disease	-0.562	1.21	0.872	1.
Bacteroides_fragilis	All Ulcerative Colitis	-1.45	1.19	0.269	1.
Bacteroides_intestinalis	All Crohn's Disease	0.993	2.17	0.647	1.
Bacteroides_intestinalis	All Ulcerative Colitis	1.4	2.11	0.506	1.
Bacteroides_nordii	All Crohn's Disease	-0.0828	2.37	0.972	1.
Bacteroides_nordii	All Ulcerative Colitis	0.887	2.16	0.681	1.
Bacteroides_ovatus	All Crohn's Disease	0.406	1.12	0.808	1.
Bacteroides_ovatus	All Ulcerative Colitis	0.61	1.1	0.403	1.
Bacteroides_salyersiae	All Crohn's Disease	1.61	2.12	0.448	1.
Bacteroides_salyersiae	All Ulcerative Colitis	-0.0481	2.31	0.983	1.

Bacteroides_stercoris	All Crohn's Disease	0.933	1.2	0.685	1.
Bacteroides_stercoris	All Ulcerative Colitis	0.28	1.19	0.931	1.
Bacteroides_thetaiotaomicron	All Crohn's Disease	0.363	1.12	0.634	1.
Bacteroides_thetaiotaomicron	All Ulcerative Colitis	-0.43	1.09	0.605	1.
Bacteroides_uniformis	All Crohn's Disease	-0.939	2.17	0.486	1.
Bacteroides_uniformis	All Ulcerative Colitis	-2.2	2.08	0.474	1.
Bacteroides_xylanisolvens	All Crohn's Disease	0.618	1.21	0.848	1.
Bacteroides_xylanisolvens	All Ulcerative Colitis	-1.08	1.35	0.666	1.
Barnesiella_intestinihominis	All Crohn's Disease	0.598	1.21	0.856	1.
Barnesiella_intestinihominis	All Ulcerative Colitis	0.218	1.2	0.979	1.
Bifidobacterium_adolescentis	All Crohn's Disease	2.43	2.09	0.244	1.
Bifidobacterium_adolescentis	All Ulcerative Colitis	2.32	2.07	0.263	1.
Bifidobacterium_animalis	All Crohn's Disease	-1.94	1.65	0.239	1.
Bifidobacterium_animalis	All Ulcerative Colitis	6.10×10^{-03}	1.23	0.959	1.
Bifidobacterium_bifidum	All Crohn's Disease	0.976	1.21	0.138	1.
Bifidobacterium_bifidum	All Ulcerative Colitis	0.735	1.18	0.662	1.
Bifidobacterium_breve	All Crohn's Disease	-0.781	1.34	0.561	1.
Bifidobacterium_breve	All Ulcerative Colitis	-1.12	1.36	0.414	1.
Bifidobacterium_catenulatum	All Crohn's Disease	0.985	2.17	0.65	1.
Bifidobacterium_catenulatum	All Ulcerative Colitis	0.897	2.15	0.676	1.
Bifidobacterium_dentium	All Crohn's Disease	-0.938	1.36	0.739	1.
Bifidobacterium_dentium	All Ulcerative Colitis	-2.80×10^{-03}	1.23	0.831	1.
Bifidobacterium_longum	All Crohn's Disease	-1.03	2.17	0.234	1.
Bifidobacterium_longum	All Ulcerative Colitis	-1.32	2.11	0.546	1.
Bifidobacterium_pseudocatenulatum	All Crohn's Disease	-0.879	1.21	0.309	1.
Bifidobacterium_pseudocatenulatum	All Ulcerative Colitis	-1.65	1.22	0.0678	1.
Bilophila_wadsworthia	All Crohn's Disease	-0.886	1.21	0.713	1.
Bilophila_wadsworthia	All Ulcerative Colitis	-1.93	1.24	0.222	1.
Bittarella_massiliensis	All Crohn's Disease	0.558	1.21	0.731	1.
Bittarella_massiliensis	All Ulcerative Colitis	-0.0153	1.21	0.969	1.
Blautia_argi	All Crohn's Disease	-1.9	1.65	0.249	1.
Blautia_argi	All Ulcerative Colitis	-0.4	1.25	0.749	1.
Blautia_caecimuris	All Crohn's Disease	-1.33	1.21	0.469	1.
Blautia_caecimuris	All Ulcerative Colitis	-1.01	1.18	0.628	1.
Blautia_faecis	All Crohn's Disease	-0.0583	2.37	0.508	1.
Blautia_faecis	All Ulcerative Colitis	-1.44	2.12	0.303	1.
Blautia_glucerasea	All Crohn's Disease	-0.208	1.26	0.869	1.
Blautia_glucerasea	All Ulcerative Colitis	-1.07	1.35	0.427	1.
Blautia_hansenii	All Crohn's Disease	0.832	2.18	0.703	1.
Blautia_hansenii	All Ulcerative Colitis	2.65	2.11	0.209	1.
Blautia_hominis	All Crohn's Disease	-0.0776	2.37	0.974	1.
Blautia_hominis	All Ulcerative Colitis	2.2	2.08	0.291	1.
Blautia_hydrogenotrophica	All Crohn's Disease	2.73	2.08	0.19	1.
Blautia_hydrogenotrophica	All Ulcerative Colitis	1.43	2.11	0.497	1.
Blautia_massiliensis	All Crohn's Disease	-1.02	2.17	0.768	1.
Blautia_massiliensis	All Ulcerative Colitis	-2.34	2.07	0.451	1.
Blautia_obeum	All Crohn's Disease	0.192	1.26	0.42	1.
Blautia_obeum	All Ulcerative Colitis	-0.689	1.19	0.768	1.
Blautia_producta	All Crohn's Disease	-0.143	1.14	0.959	1.
Blautia_producta	All Ulcerative Colitis	0.179	1.15	0.529	1.
Blautia_schinkii	All Crohn's Disease	-0.279	1.26	0.162	1.
Blautia_schinkii	All Ulcerative Colitis	-0.402	1.24	0.194	1.
Blautia_SGB4805	All Crohn's Disease	-0.268	1.23	0.811	1.
Blautia_SGB4805	All Ulcerative Colitis	-1.9	1.21	0.222	1.
Blautia_SGB4815	All Crohn's Disease	0.33	1.12	0.464	1.
Blautia_SGB4815	All Ulcerative Colitis	-0.642	1.1	0.564	1.
Blautia_SGB4831	All Crohn's Disease	0.113	1.24	0.947	1.
Blautia_SGB4831	All Ulcerative Colitis	-0.988	1.35	0.712	1.
Blautia_sp_AF19_10LB	All Crohn's Disease	2.49	2.09	0.234	1.
Blautia_sp_AF19_10LB	All Ulcerative Colitis	0.547	2.21	0.804	1.
Blautia_sp_MSK_20_85	All Crohn's Disease	-0.237	1.26	0.851	1.
Blautia_sp_MSK_20_85	All Ulcerative Colitis	-1.81	1.54	0.24	1.

Blautia_sp_MSK_21_1	All Crohn's Disease	-0.774	1.34	0.565	1.
Blautia_sp_MSK_21_1	All Ulcerative Colitis	-0.64	1.28	0.619	1.
Blautia_sp_OF03_15BH	All Crohn's Disease	-0.41	1.29	0.594	1.
Blautia_sp_OF03_15BH	All Ulcerative Colitis	-0.56	1.32	0.836	1.
Blautia_stercoris	All Crohn's Disease	2.01	2.1	0.337	1.
Blautia_stercoris	All Ulcerative Colitis	1.41	2.11	0.504	1.
Blautia_wexlerae	All Crohn's Disease	-0.984	2.17	0.319	1.
Blautia_wexlerae	All Ulcerative Colitis	-0.0502	2.28	0.57	1.
Butyricicoccus_SGB14990	All Crohn's Disease	-2.76	1.58	0.0795	1.
Butyricicoccus_SGB14990	All Ulcerative Colitis	-2.8	1.5	0.0613	1.
Butyricicoccus_sp_AM29_23AC	All Crohn's Disease	2.13	2.1	0.311	1.
Butyricicoccus_sp_AM29_23AC	All Ulcerative Colitis	-0.509	2.47	0.837	1.
Butyricimonas_SGB1783	All Crohn's Disease	1.1	2.17	0.614	1.
Butyricimonas_SGB1783	All Ulcerative Colitis	0.311	2.27	0.891	1.
Campylobacter_gracilis	All Crohn's Disease	-0.0218	2.36	0.993	1.
Campylobacter_gracilis	All Ulcerative Colitis	1.4	2.11	0.506	1.
Candidatus_Avimicrobium_caecorum	All Crohn's Disease	-0.282	1.26	0.969	1.
Candidatus_Avimicrobium_caecorum	All Ulcerative Colitis	3.50×10^{-04}	1.21	0.677	1.
Candidatus_Avimonas_narfia	All Crohn's Disease	-0.044	2.36	0.985	1.
Candidatus_Avimonas_narfia	All Ulcerative Colitis	0.904	2.15	0.674	1.
Candidatus_Borkfalkia_ceftriaxoniphila	All Crohn's Disease	0.995	2.17	0.646	1.
Candidatus_Borkfalkia_ceftriaxoniphila	All Ulcerative Colitis	0.885	2.15	0.68	1.
Candidatus_Cibiobacter_qucibialis	All Crohn's Disease	0.719	1.14	0.776	1.
Candidatus_Cibiobacter_qucibialis	All Ulcerative Colitis	-0.657	1.1	0.528	1.
Candidatus_Gastranaerophilales_bacterium	All Crohn's Disease	1.54	2.12	0.467	1.
Candidatus_Gastranaerophilales_bacterium	All Ulcerative Colitis	0.0448	2.29	0.984	1.
Candidatus_Parachristensenella_avicola	All Crohn's Disease	-0.0481	2.36	0.984	1.
Candidatus_Parachristensenella_avicola	All Ulcerative Colitis	0.905	2.15	0.673	1.
Candidatus_Paralachnospira_caecorum	All Crohn's Disease	0.955	2.17	0.66	1.
Candidatus_Paralachnospira_caecorum	All Ulcerative Colitis	0.902	2.15	0.675	1.
Candidatus_Pararuminococcus_gallinarum	All Crohn's Disease	2.	2.1	0.339	1.
Candidatus_Pararuminococcus_gallinarum	All Ulcerative Colitis	0.9	2.15	0.675	1.
Candidatus_Pseudoruminococcus_merdavium	All Crohn's Disease	0.905	2.17	0.677	1.
Candidatus_Pseudoruminococcus_merdavium	All Ulcerative Colitis	1.4	2.12	0.51	1.
Candidatus_Saccharibacteria_unclassified_SGB19850	All Crohn's Disease	-2.35	1.29	0.133	1.
Candidatus_Saccharibacteria_unclassified_SGB19850	All Ulcerative Colitis	-1.19	1.23	0.555	1.
Candidatus_Schneewindia_gallinarum	All Crohn's Disease	0.927	2.17	0.669	1.
Candidatus_Schneewindia_gallinarum	All Ulcerative Colitis	0.873	2.16	0.686	1.
Catabacter_hongkongensis	All Crohn's Disease	1.5	2.12	0.478	1.
Catabacter_hongkongensis	All Ulcerative Colitis	1.41	2.12	0.505	1.
Catenibacillus_scindens	All Crohn's Disease	-0.829	1.34	0.538	1.
Catenibacillus_scindens	All Ulcerative Colitis	-0.944	1.31	0.472	1.
Christensenella_massiliensis	All Crohn's Disease	0.0198	2.36	0.993	1.
Christensenella_massiliensis	All Ulcerative Colitis	-0.0766	2.33	0.974	1.
Christensenella_minuta	All Crohn's Disease	0.133	1.23	0.717	1.
Christensenella_minuta	All Ulcerative Colitis	-0.408	1.25	0.806	1.
Christensenellaceae_bacterium	All Crohn's Disease	-0.743	1.14	0.763	1.
Christensenellaceae_bacterium	All Ulcerative Colitis	-1.41	1.16	0.399	1.
Citrobacter_freundii	All Crohn's Disease	0.875	2.18	0.688	1.
Citrobacter_freundii	All Ulcerative Colitis	0.761	2.2	0.729	1.
Clostridia_bacterium	All Crohn's Disease	-0.988	2.17	0.218	1.
Clostridia_bacterium	All Ulcerative Colitis	-2.71	2.07	0.0462	1.
Clostridia_bacterium_UC5_1_1D1	All Crohn's Disease	-0.984	1.21	0.658	1.
Clostridia_bacterium_UC5_1_1D1	All Ulcerative Colitis	-1.58	1.19	0.336	1.
Clostridia_unclassified_SGB14844	All Crohn's Disease	1.48	2.12	0.486	1.
Clostridia_unclassified_SGB14844	All Ulcerative Colitis	1.37	2.13	0.52	1.
Clostridia_unclassified_SGB15402	All Crohn's Disease	0.929	2.17	0.669	1.
Clostridia_unclassified_SGB15402	All Ulcerative Colitis	0.877	2.16	0.685	1.
Clostridia_unclassified_SGB4121	All Crohn's Disease	-1.11	2.18	0.847	1.
Clostridia_unclassified_SGB4121	All Ulcerative Colitis	-1.96	2.11	0.58	1.
Clostridia_unclassified_SGB4367	All Crohn's Disease	-0.857	1.35	0.524	1.
Clostridia_unclassified_SGB4367	All Ulcerative Colitis	-0.403	1.24	0.746	1.

Clostridia_unclassified_SGB6276	All Crohn's Disease	1.53	2.12	0.472	1.
Clostridia_unclassified_SGB6276	All Ulcerative Colitis	0.0243	2.3	0.992	1.
Clostridiaceae_bacterium	All Crohn's Disease	-1.57	2.12	0.55	1.
Clostridiaceae_bacterium	All Ulcerative Colitis	-2.97	2.07	0.281	1.
Clostridiaceae_bacterium_DONG20_135	All Crohn's Disease	1.02	2.17	0.638	1.
Clostridiaceae_bacterium_DONG20_135	All Ulcerative Colitis	0.826	2.16	0.702	1.
Clostridiaceae_bacterium_NSJ_33	All Crohn's Disease	-0.14	2.37	0.953	1.
Clostridiaceae_bacterium_NSJ_33	All Ulcerative Colitis	1.37	2.13	0.52	1.
Clostridiaceae_bacterium_OM08_6BH	All Crohn's Disease	-1.63	1.23	0.338	1.
Clostridiaceae_bacterium_OM08_6BH	All Ulcerative Colitis	-3.38	1.5	0.0472	1.
Clostridiales_bacterium	All Crohn's Disease	0.59	1.21	0.192	1.
Clostridiales_bacterium	All Ulcerative Colitis	-1.82	1.54	0.238	1.
Clostridiales_bacterium_1_7_47FAA	All Crohn's Disease	2.01	2.1	0.337	1.
Clostridiales_bacterium_1_7_47FAA	All Ulcerative Colitis	0.892	2.15	0.678	1.
Clostridiales_bacterium_KLE1615	All Crohn's Disease	-0.627	1.21	0.801	1.
Clostridiales_bacterium_KLE1615	All Ulcerative Colitis	-0.989	1.18	0.643	1.
Clostridiales_bacterium_Marseille_P5551	All Crohn's Disease	0.926	2.17	0.67	1.
Clostridiales_bacterium_Marseille_P5551	All Ulcerative Colitis	0.872	2.16	0.687	1.
Clostridiales_bacterium_NSJ_40	All Crohn's Disease	-0.0828	2.37	0.972	1.
Clostridiales_bacterium_NSJ_40	All Ulcerative Colitis	0.887	2.16	0.681	1.
Clostridiales_bacterium_UBA1390	All Crohn's Disease	0.929	2.17	0.669	1.
Clostridiales_bacterium_UBA1390	All Ulcerative Colitis	0.877	2.16	0.685	1.
Clostridiales_Family_XIII_Incertae_Sedis_unclassified_SGB3978	All Crohn's Disease	-1.77	1.65	0.282	1.
Clostridiales_Family_XIII_Incertae_Sedis_unclassified_SGB3978	All Ulcerative Colitis	-1.54	1.54	0.316	1.
Clostridiales_unclassified_SGB15145	All Crohn's Disease	1.04	2.17	0.633	1.
Clostridiales_unclassified_SGB15145	All Ulcerative Colitis	0.765	2.17	0.724	1.
Clostridioides_difficile	All Crohn's Disease	0.988	2.17	0.649	1.
Clostridioides_difficile	All Ulcerative Colitis	0.894	2.15	0.677	1.
Clostridium_disporicum	All Crohn's Disease	-1.35	1.21	0.459	1.
Clostridium_disporicum	All Ulcerative Colitis	-2.3	1.24	0.124	1.
Clostridium_fessum	All Crohn's Disease	-2.76	2.08	0.337	1.
Clostridium_fessum	All Ulcerative Colitis	-3.53	2.08	0.172	1.
Clostridium_innocuum	All Crohn's Disease	-1.12	1.23	0.226	1.
Clostridium_innocuum	All Ulcerative Colitis	0.827	1.38	0.226	1.
Clostridium_leptum	All Crohn's Disease	-2.05	2.1	0.51	1.
Clostridium_leptum	All Ulcerative Colitis	-3.44	2.08	0.185	1.
Clostridium_methylpentosum	All Crohn's Disease	1.99	2.1	0.344	1.
Clostridium_methylpentosum	All Ulcerative Colitis	0.0453	2.29	0.984	1.
Clostridium_paraputrificum	All Crohn's Disease	-0.88	1.35	0.525	1.
Clostridium_paraputrificum	All Ulcerative Colitis	0.357	1.2	0.917	1.
Clostridium_perfringens	All Crohn's Disease	1.54	2.12	0.468	1.
Clostridium_perfringens	All Ulcerative Colitis	1.43	2.11	0.497	1.
Clostridium_phoceensis	All Crohn's Disease	-0.0834	1.12	0.716	1.
Clostridium_phoceensis	All Ulcerative Colitis	-0.95	1.13	0.643	1.
Clostridium_saccharogumia	All Crohn's Disease	0.236	1.23	0.537	1.
Clostridium_saccharogumia	All Ulcerative Colitis	-1.93	1.59	0.226	1.
Clostridium_scindens	All Crohn's Disease	-0.246	1.23	0.5	1.
Clostridium_scindens	All Ulcerative Colitis	-1.57	1.19	0.338	1.
Clostridium_SGB6179	All Crohn's Disease	-1.34	1.21	0.175	1.
Clostridium_SGB6179	All Ulcerative Colitis	-2.31	1.24	0.116	1.
Clostridium_sp_AF15_49	All Crohn's Disease	-1.8	1.64	0.272	1.
Clostridium_sp_AF15_49	All Ulcerative Colitis	-1.07	1.35	0.427	1.
Clostridium_sp_AF20_17LB	All Crohn's Disease	0.0357	1.11	0.864	1.
Clostridium_sp_AF20_17LB	All Ulcerative Colitis	-1.16	1.14	0.522	1.
Clostridium_sp_AF27_2AA	All Crohn's Disease	1.59	2.12	0.454	1.
Clostridium_sp_AF27_2AA	All Ulcerative Colitis	0.862	2.15	0.689	1.
Clostridium_sp_AF34_10BH	All Crohn's Disease	1.77	1.26	0.296	1.
Clostridium_sp_AF34_10BH	All Ulcerative Colitis	-0.735	1.1	0.402	1.
Clostridium_sp_AF36_4	All Crohn's Disease	-0.354	1.12	0.753	1.
Clostridium_sp_AF36_4	All Ulcerative Colitis	-1.43	1.16	0.389	1.
Clostridium_sp_AM22_11AC	All Crohn's Disease	-2.03	2.1	0.554	1.
Clostridium_sp_AM22_11AC	All Ulcerative Colitis	-3.2	2.07	0.23	1.

Clostridium_sp_AM33_3	All Crohn's Disease	-0.293	1.12	0.958	1.
Clostridium_sp_AM33_3	All Ulcerative Colitis	-0.975	1.14	0.179	1.
Clostridium_sp_AM49_4BH	All Crohn's Disease	2.37	2.09	0.256	1.
Clostridium_sp_AM49_4BH	All Ulcerative Colitis	0.0499	2.29	0.983	1.
Clostridium_sp_AT4	All Crohn's Disease	-0.312	1.27	0.676	1.
Clostridium_sp_AT4	All Ulcerative Colitis	-0.407	1.25	0.935	1.
Clostridium_sp_Marseille_P3244	All Crohn's Disease	1.54	2.12	0.466	1.
Clostridium_sp_Marseille_P3244	All Ulcerative Colitis	0.905	2.15	0.674	1.
Clostridium_sp_NSJ_42	All Crohn's Disease	0.898	1.21	0.706	1.
Clostridium_sp_NSJ_42	All Ulcerative Colitis	-0.927	1.32	0.456	1.
Clostridium_sp_SN20	All Crohn's Disease	-0.224	1.26	0.859	1.
Clostridium_sp_SN20	All Ulcerative Colitis	-1.01	1.33	0.449	1.
Clostridium_spiroforme	All Crohn's Disease	-0.443	1.13	0.758	1.
Clostridium_spiroforme	All Ulcerative Colitis	0.0623	1.1	0.708	1.
Clostridium_symbiosum	All Crohn's Disease	0.4	1.12	0.564	1.
Clostridium_symbiosum	All Ulcerative Colitis	-0.256	1.09	0.329	1.
Collinsella_aerofaciens	All Crohn's Disease	0.355	1.12	0.573	1.
Collinsella_aerofaciens	All Ulcerative Colitis	-1.03	1.12	0.589	1.
Collinsella_intestinalis	All Crohn's Disease	-0.104	2.37	0.965	1.
Collinsella_intestinalis	All Ulcerative Colitis	2.22	2.09	0.288	1.
Collinsella_tanakaei	All Crohn's Disease	-0.044	2.36	0.985	1.
Collinsella_tanakaei	All Ulcerative Colitis	0.904	2.15	0.674	1.
Coprobacillus_cateniformis	All Crohn's Disease	-0.347	1.28	0.786	1.
Coprobacillus_cateniformis	All Ulcerative Colitis	-1.01	1.36	0.458	1.
Coprobacter_fastidiosus	All Crohn's Disease	1.08	2.17	0.62	1.
Coprobacter_fastidiosus	All Ulcerative Colitis	0.51	2.22	0.819	1.
Coprobacter_secundus	All Crohn's Disease	0.943	2.17	0.664	1.
Coprobacter_secundus	All Ulcerative Colitis	0.893	2.15	0.678	1.
Coprococcus_catus	All Crohn's Disease	0.589	1.21	0.474	1.
Coprococcus_catus	All Ulcerative Colitis	-0.0804	1.21	0.997	1.
Coprococcus_comes	All Crohn's Disease	0.779	1.14	0.744	1.
Coprococcus_comes	All Ulcerative Colitis	-0.836	1.12	0.702	1.
Coprococcus_eutactus	All Crohn's Disease	2.35	2.09	0.261	1.
Coprococcus_eutactus	All Ulcerative Colitis	0.87	2.16	0.687	1.
Corynebacterium_argentoratense	All Crohn's Disease	0.986	2.17	0.649	1.
Corynebacterium_argentoratense	All Ulcerative Colitis	0.0486	2.29	0.983	1.
Corynebacterium_durum	All Crohn's Disease	-0.385	1.28	0.925	1.
Corynebacterium_durum	All Ulcerative Colitis	-0.0352	1.25	0.97	1.
Desulfovibrio_fairfieldensis	All Crohn's Disease	-0.0293	2.37	0.99	1.
Desulfovibrio_fairfieldensis	All Ulcerative Colitis	0.0487	2.29	0.983	1.
Desulfovibrio_piger	All Crohn's Disease	-0.898	1.36	0.508	1.
Desulfovibrio_piger	All Ulcerative Colitis	-1.82	1.56	0.243	1.
Dialister_invisus	All Crohn's Disease	-0.616	1.21	0.612	1.
Dialister_invisus	All Ulcerative Colitis	-0.741	1.18	0.388	1.
Dialister_pneumosintes	All Crohn's Disease	1.14	2.19	0.602	1.
Dialister_pneumosintes	All Ulcerative Colitis	-0.308	2.46	0.9	1.
Dielma_fastidiosa	All Crohn's Disease	-0.67	1.14	0.471	1.
Dielma_fastidiosa	All Ulcerative Colitis	-1.42	1.2	0.417	1.
Dorea_formicigenerans	All Crohn's Disease	1.2	1.17	0.522	1.
Dorea_formicigenerans	All Ulcerative Colitis	-0.459	1.09	0.889	1.
Dorea_longicatena	All Crohn's Disease	-1.6	2.12	0.698	1.
Dorea_longicatena	All Ulcerative Colitis	-2.63	2.07	0.366	1.
Dorea_phocaeensis	All Crohn's Disease	0.973	2.17	0.654	1.
Dorea_phocaeensis	All Ulcerative Colitis	0.0523	2.29	0.982	1.
Dorea_sp_AF24_7LB	All Crohn's Disease	-0.876	1.21	0.232	1.
Dorea_sp_AF24_7LB	All Ulcerative Colitis	-3.36	1.49	0.0471	1.
Dorea_sp_AF36_15AT	All Crohn's Disease	-1.16	1.17	0.542	1.
Dorea_sp_AF36_15AT	All Ulcerative Colitis	-1.49	1.18	0.309	1.
Dysosmobacter_sp_NSJ_60	All Crohn's Disease	-1.21	1.17	0.514	1.
Dysosmobacter_sp_NSJ_60	All Ulcerative Colitis	-0.971	1.12	0.621	1.
Dysosmobacter_welbionis	All Crohn's Disease	-0.185	1.23	0.275	1.
Dysosmobacter_welbionis	All Ulcerative Colitis	-1.73	1.2	0.276	1.

Eggerthella_lenta	All Crohn's Disease	-0.916	2.17	0.858	1.
Eggerthella_lenta	All Ulcerative Colitis	-2.56	2.08	0.39	1.
Eggerthella_timonensis	All Crohn's Disease	0.962	2.17	0.658	1.
Eggerthella_timonensis	All Ulcerative Colitis	0.0471	2.29	0.984	1.
Eggerthellaceae_unclassified_SGB14322	All Crohn's Disease	0.985	2.17	0.65	1.
Eggerthellaceae_unclassified_SGB14322	All Ulcerative Colitis	0.897	2.15	0.676	1.
Eggerthellaceae_unclassified_SGB14341	All Crohn's Disease	0.197	1.23	0.0257	1.
Eggerthellaceae_unclassified_SGB14341	All Ulcerative Colitis	-0.442	1.24	4.77×10^{-03}	1.
Eikenella_corrodens	All Crohn's Disease	1.08	2.17	0.619	1.
Eikenella_corrodens	All Ulcerative Colitis	0.492	2.23	0.825	1.
Eisenbergiella_massiliensis	All Crohn's Disease	-0.919	1.21	0.342	1.
Eisenbergiella_massiliensis	All Ulcerative Colitis	-1.45	1.19	0.26	1.
Eisenbergiella_sp_OF01_20	All Crohn's Disease	1.01	2.17	0.642	1.
Eisenbergiella_sp_OF01_20	All Ulcerative Colitis	0.0175	2.29	0.994	1.
Eisenbergiella_tayi	All Crohn's Disease	3.08	2.09	0.14	1.
Eisenbergiella_tayi	All Ulcerative Colitis	0.893	2.15	0.679	1.
Emergencia_timonensis	All Crohn's Disease	0.927	2.17	0.669	1.
Emergencia_timonensis	All Ulcerative Colitis	0.873	2.16	0.686	1.
Enterocloster_aldensis	All Crohn's Disease	0.0213	1.11	0.798	1.
Enterocloster_aldensis	All Ulcerative Colitis	-0.198	1.09	0.948	1.
Enterocloster_asparagiformis	All Crohn's Disease	0.196	1.23	0.984	1.
Enterocloster_asparagiformis	All Ulcerative Colitis	-0.951	1.31	0.719	1.
Enterocloster_bolteae	All Crohn's Disease	0.801	1.34	0.798	1.
Enterocloster_bolteae	All Ulcerative Colitis	-0.505	1.18	0.891	1.
Enterocloster_citroniae	All Crohn's Disease	3.16	2.09	0.129	1.
Enterocloster_citroniae	All Ulcerative Colitis	1.97	2.08	0.345	1.
Enterocloster_clostridioformis	All Crohn's Disease	0.878	1.21	0.521	1.
Enterocloster_clostridioformis	All Ulcerative Colitis	1.02	1.2	0.632	1.
Enterocloster_lavalensis	All Crohn's Disease	-0.768	1.14	0.361	1.
Enterocloster_lavalensis	All Ulcerative Colitis	-1.88	1.23	0.0976	1.
Enterococcus_avium	All Crohn's Disease	-1.14	1.4	0.415	1.
Enterococcus_avium	All Ulcerative Colitis	-0.844	1.44	0.559	1.
Enterococcus_faecalis	All Crohn's Disease	-0.354	1.27	0.718	1.
Enterococcus_faecalis	All Ulcerative Colitis	-6.46×10^{-03}	1.24	0.621	1.
Enterococcus_faecium	All Crohn's Disease	-0.0441	2.36	0.985	1.
Enterococcus_faecium	All Ulcerative Colitis	2.16	2.08	0.299	1.
Enterococcus_gallinarum	All Crohn's Disease	0.965	2.17	0.656	1.
Enterococcus_gallinarum	All Ulcerative Colitis	1.43	2.11	0.498	1.
Enteroscipio_rubneri	All Crohn's Disease	0.927	2.17	0.669	1.
Enteroscipio_rubneri	All Ulcerative Colitis	0.873	2.16	0.686	1.
Erysipelatoclostridium_ramosum	All Crohn's Disease	0.336	1.12	0.944	1.
Erysipelatoclostridium_ramosum	All Ulcerative Colitis	0.198	1.09	0.942	1.
Erysipelatoclostridium_sp_An15	All Crohn's Disease	1.02	2.17	0.637	1.
Erysipelatoclostridium_sp_An15	All Ulcerative Colitis	-0.0244	2.31	0.992	1.
Erysipelotrichaceae_bacterium	All Crohn's Disease	-0.0692	2.37	0.977	1.
Erysipelotrichaceae_bacterium	All Ulcerative Colitis	0.899	2.15	0.676	1.
Erysipelotrichaceae_bacterium_3_1_53	All Crohn's Disease	-0.219	1.26	0.136	1.
Erysipelotrichaceae_bacterium_3_1_53	All Ulcerative Colitis	-0.126	1.22	0.19	1.
Escherichia_coli	All Crohn's Disease	1.71	1.23	0.3	1.
Escherichia_coli	All Ulcerative Colitis	1.03	1.18	0.621	1.
Eubacteriaceae_bacterium	All Crohn's Disease	0.359	1.12	0.724	1.
Eubacteriaceae_bacterium	All Ulcerative Colitis	-0.712	1.1	0.648	1.
Eubacteriaceae_bacterium_CHKCI004	All Crohn's Disease	-0.822	1.35	0.541	1.
Eubacteriaceae_bacterium_CHKCI004	All Ulcerative Colitis	-1.8	1.54	0.242	1.
Eubacterium_brachy	All Crohn's Disease	-0.259	1.26	0.569	1.
Eubacterium_brachy	All Ulcerative Colitis	0.604	1.18	0.745	1.
Eubacterium_ramulus	All Crohn's Disease	0.733	1.13	0.426	1.
Eubacterium_ramulus	All Ulcerative Colitis	-0.4	1.09	0.868	1.
Eubacterium_rectale	All Crohn's Disease	-1.08	2.17	0.742	1.
Eubacterium_rectale	All Ulcerative Colitis	-1.43	2.12	0.75	1.
Eubacterium_siraeum	All Crohn's Disease	-0.238	1.26	0.839	1.
Eubacterium_siraeum	All Ulcerative Colitis	-0.0686	1.21	0.18	1.

Eubacterium_sp_AF15_50	All Crohn's Disease	0.203	1.23	0.072	1.
Eubacterium_sp_AF15_50	All Ulcerative Colitis	-0.965	1.32	0.713	1.
Eubacterium_sp_AF34_35BH	All Crohn's Disease	2.	2.1	0.341	1.
Eubacterium_sp_AF34_35BH	All Ulcerative Colitis	0.0524	2.29	0.982	1.
Eubacterium_sp_AM28_29	All Crohn's Disease	-0.873	1.35	0.518	1.
Eubacterium_sp_AM28_29	All Ulcerative Colitis	-1.79	1.54	0.245	1.
Eubacterium_sp_An11	All Crohn's Disease	1.04	2.17	0.633	1.
Eubacterium_sp_An11	All Ulcerative Colitis	-0.0772	2.33	0.974	1.
Eubacterium_sulci	All Crohn's Disease	-0.14	1.14	0.816	1.
Eubacterium_sulci	All Ulcerative Colitis	-0.208	1.14	0.763	1.
Eubacterium_ventriosum	All Crohn's Disease	0.563	1.21	0.872	1.
Eubacterium_ventriosum	All Ulcerative Colitis	0.89	1.18	0.699	1.
Evtepia_gabavorous	All Crohn's Disease	0.965	1.21	0.668	1.
Evtepia_gabavorous	All Ulcerative Colitis	-0.14	1.22	0.802	1.
Faecalibacillus_faecis	All Crohn's Disease	0.543	1.21	0.586	1.
Faecalibacillus_faecis	All Ulcerative Colitis	2.27×10^{-03}	1.21	0.156	1.
Faecalibacillus_intestinalis	All Crohn's Disease	1.17	1.17	0.535	1.
Faecalibacillus_intestinalis	All Ulcerative Colitis	-0.114	1.08	0.945	1.
Faecalibacterium_prausnitzii	All Crohn's Disease	-1.02	2.17	0.868	1.
Faecalibacterium_prausnitzii	All Ulcerative Colitis	-1.33	2.11	0.186	1.
Faecalibacterium_SGB15346	All Crohn's Disease	1.25	1.21	0.516	1.
Faecalibacterium_SGB15346	All Ulcerative Colitis	0.368	1.2	0.376	1.
Faecalicatena_contorta	All Crohn's Disease	-2.08	2.1	0.538	1.
Faecalicatena_contorta	All Ulcerative Colitis	-3.08	2.07	0.254	1.
Faecalicatena_fissicatena	All Crohn's Disease	-0.0322	1.11	0.607	1.
Faecalicatena_fissicatena	All Ulcerative Colitis	-0.332	1.09	0.943	1.
Faecalimonas_umbilicata	All Crohn's Disease	-0.408	1.12	0.821	1.
Faecalimonas_umbilicata	All Ulcerative Colitis	-7.82×10^{-03}	1.09	0.781	1.
Finegoldia_magna	All Crohn's Disease	-0.0734	2.37	0.975	1.
Finegoldia_magna	All Ulcerative Colitis	1.84	2.09	0.378	1.
Firmicutes_bacterium	All Crohn's Disease	1.52	2.12	0.474	1.
Firmicutes_bacterium	All Ulcerative Colitis	0.882	2.16	0.683	1.
Firmicutes_bacterium_AF16_15	All Crohn's Disease	0.819	1.34	0.431	1.
Firmicutes_bacterium_AF16_15	All Ulcerative Colitis	-1.68	1.2	0.0587	1.
Flavonifractor_plautii	All Crohn's Disease	-2.01	2.1	0.52	1.
Flavonifractor_plautii	All Ulcerative Colitis	-1.79	2.09	0.628	1.
Frisingicoccus_caecimuris	All Crohn's Disease	-0.891	1.35	0.509	1.
Frisingicoccus_caecimuris	All Ulcerative Colitis	-0.404	1.25	0.747	1.
Frisingicoccus_SGB4674	All Crohn's Disease	-0.78	1.14	0.291	1.
Frisingicoccus_SGB4674	All Ulcerative Colitis	-1.87	1.23	7.54×10^{-03}	1.
Fusicatenibacter_saccharivorans	All Crohn's Disease	-1.07	2.17	0.858	1.
Fusicatenibacter_saccharivorans	All Ulcerative Colitis	-2.19	2.08	0.0811	1.
Fusobacterium_nucleatum	All Crohn's Disease	1.04	2.17	0.63	1.
Fusobacterium_nucleatum	All Ulcerative Colitis	2.27	2.07	0.274	1.
Gemella_haemolysans	All Crohn's Disease	-0.271	1.26	0.121	1.
Gemella_haemolysans	All Ulcerative Colitis	-9.94×10^{-03}	1.21	0.835	1.
Gemella_morbillorum	All Crohn's Disease	-1.24	1.18	0.501	1.
Gemella_morbillorum	All Ulcerative Colitis	-5.63×10^{-03}	1.09	0.689	1.
Gemella_sanguinis	All Crohn's Disease	-1.19	1.25	0.565	1.
Gemella_sanguinis	All Ulcerative Colitis	-0.766	1.26	0.543	1.
Gemmiger_formicilis	All Crohn's Disease	-1.64	2.12	0.686	1.
Gemmiger_SGB15299	All Crohn's Disease	-0.873	1.35	0.518	1.
Gemmiger_SGB15299	All Ulcerative Colitis	-1.79	1.54	0.245	1.
GGB12785_SGB19823	All Crohn's Disease	-1.88	1.64	0.254	1.
GGB12785_SGB19823	All Ulcerative Colitis	-0.402	1.24	0.746	1.
GGB2980_SGB3962	All Crohn's Disease	-1.27	1.18	0.488	1.
GGB2980_SGB3962	All Ulcerative Colitis	-1.36	1.17	0.427	1.
GGB2982_SGB3964	All Crohn's Disease	0.288	1.24	0.602	1.
GGB2982_SGB3964	All Ulcerative Colitis	-2.32	1.77	0.191	1.
GGB3005_SGB3996	All Crohn's Disease	1.56	2.12	0.461	1.
GGB3005_SGB3996	All Ulcerative Colitis	0.9	2.15	0.675	1.
GGB3034_SGB4030	All Crohn's Disease	0.952	2.17	0.661	1.

GGB3034_SGB4030	All Ulcerative Colitis	0.9	2.15	0.676	1.
GGB32463_SGB47515	All Crohn's Disease	-0.785	1.34	0.559	1.
GGB32463_SGB47515	All Ulcerative Colitis	-1.94	1.6	0.227	1.
GGB3256_SGB4303	All Crohn's Disease	-0.923	1.36	0.496	1.
GGB3256_SGB4303	All Ulcerative Colitis	-0.986	1.35	0.465	1.
GGB3293_SGB4348	All Crohn's Disease	0.971	2.17	0.655	1.
GGB3293_SGB4348	All Ulcerative Colitis	0.0518	2.29	0.982	1.
GGB3433_SGB4573	All Crohn's Disease	0.913	2.17	0.674	1.
GGB3433_SGB4573	All Ulcerative Colitis	0.848	2.17	0.696	1.
GGB3463_SGB4621	All Crohn's Disease	1.62	2.12	0.445	1.
GGB3463_SGB4621	All Ulcerative Colitis	1.28	2.12	0.546	1.
GGB3478_SGB4643	All Crohn's Disease	1.63	2.12	0.442	1.
GGB3478_SGB4643	All Ulcerative Colitis	0.715	2.18	0.743	1.
GGB35068_SGB47850	All Crohn's Disease	-1.85	1.64	0.261	1.
GGB35068_SGB47850	All Ulcerative Colitis	-0.939	1.31	0.475	1.
GGB3523_SGB4703	All Crohn's Disease	1.55	2.12	0.466	1.
GGB3523_SGB4703	All Ulcerative Colitis	0.0486	2.29	0.983	1.
GGB3537_SGB4727	All Crohn's Disease	0.951	2.17	0.661	1.
GGB3537_SGB4727	All Ulcerative Colitis	0.9	2.15	0.676	1.
GGB3570_SGB4777	All Crohn's Disease	2.02	2.1	0.336	1.
GGB3570_SGB4777	All Ulcerative Colitis	0.0356	2.29	0.988	1.
GGB3571_SGB4778	All Crohn's Disease	0.584	1.21	0.863	1.
GGB3571_SGB4778	All Ulcerative Colitis	-0.456	1.25	0.162	1.
GGB3583_SGB4799	All Crohn's Disease	1.58	2.12	0.457	1.
GGB3583_SGB4799	All Ulcerative Colitis	0.0384	2.29	0.987	1.
GGB3606_SGB4870	All Crohn's Disease	0.913	2.17	0.674	1.
GGB3606_SGB4870	All Ulcerative Colitis	0.848	2.17	0.696	1.
GGB3614_SGB4886	All Crohn's Disease	0.943	2.17	0.664	1.
GGB3614_SGB4886	All Ulcerative Colitis	0.893	2.15	0.678	1.
GGB36472_SGB47660	All Crohn's Disease	0.926	2.17	0.67	1.
GGB36472_SGB47660	All Ulcerative Colitis	0.872	2.16	0.687	1.
GGB3677_SGB4990	All Crohn's Disease	0.929	2.17	0.669	1.
GGB3677_SGB4990	All Ulcerative Colitis	0.877	2.16	0.685	1.
GGB3746_SGB5089	All Crohn's Disease	3.11	2.09	0.136	1.
GGB3746_SGB5089	All Ulcerative Colitis	2.12	2.08	0.307	1.
GGB42689_SGB59892	All Crohn's Disease	-0.0213	2.36	0.993	1.
GGB42689_SGB59892	All Ulcerative Colitis	1.4	2.11	0.507	1.
GGB4456_SGB6141	All Crohn's Disease	-0.337	1.12	0.878	1.
GGB4456_SGB6141	All Ulcerative Colitis	-0.77	1.11	0.737	1.
GGB4482_SGB6176	All Crohn's Disease	1.53	2.12	0.472	1.
GGB4482_SGB6176	All Ulcerative Colitis	0.0243	2.3	0.992	1.
GGB4491_SGB6188	All Crohn's Disease	1.57	2.12	0.457	1.
GGB4491_SGB6188	All Ulcerative Colitis	0.0408	2.29	0.986	1.
GGB45432_SGB63101	All Crohn's Disease	-0.416	1.12	0.0381	1.
GGB45432_SGB63101	All Ulcerative Colitis	-1.35	1.16	0.097	1.
GGB45491_SGB63163	All Crohn's Disease	0.956	2.17	0.659	1.
GGB45491_SGB63163	All Ulcerative Colitis	0.903	2.15	0.675	1.
GGB51441_SGB71759	All Crohn's Disease	0.263	1.23	0.0656	1.
GGB51441_SGB71759	All Ulcerative Colitis	-0.709	1.3	0.261	1.
GGB51510_SGB71883	All Crohn's Disease	1.03	2.17	0.633	1.
GGB51510_SGB71883	All Ulcerative Colitis	-0.07	2.32	0.976	1.
GGB51959_SGB72479	All Crohn's Disease	-0.84	1.35	0.533	1.
GGB51959_SGB72479	All Ulcerative Colitis	-1.78	1.53	0.244	1.
GGB58158_SGB79798	All Crohn's Disease	0.222	1.23	0.866	1.
GGB58158_SGB79798	All Ulcerative Colitis	-1.87	1.56	0.232	1.
GGB58233_SGB79883	All Crohn's Disease	1.6	2.12	0.45	1.
GGB58233_SGB79883	All Ulcerative Colitis	0.83	2.16	0.7	1.
GGB6561_SGB9269	All Crohn's Disease	1.04	2.17	0.632	1.
GGB6561_SGB9269	All Ulcerative Colitis	1.27	2.12	0.548	1.
GGB9176_SGB14114	All Crohn's Disease	0.943	2.17	0.664	1.
GGB9176_SGB14114	All Ulcerative Colitis	0.893	2.15	0.678	1.
GGB9186_SGB14125	All Crohn's Disease	0.974	2.17	0.653	1.

GGB9186_SGB14125	All Ulcerative Colitis	1.42	2.11	0.499	1.
GGB9480_SGB14875	All Crohn's Disease	1.03	2.17	0.636	1.
GGB9480_SGB14875	All Ulcerative Colitis	1.32	2.11	0.532	1.
GGB9494_SGB14891	All Crohn's Disease	0.971	2.17	0.654	1.
GGB9494_SGB14891	All Ulcerative Colitis	0.905	2.15	0.674	1.
GGB9522_SGB14921	All Crohn's Disease	1.49	2.12	0.482	1.
GGB9522_SGB14921	All Ulcerative Colitis	0.833	2.17	0.701	1.
GGB9530_SGB14930	All Crohn's Disease	1.56	2.12	0.462	1.
GGB9530_SGB14930	All Ulcerative Colitis	0.0522	2.28	0.982	1.
GGB9557_SGB14966	All Crohn's Disease	-0.774	1.34	0.565	1.
GGB9557_SGB14966	All Ulcerative Colitis	-1.16	1.38	0.4	1.
GGB9574_SGB14987	All Crohn's Disease	-0.0738	2.37	0.975	1.
GGB9574_SGB14987	All Ulcerative Colitis	0.895	2.15	0.678	1.
GGB9581_SGB14999	All Crohn's Disease	0.672	1.22	0.824	1.
GGB9581_SGB14999	All Ulcerative Colitis	-0.851	1.34	0.773	1.
GGB9581_SGB79823	All Crohn's Disease	1.02	2.17	0.639	1.
GGB9581_SGB79823	All Ulcerative Colitis	0.835	2.16	0.698	1.
GGB9602_SGB15031	All Crohn's Disease	-1.93	1.65	0.242	1.
GGB9602_SGB15031	All Ulcerative Colitis	-0.967	1.34	0.471	1.
GGB9608_SGB15041	All Crohn's Disease	-0.851	1.35	0.527	1.
GGB9608_SGB15041	All Ulcerative Colitis	-0.925	1.31	0.481	1.
GGB9614_SGB15049	All Crohn's Disease	-1.73	1.26	0.314	1.
GGB9614_SGB15049	All Ulcerative Colitis	-1.19	1.15	0.512	1.
GGB9615_SGB15052	All Crohn's Disease	-0.807	1.34	0.549	1.
GGB9615_SGB15052	All Ulcerative Colitis	-1.84	1.55	0.237	1.
GGB9615_SGB15053	All Crohn's Disease	0.425	1.12	0.0418	1.
GGB9615_SGB15053	All Ulcerative Colitis	-1.71	1.23	0.0593	1.
GGB9620_SGB15068	All Crohn's Disease	0.996	2.17	0.646	1.
GGB9620_SGB15068	All Ulcerative Colitis	0.883	2.15	0.681	1.
GGB9623_SGB15076	All Crohn's Disease	0.944	2.17	0.663	1.
GGB9623_SGB15076	All Ulcerative Colitis	0.894	2.15	0.678	1.
GGB9627_SGB15081	All Crohn's Disease	-0.923	1.36	0.496	1.
GGB9627_SGB15081	All Ulcerative Colitis	-0.986	1.35	0.465	1.
GGB9631_SGB15087	All Crohn's Disease	-0.873	1.35	0.518	1.
GGB9631_SGB15087	All Ulcerative Colitis	-1.79	1.54	0.245	1.
GGB9632_SGB15089	All Crohn's Disease	0.746	1.14	0.761	1.
GGB9632_SGB15089	All Ulcerative Colitis	-1.06	1.13	0.573	1.
GGB9633_SGB15090	All Crohn's Disease	-0.82	1.35	0.542	1.
GGB9633_SGB15090	All Ulcerative Colitis	-1.8	1.54	0.241	1.
GGB9633_SGB15091	All Crohn's Disease	1.49	2.12	0.483	1.
GGB9633_SGB15091	All Ulcerative Colitis	0.826	2.17	0.704	1.
GGB9635_SGB15106	All Crohn's Disease	-0.923	1.36	0.496	1.
GGB9635_SGB15106	All Ulcerative Colitis	-0.986	1.35	0.465	1.
GGB9640_SGB15115	All Crohn's Disease	-0.873	1.35	0.518	1.
GGB9640_SGB15115	All Ulcerative Colitis	-1.79	1.54	0.245	1.
GGB9642_SGB15119	All Crohn's Disease	-0.873	1.35	0.518	1.
GGB9642_SGB15119	All Ulcerative Colitis	-1.79	1.54	0.245	1.
GGB9646_SGB15123	All Crohn's Disease	1.65	2.12	0.438	1.
GGB9646_SGB15123	All Ulcerative Colitis	-0.249	2.38	0.917	1.
GGB9667_SGB15164	All Crohn's Disease	-0.191	1.26	0.709	1.
GGB9667_SGB15164	All Ulcerative Colitis	-2.03	1.64	0.216	1.
GGB9699_SGB15216	All Crohn's Disease	-0.0423	1.12	0.534	1.
GGB9699_SGB15216	All Ulcerative Colitis	-2.73	1.46	0.0614	1.
GGB9705_SGB15225	All Crohn's Disease	-0.328	1.27	0.797	1.
GGB9705_SGB15225	All Ulcerative Colitis	-1.85	1.58	0.24	1.
GGB9707_SGB15229	All Crohn's Disease	-0.796	1.14	0.736	1.
GGB9707_SGB15229	All Ulcerative Colitis	-2.74	1.46	0.0617	1.
GGB9712_SGB15244	All Crohn's Disease	-0.923	1.36	0.496	1.
GGB9712_SGB15244	All Ulcerative Colitis	-0.986	1.35	0.465	1.
GGB9730_SGB15291	All Crohn's Disease	-0.699	1.14	0.788	1.
GGB9730_SGB15291	All Ulcerative Colitis	-1.2	1.15	0.506	1.
GGB9758_SGB15368	All Crohn's Disease	-0.898	1.36	0.508	1.

GGB9758_SGB15368	All Ulcerative Colitis	-1.82	1.56	0.243	1.
Gordonibacter_pamelaeae	All Crohn's Disease	-1.29	1.21	0.491	1.
Gordonibacter_pamelaeae	All Ulcerative Colitis	-2.42	1.26	0.106	1.
Gordonibacter_urolithinfaciens	All Crohn's Disease	1.98	2.1	0.346	1.
Gordonibacter_urolithinfaciens	All Ulcerative Colitis	0.899	2.15	0.676	1.
Granulicatella_adiacens	All Crohn's Disease	0.961	2.17	0.657	1.
Granulicatella_adiacens	All Ulcerative Colitis	2.18	2.08	0.295	1.
Granulicatella_elegans	All Crohn's Disease	-0.179	2.38	0.94	1.
Granulicatella_elegans	All Ulcerative Colitis	1.31	2.15	0.543	1.
Haemophilus_parainfluenzae	All Crohn's Disease	-0.373	1.12	0.762	1.
Haemophilus_parainfluenzae	All Ulcerative Colitis	0.183	1.09	0.499	1.
Holdemania_filiformis	All Crohn's Disease	9.65×10^{-03}	1.11	0.507	1.
Holdemania_filiformis	All Ulcerative Colitis	-1.45	1.17	0.226	1.
Holdemania_massiliensis	All Crohn's Disease	-0.254	1.26	0.841	1.
Holdemania_massiliensis	All Ulcerative Colitis	-1.78	1.53	0.243	1.
Holdemania_sp_Marseille_P2844	All Crohn's Disease	0.921	2.17	0.671	1.
Holdemania_sp_Marseille_P2844	All Ulcerative Colitis	1.85	2.1	0.379	1.
Hungatella_hathewayi	All Crohn's Disease	1.19	1.17	0.524	1.
Hungatella_hathewayi	All Ulcerative Colitis	-0.167	1.09	0.938	1.
Hydrogeniiclostidium_mannosilyticum	All Crohn's Disease	0.536	1.22	0.381	1.
Hydrogeniiclostidium_mannosilyticum	All Ulcerative Colitis	-1.79	1.54	0.244	1.
Intestinibacter_bartlettii	All Crohn's Disease	-2.29	2.13	0.0413	1.
Intestinibacter_bartlettii	All Ulcerative Colitis	-0.654	2.25	0.726	1.
Intestinibacter_SGB6139	All Crohn's Disease	2.36	2.09	0.26	1.
Intestinibacter_SGB6139	All Ulcerative Colitis	0.887	2.16	0.681	1.
Intestinimonas_butyriciproducens	All Crohn's Disease	1.59	2.12	0.452	1.
Intestinimonas_butyriciproducens	All Ulcerative Colitis	2.05×10^{-03}	2.3	0.999	1.
Intestinimonas_gabonensis	All Crohn's Disease	-2.76	1.58	0.0795	1.
Intestinimonas_gabonensis	All Ulcerative Colitis	-2.8	1.5	0.0613	1.
Intestinimonas_massiliensis	All Crohn's Disease	1.6	2.12	0.45	1.
Intestinimonas_massiliensis	All Ulcerative Colitis	0.828	2.16	0.701	1.
Isoptericola_variabilis	All Crohn's Disease	0.173	1.27	0.853	1.
Isoptericola_variabilis	All Ulcerative Colitis	7.40×10^{-03}	1.23	0.955	1.
Klebsiella_pneumoniae	All Crohn's Disease	0.0515	2.37	0.983	1.
Klebsiella_pneumoniae	All Ulcerative Colitis	0.547	2.22	0.805	1.
Kytococcus_sedentarius	All Crohn's Disease	-0.833	1.34	0.535	1.
Kytococcus_sedentarius	All Ulcerative Colitis	-0.426	1.24	0.732	1.
Lachnoclostridium_edouardi	All Crohn's Disease	2.1	2.1	0.317	1.
Lachnoclostridium_edouardi	All Ulcerative Colitis	-0.335	2.41	0.889	1.
Lachnoclostridium_phocaeense	All Crohn's Disease	0.957	2.17	0.659	1.
Lachnoclostridium_phocaeense	All Ulcerative Colitis	1.43	2.11	0.497	1.
Lachnoclostridium_sp_An118	All Crohn's Disease	0.956	2.17	0.659	1.
Lachnoclostridium_sp_An118	All Ulcerative Colitis	1.84	2.09	0.379	1.
Lachnoclostridium_sp_An138	All Crohn's Disease	-0.824	1.34	0.54	1.
Lachnoclostridium_sp_An138	All Ulcerative Colitis	-0.953	1.32	0.469	1.
Lachnospira_eligens	All Crohn's Disease	0.582	1.21	0.783	1.
Lachnospira_eligens	All Ulcerative Colitis	-0.0621	1.21	0.539	1.
Lachnospira_pectinoschiza	All Crohn's Disease	-0.0825	2.37	0.972	1.
Lachnospira_pectinoschiza	All Ulcerative Colitis	2.53	2.08	0.224	1.
Lachnospira_SGB5077	All Crohn's Disease	2.	2.1	0.34	1.
Lachnospira_SGB5077	All Ulcerative Colitis	0.902	2.15	0.674	1.
Lachnospira_sp_NSJ_43	All Crohn's Disease	-1.19	1.17	0.526	1.
Lachnospira_sp_NSJ_43	All Ulcerative Colitis	-1.4	1.16	0.401	1.
Lachnospiraceae_bacterium	All Crohn's Disease	-1.66	2.12	0.559	1.
Lachnospiraceae_bacterium	All Ulcerative Colitis	-0.494	2.22	0.905	1.
Lachnospiraceae_bacterium_NSJ_29	All Crohn's Disease	-0.999	1.21	0.552	1.
Lachnospiraceae_bacterium_NSJ_29	All Ulcerative Colitis	-2.3	1.24	0.124	1.
Lachnospiraceae_bacterium_OF09_6	All Crohn's Disease	-0.87	1.35	0.519	1.
Lachnospiraceae_bacterium_OF09_6	All Ulcerative Colitis	-1.78	1.54	0.245	1.
Lachnospiraceae_bacterium_OM04_12BH	All Crohn's Disease	-0.793	1.34	0.555	1.
Lachnospiraceae_bacterium_OM04_12BH	All Ulcerative Colitis	-1.89	1.58	0.231	1.
Lachnospiraceae_bacterium_WCA3_601_WT_6H	All Crohn's Disease	-0.0489	1.12	0.772	1.

Lachnospiraceae_bacterium_WCA3_601_WT_6H	All Ulcerative Colitis	-1.88	1.23	0.239	1.
Lachnospiraceae_unclassified_SGB4882	All Crohn's Disease	0.196	1.23	0.905	1.
Lachnospiraceae_unclassified_SGB4882	All Ulcerative Colitis	-0.952	1.32	0.718	1.
Lachnospiraceae_unclassified_SGB66069	All Crohn's Disease	-0.0226	2.36	0.992	1.
Lachnospiraceae_unclassified_SGB66069	All Ulcerative Colitis	0.888	2.15	0.679	1.
Lacrimispora_amygdalina	All Crohn's Disease	-0.329	1.12	0.946	1.
Lacrimispora_amygdalina	All Ulcerative Colitis	-0.801	1.11	0.42	1.
Lacrimispora_celerecrescens	All Crohn's Disease	0.314	1.12	0.843	1.
Lacrimispora_celerecrescens	All Ulcerative Colitis	0.867	1.12	0.3	1.
Lacrimispora_saccharolytica	All Crohn's Disease	1.59	2.12	0.453	1.
Lacrimispora_saccharolytica	All Ulcerative Colitis	0.011	2.3	0.996	1.
Lacticaseibacillus_paracasei	All Crohn's Disease	-0.277	1.26	0.183	1.
Lacticaseibacillus_paracasei	All Ulcerative Colitis	0.639	1.19	0.247	1.
Lacticaseibacillus_rhamnosus	All Crohn's Disease	1.58	2.12	0.456	1.
Lacticaseibacillus_rhamnosus	All Ulcerative Colitis	2.11	2.08	0.311	1.
Lactobacillus_acidophilus	All Crohn's Disease	-0.0762	2.37	0.974	1.
Lactobacillus_acidophilus	All Ulcerative Colitis	1.43	2.11	0.498	1.
Lactobacillus_gasseri	All Crohn's Disease	1.01	2.17	0.639	1.
Lactobacillus_gasseri	All Ulcerative Colitis	1.74	2.09	0.405	1.
Lactococcus_lactis	All Crohn's Disease	0.312	1.12	0.72	1.
Lactococcus_lactis	All Ulcerative Colitis	0.568	1.11	0.846	1.
Lactococcus_piscium	All Crohn's Disease	-0.146	2.37	0.951	1.
Lactococcus_piscium	All Ulcerative Colitis	1.36	2.13	0.523	1.
Lactonifactor_sp_BIOML_A6	All Crohn's Disease	-0.822	1.34	0.541	1.
Lactonifactor_sp_BIOML_A6	All Ulcerative Colitis	-0.955	1.32	0.468	1.
Lancefieldella_parvula	All Crohn's Disease	-0.834	1.15	0.715	1.
Lancefieldella_parvula	All Ulcerative Colitis	-0.584	1.11	0.648	1.
Lancefieldella_rimae	All Crohn's Disease	-5.40×10^{-03}	2.36	0.998	1.
Lancefieldella_rimae	All Ulcerative Colitis	1.37	2.11	0.517	1.
Lawsonibacter_sp_NSJ_51	All Crohn's Disease	-0.044	2.36	0.985	1.
Lawsonibacter_sp_NSJ_51	All Ulcerative Colitis	0.904	2.15	0.674	1.
Lawsonibacter_sp_NSJ_52	All Crohn's Disease	1.63	2.12	0.442	1.
Lawsonibacter_sp_NSJ_52	All Ulcerative Colitis	-0.143	2.34	0.951	1.
Leuconostoc_mesenteroides	All Crohn's Disease	-0.14	2.37	0.953	1.
Leuconostoc_mesenteroides	All Ulcerative Colitis	1.37	2.13	0.52	1.
Limosilactobacillus_fermentum	All Crohn's Disease	2.01	2.1	0.339	1.
Limosilactobacillus_fermentum	All Ulcerative Colitis	0.0503	2.28	0.982	1.
Longibaculum_muris	All Crohn's Disease	-0.797	1.14	0.734	1.
Longibaculum_muris	All Ulcerative Colitis	-0.947	1.12	0.0469	1.
Longicatena_caecimuris	All Crohn's Disease	0.697	1.14	0.395	1.
Longicatena_caecimuris	All Ulcerative Colitis	-0.613	1.1	0.822	1.
Marvinbryantia_SGB4691	All Crohn's Disease	-0.0738	2.37	0.975	1.
Marvinbryantia_SGB4691	All Ulcerative Colitis	0.895	2.15	0.678	1.
Massilicoli_timonensis	All Crohn's Disease	0.992	2.17	0.647	1.
Massilicoli_timonensis	All Ulcerative Colitis	0.0435	2.29	0.985	1.
Massilimaliae_massiliensis	All Crohn's Disease	0.545	1.21	0.324	1.
Massilimaliae_massiliensis	All Ulcerative Colitis	-0.924	1.31	0.732	1.
Massilimaliae_timonensis	All Crohn's Disease	1.01	2.17	0.64	1.
Massilimaliae_timonensis	All Ulcerative Colitis	0.846	2.15	0.695	1.
Massilimicrobiota_sp_An134	All Crohn's Disease	0.964	2.17	0.657	1.
Massilimicrobiota_sp_An134	All Ulcerative Colitis	0.905	2.15	0.674	1.
Massilimicrobiota_timonensis	All Crohn's Disease	-0.28	1.26	0.969	1.
Massilimicrobiota_timonensis	All Ulcerative Colitis	-8.44×10^{-04}	1.21	0.867	1.
Massilioclostridium_coli	All Crohn's Disease	-1.86	1.64	0.257	1.
Massilioclostridium_coli	All Ulcerative Colitis	-0.41	1.24	0.741	1.
Massilistercora_timonensis	All Crohn's Disease	0.955	2.17	0.66	1.
Massilistercora_timonensis	All Ulcerative Colitis	0.902	2.15	0.675	1.
Mediterraneibacter_butyricigenes	All Crohn's Disease	-0.337	1.12	0.169	1.
Mediterraneibacter_butyricigenes	All Ulcerative Colitis	-1.49	1.17	0.367	1.
Mediterraneibacter_glycyrrhizinilyticus	All Crohn's Disease	-0.729	1.14	0.364	1.
Mediterraneibacter_glycyrrhizinilyticus	All Ulcerative Colitis	-1.06	1.13	0.572	1.
Mediterraneibacter_massiliensis	All Crohn's Disease	0.985	2.17	0.65	1.

Mediterraneibacter_massiliensis	All Ulcerative Colitis	0.897	2.15	0.676	1.
Mediterraneibacter_sp_gm002	All Crohn's Disease	-0.923	1.36	0.496	1.
Mediterraneibacter_sp_gm002	All Ulcerative Colitis	-0.986	1.35	0.465	1.
Megasphaera_micronuciformis	All Crohn's Disease	-0.139	2.37	0.953	1.
Megasphaera_micronuciformis	All Ulcerative Colitis	1.37	2.13	0.52	1.
Merdimonas_faecis	All Crohn's Disease	-0.813	1.34	0.777	1.
Merdimonas_faecis	All Ulcerative Colitis	-0.0785	1.21	0.874	1.
Mogibacterium_diversum	All Crohn's Disease	-2.51	2.09	0.407	1.
Mogibacterium_diversum	All Ulcerative Colitis	-3.05	2.08	0.265	1.
Monoglobus_pectinilyticus	All Crohn's Disease	-0.809	1.14	0.728	1.
Monoglobus_pectinilyticus	All Ulcerative Colitis	-1.36	1.16	0.427	1.
Morganella_morganii	All Crohn's Disease	-1.04	1.38	0.453	1.
Morganella_morganii	All Ulcerative Colitis	-1.25	1.47	0.397	1.
Murimonas_intestini	All Crohn's Disease	1.59	2.12	0.451	1.
Murimonas_intestini	All Ulcerative Colitis	-1.99×10^{-03}	2.3	0.999	1.
Negativibacillus_massiliensis	All Crohn's Disease	2.34	2.09	0.264	1.
Negativibacillus_massiliensis	All Ulcerative Colitis	-0.0612	2.33	0.979	1.
Neglecta_timonensis	All Crohn's Disease	0.963	2.17	0.657	1.
Neglecta_timonensis	All Ulcerative Colitis	0.905	2.15	0.674	1.
Neobittarella_massiliensis	All Crohn's Disease	-4.	2.12	0.114	1.
Neobittarella_massiliensis	All Ulcerative Colitis	-4.13	2.11	0.0968	1.
Odoribacter_splanchnicus	All Crohn's Disease	0.594	1.21	0.151	1.
Odoribacter_splanchnicus	All Ulcerative Colitis	-0.0964	1.21	0.477	1.
Oscillibacter_sp_ER4	All Crohn's Disease	-0.741	1.14	0.764	1.
Oscillibacter_sp_ER4	All Ulcerative Colitis	-2.76	1.47	0.0601	1.
Parabacteroides_distasonis	All Crohn's Disease	-0.894	1.21	0.708	1.
Parabacteroides_distasonis	All Ulcerative Colitis	-0.357	1.2	0.469	1.
Parabacteroides_goldsteinii	All Crohn's Disease	-1.78	1.65	0.281	1.
Parabacteroides_goldsteinii	All Ulcerative Colitis	-0.863	1.35	0.523	1.
Parabacteroides_merdae	All Crohn's Disease	0.38	1.12	0.929	1.
Parabacteroides_merdae	All Ulcerative Colitis	-1.11	1.13	0.547	1.
Paraprevotella_clara	All Crohn's Disease	-0.871	1.35	0.767	1.
Paraprevotella_clara	All Ulcerative Colitis	5.29×10^{-03}	1.21	0.889	1.
Parasutterella_excrementihominis	All Crohn's Disease	-0.573	1.21	0.0917	1.
Parasutterella_excrementihominis	All Ulcerative Colitis	-2.43	1.26	0.105	1.
Parasutterella_SGB9260	All Crohn's Disease	-1.77	1.65	0.284	1.
Parasutterella_SGB9260	All Ulcerative Colitis	-1.7	1.61	0.289	1.
Parvimonas_micra	All Crohn's Disease	1.03	2.17	0.636	1.
Parvimonas_micra	All Ulcerative Colitis	1.7	2.09	0.415	1.
Parvimonas_sp_KA00067	All Crohn's Disease	1.69	2.12	0.425	1.
Parvimonas_sp_KA00067	All Ulcerative Colitis	0.27	2.28	0.906	1.
Pauljensenia_hongkongensis	All Crohn's Disease	0.894	2.18	0.681	1.
Pauljensenia_hongkongensis	All Ulcerative Colitis	0.808	2.18	0.711	1.
Pediococcus_acidilactici	All Crohn's Disease	-1.51×10^{-04}	2.36	1.	1.
Pediococcus_acidilactici	All Ulcerative Colitis	2.07	2.08	0.32	1.
Peptoniphilus_coxii	All Crohn's Disease	-1.8	1.64	0.272	1.
Peptoniphilus_coxii	All Ulcerative Colitis	-1.07	1.35	0.427	1.
Peptoniphilus_harei	All Crohn's Disease	-0.0818	2.37	0.972	1.
Peptoniphilus_harei	All Ulcerative Colitis	2.2	2.08	0.29	1.
Peptostreptococcus_anaerobius	All Crohn's Disease	1.08	2.17	0.618	1.
Peptostreptococcus_anaerobius	All Ulcerative Colitis	0.445	2.24	0.842	1.
Peptostreptococcus_SGB749	All Crohn's Disease	-0.179	2.38	0.94	1.
Peptostreptococcus_SGB749	All Ulcerative Colitis	1.31	2.15	0.543	1.
Peptostreptococcus_stomatis	All Crohn's Disease	1.02	2.17	0.637	1.
Peptostreptococcus_stomatis	All Ulcerative Colitis	2.04	2.08	0.326	1.
Phascolarctobacterium_faecium	All Crohn's Disease	1.06	2.17	0.623	1.
Phascolarctobacterium_faecium	All Ulcerative Colitis	1.53	2.11	0.468	1.
Phascolarctobacterium_succinatutens	All Crohn's Disease	-0.244	1.26	0.847	1.
Phascolarctobacterium_succinatutens	All Ulcerative Colitis	-1.8	1.53	0.242	1.
Phocaeicola_dorei	All Crohn's Disease	1.99	2.1	0.343	1.
Phocaeicola_dorei	All Ulcerative Colitis	2.18	2.08	0.295	1.
Phocaeicola_massiliensis	All Crohn's Disease	1.98	2.1	0.346	1.

Phocaeicola_massiliensis	All Ulcerative Colitis	2.19	2.08	0.292	1.
Phocaeicola_vulgatus	All Crohn's Disease	-2.05	2.1	0.548	1.
Phocaeicola_vulgatus	All Ulcerative Colitis	-2.02	2.08	0.546	1.
Phocea_massiliensis	All Crohn's Disease	0.586	1.21	0.862	1.
Phocea_massiliensis	All Ulcerative Colitis	-0.0719	1.21	0.885	1.
Prevotella_buccae	All Crohn's Disease	0.913	2.17	0.674	1.
Prevotella_buccae	All Ulcerative Colitis	0.848	2.17	0.696	1.
Proteus_mirabilis	All Crohn's Disease	-0.283	2.39	0.906	1.
Proteus_mirabilis	All Ulcerative Colitis	1.67	2.16	0.439	1.
Pseudoflavonifractor_capillosus	All Crohn's Disease	1.09	2.17	0.615	1.
Pseudoflavonifractor_capillosus	All Ulcerative Colitis	0.347	2.26	0.878	1.
Pseudoflavonifractor_SGB15156	All Crohn's Disease	1.55	2.12	0.465	1.
Pseudoflavonifractor_SGB15156	All Ulcerative Colitis	1.43	2.11	0.498	1.
Pseudoruminococcus_massiliensis	All Crohn's Disease	-0.0692	2.37	0.977	1.
Pseudoruminococcus_massiliensis	All Ulcerative Colitis	0.899	2.15	0.676	1.
Romboutsia_timonensis	All Crohn's Disease	-2.19	2.11	0.509	1.
Romboutsia_timonensis	All Ulcerative Colitis	-1.92	2.11	0.446	1.
Roseburia_faecis	All Crohn's Disease	-0.682	1.22	0.82	1.
Roseburia_faecis	All Ulcerative Colitis	-0.883	1.2	0.569	1.
Roseburia_hominis	All Crohn's Disease	1.68	1.23	0.313	1.
Roseburia_hominis	All Ulcerative Colitis	0.266	1.19	0.856	1.
Roseburia_intestinalis	All Crohn's Disease	-3.05	2.11	0.275	1.
Roseburia_intestinalis	All Ulcerative Colitis	-1.79	2.13	0.641	1.
Roseburia_inulinivorans	All Crohn's Disease	-0.275	1.23	0.914	1.
Roseburia_inulinivorans	All Ulcerative Colitis	-0.917	1.19	0.688	1.
Roseburia_sp_AF02_12	All Crohn's Disease	-0.26	1.26	0.973	1.
Roseburia_sp_AF02_12	All Ulcerative Colitis	-0.0239	1.21	0.117	1.
Rothia_dentocariosa	All Crohn's Disease	-0.0296	2.36	0.99	1.
Rothia_dentocariosa	All Ulcerative Colitis	1.8	2.09	0.387	1.
Rothia_mucilaginosa	All Crohn's Disease	2.39	2.09	0.252	1.
Rothia_mucilaginosa	All Ulcerative Colitis	2.99	2.07	0.149	1.
Ruminococcaceae_bacterium	All Crohn's Disease	0.668	1.14	0.806	1.
Ruminococcaceae_bacterium	All Ulcerative Colitis	-0.582	1.12	0.587	1.
Ruminococcaceae_unclassified_SGB15234	All Crohn's Disease	0.912	2.17	0.675	1.
Ruminococcaceae_unclassified_SGB15234	All Ulcerative Colitis	0.848	2.17	0.696	1.
Ruminococcaceae_unclassified_SGB15236	All Crohn's Disease	0.162	1.23	0.308	1.
Ruminococcaceae_unclassified_SGB15236	All Ulcerative Colitis	-1.78	1.53	0.245	1.
Ruminococcaceae_unclassified_SGB15265	All Crohn's Disease	0.539	1.21	0.427	1.
Ruminococcaceae_unclassified_SGB15265	All Ulcerative Colitis	5.55×10^{-03}	1.21	0.644	1.
Ruminococcaceae_unclassified_SGB4191	All Crohn's Disease	1.05	2.17	0.629	1.
Ruminococcaceae_unclassified_SGB4191	All Ulcerative Colitis	0.702	2.18	0.748	1.
Ruminococcus_bicirculans	All Crohn's Disease	-0.0917	1.12	0.314	1.
Ruminococcus_bicirculans	All Ulcerative Colitis	0.0881	1.11	0.725	1.
Ruminococcus_bromii	All Crohn's Disease	-0.261	1.23	0.407	1.
Ruminococcus_bromii	All Ulcerative Colitis	-1.24	1.19	0.503	1.
Ruminococcus_callidus	All Crohn's Disease	0.172	1.23	0.607	1.
Ruminococcus_callidus	All Ulcerative Colitis	-0.0102	1.21	0.219	1.
Ruminococcus_gnavus	All Crohn's Disease	0.755	1.35	0.673	1.
Ruminococcus_gnavus	All Ulcerative Colitis	0.821	1.33	0.787	1.
Ruminococcus_lactaris	All Crohn's Disease	-0.509	1.31	0.865	1.
Ruminococcus_lactaris	All Ulcerative Colitis	-0.801	1.42	0.817	1.
Ruminococcus_SGB4421	All Crohn's Disease	0.992	2.17	0.647	1.
Ruminococcus_SGB4421	All Ulcerative Colitis	0.0432	2.29	0.985	1.
Ruminococcus_sp_AF41_9	All Crohn's Disease	-1.82	1.64	0.269	1.
Ruminococcus_sp_AF41_9	All Ulcerative Colitis	-1.01	1.33	0.447	1.
Ruminococcus_sp_NSJ_71	All Crohn's Disease	1.	2.17	0.643	1.
Ruminococcus_sp_NSJ_71	All Ulcerative Colitis	1.77	2.09	0.397	1.
Ruminococcus_torques	All Crohn's Disease	-9.07×10^{-03}	2.36	0.526	1.
Ruminococcus_torques	All Ulcerative Colitis	-1.32	2.11	0.169	1.
Ruthenibacterium_lactatiformans	All Crohn's Disease	-1.08	2.17	0.854	1.
Ruthenibacterium_lactatiformans	All Ulcerative Colitis	-3.05	2.08	0.263	1.
Scardovia_wiggsiae	All Crohn's Disease	-0.828	1.34	0.538	1.

Scardovia_wiggsiae	All Ulcerative Colitis	-0.434	1.24	0.727	1.
Schaalia_turicensis	All Crohn's Disease	1.76	2.14	0.411	1.
Schaalia_turicensis	All Ulcerative Colitis	0.371	2.28	0.871	1.
Sellimonas_intestinalis	All Crohn's Disease	-1.87	1.25	0.251	1.
Sellimonas_intestinalis	All Ulcerative Colitis	-1.19	1.22	0.54	1.
Slackia_exigua	All Crohn's Disease	0.987	2.17	0.649	1.
Slackia_exigua	All Ulcerative Colitis	0.895	2.15	0.677	1.
Slackia_isoflavoniconvertens	All Crohn's Disease	-1.83	1.64	0.265	1.
Slackia_isoflavoniconvertens	All Ulcerative Colitis	-0.457	1.25	0.714	1.
Slackia_piriformis	All Crohn's Disease	-1.85	1.27	0.146	1.
Slackia_piriformis	All Ulcerative Colitis	-1.9	1.25	0.128	1.
Solobacterium_SGB6833	All Crohn's Disease	-0.104	1.13	0.134	1.
Solobacterium_SGB6833	All Ulcerative Colitis	-0.228	1.12	0.714	1.
Staphylococcus_aureus	All Crohn's Disease	0.986	2.17	0.649	1.
Staphylococcus_aureus	All Ulcerative Colitis	1.41	2.11	0.503	1.
Streptococcus_anginosus	All Crohn's Disease	-1.14	1.17	0.553	1.
Streptococcus_anginosus	All Ulcerative Colitis	-0.865	1.12	0.687	1.
Streptococcus_constellatus	All Crohn's Disease	0.0761	2.37	0.974	1.
Streptococcus_constellatus	All Ulcerative Colitis	0.756	2.21	0.733	1.
Streptococcus_cristatus	All Crohn's Disease	-0.806	1.34	0.548	1.
Streptococcus_cristatus	All Ulcerative Colitis	-0.485	1.25	0.698	1.
Streptococcus_gordonii	All Crohn's Disease	-1.78	1.23	0.18	1.
Streptococcus_gordonii	All Ulcerative Colitis	-0.594	1.2	0.856	1.
Streptococcus_infantis	All Crohn's Disease	0.291	1.12	0.801	1.
Streptococcus_infantis	All Ulcerative Colitis	0.662	1.12	0.745	1.
Streptococcus_intermedius	All Crohn's Disease	-0.298	1.27	0.814	1.
Streptococcus_intermedius	All Ulcerative Colitis	-0.932	1.32	0.48	1.
Streptococcus_lutetiensis	All Crohn's Disease	0.994	2.17	0.646	1.
Streptococcus_lutetiensis	All Ulcerative Colitis	1.79	2.09	0.391	1.
Streptococcus_mitis	All Crohn's Disease	-2.16	2.11	0.513	1.
Streptococcus_mitis	All Ulcerative Colitis	-0.782	2.2	0.394	1.
Streptococcus_mutans	All Crohn's Disease	1.58	2.12	0.457	1.
Streptococcus_mutans	All Ulcerative Colitis	1.39	2.11	0.508	1.
Streptococcus_oralis	All Crohn's Disease	-0.426	1.12	0.913	1.
Streptococcus_oralis	All Ulcerative Colitis	1.31	1.17	0.461	1.
Streptococcus_parasanguinis	All Crohn's Disease	-1.1	2.17	0.716	1.
Streptococcus_parasanguinis	All Ulcerative Colitis	-0.81	2.2	0.917	1.
Streptococcus_pneumoniae	All Crohn's Disease	1.01	2.17	0.642	1.
Streptococcus_pneumoniae	All Ulcerative Colitis	0.86	2.15	0.689	1.
Streptococcus_rubneri	All Crohn's Disease	0.994	2.17	0.646	1.
Streptococcus_rubneri	All Ulcerative Colitis	0.886	2.15	0.68	1.
Streptococcus_salivarius	All Crohn's Disease	-1.08	2.17	0.856	1.
Streptococcus_salivarius	All Ulcerative Colitis	-0.514	2.22	0.967	1.
Streptococcus_sanguinis	All Crohn's Disease	-0.339	1.25	0.013	1.
Streptococcus_sp_263_SSPC	All Crohn's Disease	-0.0996	1.13	0.934	1.
Streptococcus_sp_263_SSPC	All Ulcerative Colitis	1.13	1.18	0.562	1.
Streptococcus_sp_A12	All Crohn's Disease	1.2	1.26	0.478	1.
Streptococcus_sp_A12	All Ulcerative Colitis	1.86	1.35	0.308	1.
Streptococcus_thermophilus	All Crohn's Disease	-1.03	2.17	0.0348	1.
Streptococcus_thermophilus	All Ulcerative Colitis	-1.32	2.11	0.0901	1.
Sutterella_wadsworthensis	All Crohn's Disease	-0.193	1.26	0.936	1.
Sutterella_wadsworthensis	All Ulcerative Colitis	-0.237	1.24	0.943	1.
TM7_phylum_sp_oral_taxon_348	All Crohn's Disease	-0.357	1.28	0.78	1.
TM7_phylum_sp_oral_taxon_348	All Ulcerative Colitis	-1.03	1.37	0.451	1.
Trueperella_pyogenes	All Crohn's Disease	-1.36	1.21	0.258	1.
Trueperella_pyogenes	All Ulcerative Colitis	-1.56	1.19	0.344	1.
Turicibacter_sanguinis	All Crohn's Disease	0.295	1.12	0.765	1.
Turicibacter_sanguinis	All Ulcerative Colitis	0.036	1.1	0.33	1.
Tyzzerella_nexilis	All Crohn's Disease	-1.22	1.17	0.393	1.
Tyzzerella_nexilis	All Ulcerative Colitis	-0.632	1.1	0.301	1.
Veillonella_atypica	All Crohn's Disease	-0.238	1.26	0.86	1.
Veillonella_atypica	All Ulcerative Colitis	0.259	1.19	0.805	1.

Veillonella_dispar	All Crohn's Disease	0.193	1.23	0.381	1.
Veillonella_dispar	All Ulcerative Colitis	0.284	1.19	0.8	1.
Veillonella_parvula	All Crohn's Disease	1.7	1.23	0.302	1.
Veillonella_parvula	All Ulcerative Colitis	1.04	1.18	0.614	1.
Veillonella_rogosae	All Crohn's Disease	1.06	2.17	0.625	1.
Veillonella_rogosae	All Ulcerative Colitis	0.643	2.19	0.769	1.
