# Supplementary material for: Multiomics-Based Profiling of the Fecal Microbiome Reveals Potential Disease-Specific Signatures in Pediatric IBD (PIBD)
Source: Biomolecules. 2025 May 21;15(5):746. doi: 10.3390/biom15050746 (PMC12109367; doi:10.3390/biom15050746)
Supplement: Supplementary file 1 [file biomolecules-15-00746-s001.zip › supplemental8-maaslin-diseasegroup-pathwayabundance.pdf]

Supplemental Table S8

Differential abundance of MetaCyc functional pathways in UC and Crohn's as compared to healthy microbiome, as calculated by MaAsLin3.

Pathway	Condition	Effect Size	Standard Error	P-Value	Q-Value
PWY4LZ.257..superpathway.of.fermentation..Chlamydomonas.reinhardtii.	All Ulcerative Colitis	3.15	0.719	2.55×10^{-04}	0.224
P161.PWY..acetylene.degradation..anaerobic.	All Ulcerative Colitis	3.23	0.828	8.38×10^{-04}	0.306
PWY.702..L.methionine.biosynthesis.II	All Crohn's Disease	-1.16	0.363	5.96×10^{-03}	0.367
PWY.I9..L.cysteine.biosynthesis.VI..from.L.methionine.	All Crohn's Disease	-1.18	0.393	8.95×10^{-03}	0.415
P161.PWY..acetylene.degradation..anaerobic.	All Crohn's Disease	2.18	0.85	0.0139	0.626
PWY4LZ.257..superpathway.of.fermentation..Chlamydomonas.reinhardtii.	All Crohn's Disease	1.76	0.738	0.0205	0.767
PWY0.1477..ethanolamine.utilization	All Ulcerative Colitis	1.79	0.711	0.0244	0.857
GLYCOL.GLYOXDEG.PWY..superpathway.of.glycol.metabolism.and.degradation	All Ulcerative Colitis	5.17	1.36	0.027	0.875
PWY.6902..chitin.degradation.II..Vibrio.	All Ulcerative Colitis	-1.75	0.685	0.0254	0.875
PWY.7204..pyridoxal.5..phosphate.salvage.II..plants.	All Ulcerative Colitis	5.73	1.89	0.0269	0.875
PWY0.1298..superpathway.of.pyrimidine.deoxyribonucleosides.degradation	All Ulcerative Colitis	1.74	0.7	0.0261	0.875
PWY.5130..2.oxobutanoate.degradation.I	All Crohn's Disease	1.74	0.682	0.0309	0.901
PWY.7383..anaerobic.energy.metabolism..invertebrates..cytosol.	All Ulcerative Colitis	-1.46	0.587	0.0296	0.901
PWY.7616..methanol.oxidation.to.carbon.dioxide	All Ulcerative Colitis	3.08	0.0715	0.0303	0.901
HEME.BIOSYNTHESIS.II.1..heme.b.biosynthesis.V..aerobic.	All Ulcerative Colitis	3.3	1.17	0.0326	0.903
PWY66.399..gluconeogenesis.III	All Ulcerative Colitis	-1.09	0.456	0.0328	0.903
PWY.5136..fatty.acid..beta..oxidation.II..plant.peroxisome.	All Ulcerative Colitis	1.81	0.777	0.0357	0.948
ALLANTOINDEG.PWY..superpathway.of.allantoin.degradation.in.yeast	All Crohn's Disease	1.05	1.1	0.489	1.
ALLANTOINDEG.PWY..superpathway.of.allantoin.degradation.in.yeast	All Ulcerative Colitis	0.138	1.06	0.812	1.
ANAEROFRUCAT.PWY..homolactic.fermentation	All Crohn's Disease	-0.697	0.319	0.0599	1.
ANAEROFRUCAT.PWY..homolactic.fermentation	All Ulcerative Colitis	-0.161	0.31	0.427	1.
ANAGLYCOLYSIS.PWY..glycolysis.III..from.glucose.	All Crohn's Disease	-0.175	0.245	1.	1.
ANAGLYCOLYSIS.PWY..glycolysis.III..from.glucose.	All Ulcerative Colitis	-0.288	0.239	0.127	1.
ARG.POLYAMINE.SYN..superpathway.of.arginine.and.polyamine.biosynthesis	All Crohn's Disease	0.831	0.608	0.151	1.
ARG.POLYAMINE.SYN..superpathway.of.arginine.and.polyamine.biosynthesis	All Ulcerative Colitis	0.995	0.592	0.139	1.
ARGININE.SYN4.PWY..L.ornithine.biosynthesis.II	All Crohn's Disease	0.279	0.81	0.892	1.
ARGININE.SYN4.PWY..L.ornithine.biosynthesis.II	All Ulcerative Colitis	-0.0317	0.803	0.904	1.
ARGSYN.PWY..L.arginine.biosynthesis.I..via.L.ornithine.	All Crohn's Disease	-0.244	0.3	1.	1.
ARGSYN.PWY..L.arginine.biosynthesis.I..via.L.ornithine.	All Ulcerative Colitis	-0.374	0.292	0.126	1.
ARGSYNBSUB.PWY..L.arginine.biosynthesis.II..acetyl.cycle.	All Crohn's Disease	-0.247	0.307	1.	1.
ARGSYNBSUB.PWY..L.arginine.biosynthesis.II..acetyl.cycle.	All Ulcerative Colitis	-0.366	0.299	0.141	1.
ARO.PWY..chorismate.biosynthesis.I	All Crohn's Disease	-0.201	0.261	1.	1.
ARO.PWY..chorismate.biosynthesis.I	All Ulcerative Colitis	-0.376	0.254	0.0794	1.
ASPASN.PWY..superpathway.of.L.aspartate.and.L.asparagine.biosynthesis	All Crohn's Disease	-0.3	0.262	1.	1.
ASPASN.PWY..superpathway.of.L.aspartate.and.L.asparagine.biosynthesis	All Ulcerative Colitis	-0.0954	0.256	1.	1.
AST.PWY..L.arginine.degradation.II..AST.pathway.	All Crohn's Disease	1.28	2.45	0.846	1.
AST.PWY..L.arginine.degradation.II..AST.pathway.	All Ulcerative Colitis	4.08	2.34	0.274	1.
BIOTIN.BIOSYNTHESIS.PWY..biotin.biosynthesis.I	All Crohn's Disease	0.131	0.667	1.	1.
BIOTIN.BIOSYNTHESIS.PWY..biotin.biosynthesis.I	All Ulcerative Colitis	0.526	0.649	0.507	1.
BRANCHED.CHAIN.AA.SYN.PWY superpathway.of.branched.chain.amino.acid.biosynthesis	All Crohn's Disease	-0.316	0.288	1.	1.
BRANCHED.CHAIN.AA.SYN.PWY superpathway.of.branched.chain.amino.acid.biosynthesis	All Ulcerative Colitis	-0.362	0.281	0.121	1.
CALVIN.PWY..Calvin.Benson.Bassham.cycle	All Crohn's Disease	-0.0113	0.232	1.	1.
CALVIN.PWY..Calvin.Benson.Bassham.cycle	All Ulcerative Colitis	-0.109	0.226	1.	1.
CENTFERM.PWY..pyruvate.fermentation.to.butanoate	All Crohn's Disease	-0.0473	0.728	0.874	1.
CENTFERM.PWY..pyruvate.fermentation.to.butanoate	All Ulcerative Colitis	-0.242	0.716	0.76	1.
CITRULBIO.PWY..L.citrulline.biosynthesis	All Crohn's Disease	0.606	0.767	0.388	1.
CITRULBIO.PWY..L.citrulline.biosynthesis	All Ulcerative Colitis	0.0678	0.747	1.	1.
COA.PWY..coenzyme.A.biosynthesis.I..prokaryotic.	All Crohn's Disease	-0.073	0.24	1.	1.
COA.PWY..coenzyme.A.biosynthesis.I..prokaryotic.	All Ulcerative Colitis	-0.294	0.234	0.113	1.
COA.PWY.1..superpathway.of.coenzyme.A.biosynthesis.III..mammals.	All Crohn's Disease	-0.0689	0.245	1.	1.
COA.PWY.1..superpathway.of.coenzyme.A.biosynthesis.III..mammals.	All Ulcerative Colitis	-0.295	0.239	0.12	1.
COBALSYN.PWY..superpathway.of.adenosylcobalamin.salvage.from.cobinamide.I	All Crohn's Disease	-0.553	0.434	0.275	1.
COBALSYN.PWY..superpathway.of.adenosylcobalamin.salvage.from.cobinamide.I	All Ulcerative Colitis	-0.497	0.423	0.178	1.
COLANSYN.PWY..colanic.acid.building.blocks.biosynthesis	All Crohn's Disease	-0.0474	0.279	1.	1.
COLANSYN.PWY..colanic.acid.building.blocks.biosynthesis	All Ulcerative Colitis	0.154	0.272	1.	1.
COMPLETE.ARO.PWY..superpathway.of.aromatic.amino.acid.biosynthesis	All Crohn's Disease	-0.218	0.27	1.	1.
COMPLETE.ARO.PWY..superpathway.of.aromatic.amino.acid.biosynthesis	All Ulcerative Colitis	-0.393	0.263	0.0786	1.
CRNFORCAT.PWY..creatinine.degradation.I	All Crohn's Disease	-0.0192	0.821	0.988	1.
DAPLYSINESYN.PWY..L.lysine.biosynthesis.I	All Crohn's Disease	-0.78	0.453	0.129	1.
DAPLYSINESYN.PWY..L.lysine.biosynthesis.I	All Ulcerative Colitis	0.088	0.441	1.	1.
DTDPRHAMSYN.PWY..dTDP..beta..L.rhamnose.biosynthesis	All Crohn's Disease	-0.159	0.331	1.	1.
DTDPRHAMSYN.PWY..dTDP..beta..L.rhamnose.biosynthesis	All Ulcerative Colitis	-0.481	0.322	0.0892	1.
ECASYN.PWY..enterobacterial.common.antigen.biosynthesis	All Crohn's Disease	2.78	2.5	0.506	1.
ECASYN.PWY..enterobacterial.common.antigen.biosynthesis	All Ulcerative Colitis	2.83	2.34	0.49	1.
FAO.PWY..fatty.acid..beta..oxidation.I..generic.	All Crohn's Disease	1.16	2.78	0.675	1.
FAO.PWY..fatty.acid..beta..oxidation.I..generic.	All Ulcerative Colitis	2.64	2.73	0.592	1.
FASYN.ELONG.PWY..fatty.acid.elongation....saturated	All Crohn's Disease	0.128	0.76	1.	1.
FASYN.ELONG.PWY..fatty.acid.elongation....saturated	All Ulcerative Colitis	1.03	0.74	0.214	1.
FASYN.INITIAL.PWY..superpathway.of.fatty.acid.biosynthesis.initiation..E..coli.	All Crohn's Disease	-1.98	0.876	0.0876	1.
FASYN.INITIAL.PWY..superpathway.of.fatty.acid.biosynthesis.initiation..E..coli.	All Ulcerative Colitis	1.07	0.897	0.409	1.
FERMENTATION.PWY..mixed.acid.fermentation	All Crohn's Disease	-0.262	0.49	1.	1.
FERMENTATION.PWY..mixed.acid.fermentation	All Ulcerative Colitis	-0.274	0.477	0.453	1.

FOLSYN.PWY..superpathway.of.tetrahydrofolate.biosynthesis.and.salvage	All Crohn's Disease	0.163	0.459	0.999	1.
FOLSYN.PWY..superpathway.of.tetrahydrofolate.biosynthesis.and.salvage	All Ulcerative Colitis	-0.0121	0.449	0.991	1.
FUC.RHAMCAT.PWY..superpathway.of.fucose.and.rhamnose.degradation	All Crohn's Disease	0.0256	0.86	1.	1.
FUC.RHAMCAT.PWY..superpathway.of.fucose.and.rhamnose.degradation	All Ulcerative Colitis	0.349	0.837	0.759	1.
FUCCAT.PWY..fucose.degradation	All Crohn's Disease	0.137	0.715	1.	1.
FUCCAT.PWY..fucose.degradation	All Ulcerative Colitis	0.14	0.697	1.	1.
GALACT.GLUCUROCAT.PWY superpathway.of.hexuronide.and.hexuronate.degradation	All Crohn's Disease	0.725	0.644	0.407	1.
GALACT.GLUCUROCAT.PWY superpathway.of.hexuronide.and.hexuronate.degradation	All Ulcerative Colitis	1.5	0.63	0.0682	1.
GALACTARDEG.PWY..D.galactarate.degradation.I	All Crohn's Disease	0.804	1.24	0.737	1.
GALACTARDEG.PWY..D.galactarate.degradation.I	All Ulcerative Colitis	1.19	1.18	0.592	1.
GALACTITOLCAT.PWY..galactitol.degradation	All Crohn's Disease	0.908	1.82	0.839	1.
GALACTITOLCAT.PWY..galactitol.degradation	All Ulcerative Colitis	0.999	1.68	0.837	1.
GALACTUROCAT.PWY..D.galacturonate.degradation.I	All Crohn's Disease	0.646	0.56	0.213	1.
GALACTUROCAT.PWY..D.galacturonate.degradation.I	All Ulcerative Colitis	0.275	0.545	1.	1.
GLCMANNANAUT.PWYsuperpathway.of.N.acetylglucosamine..N.acetylmannosamine and.N.acetylneuraminate.degradation	All Crohn's Disease	0.0784	0.358	1.	1.
GLCMANNANAUT.PWY..superpathway.of.N.acetylglucosamine..N.acetylmannosamine and.N.acetylneuraminate.degradation	All Ulcerative Colitis	0.171	0.349	1.	1.
GLUCARDEG.PWY..D.glucarate.degradation.I	All Crohn's Disease	1.1	1.1	0.51	1.
GLUCARDEG.PWY..D.glucarate.degradation.I	All Ulcerative Colitis	1.38	1.06	0.415	1.
GLUCARGALACTSUPER.PWY superpathway.of.D.glucarate.and.D.galactarate.degradation	All Crohn's Disease	0.804	1.24	0.737	1.
GLUCARGALACTSUPER.PWY superpathway.of.D.glucarate.and.D.galactarate.degradation	All Ulcerative Colitis	1.19	1.18	0.592	1.
GLUCONEO.PWY..gluconeogenesis.I	All Crohn's Disease	-0.28	0.253	1.	1.
GLUCONEO.PWY..gluconeogenesis.I	All Ulcerative Colitis	-0.242	0.247	0.191	1.
GLUCOSE1PMETAB.PWY..glucose.and.glucose.1.phosphate.degradation	All Crohn's Disease	-0.365	0.436	0.756	1.
GLUCOSE1PMETAB.PWY..glucose.and.glucose.1.phosphate.degradation	All Ulcerative Colitis	-0.31	0.436	0.605	1.
GLUCUROCAT.PWY..superpathway.of..beta..D.glucuronosides.degradation	All Crohn's Disease	0.286	0.485	0.471	1.
GLUCUROCAT.PWY..superpathway.of..beta..D.glucuronosides.degradation	All Ulcerative Colitis	-0.14	0.472	1.	1.
GLUDEG.I.PWY..GABA.shunt	All Crohn's Disease	0.721	0.912	0.634	1.
GLUDEG.I.PWY..GABA.shunt	All Ulcerative Colitis	-0.272	0.893	0.904	1.
GLUTORN.PWY..L.ornithine.biosynthesis.I	All Crohn's Disease	-0.268	0.346	1.	1.
GLUTORN.PWY..L.ornithine.biosynthesis.I	All Ulcerative Colitis	-0.444	0.337	0.126	1.
GLYCOCAT.PWY..glycogen.degradation.I	All Crohn's Disease	-0.876	0.927	0.631	1.
GLYCOCAT.PWY..glycogen.degradation.I	All Ulcerative Colitis	0.214	0.907	0.991	1.
GLYCOGENSYNTH.PWY..glycogen.biosynthesis.I..from.ADP.D.Glucose.	All Crohn's Disease	-0.204	0.332	1.	1.
GLYCOGENSYNTH.PWY..glycogen.biosynthesis.I..from.ADP.D.Glucose.	All Ulcerative Colitis	-0.341	0.323	0.195	1.
GLYCOL.GLYOXDEG.PWY..superpathway.of.glycol.metabolism.and.degradation	All Crohn's Disease	3.66	1.44	0.0944	1.
GLYCOLYSIS..glycolysis.I..from.glucose.6.phosphate.	All Crohn's Disease	-0.688	0.337	0.078	1.
GLYCOLYSIS..glycolysis.I..from.glucose.6.phosphate.	All Ulcerative Colitis	-0.0808	0.328	1.	1.
GLYCOLYSIS.E.D..superpathway.of.glycolysis.and.the.Entner.Doudoroff.pathway	All Crohn's Disease	0.568	0.487	0.203	1.
GLYCOLYSIS.E.D..superpathway.of.glycolysis.and.the.Entner.Doudoroff.pathway	All Ulcerative Colitis	0.85	0.475	0.121	1.
GLYOXYLATE.BYPASS..glyoxylate.cycle	All Crohn's Disease	-1.35	2.22	0.0867	1.
GLYOXYLATE.BYPASS..glyoxylate.cycle	All Ulcerative Colitis	0.454	2.25	0.428	1.
GOLPDLCAT.PWY..superpathway.of.glycerol.degradation.to.1.3.propanediol	All Crohn's Disease	-0.428	0.588	0.795	1.
GOLPDLCAT.PWY..superpathway.of.glycerol.degradation.to.1.3.propanediol	All Ulcerative Colitis	-0.61	0.575	0.416	1.
HCAMHPDEG.PWY..3.phenylpropanoate.and 3..3.hydroxyphenyl.propanoate.degradation.to.2.hydroxypentadienoate	All Crohn's Disease	0.838	3.35	0.96	1.
HCAMHPDEG.PWY..3.phenylpropanoate.and 3..3.hydroxyphenyl.propanoate.degradation.to.2.hydroxypentadienoate	All Ulcerative Colitis	1.54	3.23	0.896	1.
HEME.BIOSYNTHESIS.II..heme.b.biosynthesis.I..aerobic.	All Crohn's Disease	0.59	1.11	0.805	1.
HEME.BIOSYNTHESIS.II..heme.b.biosynthesis.I..aerobic.	All Ulcerative Colitis	1.2	1.09	0.533	1.
HEME.BIOSYNTHESIS.II.1..heme.b.biosynthesis.V..aerobic.	All Crohn's Disease	1.68	1.22	0.317	1.
HEMESYN2.PWY..heme.b.biosynthesis.II..oxygen.independent.	All Crohn's Disease	0.924	0.696	0.166	1.
HEMESYN2.PWY..heme.b.biosynthesis.II..oxygen.independent.	All Ulcerative Colitis	1.44	0.678	0.058	1.
HEXITOLDEGSUPER.PWY..superpathway.of.hexitol.degradation..bacteria.	All Crohn's Disease	1.05	1.39	0.678	1.
HEXITOLDEGSUPER.PWY..superpathway.of.hexitol.degradation..bacteria.	All Ulcerative Colitis	1.24	1.29	0.619	1.
HISDEG.PWY..L.histidine.degradation.I	All Crohn's Disease	0.144	0.586	1.	1.
HISDEG.PWY..L.histidine.degradation.I	All Ulcerative Colitis	-0.376	0.571	0.423	1.
HISTSYN.PWY..L.histidine.biosynthesis	All Crohn's Disease	-0.318	0.308	1.	1.
HISTSYN.PWY..L.histidine.biosynthesis	All Ulcerative Colitis	-0.436	0.3	0.0924	1.
HOMOSER.METSYN.PWY..L.methionine.biosynthesis.I	All Crohn's Disease	1.15	1.1	0.459	1.
HOMOSER.METSYN.PWY..L.methionine.biosynthesis.I	All Ulcerative Colitis	2.16	1.09	0.137	1.
HSERMETANA.PWY..L.methionine.biosynthesis.III	All Crohn's Disease	-0.555	0.307	0.126	1.
HSERMETANA.PWY..L.methionine.biosynthesis.III	All Ulcerative Colitis	-0.369	0.299	0.138	1.
ILEUSYN.PWY..L.isoleucine.biosynthesis.I..from.threonine.	All Crohn's Disease	-0.325	0.287	0.38	1.
ILEUSYN.PWY..L.isoleucine.biosynthesis.I..from.threonine.	All Ulcerative Colitis	-0.356	0.279	0.123	1.
KETOGLUCONMET.PWY..ketogluconate.metabolism	All Crohn's Disease	1.9	1.95	0.554	1.
KETOGLUCONMET.PWY..ketogluconate.metabolism	All Ulcerative Colitis	2.64	1.86	0.353	1.
LACTOSECAT.PWY..lactose.and.galactose.degradation.I	All Crohn's Disease	0.17	0.771	1.	1.
LACTOSECAT.PWY..lactose.and.galactose.degradation.I	All Ulcerative Colitis	0.576	0.751	0.523	1.
LIPA.CORESYN.PWY..lipid.A.core.biosynthesis..E..coli.K.12.	All Crohn's Disease	4.67	2.13	0.287	1.
LIPA.CORESYN.PWY..lipid.A.core.biosynthesis..E..coli.K.12.	All Ulcerative Colitis	3.81	1.61	0.272	1.
LIPASYN.PWY..phospholipases	All Crohn's Disease	-0.0472	3.47	0.975	1.
LIPASYN.PWY..phospholipases	All Ulcerative Colitis	2.58	3.26	0.75	1.
MET.SAM.PWY..superpathway.of.S.adenosyl.L.methionine.biosynthesis	All Crohn's Disease	1.1	1.06	0.459	1.
MET.SAM.PWY..superpathway.of.S.adenosyl.L.methionine.biosynthesis	All Ulcerative Colitis	2.05	1.04	0.142	1.

METH.ACETATE.PWY..methanogenesis.from.acetate	All Crohn's Disease	-0.607	0.617	0.63	1.
METH.ACETATE.PWY..methanogenesis.from.acetate	All Ulcerative Colitis	-0.509	0.603	0.553	1.
METHGLYUT.PWY..superpathway.of.methylglyoxal.degradation	All Crohn's Disease	1.13	0.95	0.394	1.
METHGLYUT.PWY..superpathway.of.methylglyoxal.degradation	All Ulcerative Colitis	1.55	0.933	0.246	1.
METSYN.PWY..superpathway.of.L.homoserine.and.L.methionine.biosynthesis	All Crohn's Disease	1.12	1.07	0.459	1.
METSYN.PWY..superpathway.of.L.homoserine.and.L.methionine.biosynthesis	All Ulcerative Colitis	2.08	1.06	0.141	1.
NAD.BIOSYNTHESIS.II..NAD.salvage.pathway.III..to.nicotinamide.riboside.	All Crohn's Disease	5.14×10^{-03}	0.88	0.412	1.
NAD.BIOSYNTHESIS.II..NAD.salvage.pathway.III..to.nicotinamide.riboside.	All Ulcerative Colitis	0.274	0.798	0.857	1.
NAGLIPASYN.PWY..lipid.IVA.biosynthesis..E..coli.	All Crohn's Disease	0.33	0.755	0.842	1.
NAGLIPASYN.PWY..lipid.IVA.biosynthesis..E..coli.	All Ulcerative Colitis	0.367	0.746	0.895	1.
NONMEVIPP.PWY..methylerythritol.phosphate.pathway.I	All Crohn's Disease	-0.0761	0.27	1.	1.
NONMEVIPP.PWY..methylerythritol.phosphate.pathway.I	All Ulcerative Colitis	-0.33	0.263	0.124	1.
NONOXIPENT.PWY..pentose.phosphate.pathway..non.oxidative.branch..I	All Crohn's Disease	-0.0594	0.3	1.	1.
NONOXIPENT.PWY..pentose.phosphate.pathway..non.oxidative.branch..I	All Ulcerative Colitis	-0.0823	0.292	1.	1.
OANTIGEN.PWY..O.antigen.building.blocks.biosynthesis..E..coli.	All Crohn's Disease	-0.344	0.279	0.333	1.
OANTIGEN.PWY..O.antigen.building.blocks.biosynthesis..E..coli.	All Ulcerative Colitis	-0.0496	0.271	1.	1.
ORNDEG.PWY..superpathway.of.ornithine.degradation	All Crohn's Disease	0.915	1.32	0.716	1.
ORNDEG.PWY..superpathway.of.ornithine.degradation	All Ulcerative Colitis	2.51	1.31	0.156	1.
P105.PWY..TCA.cycle.IV..2.oxoglutarate.decarboxylase.	All Crohn's Disease	0.407	1.23	0.156	1.
P105.PWY..TCA.cycle.IV..2.oxoglutarate.decarboxylase.	All Ulcerative Colitis	1.69	1.22	0.206	1.
P108.PWY..pyruvate.fermentation.to.propanoate.I	All Crohn's Disease	2.18	1.79	0.406	1.
P108.PWY..pyruvate.fermentation.to.propanoate.I	All Ulcerative Colitis	3.43	1.75	0.147	1.
P122.PWY..heterolactic.fermentation	All Crohn's Disease	0.0237	1.83	0.709	1.
P122.PWY..heterolactic.fermentation	All Ulcerative Colitis	2.27	1.77	0.349	1.
P124.PWY..Bifidobacterium.shunt	All Crohn's Disease	-1.02	1.11	0.639	1.
P124.PWY..Bifidobacterium.shunt	All Ulcerative Colitis	-0.336	1.07	0.687	1.
P125.PWY..superpathway.of..R.R..butanediol.biosynthesis	All Crohn's Disease	-1.32	1.27	0.558	1.
P125.PWY..superpathway.of..R.R..butanediol.biosynthesis	All Ulcerative Colitis	0.273	1.23	0.85	1.
P164.PWY..purine.nucleobases.degradation.I..anaerobic.	All Crohn's Disease	0.161	0.427	1.	1.
P164.PWY..purine.nucleobases.degradation.I..anaerobic.	All Ulcerative Colitis	0.0406	0.416	1.	1.
P185.PWY..formaldehyde.assimilation.III..dihydroxyacetone.cycle.	All Crohn's Disease	-1.39×10^{-03}	0.743	0.999	1.
P185.PWY..formaldehyde.assimilation.III..dihydroxyacetone.cycle.	All Ulcerative Colitis	-0.0798	0.726	0.991	1.
P4.PWY..superpathway.of.L.lysine..L.threonine.and.L.methionine.biosynthesis.I	All Crohn's Disease	0.666	0.81	0.476	1.
P4.PWY..superpathway.of.L.lysine..L.threonine.and.L.methionine.biosynthesis.I	All Ulcerative Colitis	1.49	0.806	0.185	1.
P41.PWY..pyruvate.fermentation.to.acetate.and..S..lactate.I	All Crohn's Disease	-0.494	0.32	0.196	1.
P41.PWY..pyruvate.fermentation.to.acetate.and..S..lactate.I	All Ulcerative Colitis	-0.281	0.311	0.246	1.
P42.PWY..incomplete.reductive.TCA.cycle	All Crohn's Disease	1.7	0.858	0.0989	1.
P42.PWY..incomplete.reductive.TCA.cycle	All Ulcerative Colitis	1.41	0.822	0.227	1.
P441.PWY..superpathway.of.N.acetylneuraminate.degradation	All Crohn's Disease	0.462	0.319	0.205	1.
P441.PWY..superpathway.of.N.acetylneuraminate.degradation	All Ulcerative Colitis	0.67	0.312	0.143	1.
P461.PWY..hexitol.fermentation.to.lactate..formate..ethanol.and.acetate	All Crohn's Disease	0.416	0.914	0.6	1.
P461.PWY..hexitol.fermentation.to.lactate..formate..ethanol.and.acetate	All Ulcerative Colitis	0.0149	0.89	1.	1.
P621.PWY..nylon.6.oligomer.degradation	All Crohn's Disease	0.985	1.09	0.576	1.
P621.PWY..nylon.6.oligomer.degradation	All Ulcerative Colitis	0.497	1.06	0.862	1.
PANTO.PWY..phosphopantothenate.biosynthesis.I	All Crohn's Disease	-0.108	0.265	1.	1.
PANTO.PWY..phosphopantothenate.biosynthesis.I	All Ulcerative Colitis	-0.417	0.258	0.0613	1.
PANTOSYN.PWY..superpathway.of.coenzyme.A.biosynthesis.I..bacteria.	All Crohn's Disease	-0.1	0.246	1.	1.
PANTOSYN.PWY..superpathway.of.coenzyme.A.biosynthesis.I..bacteria.	All Ulcerative Colitis	-0.374	0.24	0.0646	1.
PENTOSE.P.PWY..pentose.phosphate.pathway	All Crohn's Disease	-0.236	0.436	1.	1.
PENTOSE.P.PWY..pentose.phosphate.pathway	All Ulcerative Colitis	0.162	0.425	1.	1.
PEPTIDOGLYCANSYN.PWY peptidoglycan.biosynthesis.I..meso.diaminopimelate.containing.	All Crohn's Disease	-0.154	0.232	1.	1.
PEPTIDOGLYCANSYN.PWY peptidoglycan.biosynthesis.I..meso.diaminopimelate.containing.	All Ulcerative Colitis	-0.28	0.225	0.114	1.
PHOSLIPSYN.PWY..superpathway.of.phospholipid.biosynthesis.I..bacteria.	All Crohn's Disease	0.132	0.487	1.	1.
PHOSLIPSYN.PWY..superpathway.of.phospholipid.biosynthesis.I..bacteria.	All Ulcerative Colitis	-0.47	0.474	0.25	1.
POLYAMINSYN3.PWY..superpathway.of.polyamine.biosynthesis.II	All Crohn's Disease	1.48	0.907	0.202	1.
POLYAMINSYN3.PWY..superpathway.of.polyamine.biosynthesis.II	All Ulcerative Colitis	1.6	0.89	0.205	1.
POLYAMSYN.PWY..superpathway.of.polyamine.biosynthesis.I	All Crohn's Disease	0.912	0.652	0.145	1.
POLYAMSYN.PWY..superpathway.of.polyamine.biosynthesis.I	All Ulcerative Colitis	1.11	0.635	0.121	1.
POLYISOPRENSYN.PWY..polyisoprenoid.biosynthesis..E..coli.	All Crohn's Disease	0.288	0.699	0.614	1.
POLYISOPRENSYN.PWY..polyisoprenoid.biosynthesis..E..coli.	All Ulcerative Colitis	0.493	0.681	0.559	1.
PPGPPMET.PWY..ppGpp.metabolism	All Crohn's Disease	-0.292	0.612	1.	1.
PPGPPMET.PWY..ppGpp.metabolism	All Ulcerative Colitis	-0.421	0.596	0.4	1.
PROPFERM.PWY..superpathway.of.L.alanine.fermentation..Stickland.reaction.	All Crohn's Disease	1.08	0.901	0.55	1.
PROPFERM.PWY..superpathway.of.L.alanine.fermentation..Stickland.reaction.	All Ulcerative Colitis	1.25	0.661	0.392	1.
PROTOCATECHUATE.ORTHO.CLEAVAGE.PWY protocatechuate.degradation.II..ortho.cleavage.pathway.	All Crohn's Disease	0.595	0.746	0.755	1.
PROTOCATECHUATE.ORTHO.CLEAVAGE.PWY protocatechuate.degradation.II..ortho.cleavage.pathway.	All Ulcerative Colitis	-0.472	0.612	0.777	1.
PWY.1042..glycolysis.IV	All Crohn's Disease	-0.0661	0.236	1.	1.
PWY.1042..glycolysis.IV	All Ulcerative Colitis	-0.208	0.23	0.209	1.
PWY.1269..CMP.3.deoxy.D.manno.octulosonate.biosynthesis	All Crohn's Disease	0.437	0.648	0.442	1.
PWY.1269..CMP.3.deoxy.D.manno.octulosonate.biosynthesis	All Ulcerative Colitis	0.505	0.631	0.516	1.
PWY.1861..formaldehyde.assimilation.II..assimilatory.RuMP.Cycle.	All Crohn's Disease	-0.457	0.601	0.525	1.
PWY.1861..formaldehyde.assimilation.II..assimilatory.RuMP.Cycle.	All Ulcerative Colitis	-2.96×10^{-03}	0.586	1.	1.
PWY.241..C4.photosynthetic.carbon.assimilation.cycle..NADP.ME.type	All Crohn's Disease	-0.634	0.459	0.23	1.
PWY.241..C4.photosynthetic.carbon.assimilation.cycle..NADP.ME.type	All Ulcerative Colitis	0.257	0.447	1.	1.
PWY.2941..L.lysine.biosynthesis.II	All Crohn's Disease	-0.526	0.434	0.302	1.
PWY.2941..L.lysine.biosynthesis.II	All Ulcerative Colitis	-0.531	0.422	0.154	1.

PWY.2942..L.lysine.biosynthesis.III	All Crohn's Disease	-0.27	0.252	1.	1.
PWY.2942..L.lysine.biosynthesis.III	All Ulcerative Colitis	-0.209	0.246	0.237	1.
PWY.3001..superpathway.of.L.isoleucine.biosynthesis.I	All Crohn's Disease	-0.522	0.274	0.111	1.
PWY.3001..superpathway.of.L.isoleucine.biosynthesis.I	All Ulcerative Colitis	-0.323	0.267	0.136	1.
PWY.3841..folate.transformations.II..plants.	All Crohn's Disease	-0.0899	0.243	1.	1.
PWY.3841..folate.transformations.II..plants.	All Ulcerative Colitis	-0.278	0.237	0.134	1.
PWY.4041...gamma..glutamyl.cycle	All Crohn's Disease	-0.696	0.424	0.151	1.
PWY.4041...gamma..glutamyl.cycle	All Ulcerative Colitis	0.0857	0.413	1.	1.
PWY.4984..urea.cycle	All Crohn's Disease	0.34	0.78	0.605	1.
PWY.4984..urea.cycle	All Ulcerative Colitis	-0.68	0.759	0.321	1.
PWY.5005..biotin.biosynthesis.II	All Crohn's Disease	0.022	0.565	1.	1.
PWY.5005..biotin.biosynthesis.II	All Ulcerative Colitis	-0.608	0.574	0.42	1.
PWY.5022..4.aminobutanoate.degradation.V	All Crohn's Disease	0.614	0.731	0.591	1.
PWY.5022..4.aminobutanoate.degradation.V	All Ulcerative Colitis	0.0861	0.731	0.591	1.
PWY.5030..L.histidine.degradation.III	All Crohn's Disease	0.0354	0.699	1.	1.
PWY.5030..L.histidine.degradation.III	All Ulcerative Colitis	-0.796	0.681	0.206	1.
PWY.5097..L.lysine.biosynthesis.VI	All Crohn's Disease	-0.169	0.277	1.	1.
PWY.5097..L.lysine.biosynthesis.VI	All Ulcerative Colitis	-0.262	0.27	0.205	1.
PWY.5100..pyruvate.fermentation.to.acetate.and.lactate.II	All Crohn's Disease	-0.494	0.321	0.198	1.
PWY.5100..pyruvate.fermentation.to.acetate.and.lactate.II	All Ulcerative Colitis	-0.271	0.313	0.261	1.
PWY.5103..L.isoleucine.biosynthesis.III	All Crohn's Disease	-0.338	0.293	0.367	1.
PWY.5103..L.isoleucine.biosynthesis.III	All Ulcerative Colitis	-0.36	0.285	0.127	1.
PWY.5104..L.isoleucine.biosynthesis.IV	All Crohn's Disease	0.36	1.56	0.298	1.
PWY.5104..L.isoleucine.biosynthesis.IV	All Ulcerative Colitis	2.72	1.6	0.245	1.
PWY.5121..superpathway.of.geranylgeranyl.diphosphate.biosynthesis.II..via.MEP.	All Crohn's Disease	1.06	0.558	0.054	1.
PWY.5121..superpathway.of.geranylgeranyl.diphosphate.biosynthesis.II..via.MEP.	All Ulcerative Colitis	0.659	0.543	0.304	1.
PWY.5130..2.oxobutanoate.degradation.I	All Ulcerative Colitis	0.802	0.706	0.431	1.
PWY.5136..fatty.acid..beta..oxidation.II..plant.peroxisome.	All Crohn's Disease	0.911	0.798	0.231	1.
PWY.5138..fatty.acid..beta..oxidation.IV..unsaturated..even.number.	All Crohn's Disease	0.897	2.87	0.675	1.
PWY.5138..fatty.acid..beta..oxidation.IV..unsaturated..even.number.	All Ulcerative Colitis	2.3	2.81	0.592	1.
PWY.5154..L.arginine.biosynthesis.III..via.N.acetyl.L.citrulline.	All Crohn's Disease	-0.0149	0.507	1.	1.
PWY.5154..L.arginine.biosynthesis.III..via.N.acetyl.L.citrulline.	All Ulcerative Colitis	0.0212	0.493	1.	1.
PWY.5188..tetrapyrrole.biosynthesis.I..from.glutamate.	All Crohn's Disease	0.246	0.41	0.451	1.
PWY.5188..tetrapyrrole.biosynthesis.I..from.glutamate.	All Ulcerative Colitis	0.0548	0.4	1.	1.
PWY.5189..tetrapyrrole.biosynthesis.II..from.glycine.	All Crohn's Disease	-0.382	1.07	0.669	1.
PWY.5189..tetrapyrrole.biosynthesis.II..from.glycine.	All Ulcerative Colitis	0.861	1.02	0.707	1.
PWY.5265..peptidoglycan.biosynthesis.II..staphylococci.	All Crohn's Disease	0.407	1.08	0.888	1.
PWY.5265..peptidoglycan.biosynthesis.II..staphylococci.	All Ulcerative Colitis	1.01	1.1	0.658	1.
PWY.5345..superpathway.of.L.methionine.biosynthesis..by.sulfhydrylation.	All Crohn's Disease	0.672	0.742	0.549	1.
PWY.5345..superpathway.of.L.methionine.biosynthesis..by.sulfhydrylation.	All Ulcerative Colitis	1.81	0.726	0.0509	1.
PWY.5347..superpathway.of.L.methionine.biosynthesis..transsulfuration.	All Crohn's Disease	1.1	1.05	0.459	1.
PWY.5347..superpathway.of.L.methionine.biosynthesis..transsulfuration.	All Ulcerative Colitis	2.03	1.04	0.143	1.
PWY.5367..petroselinate.biosynthesis	All Crohn's Disease	-0.664	0.782	0.703	1.
PWY.5367..petroselinate.biosynthesis	All Ulcerative Colitis	-0.85	0.772	0.417	1.
PWY.5384..sucrose.degradation.IV..sucrose.phosphorylase.	All Crohn's Disease	-0.804	0.462	0.125	1.
PWY.5384..sucrose.degradation.IV..sucrose.phosphorylase.	All Ulcerative Colitis	-0.0915	0.45	1.	1.
PWY.5464..superpathway.of.cytosolic.glycolysis..plants. pyruvate.dehydrogenase.and.TCA.cycle	All Crohn's Disease	-1.69	0.765	0.0989	1.
PWY.5464..superpathway.of.cytosolic.glycolysis..plants pyruvate.dehydrogenase.and.TCA.cycle	All Ulcerative Colitis	-0.0287	0.752	0.984	1.
PWY.5484..glycolysis.II..from.fructose.6.phosphate.	All Crohn's Disease	-0.699	0.341	0.0765	1.
PWY.5484..glycolysis.II..from.fructose.6.phosphate.	All Ulcerative Colitis	-0.0855	0.332	1.	1.
PWY.5494..pyruvate.fermentation.to.propanoate.II..acrylate.pathway.	All Crohn's Disease	1.1	0.94	0.563	1.
PWY.5494..pyruvate.fermentation.to.propanoate.II..acrylate.pathway.	All Ulcerative Colitis	1.25	0.69	0.414	1.
PWY.5497..purine.nucleobases.degradation.II..anaerobic.	All Crohn's Disease	-0.329	0.66	0.909	1.
PWY.5497..purine.nucleobases.degradation.II..anaerobic.	All Ulcerative Colitis	-0.416	0.655	0.696	1.
PWY.5505..L.glutamate.and.L.glutamine.biosynthesis	All Crohn's Disease	-0.108	1.05	0.999	1.
PWY.5505..L.glutamate.and.L.glutamine.biosynthesis	All Ulcerative Colitis	-0.789	1.02	0.639	1.
PWY.5531..3.8.divinyl.chlorophyllide.a.biosynthesis.II..anaerobic.	All Crohn's Disease	-0.0824	1.13	0.241	1.
PWY.5531..3.8.divinyl.chlorophyllide.a.biosynthesis.II..anaerobic.	All Ulcerative Colitis	4.6	1.88	0.225	1.
PWY.561..superpathway.of.glyoxylate.cycle.and.fatty.acid.degradation	All Crohn's Disease	0.0487	2.05	0.486	1.
PWY.561..superpathway.of.glyoxylate.cycle.and.fatty.acid.degradation	All Ulcerative Colitis	1.55	2.04	0.579	1.
PWY.5656..mannosylglycerate.biosynthesis.I	All Crohn's Disease	0.593	2.14	0.947	1.
PWY.5656..mannosylglycerate.biosynthesis.I	All Ulcerative Colitis	2.94	2.04	0.394	1.
PWY.5659..GDP.mannose.biosynthesis	All Crohn's Disease	-0.321	0.3	0.408	1.
PWY.5659..GDP.mannose.biosynthesis	All Ulcerative Colitis	0.0222	0.292	1.	1.
PWY.5667..CDP.diacylglycerol.biosynthesis.I	All Crohn's Disease	-0.0277	0.233	1.	1.
PWY.5667..CDP.diacylglycerol.biosynthesis.I	All Ulcerative Colitis	-0.287	0.227	0.11	1.
PWY.5675..nitrate.reduction.V..assimilatory.	All Crohn's Disease	-1.09	2.49	0.331	1.
PWY.5675..nitrate.reduction.V..assimilatory.	All Ulcerative Colitis	3.22	2.52	0.426	1.
PWY.5676..acetyl.CoA.fermentation.to.butanoate.II	All Crohn's Disease	-0.479	0.572	0.48	1.
PWY.5676..acetyl.CoA.fermentation.to.butanoate.II	All Ulcerative Colitis	-0.357	0.557	0.43	1.
PWY.5677..succinate.fermentation.to.butanoate	All Crohn's Disease	0.463	1.14	0.444	1.
PWY.5677..succinate.fermentation.to.butanoate	All Ulcerative Colitis	0.563	1.06	0.688	1.
PWY.5686..UMP.biosynthesis.I	All Crohn's Disease	-0.164	0.259	1.	1.
PWY.5686..UMP.biosynthesis.I	All Ulcerative Colitis	-0.327	0.252	0.112	1.
PWY.5690..TCA.cycle.II..plants.and.fungi.	All Crohn's Disease	-0.27	0.712	0.703	1.
PWY.5690..TCA.cycle.II..plants.and.fungi.	All Ulcerative Colitis	0.267	0.695	0.633	1.
PWY.5695..inosine.5..phosphate.degradation	All Crohn's Disease	-0.182	0.269	1.	1.
PWY.5695..inosine.5..phosphate.degradation	All Ulcerative Colitis	-0.123	0.262	1.	1.

PWY.5747..2.methylcitrate.cycle.II	All Crohn's Disease	2.4	3.1	0.718	1.
PWY.5747..2.methylcitrate.cycle.II	All Ulcerative Colitis	4.02	2.78	0.407	1.
PWY.5837..2.carboxy.1.4.naphthoquinol.biosynthesis	All Crohn's Disease	1.05	1.07	0.52	1.
PWY.5837..2.carboxy.1.4.naphthoquinol.biosynthesis	All Ulcerative Colitis	0.889	1.04	0.395	1.
PWY.5838..superpathway.of.menaquinol.8.biosynthesis.I	All Crohn's Disease	0.951	0.831	0.41	1.
PWY.5838..superpathway.of.menaquinol.8.biosynthesis.I	All Ulcerative Colitis	0.748	0.811	0.395	1.
PWY.5840..superpathway.of.menaquinol.7.biosynthesis	All Crohn's Disease	0.587	0.845	0.694	1.
PWY.5840..superpathway.of.menaquinol.7.biosynthesis	All Ulcerative Colitis	0.857	0.831	0.598	1.
PWY.5845..superpathway.of.menaquinol.9.biosynthesis	All Crohn's Disease	1.16	1.04	0.44	1.
PWY.5845..superpathway.of.menaquinol.9.biosynthesis	All Ulcerative Colitis	1.49	1.02	0.332	1.
PWY.5850..superpathway.of.menaquinol.6.biosynthesis	All Crohn's Disease	-0.663	1.52	0.492	1.
PWY.5850..superpathway.of.menaquinol.6.biosynthesis	All Ulcerative Colitis	0.62	1.52	0.715	1.
PWY.5860..superpathway.of.demethylmenaquinol.6.biosynthesis.I	All Crohn's Disease	-0.691	1.58	0.492	1.
PWY.5860..superpathway.of.demethylmenaquinol.6.biosynthesis.I	All Ulcerative Colitis	0.697	1.58	0.715	1.
PWY.5861..superpathway.of.demethylmenaquinol.8.biosynthesis.I	All Crohn's Disease	0.939	0.879	0.457	1.
PWY.5861..superpathway.of.demethylmenaquinol.8.biosynthesis.I	All Ulcerative Colitis	0.781	0.858	0.395	1.
PWY.5862..superpathway.of.demethylmenaquinol.9.biosynthesis	All Crohn's Disease	1.18	1.09	0.466	1.
PWY.5862..superpathway.of.demethylmenaquinol.9.biosynthesis	All Ulcerative Colitis	1.56	1.07	0.332	1.
PWY.5896..superpathway.of.menaquinol.10.biosynthesis	All Crohn's Disease	-0.663	1.52	0.492	1.
PWY.5896..superpathway.of.menaquinol.10.biosynthesis	All Ulcerative Colitis	0.62	1.52	0.715	1.
PWY.5897..superpathway.of.menaquinol.11.biosynthesis	All Crohn's Disease	0.858	0.868	0.505	1.
PWY.5897..superpathway.of.menaquinol.11.biosynthesis	All Ulcerative Colitis	0.688	0.847	0.395	1.
PWY.5898..superpathway.of.menaquinol.12.biosynthesis	All Crohn's Disease	0.858	0.868	0.505	1.
PWY.5898..superpathway.of.menaquinol.12.biosynthesis	All Ulcerative Colitis	0.688	0.847	0.395	1.
PWY.5899..superpathway.of.menaquinol.13.biosynthesis	All Crohn's Disease	0.858	0.868	0.505	1.
PWY.5899..superpathway.of.menaquinol.13.biosynthesis	All Ulcerative Colitis	0.688	0.847	0.395	1.
PWY.5910..superpathway.of.geranylgeranyldiphosphate.biosynthesis.I..via.mevalonate.	All Crohn's Disease	1.02	0.936	0.458	1.
PWY.5910..superpathway.of.geranylgeranyldiphosphate.biosynthesis.I..via.mevalonate.	All Ulcerative Colitis	2.33	0.923	0.0559	1.
PWY.5913..partial.TCA.cycle..obligate.autotrophs.	All Crohn's Disease	-0.572	0.479	0.304	1.
PWY.5913..partial.TCA.cycle..obligate.autotrophs.	All Ulcerative Colitis	-0.042	0.467	1.	1.
PWY.5918..superpathway.of.heme.b.biosynthesis.from.glutamate	All Crohn's Disease	0.339	1.05	0.855	1.
PWY.5918..superpathway.of.heme.b.biosynthesis.from.glutamate	All Ulcerative Colitis	0.833	1.02	0.724	1.
PWY.5920..superpathway.of.heme.b.biosynthesis.from.glycine	All Crohn's Disease	0.658	2.29	0.943	1.
PWY.5920..superpathway.of.heme.b.biosynthesis.from.glycine	All Ulcerative Colitis	2.52	2.13	0.506	1.
PWY.5941..glycogen.degradation.II	All Crohn's Disease	-0.37	0.338	0.382	1.
PWY.5941..glycogen.degradation.II	All Ulcerative Colitis	-0.343	0.329	0.201	1.
PWY.5971..palmitate.biosynthesis..type.II.fatty.acid.synthase.	All Crohn's Disease	0.0922	0.756	0.999	1.
PWY.5971..palmitate.biosynthesis..type.II.fatty.acid.synthase.	All Ulcerative Colitis	0.783	0.746	0.593	1.
PWY.5972..stearate.biosynthesis.I..animals.	All Crohn's Disease	-0.506	2.12	0.958	1.
PWY.5972..stearate.biosynthesis.I..animals.	All Ulcerative Colitis	3.06	1.97	0.468	1.
PWY.5973..cis.vaccenate.biosynthesis	All Crohn's Disease	0.0718	0.445	1.	1.
PWY.5973..cis.vaccenate.biosynthesis	All Ulcerative Colitis	-0.0177	0.434	1.	1.
PWY.5981..CDP.diacylglycerol.biosynthesis.III	All Crohn's Disease	-1.06	1.31	0.455	1.
PWY.5981..CDP.diacylglycerol.biosynthesis.III	All Ulcerative Colitis	-0.236	1.27	0.801	1.
PWY.5989..stearate.biosynthesis.II..bacteria.and.plants.	All Crohn's Disease	0.0858	0.423	1.	1.
PWY.5989..stearate.biosynthesis.II..bacteria.and.plants.	All Ulcerative Colitis	0.699	0.412	0.151	1.
PWY.5994..palmitate.biosynthesis..type.I.fatty.acid.synthase.	All Crohn's Disease	0.223	0.893	0.519	1.
PWY.5994..palmitate.biosynthesis..type.I.fatty.acid.synthase.	All Ulcerative Colitis	2.27	0.966	0.0856	1.
PWY.6121..5.aminoimidazole.ribonucleotide.biosynthesis.I	All Crohn's Disease	-0.119	0.231	1.	1.
PWY.6121..5.aminoimidazole.ribonucleotide.biosynthesis.I	All Ulcerative Colitis	-0.268	0.225	0.126	1.
PWY.6122..5.aminoimidazole.ribonucleotide.biosynthesis.II	All Crohn's Disease	-0.18	0.247	1.	1.
PWY.6122..5.aminoimidazole.ribonucleotide.biosynthesis.II	All Ulcerative Colitis	-0.285	0.24	0.133	1.
PWY.6123..inosine.5..phosphate.biosynthesis.I	All Crohn's Disease	-0.314	0.237	1.	1.
PWY.6123..inosine.5..phosphate.biosynthesis.I	All Ulcerative Colitis	-0.273	0.231	0.13	1.
PWY.6124..inosine.5..phosphate.biosynthesis.II	All Crohn's Disease	-0.336	0.242	0.281	1.
PWY.6124..inosine.5..phosphate.biosynthesis.II	All Ulcerative Colitis	-0.269	0.236	0.141	1.
PWY.6125..superpathway.of.guanosine.nucleotides.de.novo.biosynthesis.II	All Crohn's Disease	-0.0673	0.5	1.	1.
PWY.6125..superpathway.of.guanosine.nucleotides.de.novo.biosynthesis.II	All Ulcerative Colitis	0.0288	0.487	1.	1.
PWY.6126..superpathway.of.adenosine.nucleotides.de.novo.biosynthesis.II	All Crohn's Disease	-0.0973	0.461	1.	1.
PWY.6126..superpathway.of.adenosine.nucleotides.de.novo.biosynthesis.II	All Ulcerative Colitis	-0.0105	0.449	1.	1.
PWY.6143..CMP.pseudaminate.biosynthesis	All Crohn's Disease	0.234	1.68	0.945	1.
PWY.6147..6.hydroxymethyl.dihydropterin.diphosphate.biosynthesis.I	All Crohn's Disease	-0.454	0.554	0.494	1.
PWY.6147..6.hydroxymethyl.dihydropterin.diphosphate.biosynthesis.I	All Ulcerative Colitis	-0.258	0.539	0.526	1.
PWY.6151..S.adenosyl.L.methionine.salvage.I	All Crohn's Disease	-0.321	0.298	0.404	1.
PWY.6151..S.adenosyl.L.methionine.salvage.I	All Ulcerative Colitis	-0.419	0.29	0.0918	1.
PWY.6163..chorismate.biosynthesis.from.3.dehydroquinate	All Crohn's Disease	-0.179	0.264	1.	1.
PWY.6163..chorismate.biosynthesis.from.3.dehydroquinate	All Ulcerative Colitis	-0.413	0.257	0.0626	1.
PWY.6168..flavin.biosynthesis.III..fungi.	All Crohn's Disease	-0.545	0.936	0.773	1.
PWY.6168..flavin.biosynthesis.III..fungi.	All Ulcerative Colitis	0.0417	0.909	0.747	1.
PWY.621..sucrose.degradation.III..sucrose.invertase.	All Crohn's Disease	-0.673	0.416	0.294	1.
PWY.621..sucrose.degradation.III..sucrose.invertase.	All Ulcerative Colitis	-0.0932	0.407	0.991	1.
PWY.622..starch.biosynthesis	All Crohn's Disease	-2.01	0.762	0.0409	1.
PWY.622..starch.biosynthesis	All Ulcerative Colitis	0.326	0.764	0.468	1.
PWY.6270..isoprene.biosynthesis.I	All Crohn's Disease	-0.213	0.332	1.	1.
PWY.6270..isoprene.biosynthesis.I	All Ulcerative Colitis	-0.525	0.324	0.0691	1.
PWY.6277..superpathway.of.5.aminoimidazole.ribonucleotide.biosynthesis	All Crohn's Disease	-0.18	0.247	1.	1.
PWY.6277..superpathway.of.5.aminoimidazole.ribonucleotide.biosynthesis	All Ulcerative Colitis	-0.285	0.24	0.133	1.
PWY.6282..palmitoleate.biosynthesis.I..from..5Z..dodec.5.enoate.	All Crohn's Disease	0.108	0.716	1.	1.
PWY.6282..palmitoleate.biosynthesis.I..from..5Z..dodec.5.enoate.	All Ulcerative Colitis	1.04	0.697	0.187	1.
PWY.6284..superpathway.of.unsaturated.fatty.acids.biosynthesis..E..coli.	All Crohn's Disease	-0.257	0.729	0.999	1.

PWY.6284..superpathway.of.unsaturated.fatty.acids.biosynthesis..E..coli.	All Ulcerative Colitis	0.0277	0.72	0.905	1.
PWY.6285..superpathway.of.fatty.acids.biosynthesis..E..coli.	All Crohn's Disease	1.39	1.36	0.522	1.
PWY.6285..superpathway.of.fatty.acids.biosynthesis..E..coli.	All Ulcerative Colitis	1.65	1.29	0.341	1.
PWY.6292..superpathway.of.L.cysteine.biosynthesis..mammalian.	All Crohn's Disease	-0.163	0.541	1.	1.
PWY.6292..superpathway.of.L.cysteine.biosynthesis..mammalian.	All Ulcerative Colitis	-0.629	0.527	0.185	1.
PWY.6293..superpathway.of.L.cysteine.biosynthesis..fungi.	All Crohn's Disease	-1.11	1.35	0.305	1.
PWY.6293..superpathway.of.L.cysteine.biosynthesis..fungi.	All Ulcerative Colitis	-0.844	1.16	0.677	1.
PWY.6305..superpathway.of.putrescine.biosynthesis	All Crohn's Disease	0.558	0.73	0.399	1.
PWY.6305..superpathway.of.putrescine.biosynthesis	All Ulcerative Colitis	0.467	0.711	0.6	1.
PWY.6317..D.galactose.degradation.I..Leloir.pathway.	All Crohn's Disease	-0.291	0.335	1.	1.
PWY.6317..D.galactose.degradation.I..Leloir.pathway.	All Ulcerative Colitis	-0.327	0.326	0.213	1.
PWY.6353..purine.nucleotides.degradation.II..aerobic.	All Crohn's Disease	-0.116	0.445	1.	1.
PWY.6353..purine.nucleotides.degradation.II..aerobic.	All Ulcerative Colitis	-0.114	0.433	1.	1.
PWY.6385..peptidoglycan.biosynthesis.III..mycobacteria.	All Crohn's Disease	-0.133	0.229	1.	1.
PWY.6385..peptidoglycan.biosynthesis.III..mycobacteria.	All Ulcerative Colitis	-0.263	0.223	0.127	1.
PWY.6386..UDP.N.acetylmuramoyl.pentapeptide.biosynthesis.II..lysine.containing.	All Crohn's Disease	-0.169	0.242	1.	1.
PWY.6386..UDP.N.acetylmuramoyl.pentapeptide.biosynthesis.II..lysine.containing.	All Ulcerative Colitis	-0.296	0.235	0.115	1.
PWY.6387..UDP.N.acetylmuramoyl.pentapeptide.biosynthesis.I meso.diaminopimelate.containing.	All Crohn's Disease	-0.169	0.242	1.	1.
PWY.6387..UDP.N.acetylmuramoyl.pentapeptide.biosynthesis.I meso.diaminopimelate.containing.	All Ulcerative Colitis	-0.292	0.235	0.118	1.
PWY.6435..4.hydroxybenzoate.biosynthesis.III..plants.	All Crohn's Disease	-0.419	0.899	0.494	1.
PWY.6435..4.hydroxybenzoate.biosynthesis.III..plants.	All Ulcerative Colitis	1.09	0.981	0.178	1.
PWY.6470..peptidoglycan.biosynthesis.V...beta..lactam.resistance.	All Crohn's Disease	-0.329	0.916	0.951	1.
PWY.6470..peptidoglycan.biosynthesis.V...beta..lactam.resistance.	All Ulcerative Colitis	-1.07	0.896	0.372	1.
PWY.6478..GDP.D.glycero..alpha..D.manno.heptose.biosynthesis	All Crohn's Disease	-0.0145	1.43	0.534	1.
PWY.6478..GDP.D.glycero..alpha..D.manno.heptose.biosynthesis	All Ulcerative Colitis	-2.77	1.36	0.236	1.
PWY.6507..4.deoxy.L.threo.hex.4.enopyranuronate.degradation	All Crohn's Disease	0.824	0.574	0.132	1.
PWY.6507..4.deoxy.L.threo.hex.4.enopyranuronate.degradation	All Ulcerative Colitis	0.392	0.559	0.593	1.
PWY.6518..bile.acids.epimerization	All Crohn's Disease	-0.863	2.05	0.704	1.
PWY.6518..bile.acids.epimerization	All Ulcerative Colitis	-0.125	2.22	1.	1.
PWY.6519..8.amino.7.oxononanoate.biosynthesis.I	All Crohn's Disease	0.171	0.692	1.	1.
PWY.6519..8.amino.7.oxononanoate.biosynthesis.I	All Ulcerative Colitis	0.601	0.674	0.454	1.
PWY.6527..stachyose.degradation	All Crohn's Disease	-0.335	0.37	0.478	1.
PWY.6527..stachyose.degradation	All Ulcerative Colitis	-0.214	0.36	0.407	1.
PWY.6531..mannitol.cycle	All Crohn's Disease	-0.269	1.35	0.304	1.
PWY.6531..mannitol.cycle	All Ulcerative Colitis	0.409	1.36	0.38	1.
PWY.6545..pyrimidine.deoxyribonucleotides.de.novo.biosynthesis.III	All Crohn's Disease	-0.0809	1.58	0.486	1.
PWY.6545..pyrimidine.deoxyribonucleotides.de.novo.biosynthesis.III	All Ulcerative Colitis	1.06	1.56	0.524	1.
PWY.6549..L.glutamine.biosynthesis.III	All Crohn's Disease	-0.312	0.439	1.	1.
PWY.6549..L.glutamine.biosynthesis.III	All Ulcerative Colitis	-0.154	0.427	1.	1.
PWY.6572..chondroitin.sulfate.degradation.I..bacterial.	All Crohn's Disease	-1.93	1.78	0.561	1.
PWY.6572..chondroitin.sulfate.degradation.I..bacterial.	All Ulcerative Colitis	-3.14	1.91	0.264	1.
PWY.6588..pyruvate.fermentation.to.acetone	All Crohn's Disease	0.994	1.16	0.488	1.
PWY.6588..pyruvate.fermentation.to.acetone	All Ulcerative Colitis	1.7	1.15	0.321	1.
PWY.6590..superpathway.of.Clostridium.acetobutylicum.acidogenic.fermentation	All Crohn's Disease	-0.0569	0.715	0.874	1.
PWY.6590..superpathway.of.Clostridium.acetobutylicum.acidogenic.fermentation	All Ulcerative Colitis	-0.243	0.703	0.76	1.
PWY.6595..superpathway.of.guanosine.nucleotides.degradation..plants.	All Crohn's Disease	0.0472	0.957	1.	1.
PWY.6595..superpathway.of.guanosine.nucleotides.degradation..plants.	All Ulcerative Colitis	0.866	0.932	0.412	1.
PWY.6606..guanosine.nucleotides.degradation.II	All Crohn's Disease	-0.105	0.482	1.	1.
PWY.6606..guanosine.nucleotides.degradation.II	All Ulcerative Colitis	-0.112	0.469	1.	1.
PWY.6607..guanosine.nucleotides.degradation.I	All Crohn's Disease	0.0521	0.953	1.	1.
PWY.6607..guanosine.nucleotides.degradation.I	All Ulcerative Colitis	0.906	0.927	0.387	1.
PWY.6608..guanosine.nucleotides.degradation.III	All Crohn's Disease	-0.139	0.354	1.	1.
PWY.6608..guanosine.nucleotides.degradation.III	All Ulcerative Colitis	0.0941	0.344	1.	1.
PWY.6609..adenine.and.adenosine.salvage.III	All Crohn's Disease	0.0503	0.232	1.	1.
PWY.6609..adenine.and.adenosine.salvage.III	All Ulcerative Colitis	-0.153	0.226	1.	1.
PWY.6612..superpathway.of.tetrahydrofolate.biosynthesis	All Crohn's Disease	0.164	0.487	0.999	1.
PWY.6612..superpathway.of.tetrahydrofolate.biosynthesis	All Ulcerative Colitis	4.81×10^{-03}	0.476	0.991	1.
PWY.6628..superpathway.of.L.phenylalanine.biosynthesis	All Crohn's Disease	-0.736	0.357	0.0732	1.
PWY.6628..superpathway.of.L.phenylalanine.biosynthesis	All Ulcerative Colitis	-0.402	0.347	0.17	1.
PWY.6630..superpathway.of.L.tyrosine.biosynthesis	All Crohn's Disease	-0.497	0.381	0.272	1.
PWY.6630..superpathway.of.L.tyrosine.biosynthesis	All Ulcerative Colitis	-0.283	0.371	0.325	1.
PWY.6690..cinnamate.and.3.hydroxycinnamate.degradation.to.2.hydroxypentadienoate	All Crohn's Disease	0.838	3.35	0.96	1.
PWY.6690..cinnamate.and.3.hydroxycinnamate.degradation.to.2.hydroxypentadienoate	All Ulcerative Colitis	1.54	3.23	0.896	1.
PWY.6700..queuosine.biosynthesis.I..de.novo.	All Crohn's Disease	0.0899	0.253	1.	1.
PWY.6700..queuosine.biosynthesis.I..de.novo.	All Ulcerative Colitis	-0.282	0.247	0.145	1.
PWY.6703..preQ0.biosynthesis	All Crohn's Disease	-0.111	0.274	1.	1.
PWY.6703..preQ0.biosynthesis	All Ulcerative Colitis	-0.246	0.267	0.221	1.
PWY.6731..starch.degradation.III	All Crohn's Disease	-0.471	0.622	0.525	1.
PWY.6731..starch.degradation.III	All Ulcerative Colitis	-0.763	0.606	0.172	1.
PWY.6749..CMP.legionaminate.biosynthesis.I	All Crohn's Disease	-3.81	2.04	0.189	1.
PWY.6749..CMP.legionaminate.biosynthesis.I	All Ulcerative Colitis	-4.02	2.01	0.138	1.
PWY.6803..phosphatidylcholine.acyl.editing	All Crohn's Disease	0.459	2.33	0.334	1.
PWY.6803..phosphatidylcholine.acyl.editing	All Ulcerative Colitis	2.06	2.33	0.478	1.
PWY.6807..xyloglucan.degradation.II..exoglucanase.	All Crohn's Disease	-0.988	1.42	0.782	1.
PWY.6807..xyloglucan.degradation.II..exoglucanase.	All Ulcerative Colitis	-2.68	1.48	0.181	1.
PWY.6823..molybdopterin.biosynthesis	All Crohn's Disease	-0.136	0.363	1.	1.
PWY.6823..molybdopterin.biosynthesis	All Ulcerative Colitis	-0.161	0.354	0.485	1.
PWY.6859..all.trans.farnesol.biosynthesis	All Crohn's Disease	1.43	0.914	0.113	1.

PWY.6859..all.trans.farnesol.biosynthesis	All Ulcerative Colitis	1.6	0.89	0.103	1.
PWY.6876..isopropanol.biosynthesis..engineered.	All Crohn's Disease	1.39	1.79	0.275	1.
PWY.6876..isopropanol.biosynthesis..engineered.	All Ulcerative Colitis	1.68	1.26	0.504	1.
PWY.6895..superpathway.of.thiamine.diphosphate.biosynthesis.II	All Crohn's Disease	0.22	0.546	0.601	1.
PWY.6895..superpathway.of.thiamine.diphosphate.biosynthesis.II	All Ulcerative Colitis	-0.621	0.531	0.193	1.
PWY.6897..thiamine.diphosphate.salvage.II	All Crohn's Disease	-0.163	0.298	1.	1.
PWY.6897..thiamine.diphosphate.salvage.II	All Ulcerative Colitis	-0.302	0.29	0.189	1.
PWY.6901..superpathway.of.glucose.and.xylose.degradation	All Crohn's Disease	-0.378	0.369	0.411	1.
PWY.6901..superpathway.of.glucose.and.xylose.degradation	All Ulcerative Colitis	9.89×10^{-03}	0.36	1.	1.
PWY.6902..chitin.degradation.II..Vibrio.	All Crohn's Disease	-0.65	0.7	0.658	1.
PWY.6906..chitin.derivatives.degradation	All Crohn's Disease	-0.511	0.922	0.786	1.
PWY.6906..chitin.derivatives.degradation	All Ulcerative Colitis	-0.443	0.91	0.811	1.
PWY.6920..6.gingerol.analog.biosynthesis..engineered.	All Crohn's Disease	-1.18	0.965	0.324	1.
PWY.6920..6.gingerol.analog.biosynthesis..engineered.	All Ulcerative Colitis	-0.766	1.08	0.0962	1.
PWY.6936..seleno.amino.acid.biosynthesis..plants.	All Crohn's Disease	-0.57	0.3	0.107	1.
PWY.6936..seleno.amino.acid.biosynthesis..plants.	All Ulcerative Colitis	-0.349	0.292	0.146	1.
PWY.6953..dTDP.3.acetamido..alpha..D.fucose.biosynthesis	All Crohn's Disease	-2.44	0.709	0.152	1.
PWY.6953..dTDP.3.acetamido..alpha..D.fucose.biosynthesis	All Ulcerative Colitis	-2.19	0.764	0.183	1.
PWY.6961..L.ascorbate.degradation.II..bacterial..aerobic.	All Crohn's Disease	1.52	1.51	0.527	1.
PWY.6961..L.ascorbate.degradation.II..bacterial..aerobic.	All Ulcerative Colitis	3.49	1.46	0.0727	1.
PWY.6969..TCA.cycle.V..2.oxoglutarate.synthase.	All Crohn's Disease	-0.57	0.795	0.534	1.
PWY.6969..TCA.cycle.V..2.oxoglutarate.synthase.	All Ulcerative Colitis	-0.237	0.774	0.677	1.
PWY.6992..1.5.anhydrofructose.degradation	All Crohn's Disease	-1.86	1.69	0.545	1.
PWY.6992..1.5.anhydrofructose.degradation	All Ulcerative Colitis	-1.33	1.63	0.657	1.
PWY.7013...S..propane.1.2.diol.degradation	All Crohn's Disease	0.0481	0.728	1.	1.
PWY.7013...S..propane.1.2.diol.degradation	All Ulcerative Colitis	0.847	0.709	0.295	1.
PWY.702..L.methionine.biosynthesis.II	All Ulcerative Colitis	-0.578	0.354	0.0708	1.
PWY.7094..fatty.acid.salvage	All Crohn's Disease	2.27	2.94	0.712	1.
PWY.7094..fatty.acid.salvage	All Ulcerative Colitis	4.24	2.81	0.358	1.
PWY.7111..pyruvate.fermentation.to.isobutanol..engineered.	All Crohn's Disease	-0.398	0.276	0.244	1.
PWY.7111..pyruvate.fermentation.to.isobutanol..engineered.	All Ulcerative Colitis	-0.244	0.268	0.226	1.
PWY.7115..C4.photosynthetic.carbon.assimilation.cycle..NAD.ME.type	All Crohn's Disease	-0.602	0.648	0.418	1.
PWY.7115..C4.photosynthetic.carbon.assimilation.cycle..NAD.ME.type	All Ulcerative Colitis	0.19	0.631	1.	1.
PWY.7117..C4.photosynthetic.carbon.assimilation.cycle..PEPCK.type	All Crohn's Disease	-0.891	0.518	0.125	1.
PWY.7117..C4.photosynthetic.carbon.assimilation.cycle..PEPCK.type	All Ulcerative Colitis	0.204	0.505	1.	1.
PWY.7118..chitin.deacetylation	All Crohn's Disease	-0.8	2.28	0.175	1.
PWY.7118..chitin.deacetylation	All Ulcerative Colitis	1.16	2.29	0.435	1.
PWY.7184..pyrimidine.deoxyribonucleotides.de.novo.biosynthesis.I	All Crohn's Disease	-0.0639	1.66	0.486	1.
PWY.7184..pyrimidine.deoxyribonucleotides.de.novo.biosynthesis.I	All Ulcerative Colitis	1.15	1.64	0.524	1.
PWY.7196..superpathway.of.pyrimidine.ribonucleosides.salvage	All Crohn's Disease	-2.53	1.14	0.108	1.
PWY.7196..superpathway.of.pyrimidine.ribonucleosides.salvage	All Ulcerative Colitis	-0.611	1.22	0.469	1.
PWY.7197..pyrimidine.deoxyribonucleotide.phosphorylation	All Crohn's Disease	-0.126	0.493	1.	1.
PWY.7197..pyrimidine.deoxyribonucleotide.phosphorylation	All Ulcerative Colitis	0.0648	0.48	1.	1.
PWY.7198..pyrimidine.deoxyribonucleotides.de.novo.biosynthesis.IV	All Crohn's Disease	-0.123	0.512	1.	1.
PWY.7198..pyrimidine.deoxyribonucleotides.de.novo.biosynthesis.IV	All Ulcerative Colitis	0.348	0.499	0.608	1.
PWY.7199..pyrimidine.deoxyribonucleosides.salvage	All Crohn's Disease	0.108	0.244	1.	1.
PWY.7199..pyrimidine.deoxyribonucleosides.salvage	All Ulcerative Colitis	-0.225	0.238	0.198	1.
PWY.7204..pyridoxal.5..phosphate.salvage.II..plants.	All Crohn's Disease	3.38	1.91	0.193	1.
PWY.7208..superpathway.of.pyrimidine.nucleobases.salvage	All Crohn's Disease	0.0632	0.419	1.	1.
PWY.7208..superpathway.of.pyrimidine.nucleobases.salvage	All Ulcerative Colitis	-0.0422	0.408	1.	1.
PWY.7209..superpathway.of.pyrimidine.ribonucleosides.degradation	All Crohn's Disease	-0.857	0.845	0.597	1.
PWY.7209..superpathway.of.pyrimidine.ribonucleosides.degradation	All Ulcerative Colitis	-0.867	0.792	0.43	1.
PWY.7210..pyrimidine.deoxyribonucleotides.biosynthesis.from.CTP	All Crohn's Disease	-0.381	1.59	0.83	1.
PWY.7210..pyrimidine.deoxyribonucleotides.biosynthesis.from.CTP	All Ulcerative Colitis	0.887	1.57	0.394	1.
PWY.7211..superpathway.of.pyrimidine.deoxyribonucleotides.de.novo.biosynthesis	All Crohn's Disease	-0.443	1.38	0.487	1.
PWY.7211..superpathway.of.pyrimidine.deoxyribonucleotides.de.novo.biosynthesis	All Ulcerative Colitis	0.439	1.38	0.548	1.
PWY.7220..adenosine.deoxyribonucleotides.de.novo.biosynthesis.II	All Crohn's Disease	-0.0201	0.55	1.	1.
PWY.7220..adenosine.deoxyribonucleotides.de.novo.biosynthesis.II	All Ulcerative Colitis	0.0921	0.536	1.	1.
PWY.7221..guanosine.ribonucleotides.de.novo.biosynthesis	All Crohn's Disease	-0.196	0.245	1.	1.
PWY.7221..guanosine.ribonucleotides.de.novo.biosynthesis	All Ulcerative Colitis	-0.319	0.238	0.0988	1.
PWY.7222..guanosine.deoxyribonucleotides.de.novo.biosynthesis.II	All Crohn's Disease	-0.0201	0.55	1.	1.
PWY.7222..guanosine.deoxyribonucleotides.de.novo.biosynthesis.II	All Ulcerative Colitis	0.0921	0.536	1.	1.
PWY.7228..superpathway.of.guanosine.nucleotides.de.novo.biosynthesis.I	All Crohn's Disease	-0.0859	0.475	1.	1.
PWY.7228..superpathway.of.guanosine.nucleotides.de.novo.biosynthesis.I	All Ulcerative Colitis	0.0118	0.463	1.	1.
PWY.7229..superpathway.of.adenosine.nucleotides.de.novo.biosynthesis.I	All Crohn's Disease	-0.131	0.405	1.	1.
PWY.7229..superpathway.of.adenosine.nucleotides.de.novo.biosynthesis.I	All Ulcerative Colitis	-0.0545	0.394	1.	1.
PWY.7234..inosine.5..phosphate.biosynthesis.III	All Crohn's Disease	-0.535	0.536	0.392	1.
PWY.7234..inosine.5..phosphate.biosynthesis.III	All Ulcerative Colitis	0.114	0.522	1.	1.
PWY.7237..myo...chiro..and.scyllo.inositol.degradation	All Crohn's Disease	-0.489	0.358	0.44	1.
PWY.7237..myo...chiro..and.scyllo.inositol.degradation	All Ulcerative Colitis	-0.552	0.35	0.153	1.
PWY.7238..sucrose.biosynthesis.II	All Crohn's Disease	-0.323	0.349	0.474	1.
PWY.7238..sucrose.biosynthesis.II	All Ulcerative Colitis	-0.41	0.34	0.154	1.
PWY.724..superpathway.of.L.lysine..L.threonine.and.L.methionine.biosynthesis.II	All Crohn's Disease	-0.249	0.247	1.	1.
PWY.724..superpathway.of.L.lysine..L.threonine.and.L.methionine.biosynthesis.II	All Ulcerative Colitis	-0.282	0.241	0.136	1.
PWY.7242..D.fructuronate.degradation	All Crohn's Disease	0.418	0.539	0.375	1.
PWY.7242..D.fructuronate.degradation	All Ulcerative Colitis	0.0461	0.525	1.	1.
PWY.7282..4.amino.2.methyl.5.diphosphomethylpyrimidine.biosynthesis.II	All Crohn's Disease	0.3	0.427	0.396	1.
PWY.7282..4.amino.2.methyl.5.diphosphomethylpyrimidine.biosynthesis.II	All Ulcerative Colitis	-0.0491	0.416	1.	1.
PWY.7312..dTDP..beta..D.fucofuranose.biosynthesis	All Crohn's Disease	1.63	1.7	0.584	1.
PWY.7312..dTDP..beta..D.fucofuranose.biosynthesis	All Ulcerative Colitis	-1.51	2.08	0.722	1.

PWY.7315..dTDP.N.acetylthomosamine.biosynthesis	All Crohn's Disease	-0.341	1.25	0.829	1.
PWY.7315..dTDP.N.acetylthomosamine.biosynthesis	All Ulcerative Colitis	-0.254	1.22	0.78	1.
PWY.7316..dTDP.N.acetylviosamine.biosynthesis	All Crohn's Disease	0.23	2.72	0.459	1.
PWY.7316..dTDP.N.acetylviosamine.biosynthesis	All Ulcerative Colitis	-3.15	3.68	0.663	1.
PWY.7323..superpathway.of.GDP.mannose.derived O.antigen.building.blocks.biosynthesis	All Crohn's Disease	0.222	0.48	0.55	1.
PWY.7323..superpathway.of.GDP.mannose.derived O.antigen.building.blocks.biosynthesis	All Ulcerative Colitis	0.613	0.467	0.273	1.
PWY.7328..superpathway.of.UDP.glucose.derived O.antigen.building.blocks.biosynthesis	All Crohn's Disease	0.0137	0.355	1.	1.
PWY.7328..superpathway.of.UDP.glucose.derived O.antigen.building.blocks.biosynthesis	All Ulcerative Colitis	0.209	0.346	1.	1.
PWY.7340..9.cis..11.trans.octadecadienoyl.CoA.degradation isomerase.dependent..yeast.	All Crohn's Disease	0.594	2.48	0.881	1.
PWY.7340..9.cis..11.trans.octadecadienoyl.CoA.degradation isomerase.dependent..yeast.	All Ulcerative Colitis	2.49	2.43	0.592	1.
PWY.7345..superpathway.of.anaerobic.sucrose.degradation	All Crohn's Disease	0.416	0.392	0.406	1.
PWY.7345..superpathway.of.anaerobic.sucrose.degradation	All Ulcerative Colitis	1.04	0.387	0.0426	1.
PWY.7356..thiamine.diphosphate.salvage.IV..yeast.	All Crohn's Disease	-0.758	0.732	0.585	1.
PWY.7356..thiamine.diphosphate.salvage.IV..yeast.	All Ulcerative Colitis	-0.0605	0.715	0.991	1.
PWY.7357..thiamine.phosphate.formation.from.pyrithiamine.and.oxythiamine..yeast.	All Crohn's Disease	-0.195	0.297	1.	1.
PWY.7357..thiamine.phosphate.formation.from.pyrithiamine.and.oxythiamine..yeast.	All Ulcerative Colitis	-0.292	0.289	0.199	1.
PWY.7371..1.4.dihydroxy.6.naphthoate.biosynthesis.II	All Crohn's Disease	1.91	1.02	0.232	1.
PWY.7371..1.4.dihydroxy.6.naphthoate.biosynthesis.II	All Ulcerative Colitis	1.79	2.28	0.0627	1.
PWY.7383..anaerobic.energy.metabolism..invertebrates..cytosol.	All Crohn's Disease	-0.897	0.588	0.316	1.
PWY.7385..1.3.propanediol.biosynthesis..engineered.	All Crohn's Disease	4.12	1.5	0.204	1.
PWY.7385..1.3.propanediol.biosynthesis..engineered.	All Ulcerative Colitis	4.83	1.13	0.103	1.
PWY.7392..taxadiene.biosynthesis..engineered.	All Crohn's Disease	1.31	0.844	0.115	1.
PWY.7392..taxadiene.biosynthesis..engineered.	All Ulcerative Colitis	1.25	0.822	0.171	1.
PWY.7400..L.arginine.biosynthesis.IV..archaebacteria.	All Crohn's Disease	-0.19	0.244	1.	1.
PWY.7400..L.arginine.biosynthesis.IV..archaebacteria.	All Ulcerative Colitis	-0.289	0.238	0.124	1.
PWY.7434..terminal.O.glycans.residues.modification..via.type.2.precursor.disaccharide.	All Crohn's Disease	0.424	0.926	0.31	1.
PWY.7434..terminal.O.glycans.residues.modification..via.type.2.precursor.disaccharide.	All Ulcerative Colitis	0.569	0.884	0.34	1.
PWY.7456...beta...1.4..mannan.degradation	All Crohn's Disease	-0.745	0.638	0.299	1.
PWY.7456...beta...1.4..mannan.degradation	All Ulcerative Colitis	-0.74	0.621	0.194	1.
PWY.7560..methylerythritol.phosphate.pathway.II	All Crohn's Disease	-0.243	0.344	1.	1.
PWY.7560..methylerythritol.phosphate.pathway.II	All Ulcerative Colitis	-0.561	0.335	0.0634	1.
PWY.7616..methanol.oxidation.to.carbon.dioxide	All Crohn's Disease	0.271	0.0716	0.247	1.
PWY.7663..gondoate.biosynthesis..anaerobic.	All Crohn's Disease	0.109	0.487	1.	1.
PWY.7663..gondoate.biosynthesis..anaerobic.	All Ulcerative Colitis	0.404	0.474	0.513	1.
PWY.7664..oleate.biosynthesis.IV..anaerobic.	All Crohn's Disease	0.13	0.774	1.	1.
PWY.7664..oleate.biosynthesis.IV..anaerobic.	All Ulcerative Colitis	1.06	0.754	0.209	1.
PWY.7688..dTDP..alpha..D.ravidosamine.and dTDP.4.acetyl..alpha..D.ravidosamine.biosynthesis	All Crohn's Disease	-2.14	2.36	0.652	1.
PWY.7688..dTDP..alpha..D.ravidosamine.and dTDP.4.acetyl..alpha..D.ravidosamine.biosynthesis	All Ulcerative Colitis	-2.57	2.35	0.504	1.
PWY.7754..bile.acid.7.alpha..dehydroxylation	All Crohn's Disease	0.218	0.868	0.472	1.
PWY.7754..bile.acid.7.alpha..dehydroxylation	All Ulcerative Colitis	1.29	0.971	0.486	1.
PWY.7761..NAD.salvage.pathway.II..PNC.IV.cycle.	All Crohn's Disease	0.0828	0.47	1.	1.
PWY.7761..NAD.salvage.pathway.II..PNC.IV.cycle.	All Ulcerative Colitis	0.387	0.457	0.52	1.
PWY.7790..UMP.biosynthesis.II	All Crohn's Disease	-0.164	0.259	1.	1.
PWY.7790..UMP.biosynthesis.II	All Ulcerative Colitis	-0.327	0.252	0.112	1.
PWY.7791..UMP.biosynthesis.III	All Crohn's Disease	-0.164	0.259	1.	1.
PWY.7791..UMP.biosynthesis.III	All Ulcerative Colitis	-0.327	0.252	0.112	1.
PWY.7851..coenzyme.A.biosynthesis.II..eukaryotic.	All Crohn's Disease	-0.0556	0.238	1.	1.
PWY.7851..coenzyme.A.biosynthesis.II..eukaryotic.	All Ulcerative Colitis	-0.272	0.232	0.132	1.
PWY.7858...5Z..dodecenoate.biosynthesis.II	All Crohn's Disease	2.27	2.29	0.556	1.
PWY.7858...5Z..dodecenoate.biosynthesis.II	All Ulcerative Colitis	3.2	2.2	0.344	1.
PWY.7874..L.threonate.degradation	All Crohn's Disease	4.07	1.36	0.178	1.
PWY.7874..L.threonate.degradation	All Ulcerative Colitis	3.85	1.06	0.137	1.
PWY.7883..anhydromuropeptides.recycling.II	All Crohn's Disease	1.93	1.81	0.475	1.
PWY.7883..anhydromuropeptides.recycling.II	All Ulcerative Colitis	1.35	1.75	0.318	1.
PWY.7942..5.oxo.L.proline.metabolism	All Crohn's Disease	1.12	1.69	0.317	1.
PWY.7942..5.oxo.L.proline.metabolism	All Ulcerative Colitis	4.24	1.74	0.0805	1.
PWY.7953..UDP.N.acetylmuramoyl.pentapeptide.biosynthesis.III meso.diaminopimelate.containing.	All Crohn's Disease	-0.0935	0.229	1.	1.
PWY.7953..UDP.N.acetylmuramoyl.pentapeptide.biosynthesis.III meso.diaminopimelate.containing.	All Ulcerative Colitis	-0.225	0.223	0.172	1.
PWY.7977..L.methionine.biosynthesis.IV	All Crohn's Disease	-0.585	0.399	0.207	1.
PWY.7977..L.methionine.biosynthesis.IV	All Ulcerative Colitis	-0.389	0.388	0.23	1.
PWY.7992..superpathway.of.menaquinol.8.biosynthesis.III	All Crohn's Disease	1.89	0.988	0.222	1.
PWY.8004..Entner.Doudoroff.pathway.I	All Crohn's Disease	-0.181	0.367	1.	1.
PWY.8004..Entner.Doudoroff.pathway.I	All Ulcerative Colitis	-0.0176	0.358	1.	1.
PWY.801..homocysteine.and.cysteine.interconversion	All Crohn's Disease	-1.13	1.37	0.305	1.
PWY.801..homocysteine.and.cysteine.interconversion	All Ulcerative Colitis	-0.855	1.18	0.679	1.
PWY.8073..lipid.IVA.biosynthesis..P..putida.	All Crohn's Disease	0.33	0.755	0.842	1.
PWY.8073..lipid.IVA.biosynthesis..P..putida.	All Ulcerative Colitis	0.367	0.746	0.895	1.
PWY.8131..5..deoxyadenosine.degradation.II	All Crohn's Disease	-0.117	0.606	1.	1.
PWY.8131..5..deoxyadenosine.degradation.II	All Ulcerative Colitis	-0.538	0.59	0.298	1.
PWY.8134..bile.acid.7.beta..dehydroxylation	All Crohn's Disease	-0.0159	0.736	0.708	1.

PWY.8134..bile.acid.7.beta..dehydroxylation	All Ulcerative Colitis	1.05	0.904	0.56	1.
PWY.8178..pentose.phosphate.pathway..non.oxidative.branch..II	All Crohn's Disease	-0.0556	0.278	1.	1.
PWY.8178..pentose.phosphate.pathway..non.oxidative.branch..II	All Ulcerative Colitis	-0.0806	0.271	1.	1.
PWY.8187..L.arginine.degradation.XIII..reductive.Stickland.reaction.	All Crohn's Disease	0.197	0.649	0.685	1.
PWY.8187..L.arginine.degradation.XIII..reductive.Stickland.reaction.	All Ulcerative Colitis	-0.0836	0.632	1.	1.
PWY.8188..L.alanine.degradation.VI..reductive.Stickland.reaction.	All Crohn's Disease	1.08	0.901	0.55	1.
PWY.8188..L.alanine.degradation.VI..reductive.Stickland.reaction.	All Ulcerative Colitis	1.25	0.661	0.392	1.
PWY.8189..L.alanine.degradation.V..oxidative.Stickland.reaction.	All Crohn's Disease	1.08	0.901	0.55	1.
PWY.8189..L.alanine.degradation.V..oxidative.Stickland.reaction.	All Ulcerative Colitis	1.25	0.661	0.392	1.
PWY.821..superpathway.of.sulfur.amino.acid.biosynthesis..Saccharomyces.cerevisiae.	All Crohn's Disease	-0.404	1.57	0.0941	1.
PWY.821..superpathway.of.sulfur.amino.acid.biosynthesis..Saccharomyces.cerevisiae.	All Ulcerative Colitis	0.664	1.58	0.293	1.
PWY.822..fructan.biosynthesis	All Crohn's Disease	-0.373	1.11	0.526	1.
PWY.822..fructan.biosynthesis	All Ulcerative Colitis	-0.152	1.02	0.981	1.
PWY.841..superpathway.of.purine.nucleotides.de.novo.biosynthesis.I	All Crohn's Disease	-0.152	0.389	1.	1.
PWY.841..superpathway.of.purine.nucleotides.de.novo.biosynthesis.I	All Ulcerative Colitis	-0.074	0.379	1.	1.
PWY.922..mevalonate.pathway.I..eukaryotes.and.bacteria.	All Crohn's Disease	0.984	1.04	0.545	1.
PWY.922..mevalonate.pathway.I..eukaryotes.and.bacteria.	All Ulcerative Colitis	2.67	1.02	0.0456	1.
PWY.I9..L.cysteine.biosynthesis.VI..from.L.methionine.	All Ulcerative Colitis	-0.646	0.383	0.0662	1.
PWY0.1061..superpathway.of.L.alanine.biosynthesis	All Crohn's Disease	-0.819	0.925	0.425	1.
PWY0.1061..superpathway.of.L.alanine.biosynthesis	All Ulcerative Colitis	0.143	0.9	1.	1.
PWY0.1241..ADP.L.glycero..beta..D.manno.heptose.biosynthesis	All Crohn's Disease	0.799	1.1	0.685	1.
PWY0.1241..ADP.L.glycero..beta..D.manno.heptose.biosynthesis	All Ulcerative Colitis	0.402	1.07	0.716	1.
PWY0.1261..anhydromuropeptides.recycling.I	All Crohn's Disease	0.491	0.744	0.707	1.
PWY0.1261..anhydromuropeptides.recycling.I	All Ulcerative Colitis	-0.241	0.727	0.88	1.
PWY0.1277..3.phenylpropanoate.and.3..3.hydroxyphenyl.propanoate.degradation	All Crohn's Disease	0.6	2.98	0.972	1.
PWY0.1277..3.phenylpropanoate.and.3..3.hydroxyphenyl.propanoate.degradation	All Ulcerative Colitis	1.73	2.88	0.84	1.
PWY0.1296..purine.ribonucleosides.degradation	All Crohn's Disease	1.57×10^{-04}	0.314	1.	1.
PWY0.1296..purine.ribonucleosides.degradation	All Ulcerative Colitis	-0.155	0.306	1.	1.
PWY0.1297..superpathway.of.purine.deoxyribonucleosides.degradation	All Crohn's Disease	-0.718	0.58	0.274	1.
PWY0.1297..superpathway.of.purine.deoxyribonucleosides.degradation	All Ulcerative Colitis	-0.477	0.565	0.326	1.
PWY0.1298..superpathway.of.pyrimidine.deoxyribonucleosides.degradation	All Crohn's Disease	0.755	0.719	0.262	1.
PWY0.1319..CDP.diacylglycerol.biosynthesis.II	All Crohn's Disease	-0.0274	0.232	1.	1.
PWY0.1319..CDP.diacylglycerol.biosynthesis.II	All Ulcerative Colitis	-0.286	0.226	0.109	1.
PWY0.1337..oleate..beta..oxidation	All Crohn's Disease	3.91	2.58	0.358	1.
PWY0.1337..oleate..beta..oxidation	All Ulcerative Colitis	4.37	2.32	0.258	1.
PWY0.1338..polymyxin.resistance	All Crohn's Disease	3.54	2.77	0.422	1.
PWY0.1338..polymyxin.resistance	All Ulcerative Colitis	3.4	2.59	0.434	1.
PWY0.1415..superpathway.of.heme.b.biosynthesis.from.uroporphyrinogen.III	All Crohn's Disease	2.1	1.34	0.248	1.
PWY0.1415..superpathway.of.heme.b.biosynthesis.from.uroporphyrinogen.III	All Ulcerative Colitis	3.03	1.23	0.0693	1.
PWY0.1477..ethanolamine.utilization	All Crohn's Disease	1.11	0.73	0.119	1.
PWY0.1479..tRNA.processing	All Crohn's Disease	-0.287	0.511	1.	1.
PWY0.1479..tRNA.processing	All Ulcerative Colitis	0.413	0.498	0.521	1.
PWY0.1586..peptidoglycan.maturation..meso.diaminopimelate.containing.	All Crohn's Disease	-0.374	0.329	0.363	1.
PWY0.1586..peptidoglycan.maturation..meso.diaminopimelate.containing.	All Ulcerative Colitis	-0.335	0.32	0.197	1.
PWY0.162..superpathway.of.pyrimidine.ribonucleotides.de.novo.biosynthesis	All Crohn's Disease	-0.105	0.438	1.	1.
PWY0.162..superpathway.of.pyrimidine.ribonucleotides.de.novo.biosynthesis	All Ulcerative Colitis	0.0902	0.426	1.	1.
PWY0.166..superpathway.of.pyrimidine.deoxyribonucleotides de.novo.biosynthesis..E..coli.	All Crohn's Disease	1.02	2.85	0.859	1.
PWY0.166..superpathway.of.pyrimidine.deoxyribonucleotides de.novo.biosynthesis..E..coli.	All Ulcerative Colitis	2.5	2.72	0.439	1.
PWY0.301..L.ascorbate.degradation.I..bacterial..anaerobic.	All Crohn's Disease	2.24	1.75	0.377	1.
PWY0.301..L.ascorbate.degradation.I..bacterial..anaerobic.	All Ulcerative Colitis	4.5	1.69	0.043	1.
PWY0.42..2.methylcitrate.cycle.I	All Crohn's Disease	0.427	2.53	0.84	1.
PWY0.42..2.methylcitrate.cycle.I	All Ulcerative Colitis	2.73	2.47	0.538	1.
PWY0.461..L.lysine.degradation.I	All Crohn's Disease	5.12	2.66	0.513	1.
PWY0.461..L.lysine.degradation.I	All Ulcerative Colitis	3.32	2.11	0.6	1.
PWY0.781..aspartate.superpathway	All Crohn's Disease	0.655	0.805	0.476	1.
PWY0.781..aspartate.superpathway	All Ulcerative Colitis	1.48	0.801	0.185	1.
PWY0.845..superpathway.of.pyridoxal.5..phosphate.biosynthesis.and.salvage	All Crohn's Disease	0.558	0.649	0.343	1.
PWY0.845..superpathway.of.pyridoxal.5..phosphate.biosynthesis.and.salvage	All Ulcerative Colitis	-0.275	0.632	0.57	1.
PWY0.862...5Z..dodecenoate.biosynthesis.I	All Crohn's Disease	0.131	0.792	1.	1.
PWY0.862...5Z..dodecenoate.biosynthesis.I	All Ulcerative Colitis	1.1	0.771	0.204	1.
PWY1G.0..mycothiol.biosynthesis	All Crohn's Disease	-0.868	0.528	0.605	1.
PWY1G.0..mycothiol.biosynthesis	All Ulcerative Colitis	-0.927	0.41	0.429	1.
PWY1ZNC.1..assimilatory.sulfate.reduction.IV	All Crohn's Disease	-0.299	0.799	0.999	1.
PWY1ZNC.1..assimilatory.sulfate.reduction.IV	All Ulcerative Colitis	-0.146	0.782	0.991	1.
PWY3O.4107..NAD.salvage.pathway.V..PNC.V.cycle.	All Crohn's Disease	-1.35	1.05	0.233	1.
PWY3O.4107..NAD.salvage.pathway.V..PNC.V.cycle.	All Ulcerative Colitis	0.72	1.02	0.543	1.
PWY4FS.7..phosphatidylglycerol.biosynthesis.I..plastidic.	All Crohn's Disease	0.0559	0.5	1.	1.
PWY4FS.7..phosphatidylglycerol.biosynthesis.I..plastidic.	All Ulcerative Colitis	-0.185	0.487	0.578	1.
PWY4FS.8..phosphatidylglycerol.biosynthesis.II..non.plastidic.	All Crohn's Disease	0.0558	0.5	1.	1.
PWY4FS.8..phosphatidylglycerol.biosynthesis.II..non.plastidic.	All Ulcerative Colitis	-0.185	0.487	0.578	1.
PWY66.367..ketogenesis	All Crohn's Disease	0.712	2.22	0.785	1.
PWY66.367..ketogenesis	All Ulcerative Colitis	0.309	1.83	0.999	1.
PWY66.389..phytol.degradation	All Crohn's Disease	4.25	2.49	0.201	1.
PWY66.389..phytol.degradation	All Ulcerative Colitis	5.19	2.47	0.115	1.
PWY66.391..fatty.acid..beta..oxidation.VI..mammalian.peroxisome.	All Crohn's Disease	-0.369	0.979	0.683	1.
PWY66.391..fatty.acid..beta..oxidation.VI..mammalian.peroxisome.	All Ulcerative Colitis	0.872	0.959	0.601	1.
PWY66.399..gluconeogenesis.III	All Crohn's Disease	-0.643	0.456	0.394	1.
PWY66.409..superpathway.of.purine.nucleotide.salvage	All Crohn's Disease	-0.39	0.48	0.509	1.

PWY66.409..superpathway.of.purine.nucleotide.salvage	All Ulcerative Colitis	-0.309	0.467	0.403	1.
PWY66.429..fatty.acid.biosynthesis.initiation..mitochondria.	All Crohn's Disease	0.0623	0.246	1.	1.
PWY66.429..fatty.acid.biosynthesis.initiation..mitochondria.	All Ulcerative Colitis	-0.222	0.24	0.205	1.
PYRIDNUCSAL.PWY..NAD.salvage.pathway.I..PNC.VI.cycle.	All Crohn's Disease	-1.02	0.929	0.316	1.
PYRIDNUCSAL.PWY..NAD.salvage.pathway.I..PNC.VI.cycle.	All Ulcerative Colitis	0.774	0.904	0.456	1.
PYRIDNUCSYN.PWY..NAD.de.novo.biosynthesis.I..from.aspartate.	All Crohn's Disease	-0.22	0.246	1.	1.
PYRIDNUCSYN.PWY..NAD.de.novo.biosynthesis.I..from.aspartate.	All Ulcerative Colitis	-0.223	0.239	0.203	1.
PYRIDOXSYN.PWY..pyridoxal.5..phosphate.biosynthesis.I	All Crohn's Disease	0.604	0.717	0.357	1.
PYRIDOXSYN.PWY..pyridoxal.5..phosphate.biosynthesis.I	All Ulcerative Colitis	-0.366	0.698	0.521	1.
RHAMCAT.PWY..L.rhamnose.degradation.I	All Crohn's Disease	0.665	0.35	0.0461	1.
RHAMCAT.PWY..L.rhamnose.degradation.I	All Ulcerative Colitis	0.0871	0.34	1.	1.
RIBOSYN2.PWY..flavin.biosynthesis.I..bacteria.and.plants.	All Crohn's Disease	2.72×10^{-03}	0.304	1.	1.
RIBOSYN2.PWY..flavin.biosynthesis.I..bacteria.and.plants.	All Ulcerative Colitis	-0.194	0.296	0.348	1.
RUMP.PWY..formaldehyde.oxidation.I	All Crohn's Disease	-0.509	0.742	0.806	1.
RUMP.PWY..formaldehyde.oxidation.I	All Ulcerative Colitis	0.612	0.727	0.729	1.
SALVADEHYPOX.PWY..adenosine.nucleotides.degradation.II	All Crohn's Disease	-0.0464	0.528	1.	1.
SALVADEHYPOX.PWY..adenosine.nucleotides.degradation.II	All Ulcerative Colitis	0.0418	0.515	1.	1.
SER.GLYSYN.PWY..superpathway.of.L.serine.and.glycine.biosynthesis.I	All Crohn's Disease	-0.416	0.313	0.278	1.
SER.GLYSYN.PWY..superpathway.of.L.serine.and.glycine.biosynthesis.I	All Ulcerative Colitis	-0.304	0.305	0.21	1.
SO4ASSIM.PWY..assimilatory.sulfate.reduction.I	All Crohn's Disease	0.778	0.832	0.536	1.
SO4ASSIM.PWY..assimilatory.sulfate.reduction.I	All Ulcerative Colitis	2.08	0.814	0.0443	1.
SULFATE.CYS.PWY..superpathway.of.sulfate.assimilation.and.cysteine.biosynthesis	All Crohn's Disease	0.681	0.754	0.551	1.
SULFATE.CYS.PWY..superpathway.of.sulfate.assimilation.and.cysteine.biosynthesis	All Ulcerative Colitis	1.83	0.738	0.0526	1.
TCA..TCA.cycle.I..prokaryotic.	All Crohn's Disease	0.763	1.68	0.505	1.
TCA..TCA.cycle.I..prokaryotic.	All Ulcerative Colitis	1.04	1.64	0.25	1.
TCA.GLYOX.BYPASS..superpathway.of.glyoxylate.bypass.and.TCA	All Crohn's Disease	0.28	2.08	0.486	1.
TCA.GLYOX.BYPASS..superpathway.of.glyoxylate.bypass.and.TCA	All Ulcerative Colitis	1.68	2.06	0.579	1.
THISYNARA.PWY..superpathway.of.thiamine.diphosphate.biosynthesis.III..eukaryotes.	All Crohn's Disease	-0.319	0.286	0.389	1.
THISYNARA.PWY..superpathway.of.thiamine.diphosphate.biosynthesis.III..eukaryotes.	All Ulcerative Colitis	-0.322	0.278	0.153	1.
THRESYN.PWY..superpathway.of.L.threonine.biosynthesis	All Crohn's Disease	-0.467	0.249	0.122	1.
THRESYN.PWY..superpathway.of.L.threonine.biosynthesis	All Ulcerative Colitis	-0.256	0.242	0.167	1.
TRNA.CHARGING.PWY..tRNA.charging	All Crohn's Disease	-0.129	0.242	1.	1.
TRNA.CHARGING.PWY..tRNA.charging	All Ulcerative Colitis	-0.31	0.235	0.103	1.
UDPNAGSYN.PWY..UDP.N.acetyl.D.glucosamine.biosynthesis.I	All Crohn's Disease	-0.418	0.364	0.347	1.
UDPNAGSYN.PWY..UDP.N.acetyl.D.glucosamine.biosynthesis.I	All Ulcerative Colitis	0.189	0.355	1.	1.
UNINTEGRATED	All Crohn's Disease	-0.0386	0.0598	1.	1.
UNINTEGRATED	All Ulcerative Colitis	-0.0869	0.0582	1.	1.
UNMAPPED	All Crohn's Disease	0.167	0.168	1.	1.
UNMAPPED	All Ulcerative Colitis	0.238	0.164	1.	1.
VALSYN.PWY..L.valine.biosynthesis	All Crohn's Disease	-0.277	0.289	1.	1.
VALSYN.PWY..L.valine.biosynthesis	All Ulcerative Colitis	-0.339	0.282	0.141	1.
X1CMET2.PWY..folate.transformations.III..E..coli.	All Crohn's Disease	-0.393	0.327	0.332	1.
X1CMET2.PWY..folate.transformations.III..E..coli.	All Ulcerative Colitis	-0.342	0.319	0.188	1.
PWY.7992..superpathway.of.menaquinol.8.biosynthesis.III	All Ulcerative Colitis	NA	NA	0.016	0.666
AEROBACTINSYN.PWY..aerobactin.biosynthesis	All Crohn's Disease	NA	NA	0.602	1.
AEROBACTINSYN.PWY..aerobactin.biosynthesis	All Ulcerative Colitis	NA	NA	0.866	1.
ARGDEG.PWY..superpathway.of.L.arginine..putrescine and4.aminobutanoate.degradation	All Crohn's Disease	NA	NA	0.678	1.
ARGDEG.PWY..superpathway.of.L.arginine..putrescine and.4.aminobutanoate.degradation	All Ulcerative Colitis	NA	NA	0.511	1.
CARNMET.PWY..L.carnitine.degradation.I	All Crohn's Disease	NA	NA	0.665	1.
CARNMET.PWY..L.carnitine.degradation.I	All Ulcerative Colitis	NA	NA	0.29	1.
CATECHOL.ORTHO.CLEAVAGE.PWY..catechol.degradation.to..beta..ketoadipate	All Crohn's Disease	NA	NA	0.631	1.
CATECHOL.ORTHO.CLEAVAGE.PWY..catechol.degradation.to..beta..ketoadipate	All Ulcerative Colitis	NA	NA	0.732	1.
CHLOROPHYLL.SYN..3.8.divinyl.chlorophyllide.a.biosynthesis.I aerobic..light.dependent.	All Crohn's Disease	NA	NA	0.975	1.
CHLOROPHYLL.SYN..3.8.divinyl.chlorophyllide.a.biosynthesis.I aerobic..light.dependent.	All Ulcerative Colitis	NA	NA	0.678	1.
CRNFORCAT.PWY..creatinine.degradation.I	All Ulcerative Colitis	NA	NA	0.245	1.
DARABCATK12.PWY..D.arabinose.degradation.I	All Crohn's Disease	NA	NA	0.639	1.
DARABCATK12.PWY..D.arabinose.degradation.I	All Ulcerative Colitis	NA	NA	0.203	1.
DENITRIFICATION.PWY..nitrate.reduction.I..denitrification.	All Crohn's Disease	NA	NA	0.436	1.
DENITRIFICATION.PWY..nitrate.reduction.I..denitrification.	All Ulcerative Colitis	NA	NA	0.796	1.
DENOVOPURINE2.PWY..superpathway.of.purine.nucleotides.de.novo.biosynthesis.II	All Crohn's Disease	NA	NA	0.345	1.
DENOVOPURINE2.PWY..superpathway.of.purine.nucleotides.de.novo.biosynthesis.II	All Ulcerative Colitis	NA	NA	0.293	1.
GLYCOLYSIS.TCA.GLYOX.BYPASS..superpathway.of.glycolysis pyruvate.dehydrogenase..TCA..and.glyoxylate.bypass	All Crohn's Disease	NA	NA	0.635	1.
GLYCOLYSIS.TCA.GLYOX.BYPASS..superpathway.of.glycolysis pyruvate.dehydrogenase..TCA..and.glyoxylate.bypass	All Ulcerative Colitis	NA	NA	0.263	1.
KDO.NAGLIPASYN.PWY..superpathway.of..Kdo.2.lipid.A.biosynthesis	All Crohn's Disease	NA	NA	0.45	1.
KDO.NAGLIPASYN.PWY..superpathway.of..Kdo.2.lipid.A.biosynthesis	All Ulcerative Colitis	NA	NA	0.257	1.
LPSSYN.PWY..superpathway.of.lipopolysaccharide.biosynthesis	All Crohn's Disease	NA	NA	0.653	1.
LPSSYN.PWY..superpathway.of.lipopolysaccharide.biosynthesis	All Ulcerative Colitis	NA	NA	0.3	1.
ORNARGDEG.PWY..superpathway.of.L.arginine.and.L.ornithine.degradation	All Crohn's Disease	NA	NA	0.678	1.
ORNARGDEG.PWY..superpathway.of.L.arginine.and.L.ornithine.degradation	All Ulcerative Colitis	NA	NA	0.511	1.
P162.PWY..L.glutamate.degradation.V..via.hydroxyglutarate.	All Crohn's Disease	NA	NA	0.697	1.
P162.PWY..L.glutamate.degradation.V..via.hydroxyglutarate.	All Ulcerative Colitis	NA	NA	0.757	1.
P163.PWY..L.lysine.fermentation.to.acetate.and.butanoate	All Crohn's Disease	NA	NA	0.454	1.
P163.PWY..L.lysine.fermentation.to.acetate.and.butanoate	All Ulcerative Colitis	NA	NA	0.689	1.
P221.PWY..octane.oxidation	All Crohn's Disease	NA	NA	0.188	1.

P221.PWY..octane.oxidation	All Ulcerative Colitis	NA	NA	0.239	1.
P23.PWY..reductive.TCA.cycle.I	All Crohn's Disease	NA	NA	0.195	1.
P23.PWY..reductive.TCA.cycle.I	All Ulcerative Colitis	NA	NA	0.164	1.
P562.PWY..myo.inositol.degradation.I	All Crohn's Disease	NA	NA	0.644	1.
P562.PWY..myo.inositol.degradation.I	All Ulcerative Colitis	NA	NA	0.311	1.
PRPP.PWY..superpathway.of.histidine..purine..and.pyrimidine.biosynthesis	All Crohn's Disease	NA	NA	0.345	1.
PRPP.PWY..superpathway.of.histidine..purine..and.pyrimidine.biosynthesis	All Ulcerative Colitis	NA	NA	0.293	1.
PWY.3781..aerobic.respiration.I..cytochrome.c.	All Crohn's Disease	NA	NA	0.433	1.
PWY.3781..aerobic.respiration.I..cytochrome.c.	All Ulcerative Colitis	NA	NA	0.104	1.
PWY.5004..superpathway.of.L.citrulline.metabolism	All Crohn's Disease	NA	NA	0.266	1.
PWY.5004..superpathway.of.L.citrulline.metabolism	All Ulcerative Colitis	NA	NA	0.714	1.
PWY.5028..L.histidine.degradation.II	All Crohn's Disease	NA	NA	0.998	1.
PWY.5028..L.histidine.degradation.II	All Ulcerative Colitis	NA	NA	0.316	1.
PWY.5156..superpathway.of.fatty.acid.biosynthesis.II..plant.	All Crohn's Disease	NA	NA	0.998	1.
PWY.5156..superpathway.of.fatty.acid.biosynthesis.II..plant.	All Ulcerative Colitis	NA	NA	0.41	1.
PWY.5180..toluene.degradation.I..aerobic...via.o.cresol.	All Crohn's Disease	NA	NA	0.665	1.
PWY.5180..toluene.degradation.I..aerobic...via.o.cresol.	All Ulcerative Colitis	NA	NA	0.29	1.
PWY.5392..reductive.TCA.cycle.II	All Crohn's Disease	NA	NA	0.264	1.
PWY.5392..reductive.TCA.cycle.II	All Ulcerative Colitis	NA	NA	0.217	1.
PWY.5415..catechol.degradation.I..meta.cleavage.pathway.	All Crohn's Disease	NA	NA	0.475	1.
PWY.5415..catechol.degradation.I..meta.cleavage.pathway.	All Ulcerative Colitis	NA	NA	0.289	1.
PWY.5692..allantoin.degradation.to.glyoxylate.II	All Crohn's Disease	NA	NA	0.618	1.
PWY.5692..allantoin.degradation.to.glyoxylate.II	All Ulcerative Colitis	NA	NA	0.505	1.
PWY.5705..allantoin.degradation.to.glyoxylate.III	All Crohn's Disease	NA	NA	0.451	1.
PWY.5705..allantoin.degradation.to.glyoxylate.III	All Ulcerative Colitis	NA	NA	0.255	1.
PWY.5723..Rubisco.shunt	All Crohn's Disease	NA	NA	0.0628	1.
PWY.5723..Rubisco.shunt	All Ulcerative Colitis	NA	NA	0.118	1.
PWY.5855..ubiquinol.7.biosynthesis..early.decarboxylation.	All Crohn's Disease	NA	NA	0.253	1.
PWY.5855..ubiquinol.7.biosynthesis..early.decarboxylation.	All Ulcerative Colitis	NA	NA	0.238	1.
PWY.6138..CMP.N.acetylneuraminate.biosynthesis.I..eukaryotes.	All Crohn's Disease	NA	NA	0.623	1.
PWY.6138..CMP.N.acetylneuraminate.biosynthesis.I..eukaryotes.	All Ulcerative Colitis	NA	NA	0.904	1.
PWY.6143..CMP.pseudaminate.biosynthesis	All Ulcerative Colitis	NA	NA	0.059	1.
PWY.6185..4.methylcatechol.degradation..ortho.cleavage.	All Crohn's Disease	NA	NA	0.499	1.
PWY.6185..4.methylcatechol.degradation..ortho.cleavage.	All Ulcerative Colitis	NA	NA	0.765	1.
PWY.6215..4.chlorobenzoate.degradation	All Crohn's Disease	NA	NA	0.631	1.
PWY.6215..4.chlorobenzoate.degradation	All Ulcerative Colitis	NA	NA	0.732	1.
PWY.6318..L.phenylalanine.degradation.IV..mammalian..via.side.chain.	All Crohn's Disease	-1.94	NA	0.502	1.
PWY.6318..L.phenylalanine.degradation.IV..mammalian..via.side.chain.	All Ulcerative Colitis	-0.604	NA	0.741	1.
PWY.6328..L.lysine.degradation.X	All Crohn's Disease	NA	NA	0.931	1.
PWY.6328..L.lysine.degradation.X	All Ulcerative Colitis	NA	NA	0.292	1.
PWY.6396..superpathway.of.2.3.butanediol.biosynthesis	All Crohn's Disease	NA	NA	0.472	1.
PWY.6396..superpathway.of.2.3.butanediol.biosynthesis	All Ulcerative Colitis	NA	NA	0.378	1.
PWY.6471..peptidoglycan.biosynthesis.IV..Enterococcus.faecium.	All Crohn's Disease	NA	NA	0.968	1.
PWY.6471..peptidoglycan.biosynthesis.IV..Enterococcus.faecium.	All Ulcerative Colitis	NA	NA	0.378	1.
PWY.6562..norspermidine.biosynthesis	All Crohn's Disease	NA	NA	0.25	1.
PWY.6562..norspermidine.biosynthesis	All Ulcerative Colitis	NA	NA	0.51	1.
PWY.6708..ubiquinol.8.biosynthesis..early.decarboxylation.	All Crohn's Disease	NA	NA	0.651	1.
PWY.6708..ubiquinol.8.biosynthesis..early.decarboxylation.	All Ulcerative Colitis	NA	NA	0.386	1.
PWY.6922..L.N.delta..acetylornithine.biosynthesis	All Crohn's Disease	NA	NA	0.0961	1.
PWY.6922..L.N.delta..acetylornithine.biosynthesis	All Ulcerative Colitis	NA	NA	0.149	1.
PWY.7031..protein.N.glycosylation..bacterial.	All Crohn's Disease	NA	NA	0.682	1.
PWY.7031..protein.N.glycosylation..bacterial.	All Ulcerative Colitis	NA	NA	0.715	1.
PWY.7039..phosphatidate.metabolism..as.a.signaling.molecule	All Crohn's Disease	NA	NA	0.229	1.
PWY.7039..phosphatidate.metabolism..as.a.signaling.molecule	All Ulcerative Colitis	1.99	NA	0.44	1.
PWY.7159..3.8.divinyl.chlorophyllide.a.biosynthesis.III..aerobic..light.independent.	All Crohn's Disease	NA	NA	0.973	1.
PWY.7159..3.8.divinyl.chlorophyllide.a.biosynthesis.III..aerobic..light.independent.	All Ulcerative Colitis	NA	NA	0.679	1.
PWY.7187..pyrimidine.deoxyribonucleotides.de.novo.biosynthesis.II	All Crohn's Disease	NA	NA	0.345	1.
PWY.7187..pyrimidine.deoxyribonucleotides.de.novo.biosynthesis.II	All Ulcerative Colitis	NA	NA	0.293	1.
PWY.7200..superpathway.of.pyrimidine.deoxyribonucleoside.salvage	All Crohn's Disease	NA	NA	0.455	1.
PWY.7200..superpathway.of.pyrimidine.deoxyribonucleoside.salvage	All Ulcerative Colitis	NA	NA	0.396	1.
PWY.7254..TCA.cycle.VII..acetate.producers.	All Crohn's Disease	NA	NA	0.195	1.
PWY.7254..TCA.cycle.VII..acetate.producers.	All Ulcerative Colitis	NA	NA	0.121	1.
PWY.7268..cytosolic.NADPH.production..yeast.	All Crohn's Disease	NA	NA	0.614	1.
PWY.7268..cytosolic.NADPH.production..yeast.	All Ulcerative Colitis	NA	NA	0.891	1.
PWY.7269..mitochondrial.NADPH.production..yeast.	All Crohn's Disease	NA	NA	0.343	1.
PWY.7269..mitochondrial.NADPH.production..yeast.	All Ulcerative Colitis	NA	NA	0.295	1.
PWY.7279..aerobic.respiration.II..cytochrome.c...yeast.	All Crohn's Disease	NA	NA	0.667	1.
PWY.7279..aerobic.respiration.II..cytochrome.c...yeast.	All Ulcerative Colitis	NA	NA	0.378	1.
PWY.7294..D.xylose.degradation.IV	All Crohn's Disease	NA	NA	0.455	1.
PWY.7294..D.xylose.degradation.IV	All Ulcerative Colitis	NA	NA	0.686	1.
PWY.7388..octanoyl..acyl.carrier.protein..biosynthesis..mitochondria..yeast.	All Crohn's Disease	NA	NA	0.485	1.
PWY.7388..octanoyl..acyl.carrier.protein..biosynthesis..mitochondria..yeast.	All Ulcerative Colitis	NA	NA	0.16	1.
PWY.7391..isoprene.biosynthesis.II..engineered.	All Crohn's Disease	NA	NA	0.678	1.
PWY.7391..isoprene.biosynthesis.II..engineered.	All Ulcerative Colitis	NA	NA	0.287	1.
PWY.7399..methylphosphonate.degradation.II	All Crohn's Disease	2.66	NA	0.519	1.
PWY.7399..methylphosphonate.degradation.II	All Ulcerative Colitis	4.58	NA	0.482	1.
PWY.7409..phospholipid.remodeling..phosphatidylethanolamine..yeast.	All Crohn's Disease	NA	NA	0.632	1.
PWY.7409..phospholipid.remodeling..phosphatidylethanolamine..yeast.	All Ulcerative Colitis	NA	NA	0.339	1.
PWY.7446..sulfoquinovose.degradation.I	All Crohn's Disease	NA	NA	0.949	1.
PWY.7446..sulfoquinovose.degradation.I	All Ulcerative Colitis	NA	NA	0.388	1.

PWY.7723..bacterial.bioluminescence	All Crohn's Disease	0.923	NA	0.516	1.
PWY.7723..bacterial.bioluminescence	All Ulcerative Colitis	NA	NA	0.245	1.
PWY.7783..plasmalogen.degradation	All Crohn's Disease	NA	NA	0.444	1.
PWY.7783..plasmalogen.degradation	All Ulcerative Colitis	NA	NA	0.432	1.
PWY.7805...aminomethyl.phosphonate.degradation	All Crohn's Disease	NA	NA	0.645	1.
PWY.7805...aminomethyl.phosphonate.degradation	All Ulcerative Colitis	NA	NA	0.31	1.
PWY.7807..glyphosate.degradation.III	All Crohn's Disease	NA	NA	0.645	1.
PWY.7807..glyphosate.degradation.III	All Ulcerative Colitis	NA	NA	0.31	1.
PWY.7820..teichuronic.acid.biosynthesis..B..subtilis.168.	All Crohn's Disease	NA	NA	0.985	1.
PWY.7820..teichuronic.acid.biosynthesis..B..subtilis.168.	All Ulcerative Colitis	NA	NA	0.499	1.
PWY.7873..D.erythronate.degradation.II	All Crohn's Disease	NA	NA	0.651	1.
PWY.7873..D.erythronate.degradation.II	All Ulcerative Colitis	NA	NA	0.386	1.
PWY.8086...S..lactate.fermentation.to.propanoate..acetate.and.hydrogen	All Crohn's Disease	NA	NA	0.229	1.
PWY.8086...S..lactate.fermentation.to.propanoate..acetate.and.hydrogen	All Ulcerative Colitis	3.72	NA	0.44	1.
PWY.8190..L.glutamate.degradation.XI..reductive.Stickland.reaction.	All Crohn's Disease	NA	NA	0.468	1.
PWY.8190..L.glutamate.degradation.XI..reductive.Stickland.reaction.	All Ulcerative Colitis	NA	NA	0.294	1.
PWY0.1221..putrescine.degradation.II	All Crohn's Disease	NA	NA	0.94	1.
PWY0.1221..putrescine.degradation.II	All Ulcerative Colitis	NA	NA	0.543	1.
PWY0.1533..methylphosphonate.degradation.I	All Crohn's Disease	NA	NA	0.645	1.
PWY0.1533..methylphosphonate.degradation.I	All Ulcerative Colitis	NA	NA	0.31	1.
PWY0.41..allantoin.degradation.IV..anaerobic.	All Crohn's Disease	NA	NA	0.637	1.
PWY0.41..allantoin.degradation.IV..anaerobic.	All Ulcerative Colitis	NA	NA	0.259	1.
PWY490.3..nitrate.reduction.VI..assimilatory.	All Crohn's Disease	NA	NA	0.471	1.
PWY490.3..nitrate.reduction.VI..assimilatory.	All Ulcerative Colitis	NA	NA	0.99	1.
PWY66.388..ceramide.degradation.by..alpha..oxidation	All Crohn's Disease	NA	NA	0.424	1.
PWY66.388..ceramide.degradation.by..alpha..oxidation	All Ulcerative Colitis	NA	NA	0.714	1.
PWY66.430..myristate.biosynthesis..mitochondria.	All Crohn's Disease	NA	NA	0.485	1.
PWY66.430..myristate.biosynthesis..mitochondria.	All Ulcerative Colitis	NA	NA	0.16	1.
REDCITCYC..TCA.cycle.VI..Helicobacter.	All Crohn's Disease	NA	NA	0.0977	1.
REDCITCYC..TCA.cycle.VI..Helicobacter.	All Ulcerative Colitis	NA	NA	0.0776	1.
THREOCAT.PWY..superpathway.of.L.threonine.metabolism	All Crohn's Disease	NA	NA	0.712	1.
THREOCAT.PWY..superpathway.of.L.threonine.metabolism	All Ulcerative Colitis	NA	NA	0.582	1.
UBISYN.PWY..superpathway.of.ubiquinol.8.biosynthesis..early.decarboxylation.	All Crohn's Disease	NA	NA	0.651	1.
UBISYN.PWY..superpathway.of.ubiquinol.8.biosynthesis..early.decarboxylation.	All Ulcerative Colitis	NA	NA	0.386	1.
UDPNACETYLGALSYN.PWY..UDP.N.acetyl.D.glucosamine.biosynthesis.II	All Crohn's Disease	NA	NA	0.247	1.
UDPNACETYLGALSYN.PWY..UDP.N.acetyl.D.glucosamine.biosynthesis.II	All Ulcerative Colitis	NA	NA	0.697	1.
URDEGR.PWY..superpathway.of.allantoin.degradation.in.plants	All Crohn's Disease	NA	NA	0.618	1.
URDEGR.PWY..superpathway.of.allantoin.degradation.in.plants	All Ulcerative Colitis	NA	NA	0.505	1.
URSIN.PWY..ureide.biosynthesis	All Crohn's Disease	NA	NA	0.631	1.
URSIN.PWY..ureide.biosynthesis	All Ulcerative Colitis	NA	NA	0.732	1.
X3.HYDROXYPHENYLACETATE.DEGRADATION.PWY 4.hydroxyphenylacetate.degradation	All Crohn's Disease	0.376	NA	0.483	1.
X3.HYDROXYPHENYLACETATE.DEGRADATION.PWY 4.hydroxyphenylacetate.degradation	All Ulcerative Colitis	2.07	NA	0.716	1.
