# Supplementary material for: Multiomics-Based Profiling of the Fecal Microbiome Reveals Potential Disease-Specific Signatures in Pediatric IBD (PIBD)
Source: Biomolecules. 2025 May 21;15(5):746. doi: 10.3390/biom15050746 (PMC12109367; doi:10.3390/biom15050746)
Supplement: Supplementary file 1 [file biomolecules-15-00746-s001.zip › supplemental9-maaslin-diseasegroup-pathwayprevalence.pdf]

Supplemental Table S9

Differential prevalence of MetaCyc functional pathways in UC and Crohn's as compared to healthy microbiome, calculated with MaAsLin3.

Pathway	Condition	Effect Size	Standard Error	P-Value	Q-Value
PWY.7992..superpathway.of.menaquinol.8.biosynthesis.III	All Ulcerative Colitis	-3.72	1.54	0.016	0.666
GLYCOL.GLYOXDEG.PWY..superpathway.of.glycol.metabolism.and.degradation	All Ulcerative Colitis	0.499	1.18	0.027	0.875
PWY.6902..chitin.degradation.II..Vibrio.	All Ulcerative Colitis	-0.275	2.29	0.0254	0.875
PWY.7204..pyridoxal.5..phosphate.salvage.II..plants.	All Ulcerative Colitis	0.969	1.19	0.0269	0.875
PWY.5130..2.oxobutanoate.degradation.I	All Crohn's Disease	-0.998	2.17	0.0309	0.901
PWY.7383..anaerobic.energy.metabolism..invertebrates..cytosol.	All Ulcerative Colitis	-0.908	2.21	0.0296	0.901
PWY.7616..methanol.oxidation.to.carbon.dioxide	All Ulcerative Colitis	-0.402	1.24	0.0303	0.901
HEME.BIOSYNTHESIS.II.1..heme.b.biosynthesis.V..aerobic.	All Ulcerative Colitis	-0.765	1.18	0.0326	0.903
PWY66.399..gluconeogenesis.III	All Ulcerative Colitis	-0.908	2.21	0.0328	0.903
AEROBACTINSYN.PWY..aerobactin.biosynthesis	All Crohn's Disease	1.14	2.19	0.602	1.
AEROBACTINSYN.PWY..aerobactin.biosynthesis	All Ulcerative Colitis	0.387	2.29	0.866	1.
ALLANTOINDEG.PWY..superpathway.of.allantoin.degradation.in.yeast	All Crohn's Disease	-2.27	2.13	0.489	1.
ALLANTOINDEG.PWY..superpathway.of.allantoin.degradation.in.yeast	All Ulcerative Colitis	-1.25	2.18	0.812	1.
ARGDEG.PWY..superpathway.of.L.arginine..putrescine and.4.aminobutanoate.degradation	All Crohn's Disease	0.903	2.17	0.678	1.
ARGDEG.PWY..superpathway.of.L.arginine..putrescine and.4.aminobutanoate.degradation	All Ulcerative Colitis	1.39	2.12	0.511	1.
ARGININE.SYN4.PWY..L.ornithine.biosynthesis.II	All Crohn's Disease	0.101	2.37	0.892	1.
ARGININE.SYN4.PWY..L.ornithine.biosynthesis.II	All Ulcerative Colitis	-0.863	2.16	0.904	1.
AST.PWY..L.arginine.degradation.II..AST.pathway.	All Crohn's Disease	0.2	1.23	0.846	1.
AST.PWY..L.arginine.degradation.II..AST.pathway.	All Ulcerative Colitis	0.56	1.18	0.274	1.
CARNMET.PWY..L.carnitine.degradation.I	All Crohn's Disease	0.939	2.17	0.665	1.
CARNMET.PWY..L.carnitine.degradation.I	All Ulcerative Colitis	2.2	2.08	0.29	1.
CATECHOL.ORTHO.CLEAVAGE.PWY..catechol.degradation.to..beta..ketoadipate	All Crohn's Disease	1.04	2.17	0.631	1.
CATECHOL.ORTHO.CLEAVAGE.PWY..catechol.degradation.to..beta..ketoadipate	All Ulcerative Colitis	0.743	2.17	0.732	1.
CENTFERM.PWY..pyruvate.fermentation.to.butanoate	All Crohn's Disease	-0.999	2.17	0.874	1.
CENTFERM.PWY..pyruvate.fermentation.to.butanoate	All Ulcerative Colitis	-1.39	2.11	0.76	1.
CHLOROPHYLL.SYN..3.8.divinyl.chlorophyllide.a.biosynthesis.I aerobic..light.dependent.	All Crohn's Disease	-0.0738	2.37	0.975	1.
CHLOROPHYLL.SYN..3.8.divinyl.chlorophyllide.a.biosynthesis.I aerobic..light.dependent.	All Ulcerative Colitis	0.895	2.15	0.678	1.
CRNFORCAT.PWY..creatinine.degradation.I	All Crohn's Disease	0.171	1.23	0.988	1.
CRNFORCAT.PWY..creatinine.degradation.I	All Ulcerative Colitis	-1.78	1.53	0.245	1.
DARABCATK12.PWY..D.arabinose.degradation.I	All Crohn's Disease	1.02	2.17	0.639	1.
DARABCATK12.PWY..D.arabinose.degradation.I	All Ulcerative Colitis	2.64	2.07	0.203	1.
DENITRIFICATION.PWY..nitrate.reduction.I..denitrification.	All Crohn's Disease	1.65	2.12	0.436	1.
DENITRIFICATION.PWY..nitrate.reduction.I..denitrification.	All Ulcerative Colitis	0.571	2.21	0.796	1.
DENOVOPURINE2.PWY..superpathway.of.purine.nucleotides.de.novo.biosynthesis.II	All Crohn's Disease	1.98	2.1	0.345	1.
DENOVOPURINE2.PWY..superpathway.of.purine.nucleotides.de.novo.biosynthesis.II	All Ulcerative Colitis	2.18	2.08	0.293	1.
ECASYN.PWY..enterobacterial.common.antigen.biosynthesis	All Crohn's Disease	0.167	1.23	0.506	1.
ECASYN.PWY..enterobacterial.common.antigen.biosynthesis	All Ulcerative Colitis	0.926	1.18	0.49	1.
FAO.PWY..fatty.acid..beta..oxidation.I..generic.	All Crohn's Disease	0.95	1.2	0.675	1.
FAO.PWY..fatty.acid..beta..oxidation.I..generic.	All Ulcerative Colitis	1.08	1.18	0.592	1.
FASYN.INITIAL.PWY..superpathway.of.fatty.acid.biosynthesis.initiation..E..coli.	All Crohn's Disease	-1.65	2.12	0.0876	1.
FASYN.INITIAL.PWY..superpathway.of.fatty.acid.biosynthesis.initiation..E..coli.	All Ulcerative Colitis	-2.48	2.07	0.409	1.
FOLSYN.PWY..superpathway.of.tetrahydrofolate.biosynthesis.and.salvage	All Crohn's Disease	-0.0716	2.37	0.999	1.
FOLSYN.PWY..superpathway.of.tetrahydrofolate.biosynthesis.and.salvage	All Ulcerative Colitis	-0.268	2.3	0.991	1.
GALACT.GLUCUROCAT.PWY superpathway.of.hexuronide.and.hexuronate.degradation	All Crohn's Disease	-0.0716	2.37	0.407	1.
GALACT.GLUCUROCAT.PWY superpathway.of.hexuronide.and.hexuronate.degradation	All Ulcerative Colitis	-0.268	2.3	0.0682	1.
GALACTARDEG.PWY..D.galactarate.degradation.I	All Crohn's Disease	-1.05	2.17	0.737	1.
GALACTARDEG.PWY..D.galactarate.degradation.I	All Ulcerative Colitis	0.14	2.35	0.592	1.
GALACTITOLCAT.PWY..galactitol.degradation	All Crohn's Disease	-0.603	1.21	0.839	1.
GALACTITOLCAT.PWY..galactitol.degradation	All Ulcerative Colitis	0.125	1.22	0.837	1.
GLUCARDEG.PWY..D.glucarate.degradation.I	All Crohn's Disease	-1.09	2.17	0.51	1.
GLUCARDEG.PWY..D.glucarate.degradation.I	All Ulcerative Colitis	-0.364	2.26	0.415	1.
GLUCARGALACTSUPER.PWY superpathway.of.D.glucarate.and.D.galactarate.degradation	All Crohn's Disease	-1.05	2.17	0.737	1.
GLUCARGALACTSUPER.PWY superpathway.of.D.glucarate.and.D.galactarate.degradation	All Ulcerative Colitis	0.14	2.35	0.592	1.
GLUCOSE1PMETAB.PWY..glucose.and.glucose.1.phosphate.degradation	All Crohn's Disease	0.765	1.35	0.756	1.
GLUCOSE1PMETAB.PWY..glucose.and.glucose.1.phosphate.degradation	All Ulcerative Colitis	-0.044	1.22	0.605	1.
GLUDEG.I.PWY..GABA.shunt	All Crohn's Disease	-0.0562	2.37	0.634	1.
GLUDEG.I.PWY..GABA.shunt	All Ulcerative Colitis	-0.496	2.23	0.904	1.
GLYCOCAT.PWY..glycogen.degradation.I	All Crohn's Disease	-0.0716	2.37	0.631	1.
GLYCOCAT.PWY..glycogen.degradation.I	All Ulcerative Colitis	-0.268	2.3	0.991	1.
GLYCOL.GLYOXDEG.PWY..superpathway.of.glycol.metabolism.and.degradation	All Crohn's Disease	0.222	1.23	0.0944	1.
GLYCOLYSIS.TCA.GLYOX.BYPASS..superpathway.of.glycolysis pyruvate.dehydrogenase..TCA..and.glyoxylate.bypass	All Crohn's Disease	1.03	2.17	0.635	1.
GLYCOLYSIS.TCA.GLYOX.BYPASS..superpathway.of.glycolysis pyruvate.dehydrogenase..TCA..and.glyoxylate.bypass	All Ulcerative Colitis	2.32	2.07	0.263	1.
GLYOXYLATE.BYPASS..glyoxylate.cycle	All Crohn's Disease	2.7	1.34	0.0867	1.
GLYOXYLATE.BYPASS..glyoxylate.cycle	All Ulcerative Colitis	1.38	1.18	0.428	1.

GOLPDLCAT.PWY..superpathway.of.glycerol.degradation.to.1.3.propanediol	All Crohn's Disease	-0.0716	2.37	0.795	1.
GOLPDLCAT.PWY..superpathway.of.glycerol.degradation.to.1.3.propanediol	All Ulcerative Colitis	-0.268	2.3	0.416	1.
HCAMHPDEG.PWY..3.phenylpropanoate.and 3..3.hydroxyphenyl.propanoate.degradation.to.2.hydroxypentadienoate	All Crohn's Disease	0.168	1.23	0.96	1.
HCAMHPDEG.PWY..3.phenylpropanoate.and 3..3.hydroxyphenyl.propanoate.degradation.to.2.hydroxypentadienoate	All Ulcerative Colitis	0.332	1.19	0.896	1.
HEME.BIOSYNTHESIS.II..heme.b.biosynthesis.I..aerobic.	All Crohn's Disease	-0.0287	2.36	0.805	1.
HEME.BIOSYNTHESIS.II..heme.b.biosynthesis.I..aerobic.	All Ulcerative Colitis	-0.722	2.18	0.533	1.
HEME.BIOSYNTHESIS.II.1..heme.b.biosynthesis.V..aerobic.	All Crohn's Disease	-0.964	1.21	0.317	1.
HEXITOLDEGSUPER.PWY..superpathway.of.hexitol.degradation..bacteria.	All Crohn's Disease	-0.603	1.21	0.678	1.
HEXITOLDEGSUPER.PWY..superpathway.of.hexitol.degradation..bacteria.	All Ulcerative Colitis	0.125	1.22	0.619	1.
HOMOSER.METSYN.PWY..L.methionine.biosynthesis.I	All Crohn's Disease	1.83	1.64	0.459	1.
HOMOSER.METSYN.PWY..L.methionine.biosynthesis.I	All Ulcerative Colitis	0.457	1.25	0.137	1.
KDO.NAGLIPASYN.PWY..superpathway.of..Kdo.2.lipid.A.biosynthesis	All Crohn's Disease	1.6	2.12	0.45	1.
KDO.NAGLIPASYN.PWY..superpathway.of..Kdo.2.lipid.A.biosynthesis	All Ulcerative Colitis	2.35	2.07	0.257	1.
KETOGLUCONMET.PWY..ketogluconate.metabolism	All Crohn's Disease	-0.0626	1.12	0.554	1.
KETOGLUCONMET.PWY..ketogluconate.metabolism	All Ulcerative Colitis	0.624	1.11	0.353	1.
LIPA.CORESYN.PWY..lipid.A.core.biosynthesis..E..coli.K.12.	All Crohn's Disease	-0.855	1.34	0.287	1.
LIPA.CORESYN.PWY..lipid.A.core.biosynthesis..E..coli.K.12.	All Ulcerative Colitis	0.33	1.19	0.272	1.
LIPASYN.PWY..phospholipases	All Crohn's Disease	-0.25	1.26	0.975	1.
LIPASYN.PWY..phospholipases	All Ulcerative Colitis	-0.0406	1.21	0.75	1.
LPSSYN.PWY..superpathway.of.lipopolysaccharide.biosynthesis	All Crohn's Disease	0.975	2.17	0.653	1.
LPSSYN.PWY..superpathway.of.lipopolysaccharide.biosynthesis	All Ulcerative Colitis	2.15	2.08	0.3	1.
MET.SAM.PWY..superpathway.of.S.adenosyl.L.methionine.biosynthesis	All Crohn's Disease	1.83	1.64	0.459	1.
MET.SAM.PWY..superpathway.of.S.adenosyl.L.methionine.biosynthesis	All Ulcerative Colitis	0.457	1.25	0.142	1.
METH.ACETATE.PWY..methanogenesis.from.acetate	All Crohn's Disease	-0.0712	2.37	0.63	1.
METH.ACETATE.PWY..methanogenesis.from.acetate	All Ulcerative Colitis	-0.275	2.29	0.553	1.
METHGLYUT.PWY..superpathway.of.methylglyoxal.degradation	All Crohn's Disease	0.22	1.26	0.394	1.
METHGLYUT.PWY..superpathway.of.methylglyoxal.degradation	All Ulcerative Colitis	0.51	1.26	0.246	1.
METSYN.PWY..superpathway.of.L.homoserine.and.L.methionine.biosynthesis	All Crohn's Disease	1.83	1.64	0.459	1.
METSYN.PWY..superpathway.of.L.homoserine.and.L.methionine.biosynthesis	All Ulcerative Colitis	0.457	1.25	0.141	1.
NAD.BIOSYNTHESIS.II..NAD.salvage.pathway.III..to.nicotinamide.riboside.	All Crohn's Disease	-2.49	2.09	0.412	1.
NAD.BIOSYNTHESIS.II..NAD.salvage.pathway.III..to.nicotinamide.riboside.	All Ulcerative Colitis	-1.06	2.15	0.857	1.
NAGLIPASYN.PWY..lipid.IVA.biosynthesis..E..coli.	All Crohn's Disease	0.0654	2.37	0.842	1.
NAGLIPASYN.PWY..lipid.IVA.biosynthesis..E..coli.	All Ulcerative Colitis	-0.901	2.15	0.895	1.
ORNARGDEG.PWY..superpathway.of.L.arginine.and.L.ornithine.degradation	All Crohn's Disease	0.903	2.17	0.678	1.
ORNARGDEG.PWY..superpathway.of.L.arginine.and.L.ornithine.degradation	All Ulcerative Colitis	1.39	2.12	0.511	1.
ORNDEG.PWY..superpathway.of.ornithine.degradation	All Crohn's Disease	0.16	1.27	0.716	1.
ORNDEG.PWY..superpathway.of.ornithine.degradation	All Ulcerative Colitis	0.0969	1.25	0.156	1.
P105.PWY..TCA.cycle.IV..2.oxoglutarate.decarboxylase.	All Crohn's Disease	2.75	1.58	0.156	1.
P105.PWY..TCA.cycle.IV..2.oxoglutarate.decarboxylase.	All Ulcerative Colitis	2.07	1.29	0.206	1.
P108.PWY..pyruvate.fermentation.to.propanoate.I	All Crohn's Disease	1.3	1.21	0.406	1.
P108.PWY..pyruvate.fermentation.to.propanoate.I	All Ulcerative Colitis	2.	1.22	0.147	1.
P122.PWY..heterolactic.fermentation	All Crohn's Disease	0.893	1.21	0.709	1.
P122.PWY..heterolactic.fermentation	All Ulcerative Colitis	1.57	1.21	0.349	1.
P124.PWY..Bifidobacterium.shunt	All Crohn's Disease	-1.63	2.12	0.639	1.
P124.PWY..Bifidobacterium.shunt	All Ulcerative Colitis	-1.62	2.1	0.687	1.
P125.PWY..superpathway.of..R.R..butanediol.biosynthesis	All Crohn's Disease	-1.73	2.13	0.558	1.
P125.PWY..superpathway.of..R.R..butanediol.biosynthesis	All Ulcerative Colitis	-1.1	2.17	0.85	1.
P162.PWY..L.glutamate.degradation.V..via.hydroxyglutarate.	All Crohn's Disease	0.85	2.18	0.697	1.
P162.PWY..L.glutamate.degradation.V..via.hydroxyglutarate.	All Ulcerative Colitis	0.687	2.22	0.757	1.
P163.PWY..L.lysine.fermentation.to.acetate.and.butanoate	All Crohn's Disease	1.59	2.12	0.454	1.
P163.PWY..L.lysine.fermentation.to.acetate.and.butanoate	All Ulcerative Colitis	0.862	2.15	0.689	1.
P185.PWY..formaldehyde.assimilation.III..dihydroxyacetone.cycle.	All Crohn's Disease	-0.0716	2.37	0.999	1.
P185.PWY..formaldehyde.assimilation.III..dihydroxyacetone.cycle.	All Ulcerative Colitis	-0.268	2.3	0.991	1.
P221.PWY..octane.oxidation	All Crohn's Disease	2.75	2.08	0.188	1.
P221.PWY..octane.oxidation	All Ulcerative Colitis	2.44	2.07	0.239	1.
P23.PWY..reductive.TCA.cycle.I	All Crohn's Disease	2.71	2.09	0.195	1.
P23.PWY..reductive.TCA.cycle.I	All Ulcerative Colitis	2.9	2.09	0.164	1.
P4.PWY..superpathway.of.L.lysine..L.threonine.and.L.methionine.biosynthesis.I	All Crohn's Disease	1.79	1.64	0.476	1.
P4.PWY..superpathway.of.L.lysine..L.threonine.and.L.methionine.biosynthesis.I	All Ulcerative Colitis	0.268	1.24	0.185	1.
P42.PWY..incomplete.reductive.TCA.cycle	All Crohn's Disease	-1.01	2.17	0.0989	1.
P42.PWY..incomplete.reductive.TCA.cycle	All Ulcerative Colitis	-7.56×10^{-03}	2.3	0.227	1.
P441.PWY..superpathway.of.N.acetylneuraminate.degradation	All Crohn's Disease	-0.0716	2.37	0.205	1.
P441.PWY..superpathway.of.N.acetylneuraminate.degradation	All Ulcerative Colitis	-0.268	2.3	0.143	1.
P562.PWY..myo.inositol.degradation.I	All Crohn's Disease	1.	2.17	0.644	1.
P562.PWY..myo.inositol.degradation.I	All Ulcerative Colitis	2.1	2.08	0.311	1.
P621.PWY..nylon.6.oligomer.degradation	All Crohn's Disease	-0.386	1.27	0.576	1.
P621.PWY..nylon.6.oligomer.degradation	All Ulcerative Colitis	0.671	1.39	0.862	1.
POLYAMINSYN3.PWY..superpathway.of.polyamine.biosynthesis.II	All Crohn's Disease	-0.28	1.23	0.202	1.
POLYAMINSYN3.PWY..superpathway.of.polyamine.biosynthesis.II	All Ulcerative Colitis	-0.29	1.21	0.205	1.
PROPFERM.PWY..superpathway.of.L.alanine.fermentation..Stickland.reaction.	All Crohn's Disease	-0.295	1.27	0.55	1.
PROPFERM.PWY..superpathway.of.L.alanine.fermentation..Stickland.reaction.	All Ulcerative Colitis	8.01×10^{-03}	1.21	0.392	1.
PROTOCATECHUATE.ORTHO.CLEAVAGE.PWY protocatechuate.degradation.II..ortho.cleavage.pathway.	All Crohn's Disease	-0.9	1.35	0.755	1.
PROTOCATECHUATE.ORTHO.CLEAVAGE.PWY protocatechuate.degradation.II..ortho.cleavage.pathway.	All Ulcerative Colitis	0.011	1.22	0.777	1.
PRPP.PWY..superpathway.of.histidine..purine..and.pyrimidine.biosynthesis	All Crohn's Disease	1.98	2.1	0.345	1.
PRPP.PWY..superpathway.of.histidine..purine..and.pyrimidine.biosynthesis	All Ulcerative Colitis	2.18	2.08	0.293	1.
PWY.3781..aerobic.respiration.I..cytochrome.c.	All Crohn's Disease	1.66	2.12	0.433	1.

PWY.3781..aerobic.respiration.I..cytochrome.c.	All Ulcerative Colitis	3.38	2.08	0.104	1.
PWY.5004..superpathway.of.L.citrulline.metabolism	All Crohn's Disease	2.33	2.09	0.266	1.
PWY.5004..superpathway.of.L.citrulline.metabolism	All Ulcerative Colitis	0.8	2.18	0.714	1.
PWY.5005..biotin.biosynthesis.II	All Crohn's Disease	-0.0423	2.36	1.	1.
PWY.5005..biotin.biosynthesis.II	All Ulcerative Colitis	-1.9	2.09	0.42	1.
PWY.5022..4.aminobutanoate.degradation.V	All Crohn's Disease	-1.06	2.17	0.591	1.
PWY.5022..4.aminobutanoate.degradation.V	All Ulcerative Colitis	-1.91	2.09	0.591	1.
PWY.5028..L.histidine.degradation.II	All Crohn's Disease	-6.16×10^{-03}	2.36	0.998	1.
PWY.5028..L.histidine.degradation.II	All Ulcerative Colitis	2.08	2.08	0.316	1.
PWY.5104..L.isoleucine.biosynthesis.IV	All Crohn's Disease	1.71	1.23	0.298	1.
PWY.5104..L.isoleucine.biosynthesis.IV	All Ulcerative Colitis	0.738	1.18	0.245	1.
PWY.5130..2.oxobutanoate.degradation.I	All Ulcerative Colitis	-2.41	2.07	0.431	1.
PWY.5138..fatty.acid..beta..oxidation.IV..unsaturated..even.number.	All Crohn's Disease	0.95	1.2	0.675	1.
PWY.5138..fatty.acid..beta..oxidation.IV..unsaturated..even.number.	All Ulcerative Colitis	1.08	1.18	0.592	1.
PWY.5156..superpathway.of.fatty.acid.biosynthesis.II..plant.	All Crohn's Disease	5.65×10^{-03}	2.36	0.998	1.
PWY.5156..superpathway.of.fatty.acid.biosynthesis.II..plant.	All Ulcerative Colitis	1.72	2.09	0.41	1.
PWY.5180..toluene.degradation.I..aerobic...via.o.cresol.	All Crohn's Disease	0.939	2.17	0.665	1.
PWY.5180..toluene.degradation.I..aerobic...via.o.cresol.	All Ulcerative Colitis	2.2	2.08	0.29	1.
PWY.5189..tetrapyrrole.biosynthesis.II..from.glycine.	All Crohn's Disease	-1.69	2.12	0.669	1.
PWY.5189..tetrapyrrole.biosynthesis.II..from.glycine.	All Ulcerative Colitis	-0.832	2.19	0.707	1.
PWY.5265..peptidoglycan.biosynthesis.II..staphylococci.	All Crohn's Disease	0.139	1.28	0.888	1.
PWY.5265..peptidoglycan.biosynthesis.II..staphylococci.	All Ulcerative Colitis	-0.479	1.23	0.658	1.
PWY.5345..superpathway.of.L.methionine.biosynthesis..by.sulfhydrylation.	All Crohn's Disease	-0.0562	2.37	0.549	1.
PWY.5345..superpathway.of.L.methionine.biosynthesis..by.sulfhydrylation.	All Ulcerative Colitis	-0.496	2.23	0.0509	1.
PWY.5347..superpathway.of.L.methionine.biosynthesis..transsulfuration.	All Crohn's Disease	1.83	1.64	0.459	1.
PWY.5347..superpathway.of.L.methionine.biosynthesis..transsulfuration.	All Ulcerative Colitis	0.457	1.25	0.143	1.
PWY.5367..petroselinate.biosynthesis	All Crohn's Disease	-0.0699	2.37	0.703	1.
PWY.5367..petroselinate.biosynthesis	All Ulcerative Colitis	-0.867	2.19	0.417	1.
PWY.5392..reductive.TCA.cycle.II	All Crohn's Disease	2.34	2.09	0.264	1.
PWY.5392..reductive.TCA.cycle.II	All Ulcerative Colitis	2.58	2.09	0.217	1.
PWY.5415..catechol.degradation.I..meta.cleavage.pathway.	All Crohn's Disease	1.51	2.12	0.475	1.
PWY.5415..catechol.degradation.I..meta.cleavage.pathway.	All Ulcerative Colitis	2.21	2.08	0.289	1.
PWY.5464..superpathway.of.cytosolic.glycolysis..plants pyruvate.dehydrogenase.and.TCA.cycle	All Crohn's Disease	-0.302	1.24	0.0989	1.
PWY.5464..superpathway.of.cytosolic.glycolysis..plants pyruvate.dehydrogenase.and.TCA.cycle	All Ulcerative Colitis	-0.195	1.23	0.984	1.
PWY.5494..pyruvate.fermentation.to.propanoate.II..acrylate.pathway.	All Crohn's Disease	-0.295	1.27	0.563	1.
PWY.5494..pyruvate.fermentation.to.propanoate.II..acrylate.pathway.	All Ulcerative Colitis	8.01×10^{-03}	1.21	0.414	1.
PWY.5497..purine.nucleobases.degradation.II..anaerobic.	All Crohn's Disease	-7.19×10^{-03}	2.36	0.909	1.
PWY.5497..purine.nucleobases.degradation.II..anaerobic.	All Ulcerative Colitis	-1.33	2.11	0.696	1.
PWY.5505..L.glutamate.and.L.glutamine.biosynthesis	All Crohn's Disease	-0.0712	2.37	0.999	1.
PWY.5505..L.glutamate.and.L.glutamine.biosynthesis	All Ulcerative Colitis	-0.275	2.29	0.639	1.
PWY.5531..3.8.divinyl.chlorophyllide.a.biosynthesis.II..anaerobic.	All Crohn's Disease	-1.97	1.3	0.241	1.
PWY.5531..3.8.divinyl.chlorophyllide.a.biosynthesis.II..anaerobic.	All Ulcerative Colitis	-2.11	1.36	0.225	1.
PWY.561..superpathway.of.glyoxylate.cycle.and.fatty.acid.degradation	All Crohn's Disease	1.3	1.21	0.486	1.
PWY.561..superpathway.of.glyoxylate.cycle.and.fatty.acid.degradation	All Ulcerative Colitis	1.1	1.18	0.579	1.
PWY.5656..mannosylglycerate.biosynthesis.I	All Crohn's Disease	0.199	1.23	0.947	1.
PWY.5656..mannosylglycerate.biosynthesis.I	All Ulcerative Colitis	0.564	1.18	0.394	1.
PWY.5675..nitrate.reduction.V..assimilatory.	All Crohn's Disease	1.64	1.23	0.331	1.
PWY.5675..nitrate.reduction.V..assimilatory.	All Ulcerative Colitis	0.978	1.19	0.426	1.
PWY.5677..succinate.fermentation.to.butanoate	All Crohn's Disease	-1.39	1.22	0.444	1.
PWY.5677..succinate.fermentation.to.butanoate	All Ulcerative Colitis	-0.917	1.19	0.688	1.
PWY.5690..TCA.cycle.II..plants.and.fungi.	All Crohn's Disease	-1.58	2.12	0.703	1.
PWY.5690..TCA.cycle.II..plants.and.fungi.	All Ulcerative Colitis	-1.78	2.09	0.633	1.
PWY.5692..allantoin.degradation.to.glyoxylate.II	All Crohn's Disease	1.08	2.17	0.618	1.
PWY.5692..allantoin.degradation.to.glyoxylate.II	All Ulcerative Colitis	1.42	2.12	0.505	1.
PWY.5705..allantoin.degradation.to.glyoxylate.III	All Crohn's Disease	1.59	2.12	0.451	1.
PWY.5705..allantoin.degradation.to.glyoxylate.III	All Ulcerative Colitis	2.36	2.07	0.255	1.
PWY.5723..Rubisco.shunt	All Crohn's Disease	3.94	2.12	0.0628	1.
PWY.5723..Rubisco.shunt	All Ulcerative Colitis	3.24	2.07	0.118	1.
PWY.5747..2.methylcitrate.cycle.II	All Crohn's Disease	-0.229	1.26	0.718	1.
PWY.5747..2.methylcitrate.cycle.II	All Ulcerative Colitis	0.529	1.18	0.407	1.
PWY.5837..2.carboxy.1.4.naphthoquinol.biosynthesis	All Crohn's Disease	0.777	1.35	0.52	1.
PWY.5837..2.carboxy.1.4.naphthoquinol.biosynthesis	All Ulcerative Colitis	1.99	1.63	0.395	1.
PWY.5838..superpathway.of.menaquinol.8.biosynthesis.I	All Crohn's Disease	0.777	1.35	0.41	1.
PWY.5838..superpathway.of.menaquinol.8.biosynthesis.I	All Ulcerative Colitis	1.99	1.63	0.395	1.
PWY.5840..superpathway.of.menaquinol.7.biosynthesis	All Crohn's Disease	0.74	1.36	0.694	1.
PWY.5840..superpathway.of.menaquinol.7.biosynthesis	All Ulcerative Colitis	1.09	1.41	0.598	1.
PWY.5845..superpathway.of.menaquinol.9.biosynthesis	All Crohn's Disease	1.11	1.19	0.44	1.
PWY.5845..superpathway.of.menaquinol.9.biosynthesis	All Ulcerative Colitis	1.71	1.29	0.332	1.
PWY.5850..superpathway.of.menaquinol.6.biosynthesis	All Crohn's Disease	1.29	1.21	0.492	1.
PWY.5850..superpathway.of.menaquinol.6.biosynthesis	All Ulcerative Colitis	0.86	1.18	0.715	1.
PWY.5855..ubiquinol.7.biosynthesis..early.decarboxylation.	All Crohn's Disease	2.39	2.09	0.253	1.
PWY.5855..ubiquinol.7.biosynthesis..early.decarboxylation.	All Ulcerative Colitis	2.45	2.07	0.238	1.
PWY.5860..superpathway.of.demethylmenaquinol.6.biosynthesis.I	All Crohn's Disease	1.29	1.21	0.492	1.
PWY.5860..superpathway.of.demethylmenaquinol.6.biosynthesis.I	All Ulcerative Colitis	0.86	1.18	0.715	1.
PWY.5861..superpathway.of.demethylmenaquinol.8.biosynthesis.I	All Crohn's Disease	0.777	1.35	0.457	1.
PWY.5861..superpathway.of.demethylmenaquinol.8.biosynthesis.I	All Ulcerative Colitis	1.99	1.63	0.395	1.
PWY.5862..superpathway.of.demethylmenaquinol.9.biosynthesis	All Crohn's Disease	1.11	1.19	0.466	1.
PWY.5862..superpathway.of.demethylmenaquinol.9.biosynthesis	All Ulcerative Colitis	1.71	1.29	0.332	1.

PWY.5896..superpathway.of.menaquinol.10.biosynthesis	All Crohn's Disease	1.29	1.21	0.492	1.
PWY.5896..superpathway.of.menaquinol.10.biosynthesis	All Ulcerative Colitis	0.86	1.18	0.715	1.
PWY.5897..superpathway.of.menaquinol.11.biosynthesis	All Crohn's Disease	0.777	1.35	0.505	1.
PWY.5897..superpathway.of.menaquinol.11.biosynthesis	All Ulcerative Colitis	1.99	1.63	0.395	1.
PWY.5898..superpathway.of.menaquinol.12.biosynthesis	All Crohn's Disease	0.777	1.35	0.505	1.
PWY.5898..superpathway.of.menaquinol.12.biosynthesis	All Ulcerative Colitis	1.99	1.63	0.395	1.
PWY.5899..superpathway.of.menaquinol.13.biosynthesis	All Crohn's Disease	0.777	1.35	0.505	1.
PWY.5899..superpathway.of.menaquinol.13.biosynthesis	All Ulcerative Colitis	1.99	1.63	0.395	1.
PWY.5910..superpathway.of.geranylgeranyldiphosphate.biosynthesis.I..via.mevalonate.	All Crohn's Disease	-0.448	1.31	0.458	1.
PWY.5910..superpathway.of.geranylgeranyldiphosphate.biosynthesis.I..via.mevalonate.	All Ulcerative Colitis	0.428	1.41	0.0559	1.
PWY.5918..superpathway.of.heme.b.biosynthesis.from.glutamate	All Crohn's Disease	-1.08	2.17	0.855	1.
PWY.5918..superpathway.of.heme.b.biosynthesis.from.glutamate	All Ulcerative Colitis	-1.04	2.15	0.724	1.
PWY.5920..superpathway.of.heme.b.biosynthesis.from.glycine	All Crohn's Disease	0.186	1.23	0.943	1.
PWY.5920..superpathway.of.heme.b.biosynthesis.from.glycine	All Ulcerative Colitis	0.877	1.18	0.506	1.
PWY.5971..palmitate.biosynthesis..type.II.fatty.acid.synthase.	All Crohn's Disease	-0.0699	2.37	0.999	1.
PWY.5971..palmitate.biosynthesis..type.II.fatty.acid.synthase.	All Ulcerative Colitis	-0.867	2.19	0.593	1.
PWY.5972..stearate.biosynthesis.I..animals.	All Crohn's Disease	-0.33	1.27	0.958	1.
PWY.5972..stearate.biosynthesis.I..animals.	All Ulcerative Colitis	7.63×10^{-03}	1.23	0.468	1.
PWY.5994..palmitate.biosynthesis..type.I.fatty.acid.synthase.	All Crohn's Disease	-2.15	2.1	0.519	1.
PWY.5994..palmitate.biosynthesis..type.I.fatty.acid.synthase.	All Ulcerative Colitis	-3.04	2.09	0.0856	1.
PWY.6138..CMP.N.acetylneuraminate.biosynthesis.I..eukaryotes.	All Crohn's Disease	1.07	2.17	0.623	1.
PWY.6138..CMP.N.acetylneuraminate.biosynthesis.I..eukaryotes.	All Ulcerative Colitis	-0.289	2.4	0.904	1.
PWY.6143..CMP.pseudaminate.biosynthesis	All Crohn's Disease	-0.334	1.12	0.945	1.
PWY.6143..CMP.pseudaminate.biosynthesis	All Ulcerative Colitis	-2.86	1.52	0.059	1.
PWY.6168..flavin.biosynthesis.III..fungi.	All Crohn's Disease	0.724	1.14	0.773	1.
PWY.6168..flavin.biosynthesis.III..fungi.	All Ulcerative Colitis	0.751	1.11	0.747	1.
PWY.6185..4.methylcatechol.degradation..ortho.cleavage.	All Crohn's Disease	1.44	2.13	0.499	1.
PWY.6185..4.methylcatechol.degradation..ortho.cleavage.	All Ulcerative Colitis	0.664	2.22	0.765	1.
PWY.621..sucrose.degradation.III..sucrose.invertase.	All Crohn's Disease	-0.0712	2.37	0.294	1.
PWY.621..sucrose.degradation.III..sucrose.invertase.	All Ulcerative Colitis	-0.275	2.29	0.991	1.
PWY.6215..4.chlorobenzoate.degradation	All Crohn's Disease	1.04	2.17	0.631	1.
PWY.6215..4.chlorobenzoate.degradation	All Ulcerative Colitis	0.743	2.17	0.732	1.
PWY.622..starch.biosynthesis	All Crohn's Disease	-1.62	2.12	0.0409	1.
PWY.622..starch.biosynthesis	All Ulcerative Colitis	-2.29	2.07	0.468	1.
PWY.6284..superpathway.of.unsaturated.fatty.acids.biosynthesis..E..coli.	All Crohn's Disease	-0.0699	2.37	0.999	1.
PWY.6284..superpathway.of.unsaturated.fatty.acids.biosynthesis..E..coli.	All Ulcerative Colitis	-0.867	2.19	0.905	1.
PWY.6285..superpathway.of.fatty.acids.biosynthesis..E..coli.	All Crohn's Disease	0.527	1.21	0.522	1.
PWY.6285..superpathway.of.fatty.acids.biosynthesis..E..coli.	All Ulcerative Colitis	1.59	1.21	0.341	1.
PWY.6293..superpathway.of.L.cysteine.biosynthesis..fungi.	All Crohn's Disease	-2.9	2.09	0.305	1.
PWY.6293..superpathway.of.L.cysteine.biosynthesis..fungi.	All Ulcerative Colitis	-0.316	2.26	0.677	1.
PWY.6318..L.phenylalanine.degradation.IV..mammalian..via.side.chain.	All Crohn's Disease	-0.909	1.35	0.502	1.
PWY.6318..L.phenylalanine.degradation.IV..mammalian..via.side.chain.	All Ulcerative Colitis	-0.415	1.26	0.741	1.
PWY.6328..L.lysine.degradation.X	All Crohn's Disease	-0.205	2.38	0.931	1.
PWY.6328..L.lysine.degradation.X	All Ulcerative Colitis	2.22	2.11	0.292	1.
PWY.6396..superpathway.of.2.3.butanediol.biosynthesis	All Crohn's Disease	1.52	2.12	0.472	1.
PWY.6396..superpathway.of.2.3.butanediol.biosynthesis	All Ulcerative Colitis	1.84	2.09	0.378	1.
PWY.6435..4.hydroxybenzoate.biosynthesis.III..plants.	All Crohn's Disease	-1.28	1.21	0.494	1.
PWY.6435..4.hydroxybenzoate.biosynthesis.III..plants.	All Ulcerative Colitis	-2.05	1.22	0.178	1.
PWY.6470..peptidoglycan.biosynthesis.V...beta..lactam.resistance.	All Crohn's Disease	-0.0712	2.37	0.951	1.
PWY.6470..peptidoglycan.biosynthesis.V...beta..lactam.resistance.	All Ulcerative Colitis	-0.275	2.29	0.372	1.
PWY.6471..peptidoglycan.biosynthesis.IV..Enterococcus.faecium.	All Crohn's Disease	-0.0943	2.37	0.968	1.
PWY.6471..peptidoglycan.biosynthesis.IV..Enterococcus.faecium.	All Ulcerative Colitis	1.85	2.09	0.378	1.
PWY.6478..GDP.D.glycero..alpha..D.manno.heptose.biosynthesis	All Crohn's Disease	-1.17	1.17	0.534	1.
PWY.6478..GDP.D.glycero..alpha..D.manno.heptose.biosynthesis	All Ulcerative Colitis	-1.44	1.16	0.236	1.
PWY.6518..bile.acids.epimerization	All Crohn's Disease	0.9	1.21	0.704	1.
PWY.6518..bile.acids.epimerization	All Ulcerative Colitis	4.45×10^{-03}	1.21	1.	1.
PWY.6531..mannitol.cycle	All Crohn's Disease	1.75	1.26	0.304	1.
PWY.6531..mannitol.cycle	All Ulcerative Colitis	1.46	1.17	0.38	1.
PWY.6545..pyrimidine.deoxyribonucleotides.de.novo.biosynthesis.III	All Crohn's Disease	1.77	1.65	0.486	1.
PWY.6545..pyrimidine.deoxyribonucleotides.de.novo.biosynthesis.III	All Ulcerative Colitis	1.57	1.55	0.524	1.
PWY.6562..norspermidine.biosynthesis	All Crohn's Disease	2.4	2.09	0.25	1.
PWY.6562..norspermidine.biosynthesis	All Ulcerative Colitis	1.39	2.11	0.51	1.
PWY.6572..chondroitin.sulfate.degradation.I..bacterial.	All Crohn's Disease	0.579	1.21	0.561	1.
PWY.6572..chondroitin.sulfate.degradation.I..bacterial.	All Ulcerative Colitis	-0.0543	1.21	0.264	1.
PWY.6588..pyruvate.fermentation.to.acetone	All Crohn's Disease	1.78	1.66	0.488	1.
PWY.6588..pyruvate.fermentation.to.acetone	All Ulcerative Colitis	1.9	1.7	0.321	1.
PWY.6590..superpathway.of.Clostridium.acetobutylicum.acidogenic.fermentation	All Crohn's Disease	-0.999	2.17	0.874	1.
PWY.6590..superpathway.of.Clostridium.acetobutylicum.acidogenic.fermentation	All Ulcerative Colitis	-1.39	2.11	0.76	1.
PWY.6612..superpathway.of.tetrahydrofolate.biosynthesis	All Crohn's Disease	-0.0716	2.37	0.999	1.
PWY.6612..superpathway.of.tetrahydrofolate.biosynthesis	All Ulcerative Colitis	-0.268	2.3	0.991	1.
PWY.6690..cinnamate.and.3.hydroxycinnamate.degradation.to.2.hydroxypentadienoate	All Crohn's Disease	0.168	1.23	0.96	1.
PWY.6690..cinnamate.and.3.hydroxycinnamate.degradation.to.2.hydroxypentadienoate	All Ulcerative Colitis	0.332	1.19	0.896	1.
PWY.6708..ubiquinol.8.biosynthesis..early.decarboxylation.	All Crohn's Disease	0.982	2.17	0.651	1.
PWY.6708..ubiquinol.8.biosynthesis..early.decarboxylation.	All Ulcerative Colitis	1.81	2.09	0.386	1.
PWY.6749..CMP.legionaminate.biosynthesis.I	All Crohn's Disease	0.516	1.22	0.189	1.
PWY.6749..CMP.legionaminate.biosynthesis.I	All Ulcerative Colitis	1.02	1.2	0.138	1.
PWY.6803..phosphatidylcholine.acyl.editing	All Crohn's Disease	1.63	1.23	0.334	1.
PWY.6803..phosphatidylcholine.acyl.editing	All Ulcerative Colitis	1.3	1.2	0.478	1.
PWY.6807..xyloglucan.degradation.II..exoglucanase.	All Crohn's Disease	-0.292	1.12	0.782	1.
PWY.6807..xyloglucan.degradation.II..exoglucanase.	All Ulcerative Colitis	-0.0432	1.1	0.181	1.

PWY.6876..isopropanol.biosynthesis..engineered.	All Crohn's Disease	-1.83	1.27	0.275	1.
PWY.6876..isopropanol.biosynthesis..engineered.	All Ulcerative Colitis	-0.6	1.1	0.504	1.
PWY.6902..chitin.degradation.II..Vibrio.	All Crohn's Disease	-0.0712	2.37	0.658	1.
PWY.6906..chitin.derivatives.degradation	All Crohn's Disease	0.829	1.34	0.786	1.
PWY.6906..chitin.derivatives.degradation	All Ulcerative Colitis	0.042	1.21	0.811	1.
PWY.6920..6.gingerol.analog.biosynthesis..engineered.	All Crohn's Disease	-1.65	1.23	0.324	1.
PWY.6920..6.gingerol.analog.biosynthesis..engineered.	All Ulcerative Colitis	-2.52	1.28	0.0962	1.
PWY.6922..L.N.delta..acetylornithine.biosynthesis	All Crohn's Disease	3.49	2.1	0.0961	1.
PWY.6922..L.N.delta..acetylornithine.biosynthesis	All Ulcerative Colitis	2.99	2.07	0.149	1.
PWY.6953..dTDP.3.acetamido..alpha..D.fucose.biosynthesis	All Crohn's Disease	-0.305	1.27	0.152	1.
PWY.6953..dTDP.3.acetamido..alpha..D.fucose.biosynthesis	All Ulcerative Colitis	0.0108	1.22	0.183	1.
PWY.6961..L.ascorbate.degradation.II..bacterial..aerobic.	All Crohn's Disease	0.295	1.12	0.527	1.
PWY.6961..L.ascorbate.degradation.II..bacterial..aerobic.	All Ulcerative Colitis	0.64	1.12	0.0727	1.
PWY.6992..1.5.anhydrofructose.degradation	All Crohn's Disease	-0.376	1.12	0.545	1.
PWY.6992..1.5.anhydrofructose.degradation	All Ulcerative Colitis	-0.363	1.09	0.657	1.
PWY.7031..protein.N.glycosylation..bacterial.	All Crohn's Disease	0.89	2.18	0.682	1.
PWY.7031..protein.N.glycosylation..bacterial.	All Ulcerative Colitis	0.799	2.18	0.715	1.
PWY.7039..phosphatidate.metabolism..as.a.signaling.molecule	All Crohn's Disease	-2.	1.66	0.229	1.
PWY.7039..phosphatidate.metabolism..as.a.signaling.molecule	All Ulcerative Colitis	-1.08	1.4	0.44	1.
PWY.7094..fatty.acid.salvage	All Crohn's Disease	0.204	1.23	0.712	1.
PWY.7094..fatty.acid.salvage	All Ulcerative Colitis	0.55	1.18	0.358	1.
PWY.7118..chitin.deacetylation	All Crohn's Disease	2.12	1.26	0.175	1.
PWY.7118..chitin.deacetylation	All Ulcerative Colitis	1.37	1.18	0.435	1.
PWY.7159..3.8.divinyl.chlorophyllide.a.biosynthesis.III..aerobic..light.independent.	All Crohn's Disease	-0.0789	2.37	0.973	1.
PWY.7159..3.8.divinyl.chlorophyllide.a.biosynthesis.III..aerobic..light.independent.	All Ulcerative Colitis	0.891	2.16	0.679	1.
PWY.7184..pyrimidine.deoxyribonucleotides.de.novo.biosynthesis.I	All Crohn's Disease	1.77	1.65	0.486	1.
PWY.7184..pyrimidine.deoxyribonucleotides.de.novo.biosynthesis.I	All Ulcerative Colitis	1.57	1.55	0.524	1.
PWY.7187..pyrimidine.deoxyribonucleotides.de.novo.biosynthesis.II	All Crohn's Disease	1.98	2.1	0.345	1.
PWY.7187..pyrimidine.deoxyribonucleotides.de.novo.biosynthesis.II	All Ulcerative Colitis	2.18	2.08	0.293	1.
PWY.7196..superpathway.of.pyrimidine.ribonucleosides.salvage	All Crohn's Disease	-0.972	1.21	0.108	1.
PWY.7196..superpathway.of.pyrimidine.ribonucleosides.salvage	All Ulcerative Colitis	-1.3	1.18	0.469	1.
PWY.7200..superpathway.of.pyrimidine.deoxyribonucleoside.salvage	All Crohn's Disease	1.58	2.12	0.455	1.
PWY.7200..superpathway.of.pyrimidine.deoxyribonucleoside.salvage	All Ulcerative Colitis	1.77	2.09	0.396	1.
PWY.7204..pyridoxal.5..phosphate.salvage.II..plants.	All Crohn's Disease	0.895	1.21	0.193	1.
PWY.7209..superpathway.of.pyrimidine.ribonucleosides.degradation	All Crohn's Disease	-0.68	1.22	0.597	1.
PWY.7209..superpathway.of.pyrimidine.ribonucleosides.degradation	All Ulcerative Colitis	0.466	1.28	0.43	1.
PWY.7210..pyrimidine.deoxyribonucleotides.biosynthesis.from.CTP	All Crohn's Disease	0.8	1.48	0.83	1.
PWY.7210..pyrimidine.deoxyribonucleotides.biosynthesis.from.CTP	All Ulcerative Colitis	2.36	1.93	0.394	1.
PWY.7211..superpathway.of.pyrimidine.deoxyribonucleotides.de.novo.biosynthesis	All Crohn's Disease	1.79	1.67	0.487	1.
PWY.7211..superpathway.of.pyrimidine.deoxyribonucleotides.de.novo.biosynthesis	All Ulcerative Colitis	1.58	1.61	0.548	1.
PWY.7237..myo...chiro..and.scyllo.inositol.degradation	All Crohn's Disease	-0.0712	2.37	0.44	1.
PWY.7237..myo...chiro..and.scyllo.inositol.degradation	All Ulcerative Colitis	-0.275	2.29	0.153	1.
PWY.7254..TCA.cycle.VII..acetate.producers.	All Crohn's Disease	2.7	2.09	0.195	1.
PWY.7254..TCA.cycle.VII..acetate.producers.	All Ulcerative Colitis	3.24	2.09	0.121	1.
PWY.7268..cytosolic.NADPH.production..yeast.	All Crohn's Disease	1.1	2.17	0.614	1.
PWY.7268..cytosolic.NADPH.production..yeast.	All Ulcerative Colitis	0.311	2.27	0.891	1.
PWY.7269..mitochondrial.NADPH.production..yeast.	All Crohn's Disease	1.99	2.1	0.343	1.
PWY.7269..mitochondrial.NADPH.production..yeast.	All Ulcerative Colitis	2.18	2.08	0.295	1.
PWY.7279..aerobic.respiration.II..cytochrome.c...yeast.	All Crohn's Disease	0.934	2.17	0.667	1.
PWY.7279..aerobic.respiration.II..cytochrome.c...yeast.	All Ulcerative Colitis	1.85	2.09	0.378	1.
PWY.7294..D.xylose.degradation.IV	All Crohn's Disease	1.58	2.12	0.455	1.
PWY.7294..D.xylose.degradation.IV	All Ulcerative Colitis	0.87	2.15	0.686	1.
PWY.7312..dTDP..beta..D.fucofuranose.biosynthesis	All Crohn's Disease	0.784	1.26	0.584	1.
PWY.7312..dTDP..beta..D.fucofuranose.biosynthesis	All Ulcerative Colitis	-0.266	1.35	0.722	1.
PWY.7316..dTDP.N.acetylviosamine.biosynthesis	All Crohn's Disease	1.35	1.21	0.459	1.
PWY.7316..dTDP.N.acetylviosamine.biosynthesis	All Ulcerative Colitis	-0.652	1.29	0.663	1.
PWY.7340..9 cis..11.trans.octadecadienoyl.CoA.degradation..isomerase.dependent..yeast.	All Crohn's Disease	0.543	1.21	0.881	1.
PWY.7340..9 cis..11.trans.octadecadienoyl.CoA.degradation..isomerase.dependent..yeast.	All Ulcerative Colitis	0.654	1.19	0.592	1.
PWY.7345..superpathway.of.anaerobic.sucrose.degradation	All Crohn's Disease	-0.0868	2.38	0.406	1.
PWY.7345..superpathway.of.anaerobic.sucrose.degradation	All Ulcerative Colitis	-0.278	2.33	0.0426	1.
PWY.7356..thiamine.diphosphate.salvage.IV..yeast.	All Crohn's Disease	-0.0712	2.37	0.585	1.
PWY.7356..thiamine.diphosphate.salvage.IV..yeast.	All Ulcerative Colitis	-0.275	2.29	0.991	1.
PWY.7371..1.4.dihydroxy.6.naphthoate.biosynthesis.II	All Crohn's Disease	-1.38	1.22	0.232	1.
PWY.7371..1.4.dihydroxy.6.naphthoate.biosynthesis.II	All Ulcerative Colitis	-2.85	1.33	0.0627	1.
PWY.7383..anaerobic.energy.metabolism..invertebrates..cytosol.	All Crohn's Disease	-0.0863	2.38	0.316	1.
PWY.7385..1.3.propanediol.biosynthesis..engineered.	All Crohn's Disease	-0.846	1.34	0.204	1.
PWY.7385..1.3.propanediol.biosynthesis..engineered.	All Ulcerative Colitis	0.316	1.19	0.103	1.
PWY.7388..octanoyl..acyl.carrier.protein..biosynthesis..mitochondria..yeast.	All Crohn's Disease	1.48	2.12	0.485	1.
PWY.7388..octanoyl..acyl.carrier.protein..biosynthesis..mitochondria..yeast.	All Ulcerative Colitis	2.94	2.09	0.16	1.
PWY.7391..isoprene.biosynthesis.II..engineered.	All Crohn's Disease	0.903	2.17	0.678	1.
PWY.7391..isoprene.biosynthesis.II..engineered.	All Ulcerative Colitis	2.22	2.09	0.287	1.
PWY.7399..methylphosphonate.degradation.II	All Crohn's Disease	-0.87	1.35	0.519	1.
PWY.7399..methylphosphonate.degradation.II	All Ulcerative Colitis	-0.926	1.32	0.482	1.
PWY.7409..phospholipid.remodeling..phosphatidylethanolamine..yeast.	All Crohn's Disease	1.04	2.17	0.632	1.
PWY.7409..phospholipid.remodeling..phosphatidylethanolamine..yeast.	All Ulcerative Colitis	1.99	2.08	0.339	1.
PWY.7434..terminal.O.glycans.residues.modification..via.type.2.precursor.disaccharide.	All Crohn's Disease	-2.87	2.09	0.31	1.
PWY.7434..terminal.O.glycans.residues.modification..via.type.2.precursor.disaccharide.	All Ulcerative Colitis	-2.74	2.08	0.34	1.
PWY.7446..sulfoquinovose.degradation.I	All Crohn's Disease	-0.15	2.37	0.949	1.

PWY.7446..sulfoquinovose.degradation.I	All Ulcerative Colitis	1.82	2.11	0.388	1.
PWY.7616..methanol.oxidation.to.carbon.dioxide	All Crohn's Disease	-0.278	1.26	0.247	1.
PWY.7688 dTDP..alpha..D.ravidosamine.and.dTDP.4.acetyl..alpha..D.ravidosamine.biosynthesis	All Crohn's Disease	0.927	1.21	0.652	1.
PWY.7688 dTDP..alpha..D.ravidosamine.and.dTDP.4.acetyl..alpha..D.ravidosamine.biosynthesis	All Ulcerative Colitis	0.592	1.18	0.504	1.
PWY.7723..bacterial.bioluminescence	All Crohn's Disease	-0.879	1.35	0.516	1.
PWY.7723..bacterial.bioluminescence	All Ulcerative Colitis	-1.79	1.54	0.245	1.
PWY.7754..bile.acid.7.alpha..dehydroxylation	All Crohn's Disease	-1.3	1.19	0.472	1.
PWY.7754..bile.acid.7.alpha..dehydroxylation	All Ulcerative Colitis	-0.959	1.14	0.486	1.
PWY.7783..plasmalogen.degradation	All Crohn's Disease	1.62	2.12	0.444	1.
PWY.7783..plasmalogen.degradation	All Ulcerative Colitis	1.65	2.1	0.432	1.
PWY.7805...aminomethyl.phosphonate.degradation	All Crohn's Disease	0.999	2.17	0.645	1.
PWY.7805...aminomethyl.phosphonate.degradation	All Ulcerative Colitis	2.11	2.08	0.31	1.
PWY.7807..glyphosate.degradation.III	All Crohn's Disease	0.999	2.17	0.645	1.
PWY.7807..glyphosate.degradation.III	All Ulcerative Colitis	2.11	2.08	0.31	1.
PWY.7820..teichuronic.acid.biosynthesis..B..subtilis.168.	All Crohn's Disease	-0.0438	2.36	0.985	1.
PWY.7820..teichuronic.acid.biosynthesis..B..subtilis.168.	All Ulcerative Colitis	1.42	2.11	0.499	1.
PWY.7858...5Z..dodecenoate.biosynthesis.II	All Crohn's Disease	0.579	1.21	0.556	1.
PWY.7858...5Z..dodecenoate.biosynthesis.II	All Ulcerative Colitis	1.12	1.18	0.344	1.
PWY.7873..D.erythronate.degradation.II	All Crohn's Disease	0.982	2.17	0.651	1.
PWY.7873..D.erythronate.degradation.II	All Ulcerative Colitis	1.81	2.09	0.386	1.
PWY.7874..L.threonate.degradation	All Crohn's Disease	-0.797	1.34	0.178	1.
PWY.7874..L.threonate.degradation	All Ulcerative Colitis	0.198	1.2	0.137	1.
PWY.7883..anhydromuropeptides.recycling.II	All Crohn's Disease	1.32	1.21	0.475	1.
PWY.7883..anhydromuropeptides.recycling.II	All Ulcerative Colitis	1.62	1.19	0.318	1.
PWY.7942..5.oxo.L.proline.metabolism	All Crohn's Disease	1.67	1.23	0.317	1.
PWY.7942..5.oxo.L.proline.metabolism	All Ulcerative Colitis	0.586	1.18	0.0805	1.
PWY.7992..superpathway.of.menaquinol.8.biosynthesis.III	All Crohn's Disease	-1.3	1.21	0.222	1.
PWY.801..homocysteine.and.cysteine.interconversion	All Crohn's Disease	-2.9	2.09	0.305	1.
PWY.801..homocysteine.and.cysteine.interconversion	All Ulcerative Colitis	-0.316	2.26	0.679	1.
PWY.8073..lipid.IVA.biosynthesis..P..putida.	All Crohn's Disease	0.0654	2.37	0.842	1.
PWY.8073..lipid.IVA.biosynthesis..P..putida.	All Ulcerative Colitis	-0.901	2.15	0.895	1.
PWY.8086...S..lactate.fermentation.to.propanoate..acetate.and.hydrogen	All Crohn's Disease	-2.	1.66	0.229	1.
PWY.8086...S..lactate.fermentation.to.propanoate..acetate.and.hydrogen	All Ulcerative Colitis	-1.08	1.4	0.44	1.
PWY.8134..bile.acid.7.beta..dehydroxylation	All Crohn's Disease	-0.848	1.15	0.708	1.
PWY.8134..bile.acid.7.beta..dehydroxylation	All Ulcerative Colitis	-0.957	1.14	0.56	1.
PWY.8188..L.alanine.degradation.VI..reductive.Stickland.reaction.	All Crohn's Disease	-0.295	1.27	0.55	1.
PWY.8188..L.alanine.degradation.VI..reductive.Stickland.reaction.	All Ulcerative Colitis	8.01×10^{-03}	1.21	0.392	1.
PWY.8189..L.alanine.degradation.V..oxidative.Stickland.reaction.	All Crohn's Disease	-0.295	1.27	0.55	1.
PWY.8189..L.alanine.degradation.V..oxidative.Stickland.reaction.	All Ulcerative Colitis	8.01×10^{-03}	1.21	0.392	1.
PWY.8190..L.glutamate.degradation.XI..reductive.Stickland.reaction.	All Crohn's Disease	1.54	2.12	0.468	1.
PWY.8190..L.glutamate.degradation.XI..reductive.Stickland.reaction.	All Ulcerative Colitis	2.18	2.08	0.294	1.
PWY.821..superpathway.of.sulfur.amino.acid.biosynthesis..Saccharomyces.cerevisiae.	All Crohn's Disease	2.67	1.35	0.0941	1.
PWY.821..superpathway.of.sulfur.amino.acid.biosynthesis..Saccharomyces.cerevisiae.	All Ulcerative Colitis	1.75	1.24	0.293	1.
PWY.822..fructan.biosynthesis	All Crohn's Disease	-2.13	2.1	0.526	1.
PWY.822..fructan.biosynthesis	All Ulcerative Colitis	-0.387	2.24	0.981	1.
PWY.922..mevalonate.pathway.I..eukaryotes.and.bacteria.	All Crohn's Disease	-0.448	1.31	0.545	1.
PWY.922..mevalonate.pathway.I..eukaryotes.and.bacteria.	All Ulcerative Colitis	0.428	1.41	0.0456	1.
PWY0.1221..putrescine.degradation.II	All Crohn's Disease	-0.179	2.38	0.94	1.
PWY0.1221..putrescine.degradation.II	All Ulcerative Colitis	1.31	2.15	0.543	1.
PWY0.1241..ADP.L.glycero..beta..D.manno.heptose.biosynthesis	All Crohn's Disease	0.821	1.34	0.685	1.
PWY0.1241..ADP.L.glycero..beta..D.manno.heptose.biosynthesis	All Ulcerative Colitis	0.958	1.32	0.716	1.
PWY0.1261..anhydromuropeptides.recycling.I	All Crohn's Disease	-0.0716	2.37	0.707	1.
PWY0.1261..anhydromuropeptides.recycling.I	All Ulcerative Colitis	-0.268	2.3	0.88	1.
PWY0.1277..3.phenylpropanoate.and.3..3.hydroxyphenyl.propanoate.degradation	All Crohn's Disease	0.168	1.23	0.972	1.
PWY0.1277..3.phenylpropanoate.and.3..3.hydroxyphenyl.propanoate.degradation	All Ulcerative Colitis	0.332	1.19	0.84	1.
PWY0.1337..oleate..beta..oxidation	All Crohn's Disease	-0.228	1.26	0.358	1.
PWY0.1337..oleate..beta..oxidation	All Ulcerative Colitis	0.525	1.18	0.258	1.
PWY0.1338..polymyxin.resistance	All Crohn's Disease	0.176	1.23	0.422	1.
PWY0.1338..polymyxin.resistance	All Ulcerative Colitis	0.903	1.18	0.434	1.
PWY0.1415..superpathway.of.heme.b.biosynthesis.from.uroporphyrinogen.III	All Crohn's Disease	-1.33	1.21	0.248	1.
PWY0.1415..superpathway.of.heme.b.biosynthesis.from.uroporphyrinogen.III	All Ulcerative Colitis	-0.74	1.18	0.0693	1.
PWY0.1533..methylphosphonate.degradation.I	All Crohn's Disease	0.999	2.17	0.645	1.
PWY0.1533..methylphosphonate.degradation.I	All Ulcerative Colitis	2.11	2.08	0.31	1.
PWY0.166 superpathway.of.pyrimidine.deoxyribonucleotides.de.novo.biosynthesis..E..coli.	All Crohn's Disease	0.592	1.21	0.859	1.
PWY0.166 superpathway.of.pyrimidine.deoxyribonucleotides.de.novo.biosynthesis..E..coli.	All Ulcerative Colitis	1.36	1.18	0.439	1.
PWY0.301..L.ascorbate.degradation.I..bacterial..anaerobic.	All Crohn's Disease	0.295	1.12	0.377	1.
PWY0.301..L.ascorbate.degradation.I..bacterial..anaerobic.	All Ulcerative Colitis	0.64	1.12	0.043	1.
PWY0.41..allantoin.degradation.IV..anaerobic.	All Crohn's Disease	1.02	2.17	0.637	1.
PWY0.41..allantoin.degradation.IV..anaerobic.	All Ulcerative Colitis	2.34	2.07	0.259	1.
PWY0.42..2.methylcitrate.cycle.I	All Crohn's Disease	0.635	1.21	0.84	1.
PWY0.42..2.methylcitrate.cycle.I	All Ulcerative Colitis	0.688	1.19	0.538	1.
PWY0.461..L.lysine.degradation.I	All Crohn's Disease	-0.889	1.35	0.513	1.
PWY0.461..L.lysine.degradation.I	All Ulcerative Colitis	0.0107	1.22	0.6	1.
PWY0.781..aspartate.superpathway	All Crohn's Disease	1.79	1.64	0.476	1.
PWY0.781..aspartate.superpathway	All Ulcerative Colitis	0.268	1.24	0.185	1.
PWY1G.0..mycothiol.biosynthesis	All Crohn's Disease	-0.887	1.35	0.605	1.

PWY1G.0..mycothiol.biosynthesis	All Ulcerative Colitis	-0.402	1.25	0.429	1.
PWY1ZNC.1..assimilatory.sulfate.reduction.IV	All Crohn's Disease	-0.0712	2.37	0.999	1.
PWY1ZNC.1..assimilatory.sulfate.reduction.IV	All Ulcerative Colitis	-0.275	2.29	0.991	1.
PWY490.3..nitrate.reduction.VI..assimilatory.	All Crohn's Disease	1.53	2.12	0.471	1.
PWY490.3..nitrate.reduction.VI..assimilatory.	All Ulcerative Colitis	0.0282	2.3	0.99	1.
PWY66.367..ketogenesis	All Crohn's Disease	-0.831	1.34	0.785	1.
PWY66.367..ketogenesis	All Ulcerative Colitis	-0.039	1.21	0.999	1.
PWY66.388..ceramide.degradation.by..alpha..oxidation	All Crohn's Disease	1.7	2.13	0.424	1.
PWY66.388..ceramide.degradation.by..alpha..oxidation	All Ulcerative Colitis	0.804	2.19	0.714	1.
PWY66.389..phytol.degradation	All Crohn's Disease	1.63	1.23	0.201	1.
PWY66.389..phytol.degradation	All Ulcerative Colitis	1.65	1.22	0.115	1.
PWY66.391..fatty.acid..beta..oxidation.VI..mammalian.peroxisome.	All Crohn's Disease	-1.65	2.12	0.683	1.
PWY66.391..fatty.acid..beta..oxidation.VI..mammalian.peroxisome.	All Ulcerative Colitis	-1.88	2.09	0.601	1.
PWY66.399..gluconeogenesis.III	All Crohn's Disease	-0.0863	2.38	0.394	1.
PWY66.430..myristate.biosynthesis..mitochondria.	All Crohn's Disease	1.48	2.12	0.485	1.
PWY66.430..myristate.biosynthesis..mitochondria.	All Ulcerative Colitis	2.94	2.09	0.16	1.
REDCITCYC..TCA.cycle.VI..Helicobacter.	All Crohn's Disease	3.47	2.1	0.0977	1.
REDCITCYC..TCA.cycle.VI..Helicobacter.	All Ulcerative Colitis	3.7	2.1	0.0776	1.
RUMP.PWY..formaldehyde.oxidation.I	All Crohn's Disease	-1.01	2.17	0.806	1.
RUMP.PWY..formaldehyde.oxidation.I	All Ulcerative Colitis	-1.36	2.11	0.729	1.
SO4ASSIM.PWY..assimilatory.sulfate.reduction.I	All Crohn's Disease	-0.0562	2.37	0.536	1.
SO4ASSIM.PWY..assimilatory.sulfate.reduction.I	All Ulcerative Colitis	-0.496	2.23	0.0443	1.
SULFATE.CYS.PWY..superpathway.of.sulfate.assimilation.and.cysteine.biosynthesis	All Crohn's Disease	-0.0562	2.37	0.551	1.
SULFATE.CYS.PWY..superpathway.of.sulfate.assimilation.and.cysteine.biosynthesis	All Ulcerative Colitis	-0.496	2.23	0.0526	1.
TCA..TCA.cycle.I..prokaryotic.	All Crohn's Disease	1.26	1.21	0.505	1.
TCA..TCA.cycle.I..prokaryotic.	All Ulcerative Colitis	1.82	1.22	0.25	1.
TCA.GLYOX.BYPASS..superpathway.of.glyoxylate.bypass.and.TCA	All Crohn's Disease	1.3	1.21	0.486	1.
TCA.GLYOX.BYPASS..superpathway.of.glyoxylate.bypass.and.TCA	All Ulcerative Colitis	1.1	1.18	0.579	1.
THREOCAT.PWY..superpathway.of.L.threonine.metabolism	All Crohn's Disease	0.807	2.19	0.712	1.
THREOCAT.PWY..superpathway.of.L.threonine.metabolism	All Ulcerative Colitis	1.2	2.18	0.582	1.
UBISYN.PWY..superpathway.of.ubiquinol.8.biosynthesis..early.decarboxylation.	All Crohn's Disease	0.982	2.17	0.651	1.
UBISYN.PWY..superpathway.of.ubiquinol.8.biosynthesis..early.decarboxylation.	All Ulcerative Colitis	1.81	2.09	0.386	1.
UDPNACETYLGALSYN.PWY..UDP.N.acetyl.D.glucosamine.biosynthesis.II	All Crohn's Disease	2.42	2.09	0.247	1.
UDPNACETYLGALSYN.PWY..UDP.N.acetyl.D.glucosamine.biosynthesis.II	All Ulcerative Colitis	0.84	2.15	0.697	1.
URDEGR.PWY..superpathway.of.allantoin.degradation.in.plants	All Crohn's Disease	1.08	2.17	0.618	1.
URDEGR.PWY..superpathway.of.allantoin.degradation.in.plants	All Ulcerative Colitis	1.42	2.12	0.505	1.
URSIN.PWY..ureide.biosynthesis	All Crohn's Disease	1.04	2.17	0.631	1.
URSIN.PWY..ureide.biosynthesis	All Ulcerative Colitis	0.743	2.17	0.732	1.
X3.HYDROXYPHENYLACETATE.DEGRADATION.PWY 4.hydroxyphenylacetate.degradation	All Crohn's Disease	-0.953	1.36	0.483	1.
X3.HYDROXYPHENYLACETATE.DEGRADATION.PWY 4.hydroxyphenylacetate.degradation	All Ulcerative Colitis	-0.467	1.28	0.716	1.
PWY4LZ.257..superpathway.of.fermentation..Chlamydomonas.reinhardtii.	All Ulcerative Colitis	NA	NA	2.55×10^{-04}	0.224
P161.PWY..acetylene.degradation..anaerobic.	All Ulcerative Colitis	NA	NA	8.38×10^{-04}	0.306
PWY.702..L.methionine.biosynthesis.II	All Crohn's Disease	NA	NA	5.96×10^{-03}	0.367
PWY.I9..L.cysteine.biosynthesis.VI..from.L.methionine.	All Crohn's Disease	NA	NA	8.95×10^{-03}	0.415
P161.PWY..acetylene.degradation..anaerobic.	All Crohn's Disease	NA	NA	0.0139	0.626
PWY4LZ.257..superpathway.of.fermentation..Chlamydomonas.reinhardtii.	All Crohn's Disease	NA	NA	0.0205	0.767
PWY0.1477..ethanolamine.utilization	All Ulcerative Colitis	NA	NA	0.0244	0.857
PWY0.1298..superpathway.of.pyrimidine.deoxyribonucleosides.degradation	All Ulcerative Colitis	NA	NA	0.0261	0.875
PWY.5136..fatty.acid..beta..oxidation.II..plant.peroxisome.	All Ulcerative Colitis	NA	NA	0.0357	0.948
ANAEROFRUCAT.PWY..homolactic.fermentation	All Crohn's Disease	NA	NA	0.0599	1.
ANAEROFRUCAT.PWY..homolactic.fermentation	All Ulcerative Colitis	NA	NA	0.427	1.
ANAGLYCOLYSIS.PWY..glycolysis.III..from.glucose.	All Crohn's Disease	NA	NA	1.	1.
ANAGLYCOLYSIS.PWY..glycolysis.III..from.glucose.	All Ulcerative Colitis	NA	NA	0.127	1.
ARG.POLYAMINE.SYN..superpathway.of.arginine.and.polyamine.biosynthesis	All Crohn's Disease	NA	NA	0.151	1.
ARG.POLYAMINE.SYN..superpathway.of.arginine.and.polyamine.biosynthesis	All Ulcerative Colitis	NA	NA	0.139	1.
ARGSYN.PWY..L.arginine.biosynthesis.I..via.L.ornithine.	All Crohn's Disease	NA	NA	1.	1.
ARGSYN.PWY..L.arginine.biosynthesis.I..via.L.ornithine.	All Ulcerative Colitis	NA	NA	0.126	1.
ARGSYNBSUB.PWY..L.arginine.biosynthesis.II..acetyl.cycle.	All Crohn's Disease	NA	NA	1.	1.
ARGSYNBSUB.PWY..L.arginine.biosynthesis.II..acetyl.cycle.	All Ulcerative Colitis	NA	NA	0.141	1.
ARO.PWY..chorismate.biosynthesis.I	All Crohn's Disease	NA	NA	1.	1.
ARO.PWY..chorismate.biosynthesis.I	All Ulcerative Colitis	NA	NA	0.0794	1.
ASPASN.PWY..superpathway.of.L.aspartate.and.L.asparagine.biosynthesis	All Crohn's Disease	NA	NA	1.	1.
ASPASN.PWY..superpathway.of.L.aspartate.and.L.asparagine.biosynthesis	All Ulcerative Colitis	NA	NA	1.	1.
BIOTIN.BIOSYNTHESIS.PWY..biotin.biosynthesis.I	All Crohn's Disease	NA	NA	1.	1.
BIOTIN.BIOSYNTHESIS.PWY..biotin.biosynthesis.I	All Ulcerative Colitis	NA	NA	0.507	1.
BRANCHED.CHAIN.AA.SYN.PWY superpathway.of.branched.chain.amino.acid.biosynthesis	All Crohn's Disease	NA	NA	1.	1.
BRANCHED.CHAIN.AA.SYN.PWY superpathway.of.branched.chain.amino.acid.biosynthesis	All Ulcerative Colitis	NA	NA	0.121	1.
CALVIN.PWY..Calvin.Benson.Bassham.cycle	All Crohn's Disease	NA	NA	1.	1.
CALVIN.PWY..Calvin.Benson.Bassham.cycle	All Ulcerative Colitis	NA	NA	1.	1.
CITRULBIO.PWY..L.citrulline.biosynthesis	All Crohn's Disease	NA	NA	0.388	1.
CITRULBIO.PWY..L.citrulline.biosynthesis	All Ulcerative Colitis	NA	NA	1.	1.
COA.PWY..coenzyme.A.biosynthesis.I..prokaryotic.	All Crohn's Disease	NA	NA	1.	1.
COA.PWY..coenzyme.A.biosynthesis.I..prokaryotic.	All Ulcerative Colitis	NA	NA	0.113	1.
COA.PWY.1..superpathway.of.coenzyme.A.biosynthesis.III..mammals.	All Crohn's Disease	NA	NA	1.	1.
COA.PWY.1..superpathway.of.coenzyme.A.biosynthesis.III..mammals.	All Ulcerative Colitis	NA	NA	0.12	1.
COBALSYN.PWY..superpathway.of.adenosylcobalamin.salvage.from.cobinamide.I	All Crohn's Disease	NA	NA	0.275	1.

COBALSYN.PWY..superpathway.of.adenosylcobalamin.salvage.from.cobinamide.I	All Ulcerative Colitis	NA	NA	0.178	1.
COLANSYN.PWY..colanic.acid.building.blocks.biosynthesis	All Crohn's Disease	NA	NA	1.	1.
COLANSYN.PWY..colanic.acid.building.blocks.biosynthesis	All Ulcerative Colitis	NA	NA	1.	1.
COMPLETE.ARO.PWY..superpathway.of.aromatic.amino.acid.biosynthesis	All Crohn's Disease	NA	NA	1.	1.
COMPLETE.ARO.PWY..superpathway.of.aromatic.amino.acid.biosynthesis	All Ulcerative Colitis	NA	NA	0.0786	1.
DAPLYSINESYN.PWY..L.lysine.biosynthesis.I	All Crohn's Disease	NA	NA	0.129	1.
DAPLYSINESYN.PWY..L.lysine.biosynthesis.I	All Ulcerative Colitis	NA	NA	1.	1.
DTDPRHAMSYN.PWY..dTDP..beta..L.rhamnose.biosynthesis	All Crohn's Disease	NA	NA	1.	1.
DTDPRHAMSYN.PWY..dTDP..beta..L.rhamnose.biosynthesis	All Ulcerative Colitis	NA	NA	0.0892	1.
FASYN.ELONG.PWY..fatty.acid.elongation....saturated	All Crohn's Disease	NA	NA	1.	1.
FASYN.ELONG.PWY..fatty.acid.elongation....saturated	All Ulcerative Colitis	NA	NA	0.214	1.
FERMENTATION.PWY..mixed.acid.fermentation	All Crohn's Disease	NA	NA	1.	1.
FERMENTATION.PWY..mixed.acid.fermentation	All Ulcerative Colitis	NA	NA	0.453	1.
FUC.RHAMCAT.PWY..superpathway.of.fucose.and.rhamnose.degradation	All Crohn's Disease	NA	NA	1.	1.
FUC.RHAMCAT.PWY..superpathway.of.fucose.and.rhamnose.degradation	All Ulcerative Colitis	NA	NA	0.759	1.
FUCCAT.PWY..fucose.degradation	All Crohn's Disease	NA	NA	1.	1.
FUCCAT.PWY..fucose.degradation	All Ulcerative Colitis	NA	NA	1.	1.
GALACTUROCAT.PWY..D.galacturonate.degradation.I	All Crohn's Disease	NA	NA	0.213	1.
GALACTUROCAT.PWY..D.galacturonate.degradation.I	All Ulcerative Colitis	NA	NA	1.	1.
GLCMANNANAUT.PWY..superpathway.of.N.acetylglucosamine N.acetylmannosamine.and.N.acetylneuraminate.degradation	All Crohn's Disease	NA	NA	1.	1.
GLCMANNANAUT.PWY..superpathway.of.N.acetylglucosamine N.acetylmannosamine.and.N.acetylneuraminate.degradation	All Ulcerative Colitis	NA	NA	1.	1.
GLUCONEO.PWY..gluconeogenesis.I	All Crohn's Disease	NA	NA	1.	1.
GLUCONEO.PWY..gluconeogenesis.I	All Ulcerative Colitis	NA	NA	0.191	1.
GLUCUROCAT.PWY..superpathway.of..beta..D.glucuronosides.degradation	All Crohn's Disease	NA	NA	0.471	1.
GLUCUROCAT.PWY..superpathway.of..beta..D.glucuronosides.degradation	All Ulcerative Colitis	NA	NA	1.	1.
GLUTORN.PWY..L.ornithine.biosynthesis.I	All Crohn's Disease	NA	NA	1.	1.
GLUTORN.PWY..L.ornithine.biosynthesis.I	All Ulcerative Colitis	NA	NA	0.126	1.
GLYCOGENSYNTH.PWY..glycogen.biosynthesis.I..from.ADP.D.Glucose.	All Crohn's Disease	NA	NA	1.	1.
GLYCOGENSYNTH.PWY..glycogen.biosynthesis.I..from.ADP.D.Glucose.	All Ulcerative Colitis	NA	NA	0.195	1.
GLYCOLYSIS..glycolysis.I..from.glucose.6.phosphate.	All Crohn's Disease	NA	NA	0.078	1.
GLYCOLYSIS..glycolysis.I..from.glucose.6.phosphate.	All Ulcerative Colitis	NA	NA	1.	1.
GLYCOLYSIS.E.D..superpathway.of.glycolysis.and.the.Entner.Doudoroff.pathway	All Crohn's Disease	NA	NA	0.203	1.
GLYCOLYSIS.E.D..superpathway.of.glycolysis.and.the.Entner.Doudoroff.pathway	All Ulcerative Colitis	NA	NA	0.121	1.
HEMESYN2.PWY..heme.b.biosynthesis.II..oxygen.independent.	All Crohn's Disease	NA	NA	0.166	1.
HEMESYN2.PWY..heme.b.biosynthesis.II..oxygen.independent.	All Ulcerative Colitis	NA	NA	0.058	1.
HISDEG.PWY..L.histidine.degradation.I	All Crohn's Disease	NA	NA	1.	1.
HISDEG.PWY..L.histidine.degradation.I	All Ulcerative Colitis	NA	NA	0.423	1.
HISTSYN.PWY..L.histidine.biosynthesis	All Crohn's Disease	NA	NA	1.	1.
HISTSYN.PWY..L.histidine.biosynthesis	All Ulcerative Colitis	NA	NA	0.0924	1.
HSERMETANA.PWY..L.methionine.biosynthesis.III	All Crohn's Disease	NA	NA	0.126	1.
HSERMETANA.PWY..L.methionine.biosynthesis.III	All Ulcerative Colitis	NA	NA	0.138	1.
ILEUSYN.PWY..L.isoleucine.biosynthesis.I..from.threonine.	All Crohn's Disease	NA	NA	0.38	1.
ILEUSYN.PWY..L.isoleucine.biosynthesis.I..from.threonine.	All Ulcerative Colitis	NA	NA	0.123	1.
LACTOSECAT.PWY..lactose.and.galactose.degradation.I	All Crohn's Disease	NA	NA	1.	1.
LACTOSECAT.PWY..lactose.and.galactose.degradation.I	All Ulcerative Colitis	NA	NA	0.523	1.
NONMEVIPP.PWY..methylerythritol.phosphate.pathway.I	All Crohn's Disease	NA	NA	1.	1.
NONMEVIPP.PWY..methylerythritol.phosphate.pathway.I	All Ulcerative Colitis	NA	NA	0.124	1.
NONOXIPENT.PWY..pentose.phosphate.pathway..non.oxidative.branch..I	All Crohn's Disease	NA	NA	1.	1.
NONOXIPENT.PWY..pentose.phosphate.pathway..non.oxidative.branch..I	All Ulcerative Colitis	NA	NA	1.	1.
OANTIGEN.PWY..O.antigen.building.blocks.biosynthesis..E..coli.	All Crohn's Disease	NA	NA	0.333	1.
OANTIGEN.PWY..O.antigen.building.blocks.biosynthesis..E..coli.	All Ulcerative Colitis	NA	NA	1.	1.
P164.PWY..purine.nucleobases.degradation.I..anaerobic.	All Crohn's Disease	NA	NA	1.	1.
P164.PWY..purine.nucleobases.degradation.I..anaerobic.	All Ulcerative Colitis	NA	NA	1.	1.
P41.PWY..pyruvate.fermentation.to.acetate.and..S..lactate.I	All Crohn's Disease	NA	NA	0.196	1.
P41.PWY..pyruvate.fermentation.to.acetate.and..S..lactate.I	All Ulcerative Colitis	NA	NA	0.246	1.
P461.PWY..hexitol.fermentation.to.lactate..formate..ethanol.and.acetate	All Crohn's Disease	NA	NA	0.6	1.
P461.PWY..hexitol.fermentation.to.lactate..formate..ethanol.and.acetate	All Ulcerative Colitis	NA	NA	1.	1.
PANTO.PWY..phosphopantothenate.biosynthesis.I	All Crohn's Disease	NA	NA	1.	1.
PANTO.PWY..phosphopantothenate.biosynthesis.I	All Ulcerative Colitis	NA	NA	0.0613	1.
PANTOSYN.PWY..superpathway.of.coenzyme.A.biosynthesis.I..bacteria.	All Crohn's Disease	NA	NA	1.	1.
PANTOSYN.PWY..superpathway.of.coenzyme.A.biosynthesis.I..bacteria.	All Ulcerative Colitis	NA	NA	0.0646	1.
PENTOSE.P.PWY..pentose.phosphate.pathway	All Crohn's Disease	NA	NA	1.	1.
PENTOSE.P.PWY..pentose.phosphate.pathway	All Ulcerative Colitis	NA	NA	1.	1.
PEPTIDOGLYCANSYN.PWY peptidoglycan.biosynthesis.I..meso.diaminopimelate.containing.	All Crohn's Disease	NA	NA	1.	1.
PEPTIDOGLYCANSYN.PWY peptidoglycan.biosynthesis.I..meso.diaminopimelate.containing.	All Ulcerative Colitis	NA	NA	0.114	1.
PHOSLIPSYN.PWY..superpathway.of.phospholipid.biosynthesis.I..bacteria.	All Crohn's Disease	NA	NA	1.	1.
PHOSLIPSYN.PWY..superpathway.of.phospholipid.biosynthesis.I..bacteria.	All Ulcerative Colitis	NA	NA	0.25	1.
POLYAMSYN.PWY..superpathway.of.polyamine.biosynthesis.I	All Crohn's Disease	NA	NA	0.145	1.
POLYAMSYN.PWY..superpathway.of.polyamine.biosynthesis.I	All Ulcerative Colitis	NA	NA	0.121	1.
POLYISOPRENSYN.PWY..polyisoprenoid.biosynthesis..E..coli.	All Crohn's Disease	NA	NA	0.614	1.
POLYISOPRENSYN.PWY..polyisoprenoid.biosynthesis..E..coli.	All Ulcerative Colitis	NA	NA	0.559	1.
PPGPPMET.PWY..ppGpp.metabolism	All Crohn's Disease	NA	NA	1.	1.
PPGPPMET.PWY..ppGpp.metabolism	All Ulcerative Colitis	NA	NA	0.4	1.
PWY.1042..glycolysis.IV	All Crohn's Disease	NA	NA	1.	1.
PWY.1042..glycolysis.IV	All Ulcerative Colitis	NA	NA	0.209	1.
PWY.1269..CMP.3.deoxy.D.manno.octulosonate.biosynthesis	All Crohn's Disease	NA	NA	0.442	1.

PWY.1269..CMP.3.deoxy.D.manno.octulosonate.biosynthesis	All Ulcerative Colitis	NA	NA	0.516	1.
PWY.1861..formaldehyde.assimilation.II..assimilatory.RuMP.Cycle.	All Crohn's Disease	NA	NA	0.525	1.
PWY.1861..formaldehyde.assimilation.II..assimilatory.RuMP.Cycle.	All Ulcerative Colitis	NA	NA	1.	1.
PWY.241..C4.photosynthetic.carbon.assimilation.cycle..NADP.ME.type	All Crohn's Disease	NA	NA	0.23	1.
PWY.241..C4.photosynthetic.carbon.assimilation.cycle..NADP.ME.type	All Ulcerative Colitis	NA	NA	1.	1.
PWY.2941..L.lysine.biosynthesis.II	All Crohn's Disease	NA	NA	0.302	1.
PWY.2941..L.lysine.biosynthesis.II	All Ulcerative Colitis	NA	NA	0.154	1.
PWY.2942..L.lysine.biosynthesis.III	All Crohn's Disease	NA	NA	1.	1.
PWY.2942..L.lysine.biosynthesis.III	All Ulcerative Colitis	NA	NA	0.237	1.
PWY.3001..superpathway.of.L.isoleucine.biosynthesis.I	All Crohn's Disease	NA	NA	0.111	1.
PWY.3001..superpathway.of.L.isoleucine.biosynthesis.I	All Ulcerative Colitis	NA	NA	0.136	1.
PWY.3841..folate.transformations.II..plants.	All Crohn's Disease	NA	NA	1.	1.
PWY.3841..folate.transformations.II..plants.	All Ulcerative Colitis	NA	NA	0.134	1.
PWY.4041...gamma..glutamyl.cycle	All Crohn's Disease	NA	NA	0.151	1.
PWY.4041...gamma..glutamyl.cycle	All Ulcerative Colitis	NA	NA	1.	1.
PWY.4984..urea.cycle	All Crohn's Disease	NA	NA	0.605	1.
PWY.4984..urea.cycle	All Ulcerative Colitis	NA	NA	0.321	1.
PWY.5030..L.histidine.degradation.III	All Crohn's Disease	NA	NA	1.	1.
PWY.5030..L.histidine.degradation.III	All Ulcerative Colitis	NA	NA	0.206	1.
PWY.5097..L.lysine.biosynthesis.VI	All Crohn's Disease	NA	NA	1.	1.
PWY.5097..L.lysine.biosynthesis.VI	All Ulcerative Colitis	NA	NA	0.205	1.
PWY.5100..pyruvate.fermentation.to.acetate.and.lactate.II	All Crohn's Disease	NA	NA	0.198	1.
PWY.5100..pyruvate.fermentation.to.acetate.and.lactate.II	All Ulcerative Colitis	NA	NA	0.261	1.
PWY.5103..L.isoleucine.biosynthesis.III	All Crohn's Disease	NA	NA	0.367	1.
PWY.5103..L.isoleucine.biosynthesis.III	All Ulcerative Colitis	NA	NA	0.127	1.
PWY.5121..superpathway.of.geranylgeranyl.diphosphate.biosynthesis.II..via.MEP.	All Crohn's Disease	NA	NA	0.054	1.
PWY.5121..superpathway.of.geranylgeranyl.diphosphate.biosynthesis.II..via.MEP.	All Ulcerative Colitis	NA	NA	0.304	1.
PWY.5136..fatty.acid..beta..oxidation.II..plant.peroxisome.	All Crohn's Disease	NA	NA	0.231	1.
PWY.5154..L.arginine.biosynthesis.III..via.N.acetyl.L.citrulline.	All Crohn's Disease	NA	NA	1.	1.
PWY.5154..L.arginine.biosynthesis.III..via.N.acetyl.L.citrulline.	All Ulcerative Colitis	NA	NA	1.	1.
PWY.5188..tetrapyrrole.biosynthesis.I..from.glutamate.	All Crohn's Disease	NA	NA	0.451	1.
PWY.5188..tetrapyrrole.biosynthesis.I..from.glutamate.	All Ulcerative Colitis	NA	NA	1.	1.
PWY.5384..sucrose.degradation.IV..sucrose.phosphorylase.	All Crohn's Disease	NA	NA	0.125	1.
PWY.5384..sucrose.degradation.IV..sucrose.phosphorylase.	All Ulcerative Colitis	NA	NA	1.	1.
PWY.5484..glycolysis.II..from.fructose.6.phosphate.	All Crohn's Disease	NA	NA	0.0765	1.
PWY.5484..glycolysis.II..from.fructose.6.phosphate.	All Ulcerative Colitis	NA	NA	1.	1.
PWY.5659..GDP.mannose.biosynthesis	All Crohn's Disease	NA	NA	0.408	1.
PWY.5659..GDP.mannose.biosynthesis	All Ulcerative Colitis	NA	NA	1.	1.
PWY.5667..CDP.diacylglycerol.biosynthesis.I	All Crohn's Disease	NA	NA	1.	1.
PWY.5667..CDP.diacylglycerol.biosynthesis.I	All Ulcerative Colitis	NA	NA	0.11	1.
PWY.5676..acetyl.CoA.fermentation.to.butanoate.II	All Crohn's Disease	NA	NA	0.48	1.
PWY.5676..acetyl.CoA.fermentation.to.butanoate.II	All Ulcerative Colitis	NA	NA	0.43	1.
PWY.5686..UMP.biosynthesis.I	All Crohn's Disease	NA	NA	1.	1.
PWY.5686..UMP.biosynthesis.I	All Ulcerative Colitis	NA	NA	0.112	1.
PWY.5695..inosine.5..phosphate.degradation	All Crohn's Disease	NA	NA	1.	1.
PWY.5695..inosine.5..phosphate.degradation	All Ulcerative Colitis	NA	NA	1.	1.
PWY.5913..partial.TCA.cycle..obligate.autotrophs.	All Crohn's Disease	NA	NA	0.304	1.
PWY.5913..partial.TCA.cycle..obligate.autotrophs.	All Ulcerative Colitis	NA	NA	1.	1.
PWY.5941..glycogen.degradation.II	All Crohn's Disease	NA	NA	0.382	1.
PWY.5941..glycogen.degradation.II	All Ulcerative Colitis	NA	NA	0.201	1.
PWY.5973..cis.vaccenate.biosynthesis	All Crohn's Disease	NA	NA	1.	1.
PWY.5973..cis.vaccenate.biosynthesis	All Ulcerative Colitis	NA	NA	1.	1.
PWY.5981..CDP.diacylglycerol.biosynthesis.III	All Crohn's Disease	NA	NA	0.455	1.
PWY.5981..CDP.diacylglycerol.biosynthesis.III	All Ulcerative Colitis	NA	NA	0.801	1.
PWY.5989..stearate.biosynthesis.II..bacteria.and.plants.	All Crohn's Disease	NA	NA	1.	1.
PWY.5989..stearate.biosynthesis.II..bacteria.and.plants.	All Ulcerative Colitis	NA	NA	0.151	1.
PWY.6121..5.aminoimidazole.ribonucleotide.biosynthesis.I	All Crohn's Disease	NA	NA	1.	1.
PWY.6121..5.aminoimidazole.ribonucleotide.biosynthesis.I	All Ulcerative Colitis	NA	NA	0.126	1.
PWY.6122..5.aminoimidazole.ribonucleotide.biosynthesis.II	All Crohn's Disease	NA	NA	1.	1.
PWY.6122..5.aminoimidazole.ribonucleotide.biosynthesis.II	All Ulcerative Colitis	NA	NA	0.133	1.
PWY.6123..inosine.5..phosphate.biosynthesis.I	All Crohn's Disease	NA	NA	1.	1.
PWY.6123..inosine.5..phosphate.biosynthesis.I	All Ulcerative Colitis	NA	NA	0.13	1.
PWY.6124..inosine.5..phosphate.biosynthesis.II	All Crohn's Disease	NA	NA	0.281	1.
PWY.6124..inosine.5..phosphate.biosynthesis.II	All Ulcerative Colitis	NA	NA	0.141	1.
PWY.6125..superpathway.of.guanosine.nucleotides.de.novo.biosynthesis.II	All Crohn's Disease	NA	NA	1.	1.
PWY.6125..superpathway.of.guanosine.nucleotides.de.novo.biosynthesis.II	All Ulcerative Colitis	NA	NA	1.	1.
PWY.6126..superpathway.of.adenosine.nucleotides.de.novo.biosynthesis.II	All Crohn's Disease	NA	NA	1.	1.
PWY.6126..superpathway.of.adenosine.nucleotides.de.novo.biosynthesis.II	All Ulcerative Colitis	NA	NA	1.	1.
PWY.6147..6.hydroxymethyl.dihydropterin.diphosphate.biosynthesis.I	All Crohn's Disease	NA	NA	0.494	1.
PWY.6147..6.hydroxymethyl.dihydropterin.diphosphate.biosynthesis.I	All Ulcerative Colitis	NA	NA	0.526	1.
PWY.6151..S.adenosyl.L.methionine.salvage.I	All Crohn's Disease	NA	NA	0.404	1.
PWY.6151..S.adenosyl.L.methionine.salvage.I	All Ulcerative Colitis	NA	NA	0.0918	1.
PWY.6163..chorismate.biosynthesis.from.3.dehydroquinate	All Crohn's Disease	NA	NA	1.	1.
PWY.6163..chorismate.biosynthesis.from.3.dehydroquinate	All Ulcerative Colitis	NA	NA	0.0626	1.
PWY.6270..isoprene.biosynthesis.I	All Crohn's Disease	NA	NA	1.	1.
PWY.6270..isoprene.biosynthesis.I	All Ulcerative Colitis	NA	NA	0.0691	1.
PWY.6277..superpathway.of.5.aminoimidazole.ribonucleotide.biosynthesis	All Crohn's Disease	NA	NA	1.	1.
PWY.6277..superpathway.of.5.aminoimidazole.ribonucleotide.biosynthesis	All Ulcerative Colitis	NA	NA	0.133	1.
PWY.6282..palmitoleate.biosynthesis.I..from..5Z..dodec.5.enoate.	All Crohn's Disease	NA	NA	1.	1.
PWY.6282..palmitoleate.biosynthesis.I..from..5Z..dodec.5.enoate.	All Ulcerative Colitis	NA	NA	0.187	1.

PWY.6292..superpathway.of.L.cysteine.biosynthesis..mammalian.	All Crohn's Disease	NA	NA	1.	1.
PWY.6292..superpathway.of.L.cysteine.biosynthesis..mammalian.	All Ulcerative Colitis	NA	NA	0.185	1.
PWY.6305..superpathway.of.putrescine.biosynthesis	All Crohn's Disease	NA	NA	0.399	1.
PWY.6305..superpathway.of.putrescine.biosynthesis	All Ulcerative Colitis	NA	NA	0.6	1.
PWY.6317..D.galactose.degradation.I..Leloir.pathway.	All Crohn's Disease	NA	NA	1.	1.
PWY.6317..D.galactose.degradation.I..Leloir.pathway.	All Ulcerative Colitis	NA	NA	0.213	1.
PWY.6353..purine.nucleotides.degradation.II..aerobic.	All Crohn's Disease	NA	NA	1.	1.
PWY.6353..purine.nucleotides.degradation.II..aerobic.	All Ulcerative Colitis	NA	NA	1.	1.
PWY.6385..peptidoglycan.biosynthesis.III..mycobacteria.	All Crohn's Disease	NA	NA	1.	1.
PWY.6385..peptidoglycan.biosynthesis.III..mycobacteria.	All Ulcerative Colitis	NA	NA	0.127	1.
PWY.6386..UDP.N.acetylmuramoyl.pentapeptide.biosynthesis.II..lysine.containing.	All Crohn's Disease	NA	NA	1.	1.
PWY.6386..UDP.N.acetylmuramoyl.pentapeptide.biosynthesis.II..lysine.containing.	All Ulcerative Colitis	NA	NA	0.115	1.
PWY.6387..UDP.N.acetylmuramoyl.pentapeptide.biosynthesis.I meso.diaminopimelate.containing.	All Crohn's Disease	NA	NA	1.	1.
PWY.6387..UDP.N.acetylmuramoyl.pentapeptide.biosynthesis.I meso.diaminopimelate.containing.	All Ulcerative Colitis	NA	NA	0.118	1.
PWY.6507..4.deoxy.L.threo.hex.4.enopyranuronate.degradation	All Crohn's Disease	NA	NA	0.132	1.
PWY.6507..4.deoxy.L.threo.hex.4.enopyranuronate.degradation	All Ulcerative Colitis	NA	NA	0.593	1.
PWY.6519..8.amino.7.oxononanoate.biosynthesis.I	All Crohn's Disease	NA	NA	1.	1.
PWY.6519..8.amino.7.oxononanoate.biosynthesis.I	All Ulcerative Colitis	NA	NA	0.454	1.
PWY.6527..stachyose.degradation	All Crohn's Disease	NA	NA	0.478	1.
PWY.6527..stachyose.degradation	All Ulcerative Colitis	NA	NA	0.407	1.
PWY.6549..L.glutamine.biosynthesis.III	All Crohn's Disease	NA	NA	1.	1.
PWY.6549..L.glutamine.biosynthesis.III	All Ulcerative Colitis	NA	NA	1.	1.
PWY.6595..superpathway.of.guanosine.nucleotides.degradation..plants.	All Crohn's Disease	NA	NA	1.	1.
PWY.6595..superpathway.of.guanosine.nucleotides.degradation..plants.	All Ulcerative Colitis	NA	NA	0.412	1.
PWY.6606..guanosine.nucleotides.degradation.II	All Crohn's Disease	NA	NA	1.	1.
PWY.6606..guanosine.nucleotides.degradation.II	All Ulcerative Colitis	NA	NA	1.	1.
PWY.6607..guanosine.nucleotides.degradation.I	All Crohn's Disease	NA	NA	1.	1.
PWY.6607..guanosine.nucleotides.degradation.I	All Ulcerative Colitis	NA	NA	0.387	1.
PWY.6608..guanosine.nucleotides.degradation.III	All Crohn's Disease	NA	NA	1.	1.
PWY.6608..guanosine.nucleotides.degradation.III	All Ulcerative Colitis	NA	NA	1.	1.
PWY.6609..adenine.and.adenosine.salvage.III	All Crohn's Disease	NA	NA	1.	1.
PWY.6609..adenine.and.adenosine.salvage.III	All Ulcerative Colitis	NA	NA	1.	1.
PWY.6628..superpathway.of.L.phenylalanine.biosynthesis	All Crohn's Disease	NA	NA	0.0732	1.
PWY.6628..superpathway.of.L.phenylalanine.biosynthesis	All Ulcerative Colitis	NA	NA	0.17	1.
PWY.6630..superpathway.of.L.tyrosine.biosynthesis	All Crohn's Disease	NA	NA	0.272	1.
PWY.6630..superpathway.of.L.tyrosine.biosynthesis	All Ulcerative Colitis	NA	NA	0.325	1.
PWY.6700..queuosine.biosynthesis.I..de.novo.	All Crohn's Disease	NA	NA	1.	1.
PWY.6700..queuosine.biosynthesis.I..de.novo.	All Ulcerative Colitis	NA	NA	0.145	1.
PWY.6703..preQ0.biosynthesis	All Crohn's Disease	NA	NA	1.	1.
PWY.6703..preQ0.biosynthesis	All Ulcerative Colitis	NA	NA	0.221	1.
PWY.6731..starch.degradation.III	All Crohn's Disease	NA	NA	0.525	1.
PWY.6731..starch.degradation.III	All Ulcerative Colitis	NA	NA	0.172	1.
PWY.6823..molybdopterin.biosynthesis	All Crohn's Disease	NA	NA	1.	1.
PWY.6823..molybdopterin.biosynthesis	All Ulcerative Colitis	NA	NA	0.485	1.
PWY.6859..all.trans.farnesol.biosynthesis	All Crohn's Disease	NA	NA	0.113	1.
PWY.6859..all.trans.farnesol.biosynthesis	All Ulcerative Colitis	NA	NA	0.103	1.
PWY.6895..superpathway.of.thiamine.diphosphate.biosynthesis.II	All Crohn's Disease	NA	NA	0.601	1.
PWY.6895..superpathway.of.thiamine.diphosphate.biosynthesis.II	All Ulcerative Colitis	NA	NA	0.193	1.
PWY.6897..thiamine.diphosphate.salvage.II	All Crohn's Disease	NA	NA	1.	1.
PWY.6897..thiamine.diphosphate.salvage.II	All Ulcerative Colitis	NA	NA	0.189	1.
PWY.6901..superpathway.of.glucose.and.xylose.degradation	All Crohn's Disease	NA	NA	0.411	1.
PWY.6901..superpathway.of.glucose.and.xylose.degradation	All Ulcerative Colitis	NA	NA	1.	1.
PWY.6936..seleno.amino.acid.biosynthesis..plants.	All Crohn's Disease	NA	NA	0.107	1.
PWY.6936..seleno.amino.acid.biosynthesis..plants.	All Ulcerative Colitis	NA	NA	0.146	1.
PWY.6969..TCA.cycle.V..2.oxoglutarate.synthase.	All Crohn's Disease	NA	NA	0.534	1.
PWY.6969..TCA.cycle.V..2.oxoglutarate.synthase.	All Ulcerative Colitis	NA	NA	0.677	1.
PWY.7013...S..propane.1.2.diol.degradation	All Crohn's Disease	NA	NA	1.	1.
PWY.7013...S..propane.1.2.diol.degradation	All Ulcerative Colitis	NA	NA	0.295	1.
PWY.702..L.methionine.biosynthesis.II	All Ulcerative Colitis	NA	NA	0.0708	1.
PWY.7111..pyruvate.fermentation.to.isobutanol..engineered.	All Crohn's Disease	NA	NA	0.244	1.
PWY.7111..pyruvate.fermentation.to.isobutanol..engineered.	All Ulcerative Colitis	NA	NA	0.226	1.
PWY.7115..C4.photosynthetic.carbon.assimilation.cycle..NAD.ME.type	All Crohn's Disease	NA	NA	0.418	1.
PWY.7115..C4.photosynthetic.carbon.assimilation.cycle..NAD.ME.type	All Ulcerative Colitis	NA	NA	1.	1.
PWY.7117..C4.photosynthetic.carbon.assimilation.cycle..PEPCK.type	All Crohn's Disease	NA	NA	0.125	1.
PWY.7117..C4.photosynthetic.carbon.assimilation.cycle..PEPCK.type	All Ulcerative Colitis	NA	NA	1.	1.
PWY.7197..pyrimidine.deoxyribonucleotide.phosphorylation	All Crohn's Disease	NA	NA	1.	1.
PWY.7197..pyrimidine.deoxyribonucleotide.phosphorylation	All Ulcerative Colitis	NA	NA	1.	1.
PWY.7198..pyrimidine.deoxyribonucleotides.de.novo.biosynthesis.IV	All Crohn's Disease	NA	NA	1.	1.
PWY.7198..pyrimidine.deoxyribonucleotides.de.novo.biosynthesis.IV	All Ulcerative Colitis	NA	NA	0.608	1.
PWY.7199..pyrimidine.deoxyribonucleosides.salvage	All Crohn's Disease	NA	NA	1.	1.
PWY.7199..pyrimidine.deoxyribonucleosides.salvage	All Ulcerative Colitis	NA	NA	0.198	1.
PWY.7208..superpathway.of.pyrimidine.nucleobases.salvage	All Crohn's Disease	NA	NA	1.	1.
PWY.7208..superpathway.of.pyrimidine.nucleobases.salvage	All Ulcerative Colitis	NA	NA	1.	1.
PWY.7220..adenosine.deoxyribonucleotides.de.novo.biosynthesis.II	All Crohn's Disease	NA	NA	1.	1.
PWY.7220..adenosine.deoxyribonucleotides.de.novo.biosynthesis.II	All Ulcerative Colitis	NA	NA	1.	1.
PWY.7221..guanosine.ribonucleotides.de.novo.biosynthesis	All Crohn's Disease	NA	NA	1.	1.
PWY.7221..guanosine.ribonucleotides.de.novo.biosynthesis	All Ulcerative Colitis	NA	NA	0.0988	1.
PWY.7222..guanosine.deoxyribonucleotides.de.novo.biosynthesis.II	All Crohn's Disease	NA	NA	1.	1.

PWY.7222..guanosine.deoxyribonucleotides.de.novo.biosynthesis.II	All Ulcerative Colitis	NA	NA	1.	1.
PWY.7228..superpathway.of.guanosine.nucleotides.de.novo.biosynthesis.I	All Crohn's Disease	NA	NA	1.	1.
PWY.7228..superpathway.of.guanosine.nucleotides.de.novo.biosynthesis.I	All Ulcerative Colitis	NA	NA	1.	1.
PWY.7229..superpathway.of.adenosine.nucleotides.de.novo.biosynthesis.I	All Crohn's Disease	NA	NA	1.	1.
PWY.7229..superpathway.of.adenosine.nucleotides.de.novo.biosynthesis.I	All Ulcerative Colitis	NA	NA	1.	1.
PWY.7234..inosine.5..phosphate.biosynthesis.III	All Crohn's Disease	NA	NA	0.392	1.
PWY.7234..inosine.5..phosphate.biosynthesis.III	All Ulcerative Colitis	NA	NA	1.	1.
PWY.7238..sucrose.biosynthesis.II	All Crohn's Disease	NA	NA	0.474	1.
PWY.7238..sucrose.biosynthesis.II	All Ulcerative Colitis	NA	NA	0.154	1.
PWY.724..superpathway.of.L.lysine..L.threonine.and.L.methionine.biosynthesis.II	All Crohn's Disease	NA	NA	1.	1.
PWY.724..superpathway.of.L.lysine..L.threonine.and.L.methionine.biosynthesis.II	All Ulcerative Colitis	NA	NA	0.136	1.
PWY.7242..D.fructuronate.degradation	All Crohn's Disease	NA	NA	0.375	1.
PWY.7242..D.fructuronate.degradation	All Ulcerative Colitis	NA	NA	1.	1.
PWY.7282..4.amino.2.methyl.5.diphosphomethylpyrimidine.biosynthesis.II	All Crohn's Disease	NA	NA	0.396	1.
PWY.7282..4.amino.2.methyl.5.diphosphomethylpyrimidine.biosynthesis.II	All Ulcerative Colitis	NA	NA	1.	1.
PWY.7315..dTDP.N.acetylthomosamine.biosynthesis	All Crohn's Disease	NA	NA	0.829	1.
PWY.7315..dTDP.N.acetylthomosamine.biosynthesis	All Ulcerative Colitis	NA	NA	0.78	1.
PWY.7323<br>superpathway.of.GDP.mannose.derived.O.antigen.building.blocks.biosynthesis	All Crohn's Disease	NA	NA	0.55	1.
PWY.7323<br>superpathway.of.GDP.mannose.derived.O.antigen.building.blocks.biosynthesis	All Ulcerative Colitis	NA	NA	0.273	1.
PWY.7328<br>superpathway.of.UDP.glucose.derived.O.antigen.building.blocks.biosynthesis	All Crohn's Disease	NA	NA	1.	1.
PWY.7328<br>superpathway.of.UDP.glucose.derived.O.antigen.building.blocks.biosynthesis	All Ulcerative Colitis	NA	NA	1.	1.
PWY.7357..thiamine.phosphate.formation.from.pyrithiamine.and.oxythiamine..yeast.	All Crohn's Disease	NA	NA	1.	1.
PWY.7357..thiamine.phosphate.formation.from.pyrithiamine.and.oxythiamine..yeast.	All Ulcerative Colitis	NA	NA	0.199	1.
PWY.7392..taxadiene.biosynthesis..engineered.	All Crohn's Disease	NA	NA	0.115	1.
PWY.7392..taxadiene.biosynthesis..engineered.	All Ulcerative Colitis	NA	NA	0.171	1.
PWY.7400..L.arginine.biosynthesis.IV..archaebacteria.	All Crohn's Disease	NA	NA	1.	1.
PWY.7400..L.arginine.biosynthesis.IV..archaebacteria.	All Ulcerative Colitis	NA	NA	0.124	1.
PWY.7456...beta...1.4..mannan.degradation	All Crohn's Disease	NA	NA	0.299	1.
PWY.7456...beta...1.4..mannan.degradation	All Ulcerative Colitis	NA	NA	0.194	1.
PWY.7560..methylerythritol.phosphate.pathway.II	All Crohn's Disease	NA	NA	1.	1.
PWY.7560..methylerythritol.phosphate.pathway.II	All Ulcerative Colitis	NA	NA	0.0634	1.
PWY.7663..gondoate.biosynthesis..anaerobic.	All Crohn's Disease	NA	NA	1.	1.
PWY.7663..gondoate.biosynthesis..anaerobic.	All Ulcerative Colitis	NA	NA	0.513	1.
PWY.7664..oleate.biosynthesis.IV..anaerobic.	All Crohn's Disease	NA	NA	1.	1.
PWY.7664..oleate.biosynthesis.IV..anaerobic.	All Ulcerative Colitis	NA	NA	0.209	1.
PWY.7761..NAD.salvage.pathway.II..PNC.IV.cycle.	All Crohn's Disease	NA	NA	1.	1.
PWY.7761..NAD.salvage.pathway.II..PNC.IV.cycle.	All Ulcerative Colitis	NA	NA	0.52	1.
PWY.7790..UMP.biosynthesis.II	All Crohn's Disease	NA	NA	1.	1.
PWY.7790..UMP.biosynthesis.II	All Ulcerative Colitis	NA	NA	0.112	1.
PWY.7791..UMP.biosynthesis.III	All Crohn's Disease	NA	NA	1.	1.
PWY.7791..UMP.biosynthesis.III	All Ulcerative Colitis	NA	NA	0.112	1.
PWY.7851..coenzyme.A.biosynthesis.II..eukaryotic.	All Crohn's Disease	NA	NA	1.	1.
PWY.7851..coenzyme.A.biosynthesis.II..eukaryotic.	All Ulcerative Colitis	NA	NA	0.132	1.
PWY.7953..UDP.N.acetylmuramoyl.pentapeptide.biosynthesis.III<br>meso.diaminopimelate.containing.	All Crohn's Disease	NA	NA	1.	1.
PWY.7953..UDP.N.acetylmuramoyl.pentapeptide.biosynthesis.III<br>meso.diaminopimelate.containing.	All Ulcerative Colitis	NA	NA	0.172	1.
PWY.7977..L.methionine.biosynthesis.IV	All Crohn's Disease	NA	NA	0.207	1.
PWY.7977..L.methionine.biosynthesis.IV	All Ulcerative Colitis	NA	NA	0.23	1.
PWY.8004..Entner.Doudoroff.pathway.I	All Crohn's Disease	NA	NA	1.	1.
PWY.8004..Entner.Doudoroff.pathway.I	All Ulcerative Colitis	NA	NA	1.	1.
PWY.8131..5..deoxyadenosine.degradation.II	All Crohn's Disease	NA	NA	1.	1.
PWY.8131..5..deoxyadenosine.degradation.II	All Ulcerative Colitis	NA	NA	0.298	1.
PWY.8178..pentose.phosphate.pathway..non.oxidative.branch..II	All Crohn's Disease	NA	NA	1.	1.
PWY.8178..pentose.phosphate.pathway..non.oxidative.branch..II	All Ulcerative Colitis	NA	NA	1.	1.
PWY.8187..L.arginine.degradation.XIII..reductive.Stickland.reaction.	All Crohn's Disease	NA	NA	0.685	1.
PWY.8187..L.arginine.degradation.XIII..reductive.Stickland.reaction.	All Ulcerative Colitis	NA	NA	1.	1.
PWY.841..superpathway.of.purine.nucleotides.de.novo.biosynthesis.I	All Crohn's Disease	NA	NA	1.	1.
PWY.841..superpathway.of.purine.nucleotides.de.novo.biosynthesis.I	All Ulcerative Colitis	NA	NA	1.	1.
PWY.I9..L.cysteine.biosynthesis.VI..from.L.methionine.	All Ulcerative Colitis	NA	NA	0.0662	1.
PWY0.1061..superpathway.of.L.alanine.biosynthesis	All Crohn's Disease	NA	NA	0.425	1.
PWY0.1061..superpathway.of.L.alanine.biosynthesis	All Ulcerative Colitis	NA	NA	1.	1.
PWY0.1296..purine.ribonucleosides.degradation	All Crohn's Disease	NA	NA	1.	1.
PWY0.1296..purine.ribonucleosides.degradation	All Ulcerative Colitis	NA	NA	1.	1.
PWY0.1297..superpathway.of.purine.deoxyribonucleosides.degradation	All Crohn's Disease	NA	NA	0.274	1.
PWY0.1297..superpathway.of.purine.deoxyribonucleosides.degradation	All Ulcerative Colitis	NA	NA	0.326	1.
PWY0.1298..superpathway.of.pyrimidine.deoxyribonucleosides.degradation	All Crohn's Disease	NA	NA	0.262	1.
PWY0.1319..CDP.diacylglycerol.biosynthesis.II	All Crohn's Disease	NA	NA	1.	1.
PWY0.1319..CDP.diacylglycerol.biosynthesis.II	All Ulcerative Colitis	NA	NA	0.109	1.
PWY0.1477..ethanolamine.utilization	All Crohn's Disease	NA	NA	0.119	1.
PWY0.1479..tRNA.processing	All Crohn's Disease	NA	NA	1.	1.
PWY0.1479..tRNA.processing	All Ulcerative Colitis	NA	NA	0.521	1.
PWY0.1586..peptidoglycan.maturation..meso.diaminopimelate.containing.	All Crohn's Disease	NA	NA	0.363	1.
PWY0.1586..peptidoglycan.maturation..meso.diaminopimelate.containing.	All Ulcerative Colitis	NA	NA	0.197	1.
PWY0.162..superpathway.of.pyrimidine.ribonucleotides.de.novo.biosynthesis	All Crohn's Disease	NA	NA	1.	1.
PWY0.162..superpathway.of.pyrimidine.ribonucleotides.de.novo.biosynthesis	All Ulcerative Colitis	NA	NA	1.	1.

PWY0.845..superpathway.of.pyridoxal.5..phosphate.biosynthesis.and.salvage	All Crohn's Disease	NA	NA	0.343	1.
PWY0.845..superpathway.of.pyridoxal.5..phosphate.biosynthesis.and.salvage	All Ulcerative Colitis	NA	NA	0.57	1.
PWY0.862...5Z..dodecenoate.biosynthesis.I	All Crohn's Disease	NA	NA	1.	1.
PWY0.862...5Z..dodecenoate.biosynthesis.I	All Ulcerative Colitis	NA	NA	0.204	1.
PWY3O.4107..NAD.salvage.pathway.V..PNC.V.cycle.	All Crohn's Disease	NA	NA	0.233	1.
PWY3O.4107..NAD.salvage.pathway.V..PNC.V.cycle.	All Ulcerative Colitis	NA	NA	0.543	1.
PWY4FS.7..phosphatidylglycerol.biosynthesis.I..plastidic.	All Crohn's Disease	NA	NA	1.	1.
PWY4FS.7..phosphatidylglycerol.biosynthesis.I..plastidic.	All Ulcerative Colitis	NA	NA	0.578	1.
PWY4FS.8..phosphatidylglycerol.biosynthesis.II..non.plastidic.	All Crohn's Disease	NA	NA	1.	1.
PWY4FS.8..phosphatidylglycerol.biosynthesis.II..non.plastidic.	All Ulcerative Colitis	NA	NA	0.578	1.
PWY66.409..superpathway.of.purine.nucleotide.salvage	All Crohn's Disease	NA	NA	0.509	1.
PWY66.409..superpathway.of.purine.nucleotide.salvage	All Ulcerative Colitis	NA	NA	0.403	1.
PWY66.429..fatty.acid.biosynthesis.initiation..mitochondria.	All Crohn's Disease	NA	NA	1.	1.
PWY66.429..fatty.acid.biosynthesis.initiation..mitochondria.	All Ulcerative Colitis	NA	NA	0.205	1.
PYRIDNUCSAL.PWY..NAD.salvage.pathway.I..PNC.VI.cycle.	All Crohn's Disease	NA	NA	0.316	1.
PYRIDNUCSAL.PWY..NAD.salvage.pathway.I..PNC.VI.cycle.	All Ulcerative Colitis	NA	NA	0.456	1.
PYRIDNUCSYN.PWY..NAD.de.novo.biosynthesis.I..from.aspartate.	All Crohn's Disease	NA	NA	1.	1.
PYRIDNUCSYN.PWY..NAD.de.novo.biosynthesis.I..from.aspartate.	All Ulcerative Colitis	NA	NA	0.203	1.
PYRIDOXSYN.PWY..pyridoxal.5..phosphate.biosynthesis.I	All Crohn's Disease	NA	NA	0.357	1.
PYRIDOXSYN.PWY..pyridoxal.5..phosphate.biosynthesis.I	All Ulcerative Colitis	NA	NA	0.521	1.
RHAMCAT.PWY..L.rhamnose.degradation.I	All Crohn's Disease	NA	NA	0.0461	1.
RHAMCAT.PWY..L.rhamnose.degradation.I	All Ulcerative Colitis	NA	NA	1.	1.
RIBOSYN2.PWY..flavin.biosynthesis.I..bacteria.and.plants.	All Crohn's Disease	NA	NA	1.	1.
RIBOSYN2.PWY..flavin.biosynthesis.I..bacteria.and.plants.	All Ulcerative Colitis	NA	NA	0.348	1.
SALVADEHYPOX.PWY..adenosine.nucleotides.degradation.II	All Crohn's Disease	NA	NA	1.	1.
SALVADEHYPOX.PWY..adenosine.nucleotides.degradation.II	All Ulcerative Colitis	NA	NA	1.	1.
SER.GLYSYN.PWY..superpathway.of.L.serine.and.glycine.biosynthesis.I	All Crohn's Disease	NA	NA	0.278	1.
SER.GLYSYN.PWY..superpathway.of.L.serine.and.glycine.biosynthesis.I	All Ulcerative Colitis	NA	NA	0.21	1.
THISYNARA.PWY..superpathway.of.thiamine.diphosphate.biosynthesis.III..eukaryotes.	All Crohn's Disease	NA	NA	0.389	1.
THISYNARA.PWY..superpathway.of.thiamine.diphosphate.biosynthesis.III..eukaryotes.	All Ulcerative Colitis	NA	NA	0.153	1.
THRESYN.PWY..superpathway.of.L.threonine.biosynthesis	All Crohn's Disease	NA	NA	0.122	1.
THRESYN.PWY..superpathway.of.L.threonine.biosynthesis	All Ulcerative Colitis	NA	NA	0.167	1.
TRNA.CHARGING.PWY..tRNA.charging	All Crohn's Disease	NA	NA	1.	1.
TRNA.CHARGING.PWY..tRNA.charging	All Ulcerative Colitis	NA	NA	0.103	1.
UDPNAGSYN.PWY..UDP.N.acetyl.D.glucosamine.biosynthesis.I	All Crohn's Disease	NA	NA	0.347	1.
UDPNAGSYN.PWY..UDP.N.acetyl.D.glucosamine.biosynthesis.I	All Ulcerative Colitis	NA	NA	1.	1.
UNINTEGRATED	All Crohn's Disease	NA	NA	1.	1.
UNINTEGRATED	All Ulcerative Colitis	NA	NA	1.	1.
UNMAPPED	All Crohn's Disease	NA	NA	1.	1.
UNMAPPED	All Ulcerative Colitis	NA	NA	1.	1.
VALSYN.PWY..L.valine.biosynthesis	All Crohn's Disease	NA	NA	1.	1.
VALSYN.PWY..L.valine.biosynthesis	All Ulcerative Colitis	NA	NA	0.141	1.
X1CMET2.PWY..folate.transformations.III..E..coli.	All Crohn's Disease	NA	NA	0.332	1.
X1CMET2.PWY..folate.transformations.III..E..coli.	All Ulcerative Colitis	NA	NA	0.188	1.
